# Selective cleavage of unactivated arene ring C–C bonds by iridium: key roles of benzylic C–H activation and metal–metal cooperativity†

**DOI:** 10.1039/d0sc05900e

**Published:** 2021-01-12

**Authors:** Yancong Tian, Martin Jakoobi, Roman Boulatov, Alexey G. Sergeev

**Affiliations:** Department of Chemistry, University of Liverpool Crown Street Liverpool L69 7ZD UK boulatov@liverpool.ac.uk sergeev@liverpool.ac.uk

## Abstract

The cleavage of aromatic C–C bonds is central for conversion of fossil fuels into industrial chemicals and designing novel arene functionalisations through ring opening, expansion and contraction. However, the current progress is hampered by both the lack of experimental examples of selective oxidative addition of aromatic C–C bonds and limited understanding of the factors that favour insertion into the C–C rather than the C–H bonds. Here, we describe the comprehensive mechanism of the only reported chemo- and regioselective insertion of a transition metal into a range of substituted arene rings in simple iridium(i) complexes. The experimental and computational data reveal that this ring cleavage requires both reversible scission of a benzylic C–H bond and cooperativity of two Ir centres sandwiching the arene in the product-determining intermediate. The mechanism explains the chemoselectivity and scope of this unique C–C activation in industrially important methylarenes and provides a general insight into the role of metal–metal cooperativity in the cleavage of unsaturated C–C bonds.

## Abstract 2

The detailed mechanism of iridium-mediated C–C cleavage in unactivated arenes reveals the key factors enabling the process and helps predict the scope of the cleavage reaction.

## Graphical abstract

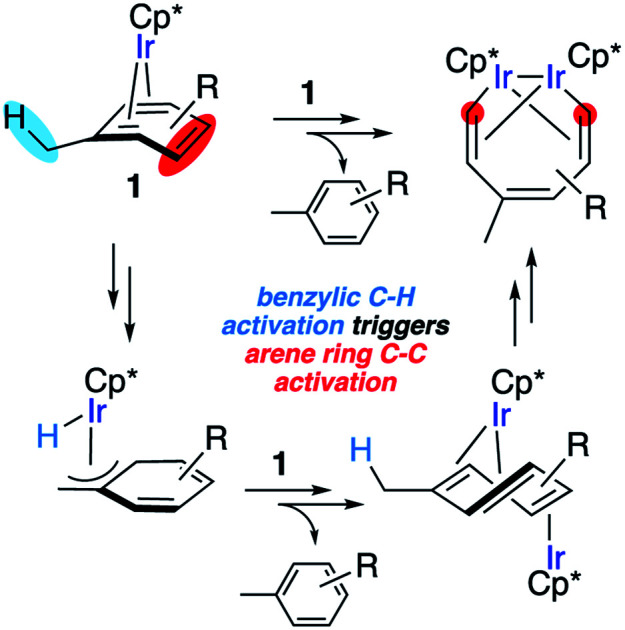

## Introduction

Arene functionalisations are a common route to many indispensable building blocks for organic synthesis.^1^ Most of these functionalisations rely on well-established activation of aromatic C–H bonds by metal complexes, which leave the aromatic system intact.^1–6^ In contrast, functionalisations that involve breaking the aromatic ring are rare despite their tremendous synthetic potential to provide convenient access to a range of ring opening, contraction and expansion products from cheap hydrocarbons.^7–10^ The main challenge in developing such transformations is the slow and unselective oxidative addition of aromatic C–C bonds due to their higher kinetic and thermodynamic stability compared to that of C–H bonds.^9,10^ As a result, insertion of metal complexes into aromatic C–H bonds is overwhelmingly more common that insertion into aromatic C–C bonds.

Of the seven examples of arene ring scission by a well-defined metal complexe reported to date,^11,12^ only cleavage of C_6_(CF_3_)_6_ by a Pt complex,^13^ and benzene and biphenylene by Al complexes^14,15^ proceed selectively. In all other cases,^16–19^ including reactions of Al complexes with alkylarenes,^19^ C–H scission competes with or even dominates the reaction.

To develop synthetic applications of aromatic C–C activation, factors that control the reactivity and selectivity and hence the substrate scope must be elucidated. Such understanding is currently lacking. The reported mechanistic studies of observed arene C–C cleavage are limited to benzene^16,20–22^ or quinoxaline^23–25^ and are exclusively computational. These DFT calculations focused primarily on C–C scission following reduction or dehydrogenation of the aromatic ring^20,21,23–25^ instead of the more fundamentally and synthetically important but little-understood C–C scission in intact arenes.^14,22,26^ Two known computationally identified examples of direct aromatic C–C activation in substituted arenes have never been realized experimentally, illustrating the challenges of integrating experimental and computational approaches in this area.^22,26^ As a result, a critical question of the role of substituents on the rate of aromatic C–C cleavage and the selectivity of C–C *vs.* C–H activation (and hence the scope) remains completely unexplored.

**Fig. 1:**
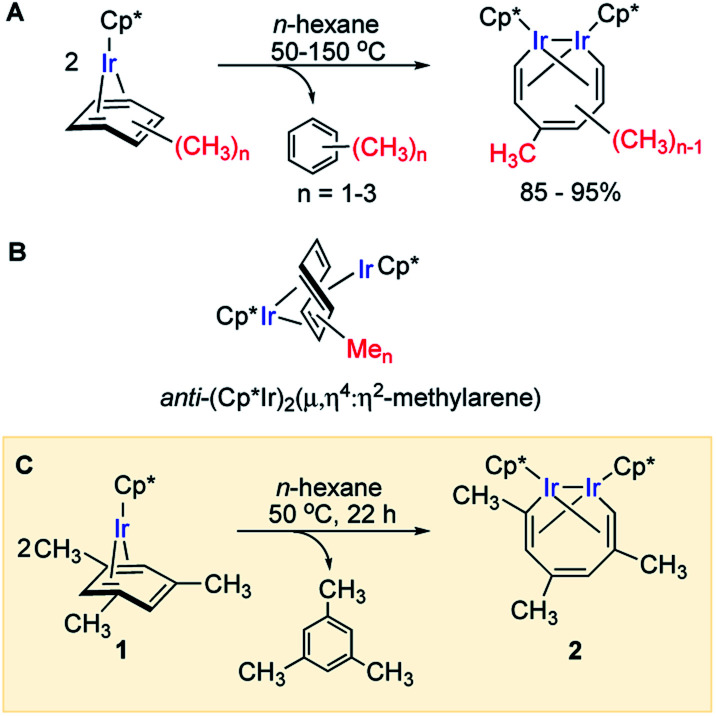
(A) Arene ring C–C scission in Cp*Ir(η^4^-methylarene) complexes. (B) The key diiridium intermediate. (C) Mesitylene ring scission in **1**.

We recently reported that simple Cp* iridium complexes cleave the arene ring of a range of industrially important unactivated arenes, including mesitylene, *o*-, *m*-, *p*-xylenes and toluene (Fig. 1A), but surprisingly not benzene. This C–C activation yields diiridium metallacycles with excellent yields and high regioselectivity without observable C–H activation products.^11,12^ Consequently, these Cp*Ir complexes provide the best starting point found so far both for developing practical strategies of arene functionalisations and for understanding the mechanism of C–C *vs.* C–H selectivity and the role of the substituents in enabling selective arene cleavage.

Here we describe the first mechanism of cleavage of arene ring C–C bonds in unactivated arenes of industrial importance that is consistent with both experimental (kinetic, isotope labelling, intermediate interception) and quantum-chemical data. Our study reveals the key role of benzylic C–H activation and metal–metal cooperativity for enabling this rare C–C oxidative addition. Analysis of the main and higher-energy reaction paths, including competing C–H activation steps, explains the unparalleled chemoselectivity and offers a straightforward model for explaining the scope of this C–C activation. Our results also provide an insight into the role of metal–metal cooperativity in activation of arene ring C–C bonds by highlighting the importance of *anti*- rather than *syn*-arrangement of the two cooperating metal centres. In a wider context, this geometrical requirement improves the understanding of a number of known cooperative C–C activations in other unsaturated molecules, where *anti*-arrangement was postulated, but not rationalised.^11,12,16,18,27–33^

## Results and Discussion

### Experimental kinetics and mechanistic observations

We previously observed that C–C bond activation in all Cp*Ir(η^4^-methylarene) complexes reported to date has a similar kinetics, which suggests a common mechanism.^11,12^ Among all these complexes, as a starting point for our experimental studies we chose Cp*Ir(η^4^-mesitylene) as a model substrate because this complex and the metallacycle product, **2**, each exists as a single isomer (Fig. 1C).

Kinetic measurements of C–C scission in **1** as 0.01–0.1 M solutions in cyclohexane-d_12_ at 40, 50, 60, 70 and 80 °C revealed first order in **1** and zero order in mesitylene during at least three reaction half times (Fig. S2 and S3†), with Δ*H*^≠^_o_ = 25.1 ± 2.1 kcal mol^−1^ and Δ*S*^≠^_o_ = −1.0 ± 6.5 cal (mol^−1^ K^−1^) derived from the Eyring plot, corresponding to Δ*G*^≠^ = 25.4 ± 3.0 kcal mol^−1^ at 50 °C (Fig. S5†). The observed first order rate law and a small Δ*S*^≠^_o_ are consistent with unimolecular rate-determining step (RDS) that does not involve dissociation of mesitylene. When **1** is heated in the presence of excess of mesitylene-d_3_, (CH_3_)_3_C_6_D_3_, no deuterium incorporation into **1** or the metallacycle product **2** is observed, which implies practically irreversible dissociation of mesitylene.

**Fig. 2:**
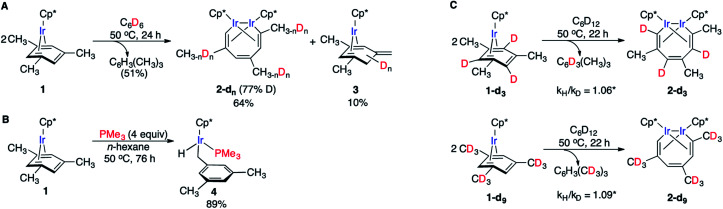
(A) H/D exchange and the formation of tautomer **3** upon heating **1** in C_6_D_6_. (B) Benzylic C–H activation upon heating of **1** in the presence PMe_3_. (C) The lack of KIE and intramolecular H/D scrambling upon cleavage of **1-d3** and **1-d9**. *KIEs were calculated from initial rates for separate reactions of the deuterated and non-deuterated species.

Several observations indicate the likely importance of benzylic C–H bond activation in conversion of **1** to **2**. First, heating **1** in benzene-d_6_ at 50 °C yielded metallacycle **2-d** (64%) with partially deuterated methyl groups of the broken arene ring and non-deuterated mesitylene (51%) as the main products after 24 h (Fig. 2A). Deuteration of **1** during this reaction was undetectable. Likewise, heating **2** in C_6_D_6_ at 50 °C for 24 h yielded no detectable amount of **2-dn**. Note that in neither experiment we observed deuteration of methyl groups of Cp* ligands. Second, thermolysis of **1** in the presence of excess PMe_3_ generated benzylic Ir hydride **4** as the main product (Fig. 2B) with no trace of metallacycle **2**. Such selective benzylic C–H bond scission in the presence of aromatic C–H bonds is unusual. Indeed, the C–H bond oxidative addition in alkylarenes typically affects aromatic C–H bonds^34–36^ and exclusive benzylic C–H cleavage mainly occurs in radical processes.^37^ Third, arene complexes lacking benzylic C–H bonds, *e.g.* Cp*Ir(η^4^-benzene), **5**, does not undergo C–C cleavage under similar conditions.^11^ Finally, a rare example^38^ of an arene tautomer (**3** in Fig. 2A), generated as a minor product of thermolysis of **1** in non-alkane solvents, is consistent with transient benzylic C–H bond activation. However, the negligible KIEs (1.06 ± 0.09 and 1.09 ± 0.09) measured in separate thermolysis experiments of **1** and its deuterated analogues **1-d3** and **1-d9** (Fig. 2C, S9, S10, Table S9†), suggest that C–H bonds are not cleaved in the RDS. The lack of detectable H/D scrambling between benzylic and arene ring hydrogens in the starting mesitylene complexes (**1-d3** and **1-d9**), the metallacycle products (**2-d3** and **2-d9**) or eliminated mesitylene, and the absence of C–C bond activation in Cp*Ir(η^4^-benzene) argue against activation of aromatic C–H bonds during conversion of **1** to metallacycle **2**.

### DFT calculations of the reaction mechanisms

The obtained experimental observations led us to three hypotheses, which we used for our computational search for the reaction mechanism:

A triple-decker *anti*-(Cp*Ir)_2_(μ,η^4^:η^2^-methylarene) intermediate involved in isomerization of Ir_2_-metallacycles (Fig. 1B)^12^ is also an intermediate in the conversion of **1** to **2**.

This diiridium species is formed from a coordinatively unsaturated mononuclear Cp*Ir(η^2^-arene) intermediate resulting from η^4^ → η^2^ sliding of the arene ligand.

The reaction requires cleavage of a benzylic, but not an aromatic C–H bond after the RDS to account for: (a) the generation of benzylic Ir hydride Cp*Ir(H)(η^1^-(CH_2_)C_6_H_3_Me_2_)(PMe_3_), **4** upon heating of **1** in the presence of PMe_3_ (Fig. 2B); (b) the lack of KIEs and H/D scrambling between benzylic and aromatic hydrogens; (c) the inertness of Cp*Ir(η^4^-benzene).

**Fig. 3:**
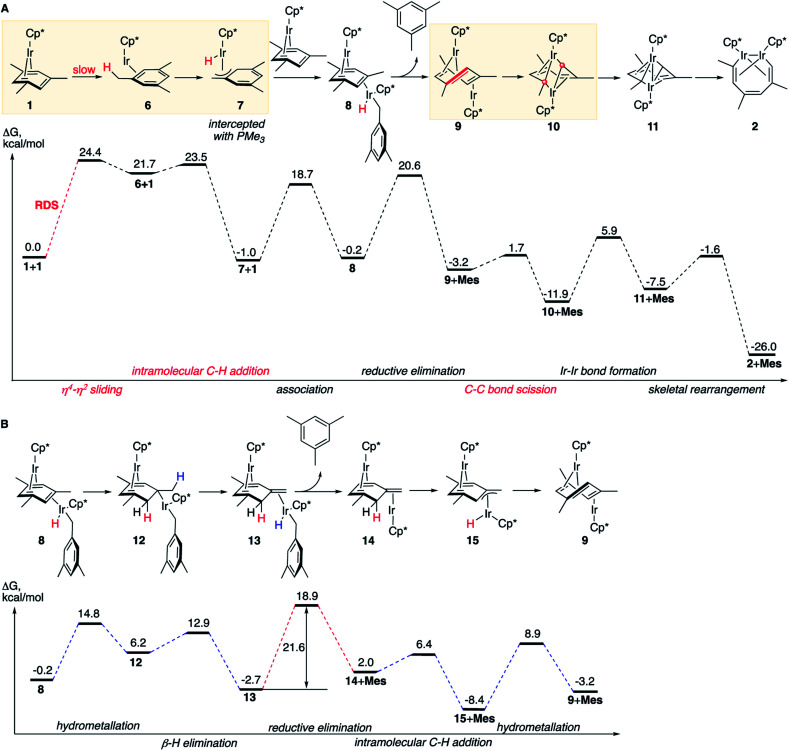
The calculated dominant reaction mechanisms and the corresponding free energy diagrams. (A) The simplest mechanism. (B) A competing path of the conversion of **8** to **9**. All free energies are relative to 2 moles of **1**, at M06-L/(6-311+G(d)+LANL2TZ)//B3LYP/(6-31G(d)+LANL2DZ), 1 M concentrations and 50 °C. **Mes** denotes mesitylene. Two red round legends indicate two carbon atoms involved in the C–C oxidative addition.

We performed all geometry optimizations, reaction path calculations and calculations of thermodynamic corrections with the B3LYP functional and a mixed basis set of LANL2DZ for Ir and 6-31G(d) for all other atoms, as recommended for calculations of activation barriers in reactions involving Ir–Ir and Ir–C bonds.^39–44^ To test the suitability of this model chemistry, we also reoptimized the lowest-energy conformers of the starting complex **1**, the final product **2**, the three highest-energy transition states and the two intermediates immediately preceding them in the main mechanisms (Fig. 3) at B3LYP-D3/def2SVP.^45^ This model chemistry likely yields a more realistic description of the electronic structure of organometallic Ir complexes, albeit at the considerable additional computational cost that precluded its use for all computations in this work. All relative electronic energies at B3LYP-D3/def2SVP were within 3 kcal mol^−1^ of those at B3LYP/(6-31G(d)+ LANL2DZ), Table S10.† The good agreement between the two sets of energies confirms that B3LYP/(6-31G(d)+ LANL2DZ) provides an appropriate balance of accuracy and performance to allow detailed enumeration of multiple reaction paths in multiple Cp*Ir(η^n^-arene) complexes, which distinguishes our current work from computational studies of arene C–C bond scission in the literature.^20,21,23–25^ We computed enthalpies and free energies by adding the thermodynamic corrections to the single-point energies calculated at the M06-L/(6-311+G(d)+LANL2TZ) level with a conductor polarizable continuum model (CPCM) of the reaction solvent.

Our calculations led to one lowest energy (dominant) mechanism that involves reversible benzylic C–H activation, and three higher energy mechanisms that occur *via* (a) aromatic C–H activation, (b) double benzylic and aromatic C–H activation and (c) without C–H activation. Comparison of these mechanisms presented below explains the observed scope of C–C scission in arenes, the role of metal–metal cooperativity and the observed exclusive formation of C–C, but not C–H activation products.

### The dominant reaction mechanism

The lowest energy mechanism is shown in Fig. 3A. The first step is the rate-determining isomerization of the starting complex **1** by η^4^ → η^2^ sliding of the mesitylene ligand with Δ*G*^≠^ of 24.4 kcal mol^−1^. The resulting high-energy coordinatively unsaturated intermediate Cp*Ir(η^2^-mesitylene), **6** undergoes facile Ir insertion into benzylic C–H bond of the coordinated mesitylene yielding hydride Cp*Ir(H)(η^3^-(CH_2_)C_6_H_3_Me_2_), **7**, with free-energy barrier of just 1.8 kcal mol^−1^. This complex binds to the uncoordinated C=C bond of starting complex Cp*Ir(η^4^-mesitylene), **1**, at its least-hindered face to form mixed-valent diiridium hydride **8** with the two Ir atoms on the opposite faces of the bridging mesitylene. Note that here the η^4^-coordinated arene acts as a strained cycloalkene ligand. This propensity of η^4^-arenes has been documented, *e.g.* in ring-opening metathesis.^46^ Conversion of **8** to the key diiridium sandwich intermediate *anti*-(Cp*Ir)_2_(μ,η^4^:η^2^-mesitylene), **9**, requires reductive elimination of mesitylene, which proceeds over nearly identical barriers in **8** and in its tautomer **13** (20.8 *vs.* 21.6 kcal mol^−1^, respectively). The latter forms rapidly from **8** by sequential hydrometallation (**8** to **12**) and β-hydrogen elimination (**12** to **13**, Fig. 3B) highlighting the ability of η^4^-arenes to mimic reactivity of strained alkenes such as norbornene and norbornadiene.^46^

At present we lack experimental estimates of the relative contributions to the reaction rate of the direct and stepwise (Fig. 3B) conversions of **8** to *anti*-(Cp*Ir)_2_(μ,η^4^:η^2^-mesitylene), **9**. As described in the next section, undetectable H/D scrambling in partially deuterated reactants (Fig. 2C) is consistent with both mechanisms, whereas observation of metallacycle **2** with partially deuterated Me groups upon heating **1** in C_6_D_6_ and the generation of tautomer **3** in non-alkane solvents (Fig. 2A) suggest the intermediacy of **13**.

The resulting diiridium(i) sandwich intermediate **9** undergoes dinuclear oxidative addition of an arene ring C–C bond in the bent bridging mesitylene ligand to give Ir(ii) complex **10** over the barrier of just 4.9 kcal mol^−1^. The subsequent formation of an Ir–Ir bond **10** and backbone reorganization in the resulting flyover complex **11** yields the product, **2**. Notably, C–C bond scission (**9** → **10**) involves one of the lowest activation barriers of the mechanism (4.9 kcal mol^−1^), considerably lower than those involving the formation of the Ir–Ir bond (17.8 kcal mol^−1^) or mesitylene elimination (>20 kcal mol^−1^), and is the lowest among reported calculated metal insertions into an arene (benzene) ring.^16,20–22^ Note that in contrast to what was proposed earlier^47^ neither η^4^-arene complex **1**, nor η^2^-arene complex **6** undergo direct insertion of iridium into the arene ring to give the corresponding iridacycloheptatriene as this insertion is kinetically prohibited under the reaction conditions (Δ*G*^≠^ > 40 kcal mol^−1^, Table S12†).

### Experimental validation of the computed mechanism

The first (unimolecular) step of the lowest energy reaction mechanism (Fig. 3) is rate determining, which tentatively agrees with the observed first order rate law. However, such direct comparison might be potentially misleading as the mechanism includes bimolecular and unimolecular steps, as well as competing paths for conversion of intermediate **8** to **9** with barriers of some steps only 3 kcal mol^−1^ lower than the barrier of RDS (24.4 kcal mol^−1^). To probe validity of the proposed mechanism, we calculated the time-dependent concentrations of all species in Fig. 3 by numerical integration of the underlying differential rate law using calculated barrier heights at starting concentrations of **1** of 0.01–0.1 M and of free mesitylene of 0–1 M, and the reaction temperatures of 50–150 °C. The results confirmed that the mechanisms in Fig. 3 reproduce the key kinetic observations: (a) the reaction rate is first order in **1** (Fig. S14†) and 0^th^ order in mesitylene (Fig. S15†); (b) The apparent activation parameters, from the Eyring plot of the calculated rate constants for the depletion of **1***vs.* the reaction time, are Δ*H*^≠^_o_ = 24.0 kcal mol^−1^ and Δ*S*^≠^_o_ = 3.1 cal (mol^−1^ K^−1^) *vs.* measured 25.1 ± 2.1 kcal mol^−1^ and Δ*S*^≠^_o_ = −1.0 ± 6.5 cal (mol^−1^ K^−1^) (Tables S7 and S20†); (c) no intermediate accumulates to a fraction that would make it detectable by ^1^H NMR (Fig. S13†).

**Fig. 4:**
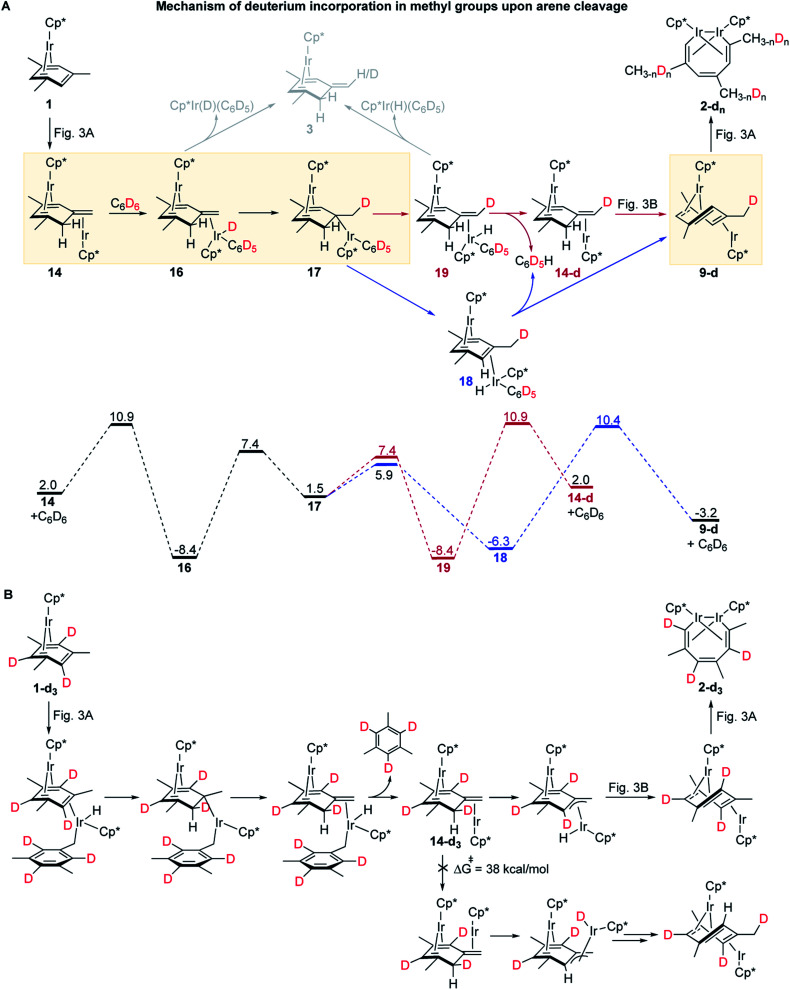
Calculated mechanisms of the observed isotope effects. (A) A competing path responsible for deuteration of **2** and the generation of a complex of a mesitylene tautomer, **3** upon thermolysis of **1** in C_6_D_6_. (B) The mechanistic origin of the high face-selectivity of H atom transfer between Ir and a Me group of the bridging mesitylene. All free energies are relative to 2 moles of **1**, at M06-L/(6-311+G(d)+LANL2TZ)//B3LYP/(6-31G(d)+LANL2DZ), 1 M concentrations and 50 °C.

The mechanisms in Fig. 3 also accommodate all observed isotope effects. First, consistent with the negligible experimental KIEs all steps involving the formation or scission of a C–H bond or Ir–H bond occur after the rate-limiting barrier. Second, the observed incorporation of D in metallacycle **2** during thermolysis of **1** in benzene-d_6_ is consistent with a kinetic competition of two reactions of arene–tautomer complex **14**: isomerisation to **9** and oxidative addition of C_6_D_6_ (Fig. 4A) to yield an Ir^III^(D)(C_6_D_5_) adduct, **16**. Retrotautomerisation of **16** to **17** deuterates the bridging mesitylene. Intermediate **17** then yields deuterated intermediate **9-d** by two competing mechanisms with loss of C_6_D_5_H (Fig. 4A; brown and blue paths). Third, the calculated high face-selectivity of C–H tautomerization (*e.g.*, **8** → **12** → **13**, Fig. 4B) ensures that the same H atom is transferred from Ir to a mesitylene sp^2^–C atom and back and prevents H/D scrambling in **1-d3** or **1-d9** (Fig. 2C) as observed. The lowest-energy path for the exchange of an aryl and a benzylic H atom requires rotation around the exocyclic C=C bond in **14-dn** (Fig. 4B) over a prohibitively high free energy barrier of 38 kcal mol^−1^. Finally, the lack of incorporation of D during thermolysis of **1** in the presence of mesitylene-d_3_, C_6_D_3_Me_3_, reflects the irreversible formation of *anti*-(Cp*Ir)_2_(μ,η^4^:η^2^-mesitylene), **9** (Δ*G*^≠^_9→8_ is 18.9 kcal mol^−1^ larger than Δ*G*^≠^_9→10_, Fig. 3A).

The formation of mesitylene tautomer complex **3**, a side product of thermolysis of **1** in non-alkane solvents, including benzene (Fig. 2A), is explained by facile and mildly endergonic dissociation of **16** and its isotopomer, **19** (Δ*G*^≠^ = 17.5 kcal mol^−1^, Δ*G*° = 4.1 kcal mol^−1^, Fig. 4A).

Finally, the formation of Cp*Ir(H)(η^1^-(CH_2_)C_6_H_3_Me_2_)(PMe_3_), **4** (Fig. 2B) instead of metallacycle **2** in the presence of PMe_3_ is consistent with the lower calculated barrier for the reaction of intermediate **7**, Cp*Ir(H)(η^3^-(CH_2_)C_6_H_3_Me_2_), with PMe_3_ (14.6 kcal mol^−1^) as opposed to the reaction of **7** with **1** (19.7 kcal mol^−1^). This suggests that PMe_3_ binds to **7** more than 10^4^-fold faster than to **1** and hence blocks the formation of diiridium intermediate **8** and subsequent C–C bond cleavage. Although an equimolar mixture of Cp*Ir(H)(η^1^-(CH_2_)C_6_H_3_Me_2_)(PMe_3_), **4**, and **1** is thermodynamically less stable by 4.1 kcal mol^−1^ than that of metallacycle **2**, PMe_3_ and mesitylene, such conversion is too slow (overall ΔG^≠^ = 35.5 kcal mol^−1^) to occur on the experimental timescale at 50 °C.

### Alternative reaction paths

We found it beneficial to systematically search for alternative paths connecting **1** and **2**, for four reasons: (1) to confirm that the reaction mechanisms discussed above (Fig. 3A and B) comprise the lowest-energy paths; (2) to understand why paths occurring through transient benzylic C–H activation, rather than aromatic C–H activation or without C–H bond scission are uniquely efficient at arene C–C cleavage; (3) to explain the observed exclusive C–C *vs.* C–H chemoselectivity; (4) to identify other factors that promote or block arene C–C bond scission at metal complexes and hence could help predict the feasibility of the cleavage.

**Fig. 5:**
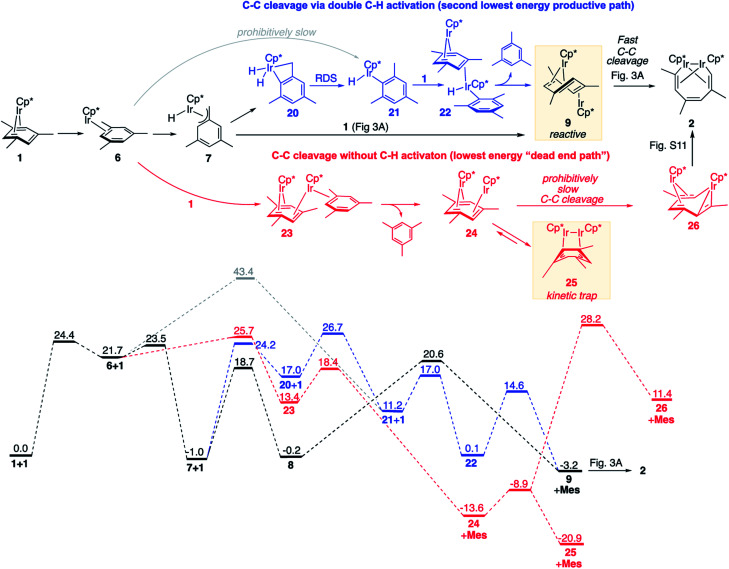
Comparison of the lowest energy path (in black; see Fig. 3 for details) and less favourable reaction paths for arene ring cleavage with and without C–H activation (in blue and red, respectively). All free energies are relative to 2 moles of **1**, at M06-L/(6-311+G(d)+LANL2TZ)//B3LYP/(6-31G(d)+LANL2DZ), 1 M concentrations and 50 °C. **Mes** denotes mesitylene.

We identified the next lowest-barrier path for arene ring cleavage (Fig. 5, blue sequence), which occurs *via* a double C–H activation and has the highest barrier of only 3.3 kcal mol^−1^ higher than that in the main mechanism (Fig. 3A and B). This path also involves benzylic C–H activation, but the resulting benzylic hydride Cp*Ir(H)(η^3^-(CH_2_)C_6_H_3_Me_2_), **7**, isomerises to an aryl hydride, Cp*Ir(H)(η^1^-C_6_H_3_Me_2_), **21**, by surprisingly facile intramolecular oxidative addition of an arene C–H bond to yield an Ir^V^ dihydride **20** followed by the rate-limiting C–H bond forming reductive elimination. Subsequent binding of **1** and reductive elimination of mesitylene yields the key intermediate *anti*-(Cp*Ir)_2_(μ,η^4^:η^2^-mesitylene), **9**, with overall Δ*G*^≠^ of 27.7 kcal mol^−1^ (Fig. 5) *vs.* 24.4 kcal in the main mechanism (Fig. 3A and B). The minor contribution of this path to the overall kinetics is consistent with its high estimated KIE (∼1.6) not being observed experimentally.

Preferential formation of benzylic hydride **7** as compared to aryl hydride **21** illustrates the unusual reactivity of the Cp*Ir moiety towards C–H bonds. First, oxidative addition of the benzylic C–H bond in Cp*Ir(η^2^-mesitylene), **6** → **7**, is considerably faster than direct oxidative addition of an aromatic C–H bond, **6** → **21**, because it requires traversal of the barrier of 1.8 kcal mol^−1^*vs.* 21.7 kcal mol^−1^. Second, the benzylic adduct Cp*Ir(H)(η^3^-(CH_2_)C_6_H_3_Me_2_), **7**, is >12 kcal mol^−1^ more stable than the aryl analogue **21**. This selectivity is orthogonal to that of a more common Cp*Ir(PMe_3_) fragment^34^ and is rare in C–H activation,^36,48–51^ but appears to be essential for the observed reactivity. It is important to note that both benzylic and aromatic C–H activation intermediates (**7** and **21**) are not kinetically stable under reaction conditions and convert into products of C–C cleavage as shown in Fig. 3 and 5. That is, C–H activation here facilitates, rather than overrides arene ring C–C activation, which contrasts to what is typically seen in classical organometallic chemistry.^1–6^ For example, in the recently reported Al(i)-mediated arene activation, the metal smoothly inserts into ring C–C bond in reaction with benzene *via* a transient η^2^-arene complex,^14^ while with xylenes C–H activation occurs exclusively and completely blocks the C–C cleavage.^19^

We also identified one lower-energy reaction path that does not involve C–H activation and does not cause the C–C scission. Consideration of this unproductive path is important for predicting the feasibility of C–C cleavage as shown in the following section. This path instead generating the reactive *anti*-(Cp*Ir)_2_(μ,η^4^:η^2^-mesitylene) intermediate **9** gives its inert *syn*-(Cp*Ir)_2_(μ,η^3^:η^3^-mesitylene) isomer **25** (Fig. 5, red sequence). This route starts with association of **1** and its unsaturated isomer Cp*Ir(η^2^-mesitylene), **6**, over the free energy barrier of 4.0 kcal mol^−1^ to give *syn*-Cp*Ir(μ,η^4^:η^2^-mesitylene)Ir(η^2^-mesitylene)Cp* (**23**), in which both Ir atoms are on the same face of the bridging mesitylene. Facile elimination of η^2^-mesitylene generates *syn*-(Cp*Ir)_2_(μ,η^4^:η^2^-mesitylene), **24**, which is in a rapid equilibrium with the Ir–Ir bonded complex *syn*-(Cp*Ir)_2_(μ,η^3^:η^3^-mesitylene), **25** (Δ*G*^≠^ = 4.7 kcal mol^−1^, Δ*G*° = −7.3 kcal mol^−1^). Our reaction path calculations suggest that the two *syn*-diiridium isomers **24** and **25** and the product of C–C bond scission, **26**, are connected by a single ambimodal^52^ transition state with an additional, lower-energy, transition state separating **24** and **25**. The very high barriers separating **25** from either **1** or **26** (46.6 and 49.1 kcal mol^−1^, respectively) means that **25** is a kinetic trap preventing arene cleavage. The calculated high kinetic and thermodynamic stability of **25** is consistent with that of other group 9 *syn* bridging arene complexes being sufficiently stable to be isolated.^53,54^

Note that in thermolysis of Cp*Ir(η^4^-mesitylene), **1**, *syn*-(Cp*Ir)_2_(μ,η^3^:η^3^-mesitylene), **25**, is not observed, despite the rate-limiting barrier for its formation (**6** → **23**, red path, Fig. 5) being only 1.3 kcal mol^−1^ higher than that of the main mechanism (**1** → **6**). The reason is that unimolecular isomerization of **6** to **7** over an 1.8 kcal mol^−1^ barrier is >10^4^-faster than diffusion-limited bimolecular binding of **6** to **1**. As mentioned above, the latter is necessary to yield the inert *syn* diiridium isomer **25***via* the red path (Fig. 5). This “dead-end” path appears to explain the inertness of arene complexes lacking benzylic C–H bonds.^11^ In particular, our detailed calculations suggest that this path has the lowest energy for thermolysis of benzene complex **5** (Fig. 5 and S12†).

### Factors enabling cleavage of arene ring C–C bonds

**Fig. 6:**
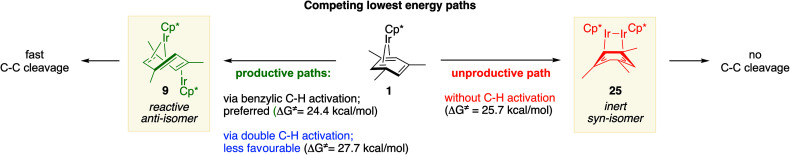
Summary of the lowest energy paths for thermolysis of **1**.

The obtained data, summarised in Fig. 6, suggest that susceptibility of Cp*Ir(η^4^-arene) to arene cleavage is determined by the kinetic competition between paths leading to the *syn* and *anti* isomers of bridging arene complexes (Cp*Ir)_2_(μ-arene). Only the *anti* isomer enables arene cleavage and it does this at least in two ways.

First, sandwiching the arene ring between two Ir atoms appears to enable cooperative C–C scission. The barrier for such scission in *anti*-(Cp*Ir)_2_(μ,η^4^:η^2^-mesitylene), **9**, 4.5 kcal mol^−1^, is considerably lower than that in the *syn* analogue, **24**, (41.8 kcal mol^−1^) or the barrier separating Cp*Ir(η^4^-mesitylene) from the corresponding iridacyloheptatriene (46.1 kcal mol^−1^) (Table S12†). The relative closeness of the last two numbers suggests that coordination of two Ir atoms to the same arene by itself labilises the arene C–C bonds negligibly. A similar difference is calculated in benzene and *m*-xylene complexes (Table S11†). The origin of this diiridium cooperativity remains to be established, but the *anti* geometry appears to enable more bonding Ir–C contacts (Ir–C distance <2.1 Å) in the transition state of C–C bond scission (two per each Ir) than either the *syn* analogue or Cp*Ir(η^4^-mesitylene) (two in each). Metal–metal cooperativity is increasingly recognized as a key factor in enabling difficult organic transformations at discrete metal complexes.^55,56^ Although the role of metal–metal cooperativity in arene C–C bond activation has received little attention, previously reported C–C oxidative additions in benzene,^16^ biphenylene,^15^ cyclopentadienyl^18^ and cyclooctatetraene ligands^28,29^ suggested the involvement of reactive intermediates with the *anti* arrangement of the two metal centers. The difference in reactivities of *anti* and *syn* isomers of these intermediates, however, have not been studied, and the role of the *anti* geometry in enabling cooperativity in C–C bond scission remains to be enumerated.

Second, the *anti* geometry prevents the formation of the Ir–Ir bond prior to scission of the arene C–C bond: when this Ir–Ir bond forms with intact arene, as occurs in the *syn* isomer, the resulting intermediate (*e.g.*, **25** in Fig. 5 and S11†) is too stable to react further. In contrast, the formation of metal–metal bond is usually thought to facilitate rather than hamper the metal-assisted C–C cleavage in arenes,^11,12,16,18^ and other aromatic and unsaturated hydrocarbons (biphenylene,^27^ cyclooctatetraene,^28,29,57,58^ and cyclopentadienyl^18^ anions, cycloalkenes^30^ and alkynes^31–33^).

**Fig. 7:**
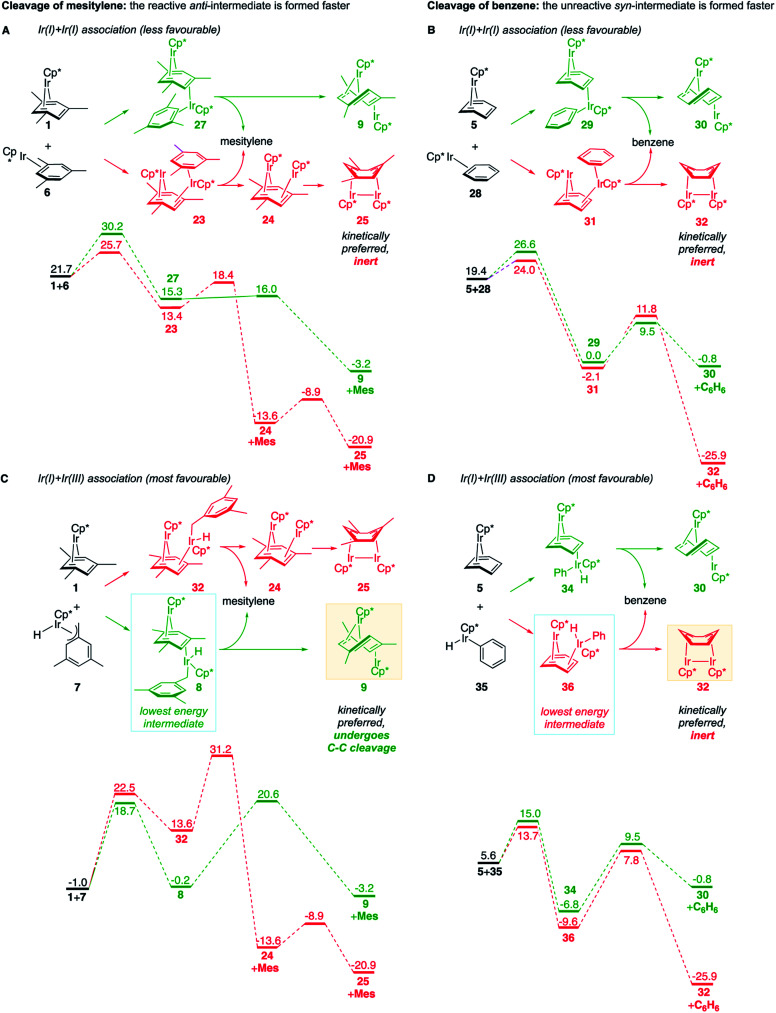
Calculated competing mechanisms of the formation of *syn* and *anti* diiridium intermediates by association of reactant Cp*Ir(η^4^-arene) with either Ir^I^ (A and B) or Ir^III^ (C and D) intermediate in thermolysis of Cp*Ir(η^4^-mesitylene) (A and C) or Cp*Ir(η^4^-benzene) (B and D). The free energies are relative to two moles of Cp*Ir(η^4^-arene) in all schemes. Pathways in green and red lead to the reactive and unreactive intermediates, respectively. The boxed structures are the predicted intermediates. All free energies are at M06-L/(6-311+G(d)+LANL2TZ)//B3LYP/(6-31G(d)+LANL2DZ), 1 M concentrations and 50 °C, relative to 2 moles of **1** (A and C) or of Cp*Ir(η^4^-C_6_H_6_) (B and D).

These roles of the *anti*-Ir_2_ intermediate in determining the outcome of thermolysis of Cp*Ir(arene) complexes appears to be general, as suggested by our calculations on benzene and *m*-xylene analogues of **1** (Table S12†). In other words, the observed cleavage of methylarenes reflects the kinetic selectivity for the formation of the *anti* isomers, which occurs after the RDS. To understand factors that determine the *anti*/*syn* selectivity and hence the range of cleavable arenes, we compare in Fig. 7 two paths leading to each *anti*- and *syn*-(Cp*Ir)_2_(μ-arene) for mesitylene, which is cleaved, and benzene, which is not. Four of these eight paths involved association of reactant Cp*Ir(η^4^-arene) with its coordinatively unsaturated Cp*IrI(η^2^-arene) isomer (Fig. 7A and B). The other four proceeded by association of Cp*Ir(η^4^-arene) with the corresponding product of oxidative addition of the C–H bond, Cp*Ir(aryl)(H) (Fig. 7C and D).

Comparison of these mechanisms for the cleavage of mesitylene and benzene highlights the key role that facile activation of benzylic C–H bond plays in arene scission in Cp*Ir(η^4^-arene) complexes. Fig. 7 shows that only association of Cp*Ir(η^4^-mesitylene), **1**, with Ir^III^ intermediate, Cp*Ir(H)(η^3^-CH_2_C_6_H_3_Me_3_), **7**, yields reactive *anti*-(Cp*Ir)_2_(μ-mesitylene), **9** by way of intermediate **8** (Fig. 7C). In all other scenarios, the formation of *syn*-diiridium intermediates is favoured both kinetically and thermodynamically, *e.g.*, **1** + **6** → **23***vs.***27** (Fig. 7A), **5** + **28** → **31***vs.***29** (Fig. 7B) and **5** + **35** → **34***vs.***36** (Fig. 7D). Because intermediate **7** is formed from intermediate **6**, the productive path (**7** → **9**) is only accessible if conversion of **6** to **7** is faster than addition of **6** to **1**. In other words, the unusually fast intramolecular oxidative addition of a benzylic C–H bond in Cp*Ir(η^2^-methylarene) enables C–C bond cleavage by outcompeting bimolecular addition of the same intermediate to Cp*Ir(η^4^-arene).^59^

### Model for predicting the scope of the C–C cleavage

Fig. 7, which summarises calculated reaction paths for the arene cleavage in mesitylene **1** and benzene **5** complexes, demonstrates that an arene C–C bond is cleaved only if the mixed-valence Ir(i)Ir(iii) *anti*-intermediate (*e.g.*, **8** for mesitylene and **36** for benzene) is formed faster than any of its three analogues (*syn* Ir(i)Ir(iii) and *syn* or *anti* Ir(i)Ir(i)). In each case, the most stable of the diiridium intermediates also forms fastest. For example, in C–C cleaving thermolysis of **1** (Fig. 7A and C), productive intermediate **8** (*anti*-Ir(i)Ir(iii)) is both most stable and is formed *via* the lowest barrier. Conversely, in thermolysis of the benzene analogue **5** (Fig. 7B and D), **36**, *syn*-Ir(i)Ir(iii), is the most stable and fastest forming diiridium intermediate which gives unproductive complex **32** inert towards C–C cleavage. In either case, *anti*- or *syn*-Ir(i)Ir(iii) intermediates form faster than their Ir(i)Ir(i) isomers.

**Fig. 8:**
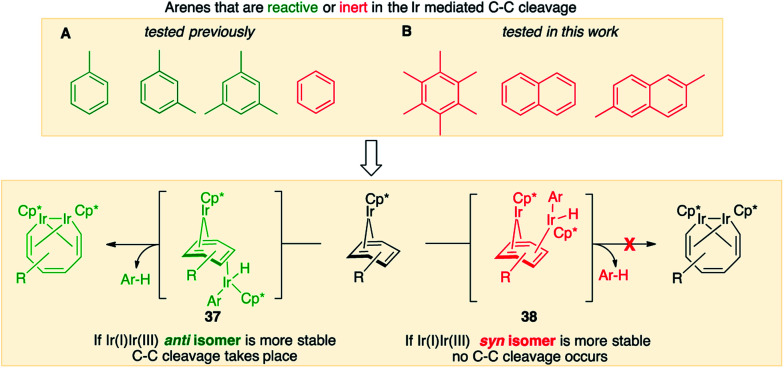
Computational model for predicting the feasibility Ir mediated arene C–C cleavages reported previously (A) and in this work (B).

**Fig. 9:**
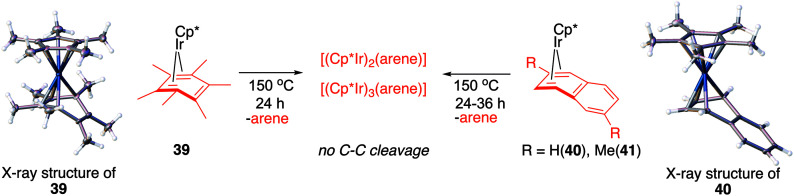
Attempted Ir-mediated cleavage of hexamethylbenzene, naphthalene and 1,6-dimethylnaphthalene complexes confirming the feasibility of the proposed model.

We hypothesised that calculation of relative energies of *anti*- and *syn*-Ir(i)–Ir(iii) intermediates (Fig. 8; **37** and **38**) can be used to predict the feasibility of the arene C–C cleavage. To test this, we first calculated the relative energy of **37** and **38** for two additional arenes, toluene and *m*-xylene, earlier shown to undergo cleavage (Fig. 8A).^11^ In both cases, *anti* isomers were found to be most stable, which agrees with experimental data (Table 1). Next, we first predicted and then experimentally tested the reactivity of three new experimentally untested unactivated arenes: fully methylated benzene as well as naphthalene and 2,6-dimethylnaphthalene (Fig. 8B). For all these arenes unproductive *syn* isomers **38** had lower energy than productive *anti***37** (Table 1) suggesting inertness toward C–C scission. We prepared the corresponding Cp*Ir(η^4^-arene) complexes **39–41** and characterised the C_6_Me_6_ (**39**) and naphthalene (**40**) complexes by X-ray (Fig. 9). Thermolysis of **39–41** at 150 °C for 24–36 h led to consumption of the starting complexes and expected release of some free arene, but no arene C–C cleavage was observed. ^1^H NMR and HR-MS spectra of the products indicated formation of inert *syn-*(Cp*Ir)_2_(arene) and (Cp*Ir)_3_(arene) complexes, which is in agreement with our theoretical predictions. The lack of C–C bond scission in **39** and **41** suggests that the presence of benzylic C–H bonds alone is insufficient to enable C–C bond scission and highlights the validity of the proposed model. Although the exact factors that determine the relative stabilities of the *syn*- and *anti*-diiridium intermediates have yet to be identified,^60^ steric effects likely to play an important role here. With the exception of the two naphthalenes, the less-stable Ir(i)Ir(iii) intermediates have more short nonbonding H⋯H contacts (<2.60 Å) than their more stable analogues (Table 1).

**Table 1:** Predicting iridium-induced arene ring cleavage in unactivated arenes using the electronic energies of diiridium bridging arene intermediates

Ar	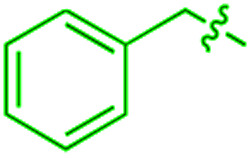	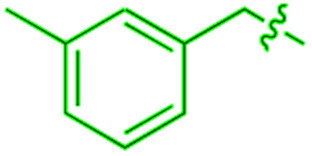	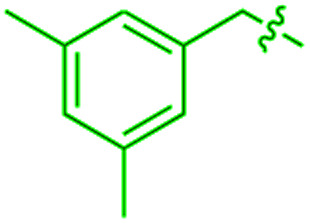	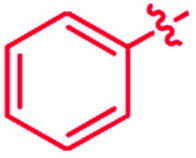	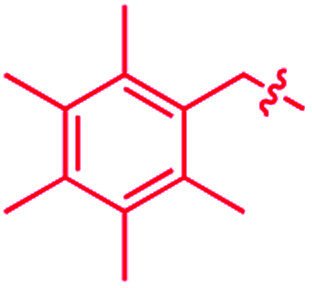	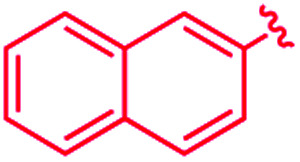	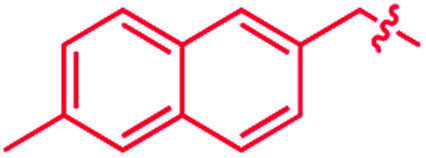
Δ*E*(*syn*/*anti*), kcal mol^−1^^a^	2.5	9.7	12.3	−1.6	−0.3	−2.0	−9.7
Excess of short H⋯H contacts^b^ in *syn* (**38**) *vs. anti* (**37**) intermediates	1	2	4	−1	−3	4	1
Arene cleavage expected?	Yes	Yes	Yes	No	No	No	No
Observed?	Yes	Yes	Yes	No	No	No	No

a The electronic energy of *syn*-Ir(i)Ir(iii) intermediate **38** relative to that of *anti*-Ir(i)Ir(iii) analogue, **37** at M06-L/6-311+G(d)+LANL2TZ)//B3LYP/(6-31G(d)+LANL2DZ)).

b All short (<2.60 Å) interactions between any two hydrogen atoms located in different ligands (*e.g.* between Ar and Cp*, Ar and ArH *etc.*).

## Conclusions

In summary, we presented here a comprehensive mechanistic analysis of the most selective and general oxidative addition of arene ring C–C bonds known to date, so far observed only in Cp*Ir(η^4^-arene) complexes. The obtained results suggest that the unique capacity of the Cp*Ir system to cleave methylated arene rings selectively results from remarkably facile reversible scission of a benzylic C–H bond. This favours the formation of the *anti*-(Cp*Ir)_2_(μ-methylarene) intermediate that undergoes fast ring C–C scission over its more thermodynamically stable and inert *syn* isomer. The higher reactivity of the *anti*-intermediate results from the cooperative action of the two Ir atoms that lowers the barrier for the C–C bond scission to less than 5 kcal mol^−1^ compared to more than 40 kcal mol^−1^ in the *syn* isomer. The analysis of a range of reaction pathways in the mesitylene and benzene complexes suggests that the occurrence of C–C scission in a specific arene can be predicted by comparing the electronic energies of just two isomeric intermediates for each arene. These energies correlate with the relative rates for the formation of the reactive *anti* and inert *syn* diiridium intermediates. Because these energies are easily calculated, they provide a useful model for predicting the scope of the process. Application of this approach successfully explained the experimentally observed C–C scission in mesitylene, *m*-xylene and toluene and the lack of C–C scission in benzene, hexamethylbenzene, naphthalene and 2,6-dimethylnaphthalene. The rate-determining step for this cleavage is η^4^ to η^2^ sliding of the arene ligand in the starting complex and further investigation of this step will provide further means to control this process.

The high reactivity of the resulting Cp*Ir(η^2^-arene) intermediate in C–H oxidative addition discovered during our mechanistic studies offers additional opportunities for developing novel functionalisation methods based both on selective arene C–C and C–H bond scissions.

## Conflicts of interest

There are no conflicts to declare.

## Supplementary Material

SC-012-D0SC05900E-s001

SC-012-D0SC05900E-s002

## References

[cit1] Arene chemistry: Reaction Mechanisms and Methods for Aromatic Compounds, ed. J. Mortier, Wiley, Hoboken, 2016

[cit2] Kuhl N., Hopkinson M. N., Wencel-Delord J., Glorius F. (2012). Angew. Chem., Int. Ed. Engl..

[cit3] Gensch T., Hopkinson M. N., Glorius F., Wencel-Delord J. (2016). Chem. Soc. Rev..

[cit4] Hartwig J. F., Larsen M. A. (2016). ACS Cent Sci..

[cit5] Wedi P., van Gemmeren M. (2018). Angew. Chem., Int. Ed..

[cit6] Kancherla S., Jørgensen K. B., Fernández-Ibáñez M. A. (2019). Synthesis.

[cit7] Reisman S. E., Nani R. R., Levin S. (2011). Synlett.

[cit8] Chen P. H., Billett B. A., Tsukamoto T., Dong G. (2017). ACS Catal..

[cit9] Jakoobi M., Sergeev A. G. (2019). Chem.–Asian J..

[cit10] Chan A. P. Y., Sergeev A. G. (2020). Coord. Chem. Rev..

[cit11] Jakoobi M., Halcovitch N., Whitehead G. F. S., Sergeev A. G. (2017). Angew. Chem., Int. Ed..

[cit12] Jakoobi M., Tian Y. C., Boulatov R., Sergeev A. G. (2019). J. Am. Chem. Soc..

[cit13] Browning J., Green M., Laguna A., Smart L. E., Spencer J. L., Stone F. G. A. (1975). J. Chem. Soc., Chem. Commun..

[cit14] Hicks J., Vasko P., Goicoechea J. M., Aldridge S. (2019). J. Am. Chem. Soc..

[cit15] Kong R. Y., Crimmin M. R. (2021). Angew. Chem., Int. Ed..

[cit16] Ellis D., McKay D., Macgregor S. A., Rosair G. M., Welch A. J. (2010). Angew. Chem., Int. Ed..

[cit17] Sattler A., Parkin G. (2010). Nature.

[cit18] Hu S., Shima T., Hou Z. (2014). Nature.

[cit19] Hicks J., Vasko P., Heilmann A., Goicoechea J. M., Aldridge S. (2020). Angew. Chem., Int. Ed..

[cit20] Kang X. H., Luo G., Luo L., Hu S. W., Luo Y., Hou Z. M. (2016). J. Am. Chem. Soc..

[cit21] Zhu B., Guan W., Yan L. K., Su Z. M. (2016). J. Am. Chem. Soc..

[cit22] Cabrera-Trujillo J. J., Fernandez I. (2020). Chem.–Eur. J..

[cit23] Miscione G. P., Carvajal M. A., Bottoni A. (2011). Organometallics.

[cit24] Li J., Yoshizawa K. (2012). Chem.–Eur. J..

[cit25] Liu Y., Zhang D., Gao J., Liu C. (2012). Chem.–Eur. J..

[cit26] Wen X., Wu X., Li J. (2018). Org. Lett..

[cit27] Perthuisot C., Edelbach B. L., Zubris D. L., Jones W. D. (1997). Organometallics.

[cit28] Geibel W., Wilke G., Goddard R., Kruger C., Mynott R. (1978). J. Organomet. Chem..

[cit29] Summerscales O. T., Jimenez-Halla J. O. C., Merino G., Power P. P. (2011). J. Am. Chem. Soc..

[cit30] Ohki Y., Suzuki H. (2000). Angew. Chem., Int. Ed. Engl..

[cit31] King R. B., Harmon C. A. (1976). Inorg. Chem..

[cit32] Cash G. G., Pettersen R. C., King R. B. (1977). J. Chem. Soc., Chem. Commun..

[cit33] Cabrera E., Daran J. C., Jeannin Y. (1988). J. Chem. Soc., Chem. Commun..

[cit34] Janowicz A. H., Bergman R. G. (1983). J. Am. Chem. Soc..

[cit35] Jones W. D., Feher F. J. (1984). J. Am. Chem. Soc..

[cit36] Burger P., Bergman R. G. (1993). J. Am. Chem. Soc..

[cit37] Yazaki R., Ohshima T. (2019). Tetrahedron Lett..

[cit38] Shubin V. G., Berezina R. N., Piottukh-Peletski V. N. (1973). J. Organomet. Chem..

[cit39] Zhao Y., Truhlar D. G. (2008). Theor. Chem. Acc..

[cit40] Liu P., Xu X. F., Dong X. F., Keitz B. K., Herbert M. B., Grubbs R. H., Houk K. N. (2012). J. Am. Chem. Soc..

[cit41] Lin M., Kang G. Y., Guo Y. A., Yu Z. X. (2012). J. Am. Chem. Soc..

[cit42] Gusev D. G. (2013). Organometallics.

[cit43] Hopmann K. H. (2016). Organometallics.

[cit44] Hong S. Y., Park Y., Hwang Y., Kim Y. B., Baik M. H., Chang S. (2018). Science.

[cit45] Weigend F. (2006). Phys. Chem. Chem. Phys..

[cit46] Zeits P. D., Fiedler T., Gladysz J. A. (2012). Chem. Commun..

[cit47] Luu Q. H., Fiedler T., Gladysz J. A. (2017). Angew. Chem., Int. Ed..

[cit48] Cleary B. P., Eisenberg R. (1992). Organometallics.

[cit49] Cleary B. P., Eisenberg R. (1995). J. Am. Chem. Soc..

[cit50] Lam W. H., Lam K. C., Lin Z., Shimada S., Perutz R. N., Marder T. B. (2004). Dalton Trans..

[cit51] Zhu Y., Fan L., Chen C. H., Finnell S. R., Foxman B. M., Ozerov O. V. (2007). Organometallics.

[cit52] Pham H. V., Houk K. N. (2014). J. Org. Chem..

[cit53] Müller J., Gaede P. E., Qiao K. (1994). J. Organomet. Chem..

[cit54] Müller J., Gaede P. E., Qiao K. (1993). Angew. Chem., Int. Ed. Engl..

[cit55] Powers I. G., Uyeda C. (2017). ACS Catal..

[cit56] Xiong N., Zhang G., Sun X., Zeng R. (2020). Chin. J. Chem..

[cit57] Edwin J., Geiger W. E., Salzer A., Ruppli U., Rheingold A. L. (1987). J. Am. Chem. Soc..

[cit58] Geiger W. E., Salzer A., Edwin J., Vonphilipsborn W., Piantini U., Rheingold A. L. (1990). J. Am. Chem. Soc..

[cit59] Other means of driving association of two mono-Ir complexes towards the kinetic product, *anti*-(Cp*Ir)_2_(μ-arene) may exist but Fig. 7 suggests that neither steric (**A***vs.***B** and **C***vs.***D**) nor electronic (Ir(i) *vs.* Ir(iii)) factors alone may suffice.

[cit60] Our computations of the relative stabilities of *syn*- and *anti*-Ir(i)Ir(iii) diiridium intermediates of few experimentally untested arenes suggest that in some cases the *anti*-isomers have higher stability even for arenes without benzylic C–H bonds (Table S15†).

