# Supplementary material for: Selective cleavage of unactivated arene ring C–C bonds by iridium: key roles of benzylic C–H activation and metal–metal cooperativity
Source: Chem Sci. 2021 Jan 12;12(10):3568–79. doi: 10.1039/d0sc05900e (PMC8179494; doi:10.1039/d0sc05900e)
Supplement: SC-012-D0SC05900E-s001 [file SC-012-D0SC05900E-s001.pdf]

Electronic Supplementary Material (ESI) for Chemical Science.
This journal is © The Royal Society of Chemistry 2021

Supporting information

Selective cleavage of unactivated arene ring C-C bonds by iridium: key roles of benzylic C-H activation and metal-metal cooperativity

Yancong Tian, Martin Jakoobi, Roman Boulatov and Alexey Sergeev**

Department of Chemistry, University of Liverpool, Crown Street, Liverpool, L69 7ZD, UK

Table of Contents

1. General experimental details S2

2. Experimental procedures and characterisation data S4

3. Preparation of η^4-arene complexes 1, 1-d$_3$, 1-d$_9$, 39-41 .... S6

4. Thermolysis of η^4-arene complexes 1, 1-d$_3$, 1-d$_9$, 39-41 .... S13

5. General procedure for kinetic measurements S19

6.1 Determination of the rate law on complex 1 .... S21

6.2 Determinaiton of the reaction order on mesitylene S23

5.3 Determination of activation parameters $\Delta H^{\neq}$ and $\Delta S^{\neq}$ S25

5.4 Measurement of kinetic isotope effects S28

7. DFT Calculations S32

8. Kinetic modeling of thermolysis of complex 1 (0.1 M) at 50 °C (Fig. 1B) using computed activation parameters (Fig. 2). S60

9. ^{1}H and ^{13}C NMR spectra S65

10. X-ray data for complexes 39 and 40 .... S117

11. References S119

1. General experimental details

Equipment and methods

All air-sensitive manipulations were conducted under an inert atmosphere using an argon-filled Innovative Technology glovebox or standard Schlenk technique under argon. All glassware was heated in an oven at 120 °C and cooled in an argon atmosphere prior to use.

NMR spectra were acquired on a Bruker Avance I (400 MHz) and Bruker Avance III HD (500 MHz) instruments at ambient temperature unless specified otherwise. Chemical shifts (δ) are reported in ppm. ^{1}H NMR spectra are reported relative to the corresponding signals of residual protons in the deuterated solvents (CF_3COOD, TFA-d δ 11.50 ppm; C_6D_6 δ 7.16 ppm; THF-d_8 δ 3.58 ppm; C_6D_{12} 1.38 ppm). ^{13}C NMR spectra were recorder with complete ^{1}H decoupling and are reported relative to the following signals of deuterated solvents: TFA-d: δ 116.60 (q) ppm; C_6D_6: δ 128.06 ppm. The splitting patterns are designated as follows: s (singlet), br. s (broad singlet), d (doublet), dd (doublet of doublets), t (triplet), m (multiplet), br. m (broad multiplet).

Elemental analyses were performed by the Microanalysis Laboratory of the Department of Chemistry, University of Liverpool on a Thermo Flash EA 112 Series instrument.

Mass spectrometry analyses were conducted by the EPSRC UK National Mass Spectrometry Facility at Swansea University. [Cp*Ir(η^6-arene)][BF_4]$_2$ complexes were sent to Swansea University in vials, whereas [Cp*Ir(η^4-arene)] and metallacycles were sent in ampulas sealed under argon or vacuum. Electrospray (ESI) and nano-Electrospray (nanoESI) ionization spectra were recorded on the OrbitrapXL; Electron Ionization (EI) spectra were recorded on the MAT95; Atmospheric Pressure Ionisation spectra (APCI) were recorded on the Xevo G2S using the Atmospheric Solids Analysis Probe (ASAP). All mass spectra (HRMS) were recorded in the positive mode.

Solvents and reagents

n-Hexane was purchased from Sigma-Aldrich, distilled from sodium benzophenone ketyl and stored under argon in glovebox. Anhydrous benzene was purchased from Alfa Aesar and stored under argon in ChemSeal™ bottles. Acetone (Fisher), DCM and Et_2O (both from Aldrich) were used without further purification.

D_2O and DMSO-d_6 were purchased from Sigma-Aldrich; DMSO-d_6 was kept over 4Å molecular seives. C_6D_6 and C_6D_{12} were purchased from Cambridge Isotope Laboratories and were degassed by freeze-pump-thaw cycles and stored over 4Å molecular sieves under an argon atmosphere in Aldrich Sure/Stor™ flasks.

[Cp*Ir(η^4-mesitylene)] **1** was prepared using the corresponding literature procedure.[1] Cobaltocene (Acros) was used as received and stored in the glovebox freezer at -28 °C. $AgBF_4$ and PMe_3 were ordered from Strem and stored in a glovebox. Mesitylene (Fluka) and hexamethyldisiloxane (HMDSO, Sigma-Aldrich) were degassed and kept under argon. Naphthalene (Acros Organics), 2,6-dimethylnaphthalene (Aldrich), trifluoroacetic acid (TFA), trifluoroacetic anhydride (both from FluoroChem), NaH (60% dispersion in mineral oil; Sigma-Aldrich) and argon (Pureshield, BOC > 99.998%) were used as received.

2. Experimental procedures and characterisation data

Mesitylene-d$_3$ (modified literature procedure)[2]

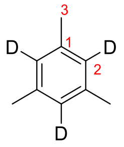

A 100 mL Schlenk bomb was evacuated and filled with argon, mesitylene (1.718 g, 14.29 mmol), trifluoroacetic anhydride (5.0 mL, 36 mmol), D_2O (5.0 mL, 277 mmol) and was left to reflux at 101 °C for 18 h. CAUTION! Mixing trifluoroacetic anhydride and D_2O is highly exothermic! The reaction mixture was cooled to room temperature, extracted with DCM (3x5 mL) and the combined extract was dried over Na_2CO_3. Removal of DCM under reduced pressure gave mesitylene with ca. 90% deuteration of the aromatic C-H protons. After repeating the deuteration procedure with the partially deuterated product and distillation (79 °C, 20 mbar), mesitylene-d$_3$ was obtained as a colourless liquid (397.9 mg, 3.229 mmol) with >99% D content at the three aromatic C-H positions.

^{1}H NMR (400 MHz, C_6D_6): δ 2.29 (s, 9H, *H3*).

^{2}H NMR (400 MHz, C_6H_6): δ 6.88 (s, 3D, *D2*).

HRMS (CI+, methane) m/z: calculated for $[C_9H_{10}D_3]^+$ 124.1200, found 124.1204.

Mesitylene-d$_9$ (modified literature procedure)[3]

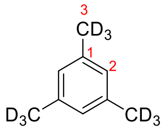

A 10 mL Schlenk bomb was evacuated and filled with argon, then charged with degassed mesitylene (1.40 mL, ca 10.0 mmol), DMSO-d$_6$ (2.00 mL), and NaH (206.0 mg of 60% dispersion in oil, 5.15 mmol) and was left to stir at 100 °C for 24 h. After cooling down the reaction mixture D_2O (4.0 mL) was used to transfer suspension to extraction funnel. The aqueous layer was washed with Et_2O (10 mL) and the combined organic layer was washed with H_2O (5 ml) and dried using K_2CO_3. After removal of Et_2O the deuterium exchange procedure was repeated 2 more times. Vacuum distillation (75 °C, 25 mbar) of the crude product afforded mesitylene-d$_9$ as a colourless liquid (140.1 mg) with >97% deuteration of the methyl groups.

^{1}H NMR (400 MHz, C_6D_6): δ 6.81 (s, 3H, *H2*).

^2H NMR (400 MHz, C_6H_6): δ 2.27 (s, 9D, *D3*).

HRMS (CI+, methane) m/z: calculated for $[C_9H_4D_9]^+$ 130.1577, found 130.1574.

3. Preparation of η^4-arene complexes 1, 1-d$_3$, 1-d$_9$, 39-41

η^4-Arene iridium (I) complexes were prepared by a two step procedure involving the synthesis of intermediate η^6-arene iridium(III) complexes and their subsequent reduction.[1]

General procedure A for the preparation of η^6-arene complexes[1]

A 20 mL vial was charged with $[Cp^*IrCl_2]_2$ (100.0 mg, 0.1255 mmol), $AgBF_4$ (97.7 mg, 0.502 mmol) and a stir bar. Acetone (3 mL) was added and the resulting light-yellow suspension was stirred at room temperature for 15 minutes. Then the precipitate was filtered and washed with acetone (ca. 4 mL) until the washings became colourless. The combined filtrate and washings were evaporated to approximately 1 mL and 0.4 mL of the corresponding arene (ca. 4 eq. per Cp*Ir fragment) was added. The reaction mixture was vigorously stirred at room temperature overnight and evaporated to dryness to give a beige solid. The crude product was dissolved in a minimal amount of trifluoroacetic acid (TFA) and precipitated with an excess of Et_2O (10 mL). This procedure was repeated until the supernatant solution became colourless. Second crop of the product can be obtained from the TFA/Et_2O mother liquor. After drying the product at 50 °C overnight under vacuum a white solid was obtained. If the solid turns light brown upon drying at elevated temperature, the compound should be redissolved in TFA, precipitated with Et_2O and washed again.

General procedure B for the preparation of η^6-arene complexes[1]

A 20 mL vial was charged with $[Cp^*IrCl_2]_2$ (100.0 mg, 0.1255 mmol), $AgBF_4$ (97.7 mg, 0.502 mmol) and a stir bar. Acetone (3 mL) was added and the resulting light-yellow suspension was stirred at room temperature for 15 minutes. Then the precipitate was filtered and the precipitate was washed with acetone (ca. 4 mL) until the washings became colourless.

The combined filtrate and washings were evaporated to approximately 1 mL and transferred to argon filled Schlenk tube/flask. After evaporating the solution to residue, corresponding naphthalene derivative (ca 8 equiv.) and 0.4 mL of $BF_3 \cdot Et_2O$ were added. Reaction mixture was

vigorously stirred at room temperature for 3 h and then evaporated to dryness. The crude mixture was dissolved in TFA and precipitated with Et_2O in argon filled Sclenk tube. The Et_2O layer was pipetted off, the white solid washed again with Et_2O (5mL) and then dried under vacuum to give white solid. This process was repeated two or three times, until washings remained colourless. The TFA/Et_2O mixtures can be used to collect second crop of product. After drying the product at 50 °C overnight under vacuum a white solid was obtained. If, however the solid turns brown during drying at elevated temperature, the compound should be redissolved in TFA, precipitated with Et_2O and washed again.

η^6-Complexes are rather poorly soluble in most organic solvents, and the most suitable sovent for recording NMR of these complexes is TFA. During NMR measurements in TFA-d the η^6-arene complexes may start to precipitate out.

[Cp*Ir(η^6-mesitylene-d_9)][BF_4]$_2$

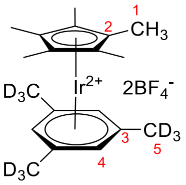

The complex was prepared according to General procedure A using [Cp*$IrCl_2$]$_2$ (125.2 mg, 0.1571 mmol), $AgBF_4$ (122.2 mg, 0.6277 mmol) and mesitylene-d_9 (122.1 mg, 0.9447 mmol). [Cp*Ir(η^6-mesitylene-d_9)][BF_4]$_2$ was isolated as white solid (126.6 mg, 0.2008 mmol) in 64% yield.

^{1}H NMR (400 MHz, TFA-d): δ 7.13 (s, 3H, *H4*), 2.32 (s, 15H, *H1*).

^{2}H NMR (400 MHz, TFA): δ 2.64 (s, 9D, *D5*).

Elemental analysis (%) calculated for $C_{19}H_{18}D_9IrB_2F_8$: C 36.21, H 4.32; found C 35.45, H 4.18.

HRMS (nanoESI+) m/z: calculated for [$^{191}IrC_{19}H_{18}D_9$]$^{2+}$ 227.6136, found 227.6135.

[Cp*Ir(η⁶-mesitylene-d₃)][BF₄]₂

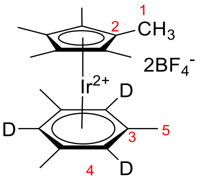

The complex was prepared according to General procedure A using $[Cp^*IrCl_2]_2$ (100.6 mg, 0.1263 mmol), $AgBF_4$ (99.2 mg, 0.5096 mmol) and mesitylene-d_3 (115.1 mg, 0.9342 mmol). $[Cp^*Ir(\eta^6\text{-mesitylene-}d_3)][BF_4]_2$ was isolated as white solid (152.9 mg, 0.2449 mmol) in 97% yield.

^{1}H NMR (400 MHz, TFA-d): δ 2.65 (s, 9H, *H5*), 2.32 (s, 15H, *H1*).

^{2}H NMR (400 MHz, TFA): δ 7.17 (br. s, 3D, *D4*).

Elemental analysis (%) calculated for $C_{19}H_{24}D_3IrB_2F_8$: C 36.55, H 4.36; found C 35.81, H 4.11.

HRMS (nanoESI+) m/z: calculated for $[^{191}IrC_{19}H_{24}D_3]^+$ 448.1823, found 448.1826.

$[Cp^*Ir(\eta^6\text{- }C_6Me_6)][BF_4]_2$ (literature procedure)[2]

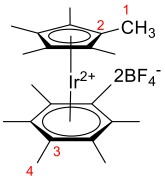

The complex was prepared according to published procedure using $[Cp^*IrCl_2]_2$ (50.1 mg, 0.0629 mmol), TFA (0.75 mL) and C_6Me_6 (50.2 mg, 0.309 mmol), which gave white solid (71.2 mg, 0.107 mmol) in 86% yield.

^{1}H NMR (400 MHz, TFA-d): δ 2.46 (s, 18H, *H4*), 2.03 (s, 15H, *H1*). ^{1}H NMR is similar to the one published previously.[4]

^{13}C NMR (100 MHz, TFA-d): δ 113.57 (*C2/C3*), 104.35 (*C3/C2*), 16.67 (*C4*), 8.83 (*C1*).

Elemental analysis (%) calculated for $C_{22}H_{33}IrB_2F_8$: C 39.84, H 5.01; found C 38.97, H 4.34.

HRMS (nanoESI+) m/z: calculated for $[^{191}IrClC_{22}H_{33}]^{2+}$ 244.1089, found 244.1090.

[Cp*Ir(η⁶-naphthalene)][BF₄]₂

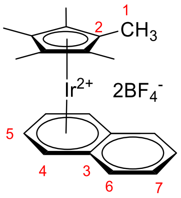

The complex was prepared according to General procedure B using $[Cp^*IrCl_2]_2$ (90.1 mg, 0.113 mmol), $AgBF_4$ (88.1 mg, 0.453 mmol), naphthalene (101.0 mg, 0.7880 mmol), $BF_3{\cdot}2H_2O$ (0.5 mL) and was left to stir overnight. $[Cp^*Ir(\eta^6\text{-naphthalene})][BF_4]_2$ was isolated as white solid (140.1 mg, 0.2227 mmol) in 99% yield.

^{1}H NMR (400 MHz, TFA-d): δ 8.51 (dd, J 6.7, 3.1 Hz, 2H, *H4*), 8.22 (dd, J 4.3, 2.7 Hz, 2H, *H6*), 8.17 (J 6.6, 3.1 Hz, 2H, *H5*), 7.67 (dd, J 4.5, 2.6 Hz, 2H, *H7*), 2.22 (s, 15H, *H1*). ^{1}H NMR spectrum is similar to PF_6 salt published previously.[5]

^{13}C NMR (100 MHz, TFA-d): δ 140.82 (*C3*, *C6* or *C7*), 128.32 (*C3*, *C6* or *C7*), 107.15 (*C3*, *C6* or *C7*), 106.45 (*C2*), 99.20 (*C4* or *C5*), 97.96 (*C5* or *C4*), 9.78 (*C1*).

Elemental analysis (%) calculated for $C_{20}H_{23}IrB_2F_8$: C 38.18, H 3.18; found C 37.87, H 3.33.

HRMS (nanoESI+) m/z: calculated for $[^{191}IrClC_{20}H_{23}]^{2+}$ 227.0697, found 227.0699.

[Cp*Ir(η^6-2,6-dimethylnaphthalene)][BF$_4$]$_2$

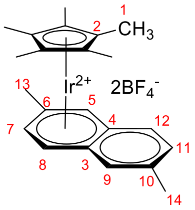

The complex was prepared according to the General procedure B using $[Cp^*IrCl_2]_2$ (100.2 mg, 0.1257 mmol), $AgBF_4$ (100.1 mg, 0.5142 mmol), 2-methylnaphthalene (78.2 mg, 0.500 mmol), $BF_3{\cdot}2H_2O$ (0.3 mL) and was left to stir overnight. [Cp*Ir(η^6-2,6-dimethylnaphthalene)][BF$_4$]$_2$ was isolated as vanilla white solid (111.8 mg, 0.17009 mmol) in 68% yield.

^{1}H NMR (400 MHz, TFA-d): δ 8.16 (dd, J 8.9, 0.9 Hz, 1H, *H11*), 7.88 (d, J 6.9 Hz, 1H, *H8*), 7.87 (s, 1H, *H5*), 7.83 (d, J 8.9 Hz, 1H, *H12*), 7.71 (s, 1H, *H9*), 7.30 (d, J 6.8 Hz, 1H, *H7*), 2.80 (s, 3H, *H14*), 2.74 (s, 3H, *H13*), 1.98 (s, 15H, *H1*).

^{13}C NMR (100 MHz, TFA-d): δ 155.05, 143.47, 128.24 (C*12*), 124.46 (*C9*), 115.28, 108.15, 105.55 (*C2*), 104.58, 99.41 (*C7*), 97.25, 97.02 (*C5*), 23.13 (*C14*), 19.16 (C13), 9.49 (C1).

Elemental analysis (%) calculated for $C_{22}H_{27}IrB_2F_8$: C 40.20, H 4.14; found C 39.86, H 4.02.

HRMS (EI+) m/z: calculated for $[^{191}IrC_{22}H_{26}]^{+}$ 481.1635, found 481.1631.

General procedure for reduction of η^6-arene complexes to η^4-arene complexes

In a glovebox, a 20 ml screw cap vial was charged with [Cp*Ir(η^4-arene)][BF_4]$_2$ (0.100 mmol), Cp_2Co (0.200 mmol), benzene (2.0 mL) and a magnetic stir bar. The vial was sealed and the reaction mixture was vigorously stirred for 2 h. *n*-Hexane (10-15 mL) was added to the resulting red-orange solution to precipitate out olive green [Cp_2Co][BF_4]. The suspension was filtered and the precipitate was washed with *n*-hexane (2x4 mL) until washings remained colorless. The combined filtrate and washings were evaporated in vacuum to give the product.

[Cp*Ir(η^4-mesitylene-d$_9$)] (1-d$_9$)

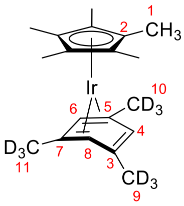

The complex was prepared according to the general procedure using [Cp*Ir(η^6-mesitylene-d$_9$)][BF_4]$_2$ (106.0 mg, 0.1682 mmol), Cp_2Co (63.4 mg, 0.335 mmol) and benzene (3.4 mL). [Cp*Ir(η^4-mesitylene-d$_9$)] (**1-d$_9$**) was isolated as orange-yellow solid (66.8 mg, 0.146 mmol) in 87% yield.

^{1}H NMR (400 MHz, C_6D_6): δ 5.39 (s, 1H, *H6*), 4.96 (d, J 1.9 Hz, 1H, *H4*), 2.67 (d, J 1.9 Hz, 1H, *H8*), 1.77 (s, 15H, *H1*). ^{1}H NMR (400 MHz, C_6D_{12}): δ 5.28 (s, 1H, *H6*), 4.52 (s, 1H, *H4*), 2.45 (d, J 1.2 Hz, 1H, *H8*), 1.83 (s, 15H, *H1*).

^{2}H NMR (400 MHz, C_6H_6): δ 2.01 (s, 3H, *D11*), 1.54 (s, 3H, *D9*), 1.25 (s, 3H, *D10*).

HRMS (ASAP) m/z: calculated for [$^{191}IrC_{19}H_{19}D_9$]$^+$ 456.2362, found 456.2362.

[Cp*Ir(η^4-mesitylene-d$_3$)] (1-d$_3$)

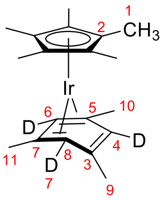

The complex was prepared according to the general procedure using [Cp*Ir(η^6-mesitylene-d$_3$)][BF_4]$_2$ (132.3 mg, 0.2119 mmol), Cp_2Co (80.0 mg, 0.423 mmol) and benzene (4.2 mL). [Cp*Ir(η^4-mesitylene-d$_3$)] (**1-d$_3$**) was isolated as yellow solid (74.8 mg, 0.166 mmol) in 78% yield.

^{1}H NMR (400 MHz, C_6D_6): δ 2.09 (s, 3H, *H11*), 1.77 (s, 15H, *H1*), 1.62 (s, 3H, *H9*), 1.33 (s, 3H, *H10*). ^{1}H NMR (400 MHz, C_6D_{12}): δ 2.07 (s, 3H, *H11*, 1.83 (s, 15H, *H1*), 1.34 (s, 3H, *H9*), 1.12 (s, 3H, *H10*).

^{2}H NMR (400 MHz, C_6H_6): δ 5.38 (s, 1D, *D6*), 4.96 (s, 1D, *D4*), 2.64 (s, 1D, *D8*).

HRMS (ASAP) m/z: calculated for $[^{191}IrC_{19}H_{25}D_3]^+$ 450.1985, found 450.1966; calculated for $[^{193}IrC_{19}H_{25}D_3]^+$ 452.2009, found 452.2016.

[Cp*Ir(η^4-C_6Me_6)] (39)

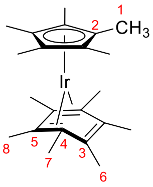

The complex was prepared according to the general procedure using [Cp*Ir(η^6-C_6Me_6)][BF_4]$_2$ (28.4 mg, 0.0428 mmol), Cp_2Co (15.4 mg, 0.0814 mmol) and DCM (1.0 mL). [Cp*Ir(η^4- C_6Me_6)] was isolated as a pale-yellow solid (20.8 mg, 0.0425 mmol) in 99% yield. Single crystals were grown from C_6H_6 using slow evaporation. Single crystals were grown from a benzene solution via slow evaporation.

^{1}H NMR (400 MHz, C_6D_6): δ 1.96 (s, 6H, *H8*), 1.66 (s, 15H, *H1*), 1.54 (s, 6H, *H6*), 1.42 (s, 6H, *H7*).

^{13}C NMR (100 MHz, C_6D_6): δ 132.96 (*C3*), 88.54 (*C2*), 79.61 (*C5*), 52.92 (*C4*), 20.09 (*C7*), 14.24 (*C6*), 14.05 (*C8*), 9.63 (*C1*).

HRMS (ASAP) m/z: calculated for $[^{191}IrC_{22}H_{33}]^+$ 488.2188, found 488.2186.

[Cp*Ir(η^4-naphthalene)] (40)

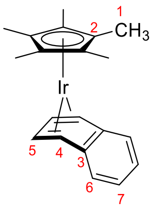

The complex was prepared according to the general procedure using [Cp*Ir(η^6-naphthalene)][BF_4]$_2$ (67.3 mg, 0.107 mmol), Cp_2Co (40.4 mg, 0.214 mmol) and benzene (2.0 mL). [Cp*Ir(η^4-naphthalene)] was isolated as greenish-yellow crystals (39.2 mg, 0.0860 mmol) in 80% yield.

^{1}H NMR (400 MHz, C_6D_6): δ 6.83 (m, 2H, *H6*), 6.73 (m, 2H, *H7*), 5.59 (d, J 4.7 Hz, 2H, *H5*), 3.48 (d, J 4.3 Hz, 2H, *H4*), 1.76 (s, 1H, *H1*).

^{1}H NMR (400 MHz, CD_2Cl_2): δ 6.57-6.49 (m, 4H, *H6+H7*), 5.66 (m, 2H, *H5*), 3.40 (m, 2H, *H4*), 1.98 (s, 15H, *H1*).

^{13}C NMR (100 MHz, C_6D_6): δ 146.71 (*C3*), 123.62 (*C7*), 121.21 (*C6*), 89.43 (*C2*), 66.44 (*C5*), 47.83 (*C4*), 10.51 (*C1*).

^{13}C NMR (100 MHz, CD_2Cl_2): δ 147.18 (*C3*), 123.16 (*C7*), 121.06 (*C6*), 90.05 (*C2*), 66.62 (*C5*), 47.51 (*C4*), 10.74 (*C1*).

HRMS (ASAP) m/z: calculated for $[^{191}IrC_{20}H_{24}]^+$ 455.1484, found 455.1473.

[Cp*Ir(η^4-2,6-dimethylnaphthalene)] (41)

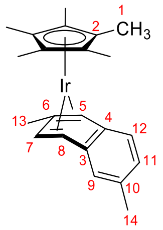

The complex was prepared according to the general procedure using [Cp*Ir(η^6-2,6-dimethylnaphthalene)][BF_4]$_2$ (100.5 mg, 0.153 mmol), Cp_2Co (57.8 mg, 0.306 mmol) and benzene (3.0 mL). [Cp*Ir(η^4-2,6-dimethylnaphthalene)] was isolated as dark-orange-brown solid (68.2 mg, 0.141 mmol) in 92% yield.

^{1}H NMR (400 MHz, C_6D_6): δ 6.79 (d, J 7. Hz, 1H, *H12*), 6.66 (s, 1H, *H9*), 6.52 (d, J 7.1 Hz, 1H, *H11*), 5.49 (d, J 4.7 Hz, 1H, *H7*), 3.44 (d, J 4.8 Hz, 1H, *H8*), 3.30 (s, 1H, *H5*), 2.06 (s, 3H, *H14*), 2.03 (s, 3H, *H13*), 1.74 (s, 15H, *H1*).

^{13}C NMR (100 MHz, C_6D_6): δ 147.20 (*C3*), 144.92 (*C4*), 131.96 (*C10*), 124.02 (*C11*), 122.36 (*C9*), 120.60 (*C12*), 89.00 (*C2*), 78.89 (*C6*), 68.82 (*C7*), 50.37 (*C5*), 47.72 (*C8*), 21.24 (*C14*), 19.64 (*C13*), 10.26 (*C1*).

HRMS (ASAP) m/z: calculated for $[^{191}IrC_{22}H_{26}]^+$ 481.1640, found 481.1624; calculated for $[^{193}IrC_{22}H_{28}]^+$ 485.1820, found 485.1820.

4. Thermolysis of η^4-arene complexes 1, 1-d$_3$, 1-d$_9$, 39-41

General procedure for arene ring C-C bond cleavage in [Cp*Ir(η^4-mesitylene-d$_n$)] complexes

In a glovebox, a 4 mL vial was charged with a solution of [Cp*Ir(η^4-mesitylene-d$_n$] (0.100 mmol) in *n*-hexane (0.80 mL). The vial was sealed with a Teflon lined screw cap and taken outside of the glovebox. The reaction mixture was stirred in an oil bath at 50 °C. The resulting mixture was evaporated in vacuum at 50 °C to give the corresponding product as a solid.

Thermolysis of complex 1-d$_3$ to give metallacycle 2-d$_3$

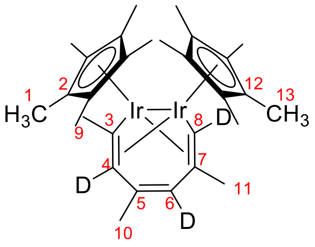

The reaction was conducted according to the general procedure using [Cp*Ir(η^4–mesitylene-d$_3$] **1-d$_3$** (13.1 mg, 0.0291 mmol) and C_6D_{12} (520.2 mg, 0.583 ml to make a 0.050 M solution). The mixture was stirred at 50 °C (oil bath temperature) for 24 h and cooled to room temperature. Evaporation of the resulting solution in vacuum gave **2-d$_3$** as a sand-yellow solid (10.9 mg, 0.01401 mmol) in 96% yield.

^{1}H NMR (500 MHz, C_6D_6): δ 2.69 (s, 3H, *H9*), 2.11 (s, 3H, *H11*), 2.01 (s, 3H, *H10*), 1.77 (s, 15H, *H1* or *H13*), 1.73 (s, 15H, *H13* or *H1*). ^{1}H NMR (400 MHz, C_6D_{12}): δ 2.43 (s, 3H, *H9*), 1.82 (s, 15H, *H1* or *H13*), 1.80 (s, 15H, *H13* or *H1*), 1.78 (s, 3H, *H10* or *H11*), 1.66 (s, 3H, *H11* or *H10*).

^{2}H NMR (77 MHz, C_6H_6): δ 8.05 (s, 1D, *D8*), 5.16 (s, 1D, *D4*), 4.06 (s, 1D, *D6*).

HRMS (ASAP) m/z: calculated for $[^{191}Ir_2C_{29}D_3H_{39}]^+$ 775.2687, found 775.2686.

Thermolysis of complex 1-d9 to give metallacycle 2-d9

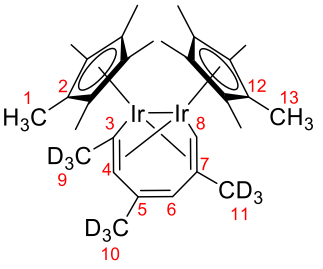

The reaction was conducted according to the general procedure using [Cp*Ir(η^4–mesitylene-d9] (**1-d9**) (13.1 mg, 0.0287 mmol) and C_6D_{12} (519.9 mg, 0.464 ml to make a 0.050 M solution). The mixture was stirred at 50 °C (oil bath temperature) for 24 h and cooled to room temperature. Evaporation of the resulting solution in vacuum gave **2-d9** as a brownish-yellow solid (10.3 mg, 0.0131 mmol) in 91% yield.

^{1}H NMR (500 MHz, C_6D_6): δ 8.04 (s, 1H, *H8*), 5.17 (s, 1H, *H6*), 4.11 (d, J 1.4 Hz, 1H, *H4*), 1.77 (s, 15H, *H1* or *H13*), 1.73 (s, 15H, *H13* or *H1*). ^{1}H NMR (400 MHz, C_6D_{12}): δ 7.69 (s, 1H, *H8*), 4.63 (s, 1H, *H6*), 3.76 (d, J 1.0 Hz, 1H, *H4*), 1.82 (s, 15H, *H2* or *H12*), 1.81 (s, 15H, *H12* or *H2*).

^{2}H NMR (77 MHz, C_6H_6): δ 2.12-1.95 (br. m, *D9+D10+D11*).

Due to lower deuterium content in the mesitylene-d9 (compared to mesitylene-d3) the HRMS spectrum resolution was not sufficient for accurate mass measurement.

Thermolysis of 1 in C_6D_6 at 50 °C

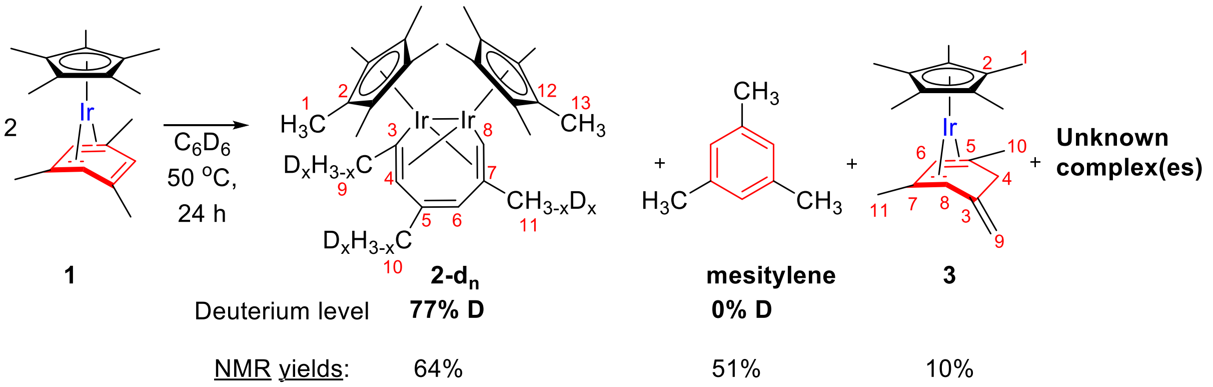

In a glovebox, a J. Young NMR tube was charged with a solution of **1** (15.2 mg, 0.0339 mmol) in C_6D_6 (0.678 mL to make up a 0.050 M solution). The NMR tube was sealed, taken from the glovebox and heated in an oil bath at 50 °C for 24 h. The NMR tube was then removed from

the bath, cooled to room temperature and a proton NMR spectrum was recored. The spectrum indicated the presence of **2-dn**, mesitylene, **3** as well as a number of unidentified species.

Complex 2-dn:

^{1}H NMR (400 MHz, C_6D_6): δ 8.04 (s, 1H, *H8*), 5.17 (s, 1H, *H6*), 4.11 (d, J 1.3 Hz, 1H, *H4*), 2.69 (s, <1H, H9), 2.11 (s, <1H, *H11*), 2.00 (d, J 1.2 Hz, <1H, *H10*), 1.77 (s, 15H, *H1* or *H13*), 1.73 (s, 15H, *H13* or *H1*). See page S99-S103 for more detailed illustration of partial deuteration of Me-groups in **2-dn**.

Minor product **3**:

^{1}H NMR (400 MHz, C_6D_6): δ 4.95 (d, J 1.7 Hz, 1H, *H9*), 4.49 (d, J 1.6 Hz, 1H, *H9*), 2.90 (d, J 16.3 Hz, 1H, *H9*), 2.52 (d, J 16.5 Hz, 1H, *H9*). The remaining peaks *H6*, *H8*, *H10* and *H11* could not be unambigiously identified in this reaction mixture, as they had merged with other peaks in their corresponding region.

Note that when an alkane solvent (*n*-hexane, cyclohexane-d_{12}) for thermolysis of **1** was replaced for THF, DCM, benzene, benzene-d_6, toluene or mesitylene some side-products were formed, one of which is **3**. Complex **3** was originally identified upon thermolysis of **1** in d_8-THF rather than in d_6-benzene, because in the former solvent fewer overlaps with other signals were observed in a ^{1}H NMR spectrum. The tentative structural assignment of **3** was based on the similarity of ^{1}H and ^{13}C NMR spectra of **3** and known $Fe(CO)_3$(mesitylene tautomer) complex.[6]

^{1}H NMR (400 MHz, THF-d_8): δ 4.45 (d, J 1.6 Hz, 1H, *H9*), 4.31 (s, 1H, *H6*), 3.97 (br. s, 1H, *H9*), 3.07 (s, 1H, *H8*), 2.51 (d, J 16.2 Hz, 1H, *H4*), 2.21 (d, J 16.1 Hz, 1H, *H4*), 1.89 (s, 15H, *H1*), 1.88 (s, 3H, *H11*), 1.26 (s, 3H, *H10*).

^{13}C NMR (100 MHz, THF-d_8): δ 155.43 (*C9*), 93.68 (*C3*), 89.17 (*C2*), 79.81 (*C7*), 70.8 (*C6*), 55.50 (*C8*), 48.28 (*C5*), 39.38 (*C4*), 25.39 (*C10*), 19.76 (*C11*), 10.07 (*C1*).

Heating complex 2 in C_6D_6 at 50 °C

In a glovebox, a J. Young NMR tube was charged with a solution of **2** (10.3 mg, 0.0132 mmol) in C_6D_6 (500 μL). The NMR tube was sealed, taken outside of the glovebox and heated in an oil bath at 50 °C for 24 h, cooled to toom temperature. ^{1}H NMR spectrum of the reaction mixture showed no detectable deuterium incorporation in **2** as the ratio of Me-group integrals and aromatic C-H integrals was 3:1 (compare with the complex **2-d$_n$** where the ratio was <1:1).

Thermolysis of 1 in the presence of 4 eq. of PMe_3 to give benzyl complex 4.

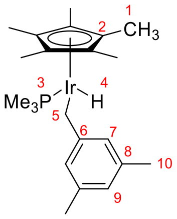

The thermolysis was conducted according to the general procedure using [Cp*Ir(η^4-mesitylene)] (**1**) (31.5 mg, 0.0704 mmol) and PMe_3 (21.4 mg, 0.281 mmol) in *n*-hexane (1.4 mL). The reaction mixture was stirred at 50 °C (heating block temperature) for 76 h. The resulting complex **4**, [Cp*Ir(PMe_3)(H)(3,5-dimethylbenzyl)], was isolated as a beige solid (36.8 mg, 0.0703 mmol) in 88% yield. Alternatively, **4** can be obtained in 88% isolated yield after heating **1** at a higher temperature of 100 °C for 1 h.

^{1}H NMR (400 MHz, C_6D_6): δ 7.20 (s, 2H, *H7*), 6.67 (s, 1H, *H9*), 3.12 (dd, J 10.7, 8.5 Hz, 1H, *H5*), 3.03 (dd, J 10.9, 3.2 Hz, 1H, *H5*), 2.32 (s, 6H, *H10*), 1.77 (d, J_{H-P} 1.4 Hz, 15H, *H1*), 1.18 (d, J_{H-P} 9.7, 9H, *H3*), -17.31 (d, J_{H-P} 36.7 Hz, 1H, *H4*).

^{13}C NMR (100 MHz, C_6D_6): δ 155.33 (d, J_{C-P} 3.3 Hz, *C6*), 135.95 (*C8*), 127.99 (*C7*), 124.26 (*C9*), 91.84 (d, J_{C-P} 3.5 Hz, *C2*), 21.75 (*C10*), 19.18 (d, J_{C-P} 36.3 Hz, *C3*), 10.16 (*C1*), -7.46 (d, J_{C-P} 6.6 Hz, *C5*).

^{31}P NMR (162 MHz, C_6D_6): δ -45.25.

HRMS (ASAP) m/z: calculated for $[^{191}IrC_{22}H_{35}P]^+$ 521.2082, found 521.2079.

Thermolysis of 1 in presence of mesitylene-d₃

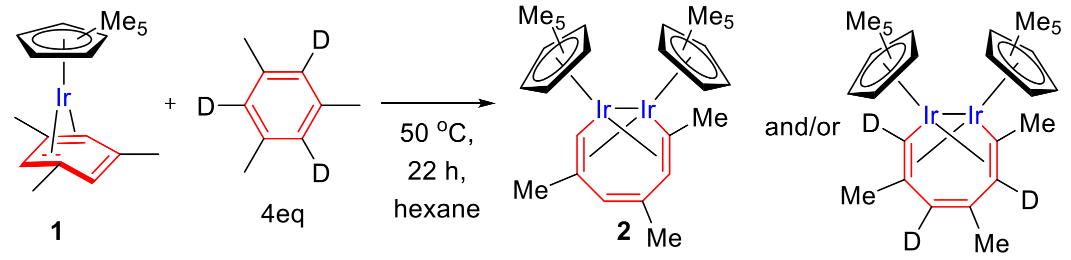

Full conversion   95% isolated yield   NOT OBSERVED

A solution of complex **1** (15.2 mg, 0.0339 mmol), mesitylene-d_3 (9.8 mg, 0.079 mmol) in *n*-hexane (678 µL to make 0.050 M solution) was stirred at 50 °C for 22 h. The solution was cooled to room temperature and evaporated in vacuum to give a brownish-yellow solid (12.5 mg, 0.0161 mmol) in 95% yield. No incorporation of deuterium in **2** was observed by ^{1}H and ^{2}H NMR spectra.

Attempted cleavage of [Cp*Ir(η^4–C_6Me_6)] (39)

[Cp*Ir(η^4–C_6Me_6)] (13.2 mg, 0.0270 mmol) in *n*-hexane (0.3 mL) was heated at 150 °C (oil bath temperature) for 24 h. The reaction mixture was cooled to room temperature and evaporation in vacuum to give beige powder (12.9 mg).

^{1}H NMR spectrum of the residue indicated complete conversion of the starting complex and formation of C_6Me_6 along with several unidentified compounds that showed multiple signals at δ 1.40-2.30 ppm. According to HRMS data, the structure of the main product agrees with the structure [(Cp*Ir)$_2$(C_6Me_6)].

HRMS (ASAP) m/z: calculated for [$^{191}Ir_2C_{32}H_{45}$]$^+$ 811.2733, found for [(Cp*Ir)$_2$(C_6Me_6)-3H] 811.2725.

Attempted cleavage of [Cp*Ir(η^4–naphthalene)] (40)

[Cp*Ir(η^4–naphthalene)] (27.6 mg, 0.0606 mmol) in *n*-hexane (0.50 mL) was heated at 150 °C (oil bath temperature) for 36 h. The reaction mixture was cooled to room temperature and evaporation in vacuum to give a brown amorphous solid (26.2 mg).

^{1}H NMR spectrum indicated incomplete consumption of the starting complex and formation of naphthalene. Additionally, new broad signals were formed at δ 1.30-2.30 ppm. No signals characteristic for the previously reported products of metal insertion into the aromatic ring were observed at δ 4.00-8.10 ppm. According to HRMS data, the structure of main products agrees with [$(Cp^*Ir)_2$(naphthalene)] and [$(Cp^*Ir)_3$(naphthalene)].

HRMS (ASAP) m/z: calculated for $[^{193}Ir_2{}^{191}IrC_{40}H_{54}]^+$ 1111.3090, found for [$(Cp^*Ir)_3$(naphthalene)+H] 1111.3091; calculated for $[^{191}Ir_2C_{30}H_{39}]^+$ 781.2264, found for [$(Cp^*Ir)_2$(naphthalene)+H] 781.2250.

Attempted cleavage of [Cp*Ir(η^4–2,6-dimethylnaphthalene)] (41)

[Cp*Ir(η^4–2,6-dimethylnaphthalene)] (12.4 mg, 0.0256 mmol) in *n*-hexane (0.3 mL) was heated at 150 °C (oil bath temperature) fur 24 h of heating in an oil bath. Evaporation of the resulting solution in vacuum gave a brown residue (10.2 mg).

^{1}H NMR spectrum of the residue indicated complete consumption of the starting complex and formation of 2,6-dimethylnaphthalene. Additionally, new broad signals appeared at δ 1.30-2.20 ppm. No signals characteristic for the previously reported products of metal insertion into the aromatic ring were observed at δ 4.00-8.10 ppm.

HRMS (ASAP and EI+) failed to give fragments corresponding to the expected product

5. General procedure for kinetic measurements

Preparation of stock solution of C_6D_{12} with hexamethyldisiloxane (HMDSO) as standard

In a glovebox, HMDSO (26 µL, 0.12 mmol) was added to C_6D_{12} (5000 µL) to prepare a stock solution for thermolysis reactions of **1** and its deuterated analogues. In between various experiments the stock solution was kept in a glovebox freezer.

Measurement of ^{1}H NMR spectra for all kinetic experiments

1) At the designated times, the NMR tube was taken from the oil bath and frozen in liquid N_2, washed with DCM to remove oil and then frozen in liquid N_2 again.
2) The sample was then warmed to room temperature, wiped clean and measured at room temperature (16 scans, d1=5 s) three times for each time point.
3) After the measurement, the sample was frozen again and before continuing thermolysis at specific temperature the sample was warmed to room temperature.

Analysis of ^{1}H NMR data

1) After the measurements were done for a specific experiment the data analysis was done using Bruker TopSpin program.
2) All the spectra were referenced to 0.09 ppm (the chemical shift of HMDSO in C_6D_{12}) and baseline correction was applied.
3) For all the aromatic C-H protons of the starting material and the product specific integral regions were defined and these were applied on all the spectra using **multi_integ3** command to obtain text file containing all the integration data.
4) The integrals were then exported to Excel to calculate the concentration of starting material at any given time.

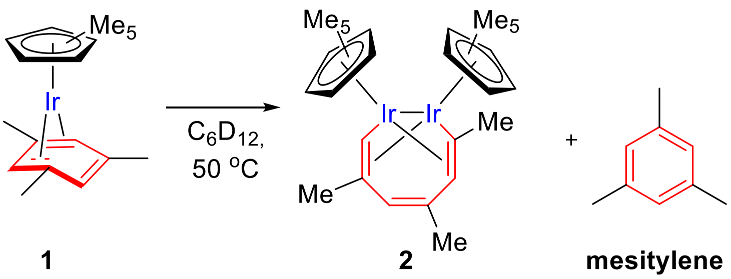

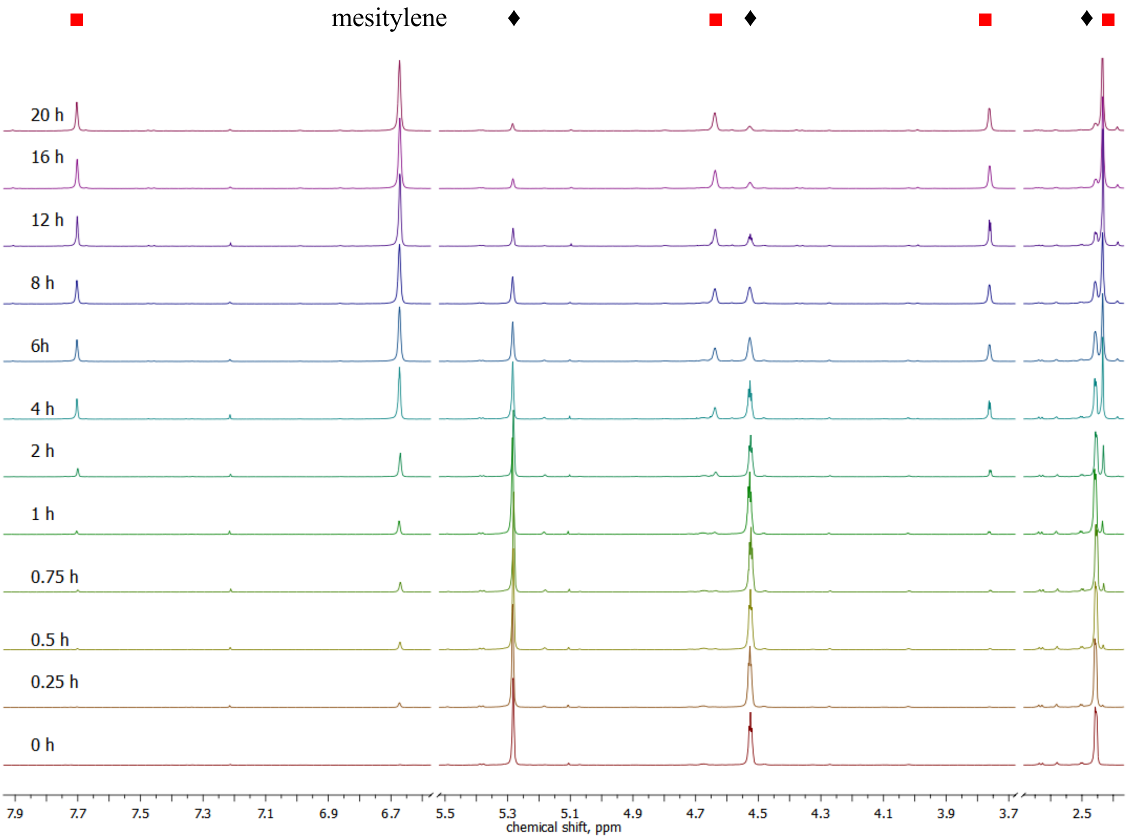

Figure S1. ^{1}H NMR (400 MHz, 25 °C, C_6D_{12}) spectra of a typical kinetic experiment on thermolysis of **1** at 50 °C. The C-H peaks corresponding to **1** and **2** are labelled as ♦ and ■ respectively.

6.1 Determination of the rate law on complex 1

In a glovebox, an NMR tube was charged with η^4–arene complex **1** (2.6-22.4 mg, 0.0058-0.0500 mmol) and then a stock solution (500-567 μL) of C_6D_{12} containing internal standard (HMDSO) was added to prepare a 0.010-0.050 M solution of **1**. The tube was attached to an NMR tube evacuation adapter, taken out of the glovebox, frozen in liquid N_2, flame sealed under vacuum, warmed to room temperature and then fully immersed into an oil bath at 50 °C. After the designated time interval, the NMR tube was taken out of the bath and ^{1}H NMR spectra were recorded as described in the general procedure.

Table S1. Concentration-time data for thermolysis of **1** at 50 ℃ at starting concentrations of **1** in the range of 0.01- 0.1 M.[a]

Time, s	c(**1**), M				
0	0.010232	0.02528	0.05059	0.07449	0.09904
900	0.009876	0.02412	0.04882	0.07102	0.09444
1800	0.009355	0.02306	0.04687	0.06869	0.09106
2700	0.008974	0.02225	0.04439	0.06512	0.08643
3600	0.008553	0.02125	0.04226	0.06161	0.08173

a) The measurement were performed during the 1st hour of the reaction when conversion was less than 18%.

Table S2. Initial rates for thermolysis of **1** at various starting concentrations of **1** (0.01- 0.1 M) at 50 ℃.[a]

c(**1**), M	Initial rate k_{int}[b], s^{-1}	Error margin for k[c], s^{-1}	ln c	ln k_{int}	Error margin for ln k_{int}[d]
0.010232	$4.73418 \cdot 10^{-7}$	$1.42098 \cdot 10^{-8}$	-4.582182	-14.563	0.03001
0.025286	$1.10422 \cdot 10^{-6}$	$3.93542 \cdot 10^{-8}$	-3.677494	-13.716	0.03564
0.050598	$2.33861 \cdot 10^{-6}$	$7.89104 \cdot 10^{-8}$	-2.983838	-12.965	0.03374
0.074498	$3.51869 \cdot 10^{-6}$	$1.33381 \cdot 10^{-7}$	-2.596977	-12.557	0.03791
0.099040	$4.73428 \cdot 10^{-6}$	$1.44887 \cdot 10^{-7}$	-2.312230	-12.260	0.03060

a) The thermolysis was conducted in the absence of added mesitylene. b) The initial rates were obtained from the linear plot of c(1) vs. time (0-1 h). c) Errors margins correspond to the standard uncertainty and were obtained the from linear least-squares analyses of the data using LINEST function in Excel. d) Errors margins correspond to the standard deviation of ln k.

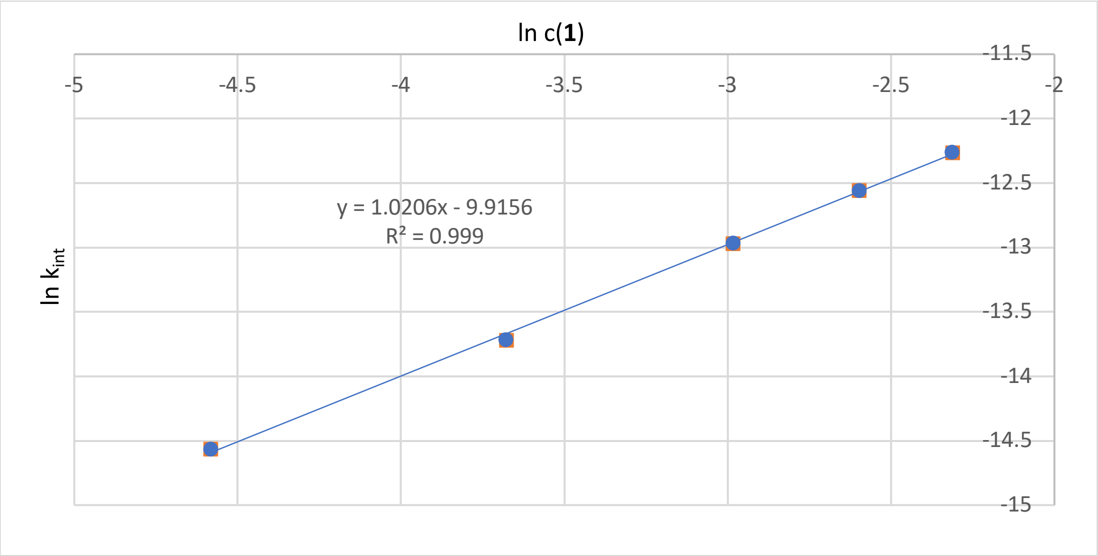

Figure S2. Determination of the order on η [4]-mesitylene complex **1** by caluculating the slope of the line ln k_{int} vs ln c(**1**). The slope of 1 suggests the reaction is 1st order on **1**.

6.2 Determinaiton of the reaction order on mesitylene

In a glovebox, an NMR tube was charged with η^4–arene complex **1** (11.1 mg, 0.0248 mmol), mesitylene (7-34 μL) and the appropriate amount of stock solution of C_6D_{12} containing internal standard (HMDSO) to obtain 0.050 M solution of **1**. The tube was attached to an NMR tube evacuation adapter, taken out of the glovebox, frozen in liquid N_2, flame sealed under vacuum, warmed to room temperature and then fully immersed into an oil bath at 50 °C. After the designated time interval, the NMR tube was taken out of the bath and ^{1}H NMR spectra were recorded as described in the general procedure.

Table S3. Concentration-time data for thermolysis of **1** (0.050 M) at various starting concentrations of mesitylene c(Mes) (0.01- 0.1 M) at 50 ℃.[a]

Time, s	c(**1**), M				
	c(Mes) =0.10 M	c(Mes) =0.21 M	c(Mes) =0.31 M	c(Mes) =0.40 M	c(Mes) =0.50 M
0	0.050023	0.050390	0.050283	0.049812	0.049311
900	0.047863	0.047511	0.048140	0.047692	0.047228
1800	0.046194	0.046172	0.046402	0.045791	0.045581
2700	0.043948	0.044471	0.044671	0.043712	0.043414
3600	0.041338	0.042403	0.042536	0.040596	0.040624

a) The initial kinetics were measured during the 1st hour of the reaction when conversion reached ≤18%.

Table S4. Initial rates for thermolysis of **1** at various starting concentrations of mesitylene (0.01- 0.1 M) at 50 ℃.

c(Mes) M	Initial rate k_{int}[a], s^{-1}	Error margin for k_{int}[b], s^{-1}	ln c_0(Mes)	ln k_{int}	Error margin for ln k_{int}[c]
0.102346	$2.33861\cdot10^{-6}$	$1.01873\cdot10^{-7}$	-2.279398	-12.96595	0.03002
0.207493	$2.11274\cdot10^{-6}$	$1.47433\cdot10^{-7}$	-1.572659	-13.06752	0.03564
0.309147	$2.1069\cdot10^{-6}$	$5.19296\cdot10^{-8}$	-1.173939	-13.07030	0.03374
0.403316	$2.49017\cdot10^{-6}$	$1.42913\cdot10^{-7}$	-0.908034	-12.9032	0.03791
0.496516	$2.35428\cdot10^{-6}$	$1.26631\cdot10^{-7}$	-0.700140	-12.95928	0.03060

a) The initial rates were obtained from the linear plot of c(**1**) vs. time (0-1 h). b) Errors margins correspond to the standard uncertainty and were obtained the from linear least-squares analyses of the data using LINEST function in Excel. c) Errors margins correspond to the standard deviation of ln k_{int}.

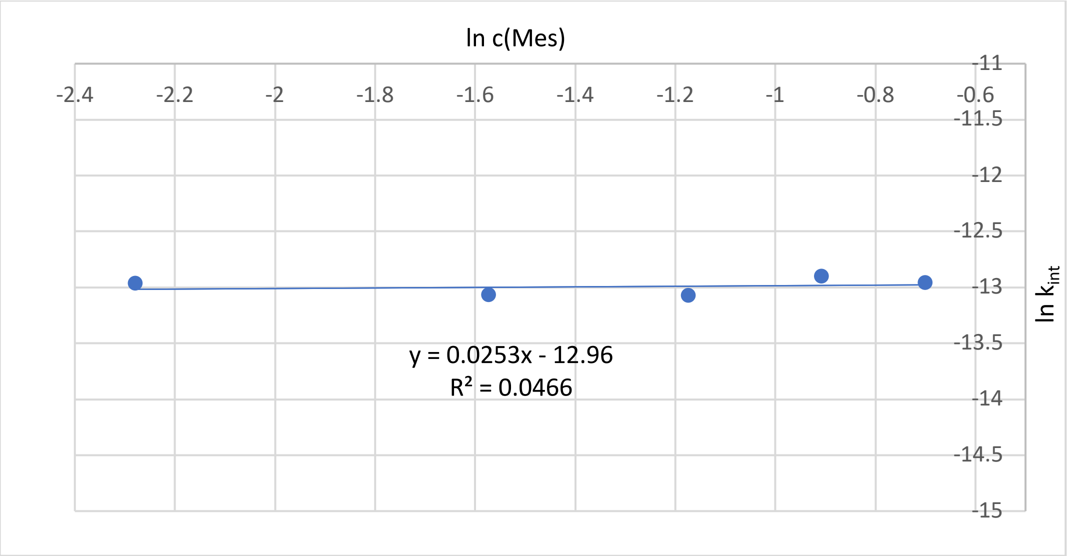

Figure S3. Determination of the order on mesitylene by calculating the slope of the line ln k_{int} vs ln c(Mes) (Table S4). The slope of 0 suggests 0th order on mesitylene.

5.3 Determination of activation parameters $\Delta H^{\neq}$ and $\Delta S^{\neq}$

In a glovebox, an NMR tube was charged with η^4–arene complex **1** (11.1 mg, 0.0248 mmol) and a stock solution (500 μL) of C_6D_{12} containing internal standard (HMDSO) to obtain 0.05 M solution of **1**. The tube was attached to an NMR tube evacuation adapter, taken out of the glovebox, frozen in liquid N_2, flame sealed under vacuum, warmed to room temperature and then fully immersed into an oil bath at 40, 50, 60, 70 or 80 °C. After the designated time interval, the NMR tube was taken out of the bath and ^{1}H NMR spectra were recorded as described in the general procedure.

Table S5. Concentration-time data for thermolysis of **1** (c=0.05 M) at 40, 50, 60, 70 and 80 °C.

Time, s	c(**1**), M	Time, s	c(**1**), M			Time, s	c(**1**), M
	40 °C		50 °C	60 °C	70 °C		80 °C
0	0.049628	0	0.050598	0.050667	0.051764	0	0.051511
1800	0.048750	900	0.048830	0.042575	0.034945	600	0.026361
3600	0.047426	1800	0.046871	0.036585	0.023396	1200	0.014906
7200	0.045852	2700	0.044439	0.031453	0.015419	1800	0.008170
14400	0.042850	3600	0.042270	0.027615	0.011100	2400	0.004684
21600	0.040405	7200	0.032408	0.016130	0.003630	3000	0.002918
28800	0.038466	14400	0.021521	0.006621	0.000460		
115200	0.018792	21600	0.014956	0.002537			
201600	0.010132	28800	0.009952	0.001614			

Table S6. Determining the activation parameters of (**1**, c=0.050 M) in the temperature range of 40-80 °C. [a]

T, °C	T, K	Rate constant k, s^{-1}[a]	Error margin for k, s^{-1}[b]	1/T (K^{-1})	ln(k/T)	Error margin for ln (k/T)
40	313	$7.899\cdot10^{-6}$	$1.12002\cdot10^{-7}$	0.003195	-17.495020	0.01418
50	323	$5.731\cdot10^{-5}$	$8.77247\cdot10^{-7}$	0.003096	-15.544715	0.03126
60	333	$1.398\cdot10^{-4}$	$4.37218\cdot10^{-6}$	0.003003	-14.683100	0.02104
70	343	$4.331\cdot10^{-4}$	$9.11083\cdot10^{-6}$	0.002915	-13.582314	0.01850
80	353	$9.945\cdot10^{-4}$	$1.83958\cdot10^{-5}$	0.002833	-12.779778	0.01418

a) The rates were obtained from the linear plot of c(Mes) vs. time. b) Errors margins correspond to the standard uncertainty and were obtained the from linear least-squares analyses of the data using LINEST function in Excel. c) Errors margins correspond to the standard deviation of ln(k/T).

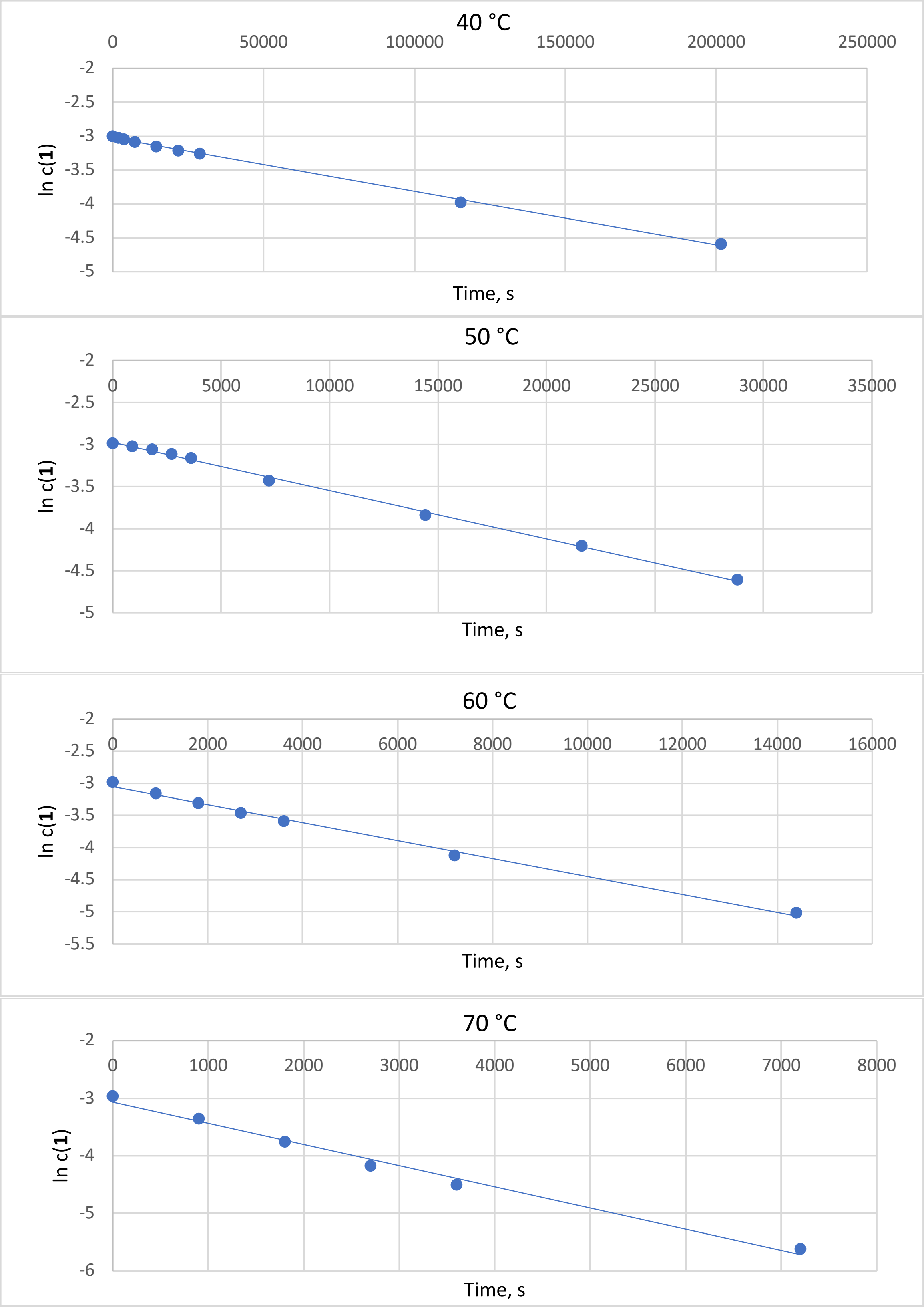

Figure S4. Plot of ln c vs time for thermolysis of **1** at different temperatures (40-80 °C). See also next page.

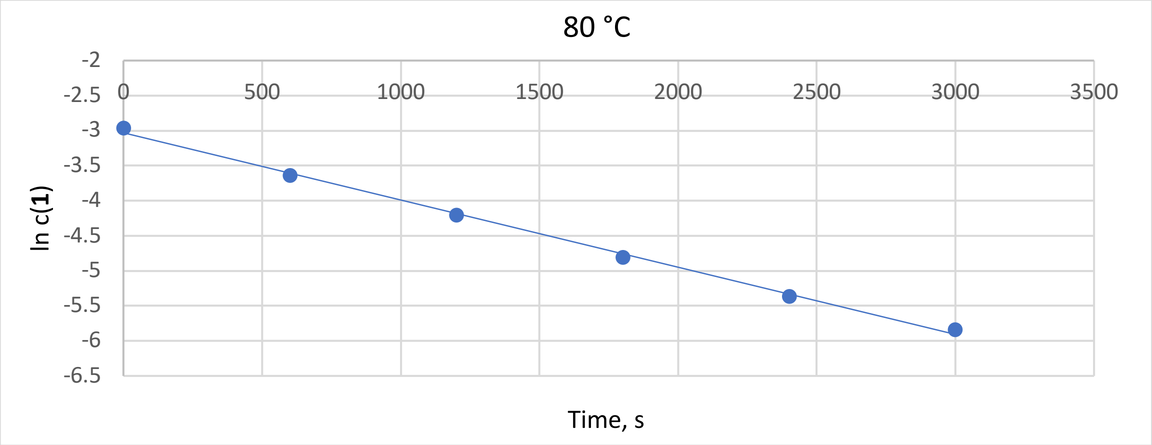

Figure S4 (continued). Plot of ln c vs time for thermolysis of **1** at different temperatures (40-80 °C).

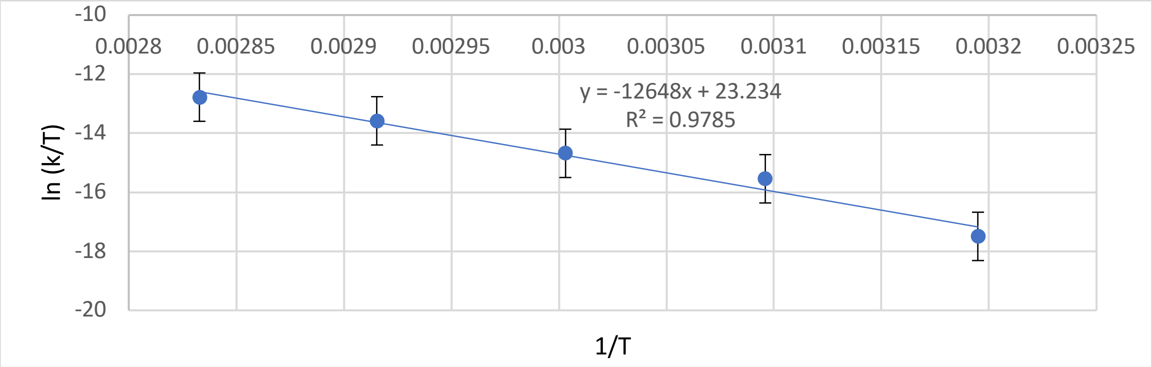

Figure S5. An Eyring plot for determining the activation parameters $\Delta H^{\neq}$ and $\Delta S^{\neq}$ for thermolysis of **1** at 40-80 °C.

Table S7. Activation parameters for thermolysis of **1**.[a]

$\Delta H^{\neq}$, kcal/mol	$\Delta S^{\neq}$, cal/(mol·K)	$\Delta G_{323}^{\neq}$, kcal/mol
25.1 ± 2.1	-1.0 ± 6.5	25.4 ± 3.0

a) Kinetic activation parameters were obtained from the linear Eyring plots of ln(k_i /T) vs. 1/T (**Fig. S5**). Errors margins correspond to the standard uncertainty and were the from linear least-squares analyses of the data using LINEST function in Excel.

5.4 Measurement of kinetic isotope effects

In a glovebox, an NMR tube was charged with η[4]–arene complex **1-d₃** (13.0 mg, 0.0288 mmol) or **1-d₉** (13.0 mg 0.0285 mmol) and a stock solution (573 µL for **1-d₃** and 560 µL for **1-d₉**) of C_6D_{12} containing internal standard (HMDSO) to obtain 0.050 M solution of **1-d₃** or **1-d₉**. The tube was attached to an NMR tube evacuation adapter, taken out of the glovebox, frozen in liquid N_2, flame sealed under vacuum, warmed to room temperature and then fully immersed into an oil bath at 50 °C. After the designated time interval, the NMR tube was taken out of the bath and ^{1}H NMR spectra were recorded as described in the general procedure.

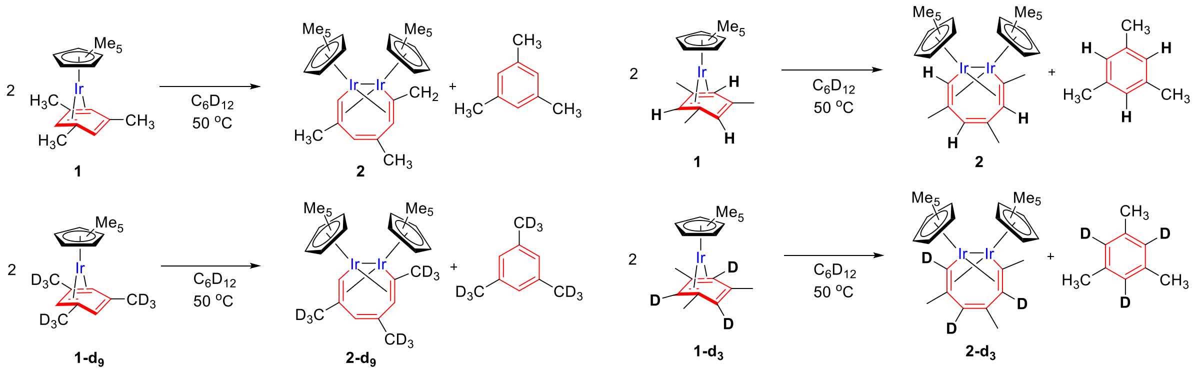

Table S8. Concentration-time data for thermolysis **1**, **1-d₃** and **1-d₉** at 50 °C.[a]

Time, s	c(**1**), M			c(**1-d₃**), M			c(**1-d₉**), M		
	run 1a	run 2a	run 3a	run 1b	run 2b	run 3b	run 1c	run 2c	run 3c
0	0.05029	0.05093	0.05056	0.05082	0.04992	0.04962	0.05082	0.04992	0.04962
900	0.04859	0.04910	0.04878	0.04911	0.04808	0.04791	0.04911	0.04808	0.04791
1800	0.04662	0.04680	0.04717	0.04712	0.04652	0.04613	0.04712	0.04652	0.04613
2700	0.04412	0.04485	0.04434	0.04490	0.04388	0.04374	0.04490	0.04388	0.04374
3600	0.04224	0.04269	0.04186	0.04287	0.04221	0.04179	0.04287	0.04221	0.04179

a) The measurements were performed during the 1st hour of the reaction when conversion was less than 18%.

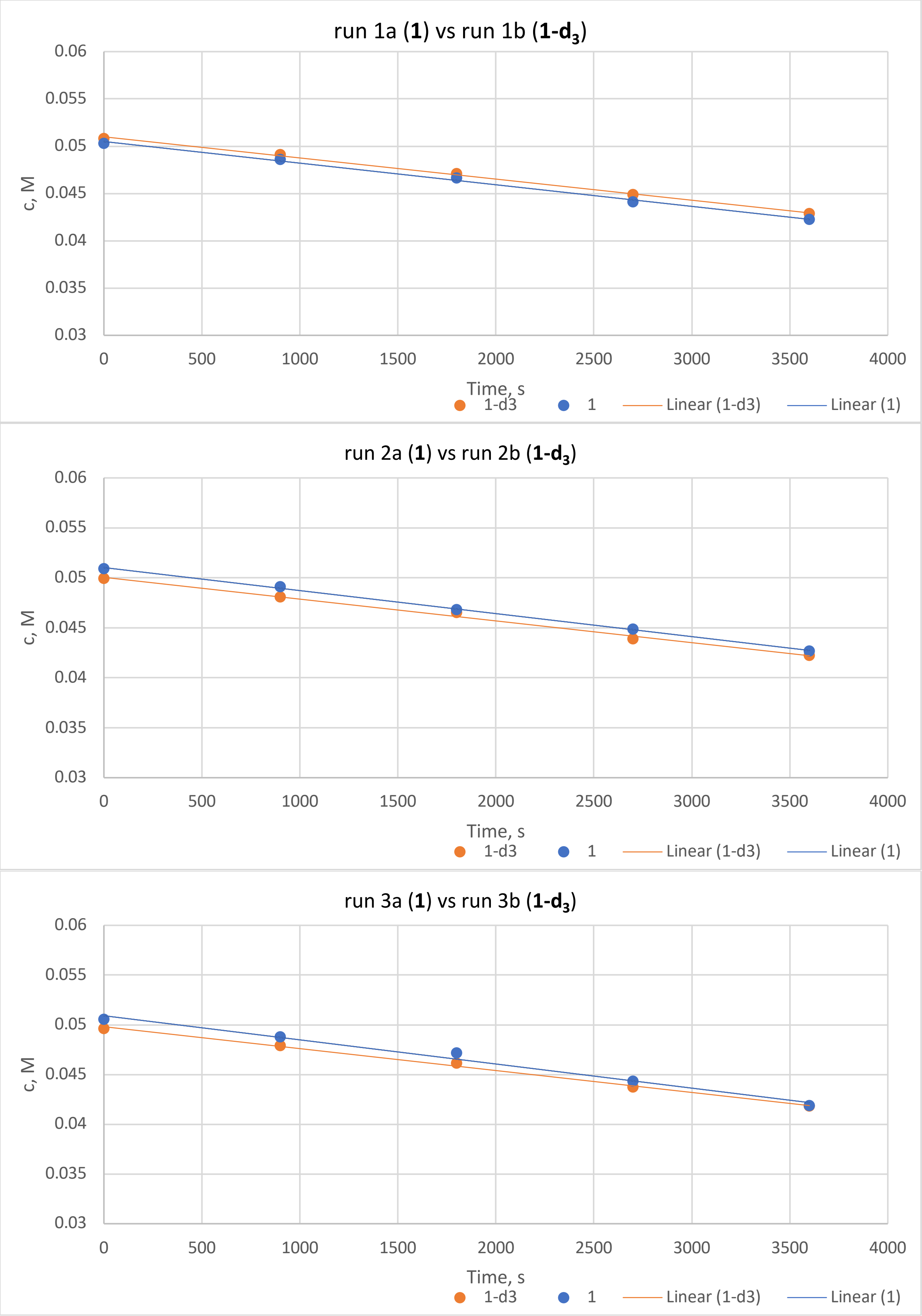

Figure S9. Concentration-time plots for three pairs of thermolysis experiments with of η^4-mesitylene **1** and η^4-mesitylene-d$_3$ **1-d$_3$** complexes at 50 °C (data from Table S8).

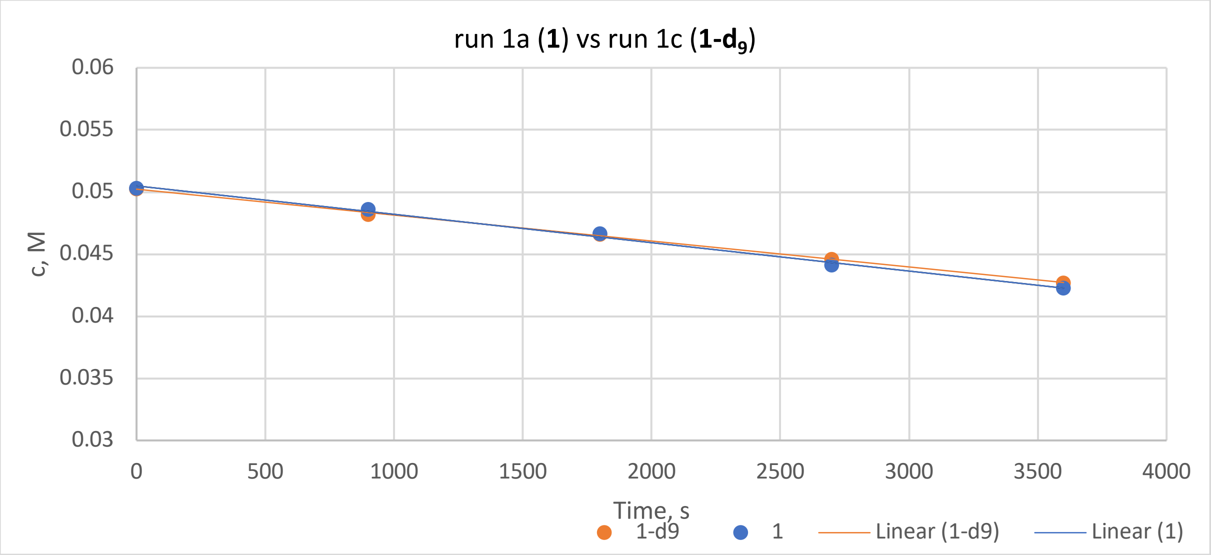

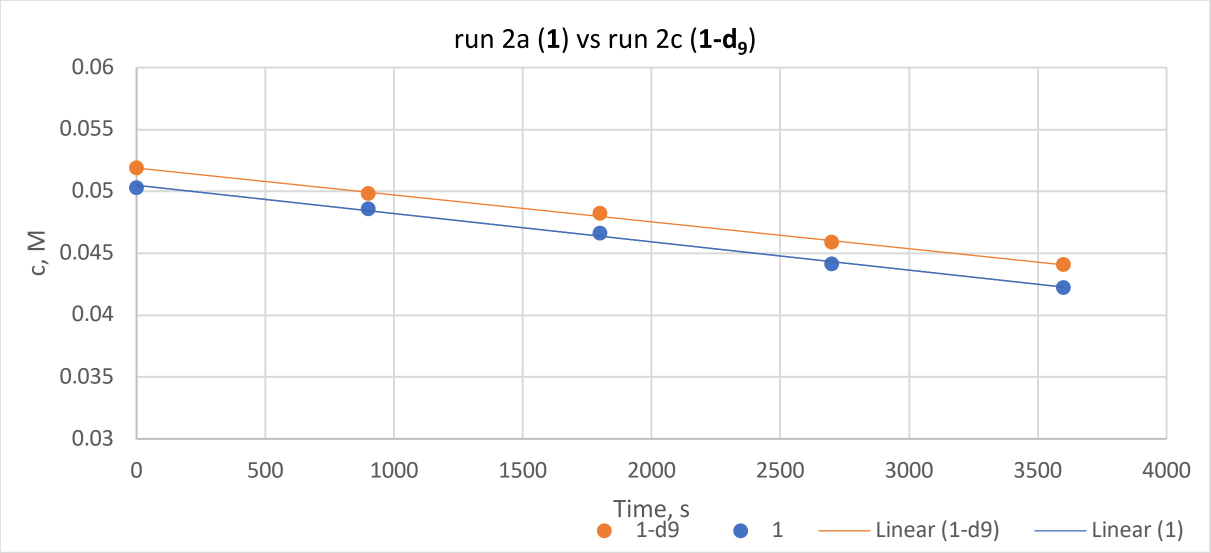

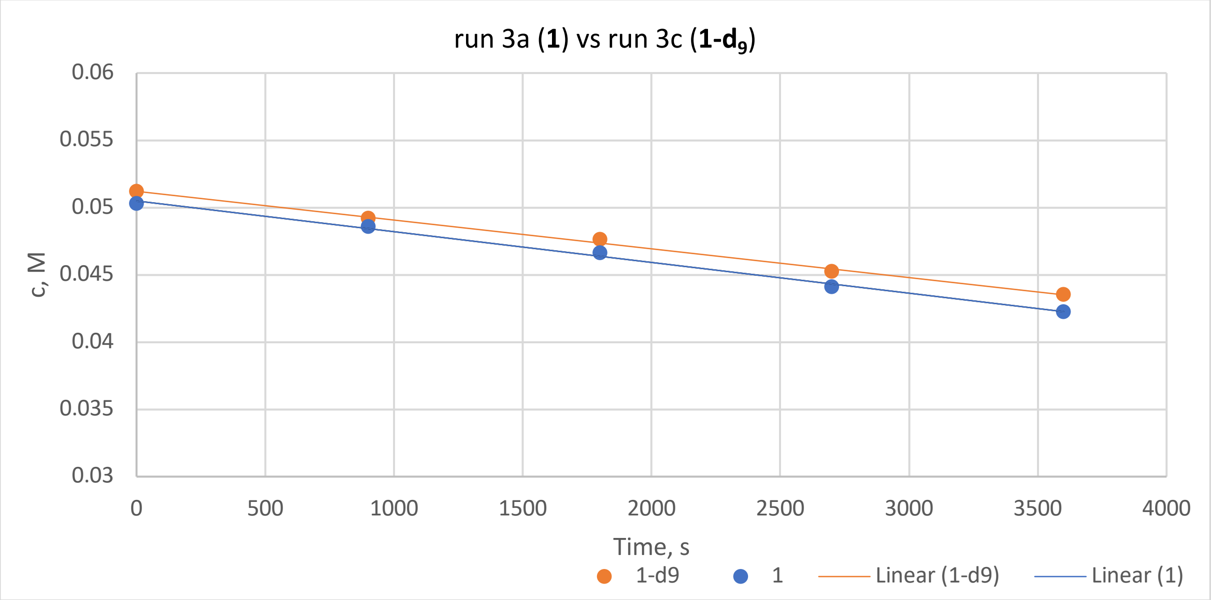

Figure S10. Concentration-time plots for three pairs of thermolysis experiments with of η^4-mesitylene **1** and η^4-mesitylene-d$_9$ **1-d$_9$** complexes at 50 °C (data from Table S8).

Table S9. Determination of KIE(**1**/**1-d$_3$**) and KIE(**1**/**1-d$_9$**) using data from Figs. S9 and S10.

	run 1	run 2	run 3
Initial rate k_{int}(**1**), s^{-1}	$2.28559 \cdot 10^{-6}$	$2.30309 \cdot 10^{-6}$	$2.42699 \cdot 10^{-6}$
Initial rate k_{int}(**1-d$_3$**), s^{-1}	$2.23494 \cdot 10^{-6}$	$2.17811 \cdot 10^{-6}$	$2.20325 \cdot 10^{-6}$
Initial rate k_{int}(**1-d$_9$**), s^{-1}	$2.08679 \cdot 10^{-6}$	$2.17168 \cdot 10^{-6}$	$2.13801 \cdot 10^{-6}$
KIE(**1**/**1-d$_3$**) = k_{int}(**1**)/ k_{int}(**1-d$_3$**)	1.02_2	1.05_7	1.10_1
KIE(**1**/**1-d$_9$**) = k_{int}(**1**)/ k_{int}(**1-d$_3$**)	1.09_5	1.06_0	1.13_5

KIE(**1**/**1-d$_3$**) = 1.06 ± 0.09 (95% confidence interval)

KIE(**1**/**1-d$_9$**) = 1.09 ± 0.09 (95% confidence interval)

7. DFT Calculations

All geometry optimizations, analytical frequency calculations and intrinsic reaction path calculations were with the B3LYP[7] functional in gas phase and a mixed basis set of LanL2DZ for iridium and 6-31G(d) for other atoms. Stabilities of converged wavefunctions were confirmed by running the “stable” test. Analytical frequency calculations confirmed that all converged minima contained 0 and all converged transition-state geometries contained exactly 1 imaginary frequency. IRCs calculations established all minima connected to each transition-state geometry. Single point energies of all converged geometries were calculated with the M06-L[8] functional and mixed basis set of LanL2TZ for the rhodium atom and 6-311+G(d) for the rest of other atoms. The solvent effects were included in single point energy calculations using the conductor polarizable continuum model (CPCM). The free energies were calculated by adding single-point energies calculated at the M06-L/(6-311+G(d)+LANL2TZ) level to thermodynamic corrections calculated in the rigid-rotor/ideal gas/quasi-harmonic approximations.[9] The effectiveness of using B3LYP for structure optimization followed by single point energy calculations with the M06 suit of functionals[10] and the suitability of B3LYP and M06-L for calculations of barrier heights involving Ir-C and C-C bond formations were reported previously.[11] All DFT calculations were performed with Gaussian 09.[12]

Table S10. Electronic activation (E_a) and reaction (E_r) energies (kcal/mol) for selected elementary steps and the overall transformation at B3LYP-D3/def2SVP and B3LYP/(6-31G(d)+LANL2DZ).

Reaction	B3LYP-D3/def2SVP		B3LYP/(6-31G(d)+LANL2DZ)	
	E_a	E_r	E_a	E_r
**1**→**6**	19.5	15.7	22.5	16.8
**6**→**7**	2.1		1.1	
**8**→**9**	21.4		23.4	
2x**1**→**2**		-31.7		-32.5

Figure S11. The full calculated mechanism and the corresponding free energy diagram of C-C cleavage in [Cp*Ir(η^4-mesitylene)] complex **1** through *syn* diiridium intermediates. All free energies are relative to 2 moles of **1**, at M06-L/(6-311+G(d)+LANL2TZ)//B3LYP/(6-31G(d)+LANL2DZ), 1 M concentrations and 50 °C. Whenever necessary to maintain the constant atomic composition, the energy states include, in addition to the species specified by its number, a mole of 1 or a mole of mesitylene.

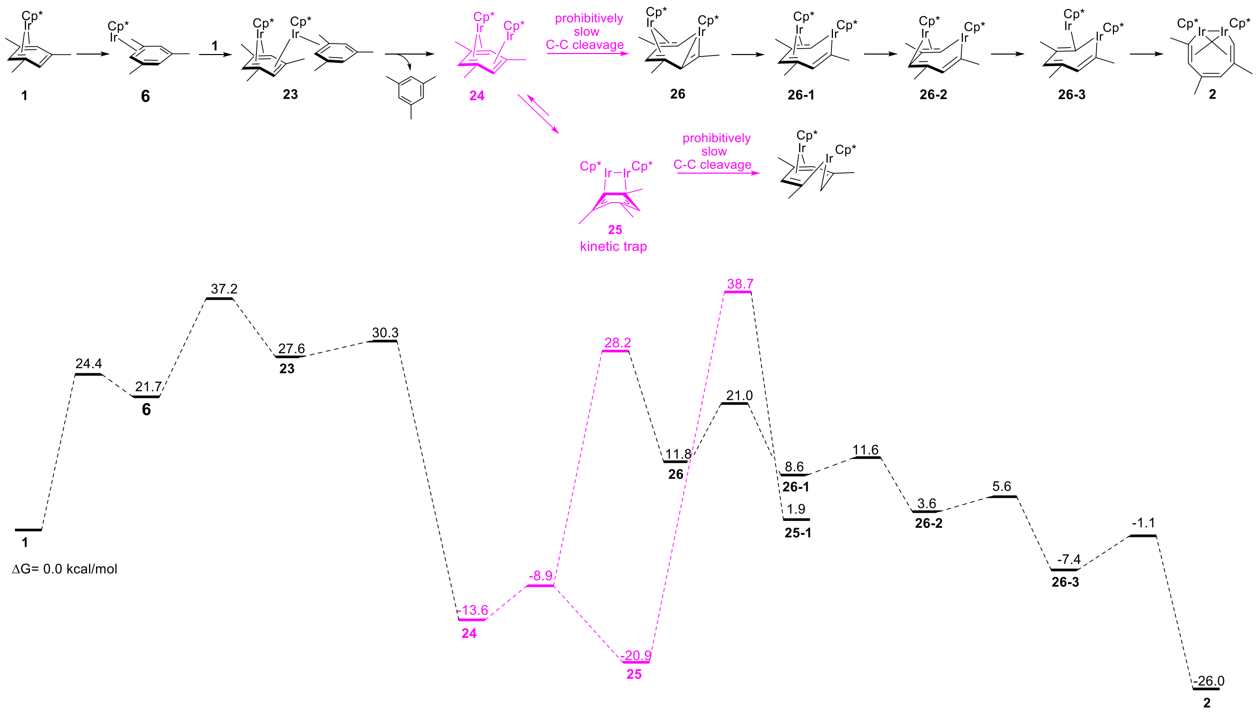

Figure S12. The calculated reaction mechanisms and the corresponding free energy diagram of C-C cleavage in [Cp*Ir(η^4-benzene] complex **5**. All free energies are relative to 2 moles of **5**, at M06-L/(6-311+G(d)+LANL2TZ)//B3LYP/(6-31G(d)+LANL2DZ), 1 M concentrations and 50 °C. Whenever necessary to maintain the constant atomic composition, the energy states include, in addition to the species specified by its number, a mole of **5** or a mole of benzene.

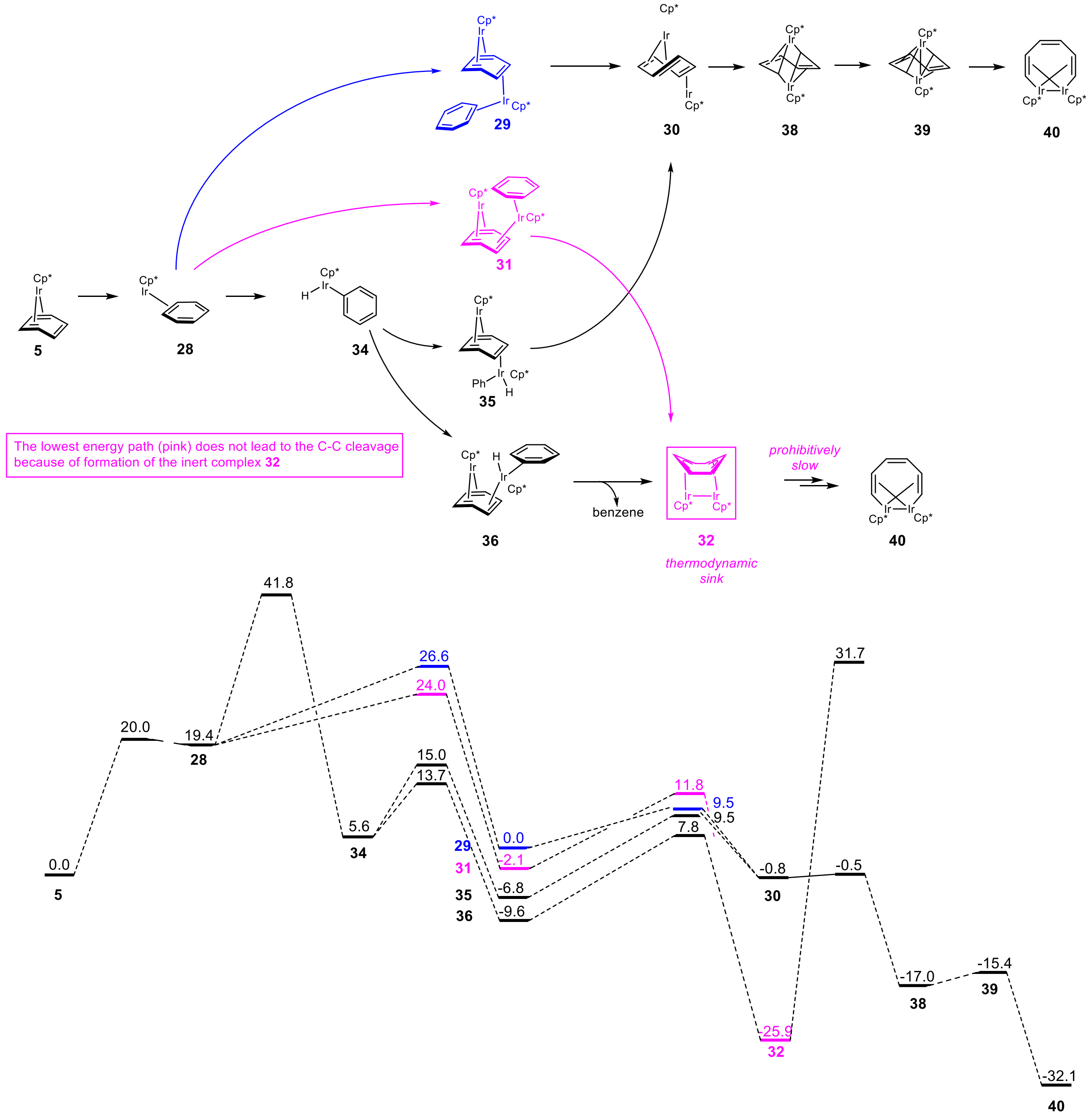

Table S11. Standard reaction free energies of the overall conversion of [Cp*Ir(η^4-arene)] (arene = benzene, *m*-xylene and mesitylene) to the corresponding metallacycles, and of the steps involving Ir-Ir bond formation, ΔG^o_{Ir-Ir}, and C-C bond scission, $\Delta G^o_{scission}$, in kcal/mol. All free energies are relative to 2 moles of [Cp*Ir(η^4-arene)], at M06-L/(6-311+G(d)+LANL2TZ)//B3LYP/(6-31G(d)+LANL2DZ), 1 M concentrations and 50 °C.

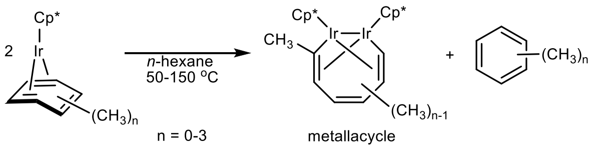

Arene complex	Metallacycle	ΔG^o_{rxn}	ΔG^o_{Ir-Ir}	$\Delta G^o_{scission}$
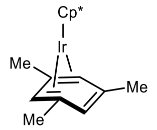	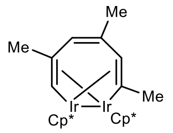	-26.0	-14.1	-8.7
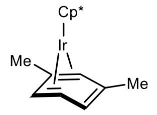	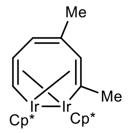	-28.9	-17.4	-9.8
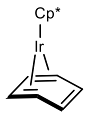	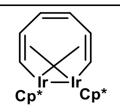	-32.1	-15.1	-16.3

Table S12. Free energy barriers of arene C-C bond scission in *anti*-[(Cp*Ir)$_2$(μ,η^4:η^2-arene)], *syn*-[(Cp*Ir)$_2$(μ,η^3:η^3-arene)] and [Cp*Ir(η^4-arene)] (arene = mesitylene, *m*-xylene and benzene) in kcal/mol. All free energies are relative to 2 moles of Cp*Ir(η^4-arene), at M06-L/(6-311+G(d)+LANL2TZ)//B3LYP/(6-31G(d)+LANL2DZ), 1 M concentrations and 50 °C.

	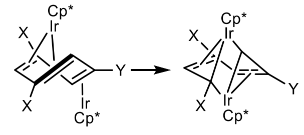	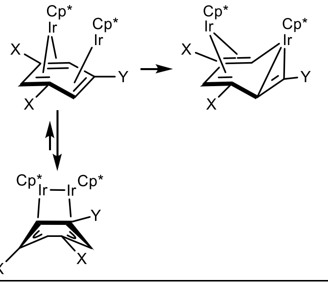	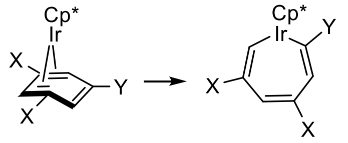
X=Y=Me	4.9	41.8	46.1
X=Me, Y=H	4.9	30.9	45.3
X=Y=H	0.2	57.3	39.1

Table S13. Standard free energies of isomerization of *anti*-[(Cp*Ir)$_2$(μ,η^4:η^2-arene)] to *syn*-[(Cp*Ir)$_2$(μ,η^3:η^3-arene)] for arene = mesitylene, *m*-xylene and benzene, in kcal/mol, relative to 2 moles of Cp*Ir(η^4-arene), at M06-L/(6-311+G(d)+LANL2TZ)//B3LYP/(6-31G(d)+LANL2DZ), 1 M concentrations and 50 °C.

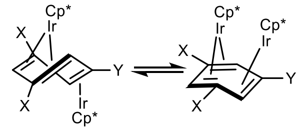	X=Y=Me	X=Me, Y=H	X=Y=H
$\Delta G^{\ddagger}_o$, kcal/mol	-10.2	-13.4	-14.3

Table S14. The electronic energies (at B3LYP/(6-31G(d)+LANL2DZ)) of the products of binding of [Cp*Ir(η^4-arene)] to either [Cp*Ir(η^2-arene)] or its Ir^{III} isomer, [Cp*Ir(H)(η^3-(CH_2)$C_6H_3Me_2$)] relative to 2 moles of Cp*Ir(η^4-arene). The lowest-energy product is highlighted in green; the last column indicates if arene cleavage is observed experimentally.

	Structures and relative electronic energies of early diirdium arene intermediates*				Arene cleavag e Expecte d?
	Ir(I)Ir(III)		Ir(I)Ir(I)		
	anti	*syn*	*anti*	*syn*	
Ar	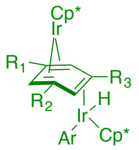	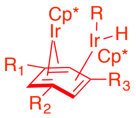	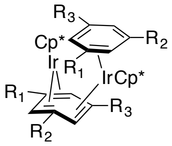	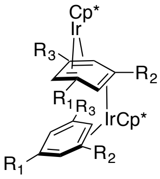	
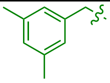	-14.8	-2.5	0.4	-2.8	Yes
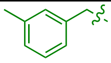	-12.3	-1.0	6.2	-0.4	Yes
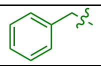	-13.7	-2.9	5.9	0.3	Yes
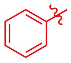	-20.5	-24.0	-15.3	-18.1	No

Table S15. The electronic energies of *syn*-Ir(I)Ir(III) intermediate **38** relative to that of *anti*-Ir(I)Ir(III) analog, **37** at M06-L/(6-311+G(d)+LANL2TZ)/B3LYP/(6-31G(d)+LANL2DZ)). Arenes that form the more stable *syn* isomer are highlighted in red, more stable *anti* – in green.

Ar	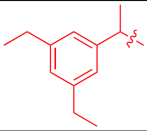	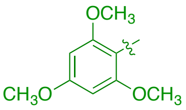	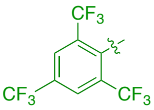
ΔEsyn/anti, kcal/mol	-0.8	4.3	6.9
Excess of short H…H contacts[1] in syn (**38**) vs. anti (**37**) intermediates	-3	4	1[3]
Arene cleavage expected?	no	yes	yes

.All short (<2.60 Å)interactions between any two hydrogen atoms located in different ligands (e.g. between Ar and Cp*, Ar and ArH etc.). [3] The *syn* isomer has also 2 short H…F (<2.67 Å) and 1 F…F short (<2.96 Å) contacts.

Table S16. Cartesian coordinates for all calculated structures.

Compound	Cartesian coordinates, Å
1	6 6.642471 6.609514 1.148416; 6 6.339879 7.312306 -0.074021; 6 4.974421 7.786871 -0.010205; 6 4.438006 7.417830 1.257724; 6 5.473812 6.734873 1.997210; 6 7.859530 5.772642 1.422180; 1 8.744635 6.177647 0.922400; 1 7.722235 4.739304 1.070026; 1 8.082991 5.729395 2.492308; 6 7.220405 7.360255 -1.289814; 1 7.059286 8.271232 -1.873785; 1 7.018384 6.504927 -1.949869; 1 8.279377 7.324569 -1.018263; 6 4.238303 8.486462 -1.116897; 1 3.471190 9.163645 -0.727595; 1 3.734222 7.766657 -1.778098; 1 4.915809 9.078731 -1.739499; 6 3.038920 7.674530 1.738604; 1 3.000484 7.785345 2.826801; 1 2.366863 6.845990 1.472266; 1 2.621848 8.586498 1.299235; 6 5.299969 6.075404 3.336609; 1 6.251136 6.004680 3.872045; 1 4.904739 5.055950 3.224492; 1 4.602767 6.629764 3.972721; 6 7.892528 10.316156 1.267071; 6 6.619388 10.894429 1.688931; 1 6.025710 11.550946 1.057081; 6 6.154095 10.450646 2.967439; 6 7.079736 9.485062 3.571464; 1 6.715964 8.981861 4.468185; 6 8.540443 9.794443 3.522433; 6 8.947867 10.222203 2.317981; 1 9.960545 10.562073 2.106052; 6 8.366204 10.587895 -0.145587; 1 9.017119 9.784487 -0.508368; 1 8.947914 11.521156 -0.191964; 1 7.526579 10.681443 -0.842658; 6 4.879495 10.926295 3.611069; 1 4.129905 11.213488 2.865478; 1 5.072327 11.800673 4.248161; 1 4.447404 10.145789 4.247227; 6 9.382507 9.689039 4.761125; 1 10.431129 9.929342 4.555829; 1 9.344934 8.674303 5.183111; 1 9.028470 10.367666 5.550436; 77 6.366153 8.766888 1.661412;
2	77 -0.467724 11.948633 5.473735; 77 -0.802661 9.534421 4.121199; 6 -1.165131 7.670795 2.861354; 6 -0.316041 11.539461 3.521161; 6 -0.520658 14.141227 5.857261; 6 0.112374 9.218213 7.058177; 6 -0.199626 7.015178 1.916334; 6 -2.241034 8.567814 2.492366; 6 0.311994 9.953492 5.747681; 6 -0.546927 6.276327 4.984105; 6 1.438117 10.869830 5.703631; 6 0.989929 13.721366 7.956087; 6 2.449282 10.910773 4.646716; 6 3.888826 11.062231 5.075267; 6 -1.321518 7.334759 4.252749; 6 -2.446672 8.082152 4.759727; 6 -0.244043 13.504381 7.127709; 6 0.720136 10.787605 2.874052; 6 -3.013538 8.831119 3.656721; 6 -1.397056 12.743763 7.520697; 6 2.097860 10.750337 3.355281; 6 -2.383169 12.876314 6.487621; 6 -1.848911 13.728602 5.453372; 6 0.330528 15.180582 5.184811; 6 -2.599241 14.241310 4.257281; 6 -1.576536 12.008859 8.818115; 6 -3.781431 12.334774 6.542994; 6 -3.104100 7.907427 6.099121; 6 -4.279309 9.635647 3.704959; 6 -2.579964 9.025824 1.102063; 1 -1.043371 12.004822 2.847190; 1 0.551356 9.772416 7.899931; 1 0.622919 8.244493 7.021240; 1 -0.940580 9.035467 7.287186; 1 0.740766 6.765218 2.415797; 1 0.040003 7.661158 1.067637; 1 -0.621606 6.083565 1.511846; 1 0.490266 6.229895 4.639676; 1 -0.996605 5.285721 4.823771; 1 -0.528292 6.457667 6.061972; 1 1.861520 11.085068 6.690186; 1 1.222181 12.848461 8.574112; 1 0.860787 14.578440 8.631965; 1 1.862395 13.923027 7.328146; 1 4.052971 12.020825 5.588189; 1 4.568160 11.025982 4.216880; 1 4.189996 10.274778 5.780996; 1 2.872786 10.651802 2.595064; 1 1.395428 14.995384 5.351888; 1 0.102009 16.186457 5.567616; 1 0.168002 15.192200 4.103326; 1 -1.922538 14.453484 3.423719; 1 -3.136968 15.170905 4.491094; 1 -3.336718 13.513725 3.903706; 1 -2.200783 11.117991 8.695322; 1 -2.058007 12.645771 9.574197; 1 -0.618231 11.681533 9.232186; 1 -4.200946 12.200948 5.542736; 1 -4.443285 13.023902 7.087607; 1 -3.823073 11.369709 7.057832; 1 -2.426074 7.457189 6.828296; 1 -3.979723 7.247433 6.017940; 1 -3.449717 8.861682 6.510158; 1 -4.465170 10.031899 4.706240; 1 -5.147216 9.018601 3.429036; 1 -4.247225 10.480857 3.009991; 1 -2.930538 10.063321 1.085117; 1 -3.380981 8.404845 0.675525; 1 -1.722643 8.952733 0.428827; 6 0.619276 10.636658 1.360594; 1 1.200899 11.431869 0.870509; 1 1.032006 9.682326 1.013827; 1 -0.411582 10.715040 1.008729;
3	77 4.726294 8.173354 1.520857; 6 6.178935 7.189039 2.832043; 6 5.716077 6.466618 4.079892; 6 6.061159 6.490509 1.569467; 1 5.692799 5.470830 1.480854; 6 6.337402 7.284358 0.407492; 6 6.253316 6.750219 -0.996444; 6 6.694266 8.658308 0.729164; 1 6.735500 9.342387 -0.118789; 6 7.606954 8.979176 1.844684; 6 7.408154 8.073550 3.041662; 6 2.862707 8.637807 2.719855; 6 2.417384 7.790859 1.638307; 6 2.681636 8.471756 0.404087; 6 3.312651 9.728162 0.716455; 6 3.396863 9.855259 2.158655; 6 2.643476 8.375686 4.182107; 1 1.660315 8.749605 4.500664; 1 3.399803 8.872770 4.796601; 1 2.679747 7.307306 4.413448; 6 1.708584 6.474822 1.786069; 1 2.014320 5.953133 2.697875; 1 1.911985 5.809615 0.940903; 1 0.619084 6.614276 1.838906; 6 2.316090 7.983905 -0.968305; 1 2.346949 6.891355 -1.029966; 1 2.995983 8.376965 -1.730369; 1 1.299449 8.299079 -1.244054; 6 3.667308 10.804594 -0.270314; 1 4.503502 11.412668 0.086941; 1 2.815539 11.478187 -0.439774; 1 3.952959 10.385836 -1.240314; 6 3.803456 11.086788 2.916854; 1 4.595983 11.633839 2.397785; 1 4.180811 10.836723 3.912896; 1 2.954034 11.773650 3.046782; 1 4.868285 5.803953 3.873936; 1 5.409980 7.174147 4.859313; 1 6.528017 5.854081 4.502408; 1 5.535545 5.925323 -1.070327; 1 7.232267 6.375273 -1.325883; 1 5.949402 7.534523 -1.698273; 1 8.300812 7.449904 3.219443; 1 7.269481 8.675297 3.949272; 6 8.475354 10.000918 1.815865; 1 8.582677 10.631489 0.936279; 1 9.102648 10.244237 2.669920;
4	77 11.834077 18.854075 19.837442; 6 13.386425 14.269358 16.674163; 1 13.966676 13.447385 16.257577; 6 11.694101 12.578285 17.504035; 1 11.664422 12.275119 18.559003; 1 12.334958 11.866486 16.973364; 6 12.194913 13.997039 17.346404; 6 11.460566 15.071663 17.874427; 1 10.511772 14.867273 18.369768; 6 12.101195 21.154253 19.850815; 6 10.323953 20.255224 21.063571; 6 11.062726 20.786270 18.918750; 6 9.951153 20.255358 19.691064; 6 11.893745 16.399842 17.760179; 6 11.664714 20.809257 21.167957; 6 13.097121 16.630232 17.063587; 1 13.451560 17.653451 16.962770; 6 13.845139 15.588833 16.516493; 6 11.078834 17.534410 18.313220; 1 10.115396 17.143064 18.665267; 1 10.862384 18.228061 17.490444; 6 11.018418 21.125508 17.456127; 1 12.024243 21.211715 17.034324; 1 10.502601 22.081184 17.282839; 1 10.485769 20.361849 16.879323; 6 13.372939 21.877075 19.505566; 1 14.175816 21.636552 20.208260; 1 13.221434 22.965657 19.531879; 1 13.722777 21.615095 18.503361; 6 9.443279 19.895626 22.227650; 1 8.668007 19.177933 21.943496; 1 8.935941 20.784968 22.628910; 1 10.015041 19.455811 23.051420; 6 8.620367 19.861661 19.118414; 1 8.732630 19.276462 18.199593; 1 8.022298 20.749587 18.869868; 1 8.038994 19.259651 19.823027; 6 15.121883 15.864341 15.753303; 1 15.944507 15.227276 16.101146; 1 15.435310 16.907670 15.862716; 1 14.997301 15.666958 14.680108; 6 12.363126 21.175341 22.447760; 1 12.112914 20.489269 23.262041; 1 12.077557 22.185076 22.778118; 1 13.451244 21.167296 22.331233; 1 10.675193 12.466956 17.112099; 15 12.391282 17.123419 21.199778; 6 13.852600 16.101858 20.720924; 1 13.695433 15.681163 19.725330; 1 14.737255 16.744711 20.686149; 1 14.019927 15.291702 21.440067; 6 12.842105 17.587179 22.937336; 1 13.674158 18.296984 22.924357; 1 11.989702 18.064318 23.430120; 1 13.133035 16.703016 23.516348; 6 11.103311 15.830682 21.525270; 1 10.200876 16.308921 21.918985; 1 10.849689 15.330080 20.588026; 1 11.457530 15.082999 22.244603; 1 13.243676 18.553307 19.155448;
5	6 6.646850 6.613444 1.140030; 6 6.329425 7.333033 -0.074334; 6 4.957318 7.785397 0.000557; 6 4.439149 7.410572 1.273934; 6 5.486634 6.723996 1.997841; 6 7.873758 5.785518 1.395435; 1 8.749899 6.202574 0.890124; 1 7.741433

	4.754128 1.036182; 1 8.108264 5.736884 2.462911; 6 7.208979 7.418331 -1.289890; 1 7.027449 8.336950 -1.856409; 1 7.025312 6.571469 -1.965930; 1 8.267727 7.401528 -1.016628; 6 4.205674 8.492907 -1.090005; 1 3.445883 9.168724 -0.684392; 1 3.690461 7.777178 -1.746819; 1 4.874569 9.087467 -1.719550; 6 3.042589 7.652750 1.769729; 1 3.012096 7.740156 2.859977; 1 2.372338 6.827168 1.489964; 1 2.619510 8.572222 1.352438; 6 5.328464 6.058919 3.336261; 1 6.287378 5.977340 3.855692; 1 4.922517 5.043828 3.223492; 1 4.646227 6.615777 3.986058; 6 7.942779 10.217947 1.287220; 6 6.678455 10.877624 1.613621; 1 6.139173 11.532110 0.935519; 6 6.164431 10.498162 2.890691; 6 7.017326 9.534852 3.587054; 1 6.598363 9.077692 4.481334; 6 8.482611 9.795530 3.566167; 6 8.965209 10.151713 2.367006; 1 9.992796 10.456898 2.183723; 77 6.362229 8.759411 1.655594; 1 8.295860 10.330903 0.264072; 1 5.202936 10.840824 3.260928; 1 9.067642 9.774075 4.482659;
1-6_ts	77 -0.763382 2.692244 4.820227; 6 -2.576652 3.156152 5.815703; 6 -1.628740 2.529584 6.762703; 6 0.543474 3.774587 3.467473; 6 -0.284907 2.970807 2.612677; 6 -0.072936 1.620355 3.055957; 6 1.109894 1.576872 3.947599; 6 1.489866 2.896240 4.199463; 6 2.609098 3.390911 5.064174; 6 1.736285 0.319362 4.470694; 6 -0.741996 0.400288 2.496559; 6 -1.213163 3.444115 1.533723; 6 0.651413 5.269611 3.433461; 1 -1.762862 1.464752 6.975551; 6 -1.050550 3.310412 7.856830; 6 -0.241273 2.584212 8.899491; 6 -1.273407 4.653225 7.913781; 1 -0.843154 5.239596 8.724125; 6 -2.090858 5.331202 6.930294; 6 -2.263013 6.826849 7.042031; 6 -2.708971 4.609693 5.949533; 1 -3.371101 5.114858 5.246468; 6 -3.816342 2.410543 5.336170; 1 -1.751879 0.627150 2.143269; 1 -0.818787 -0.391774 3.247689; 1 -0.169847 -0.001043 1.647634; 1 -2.072890 2.776054 1.421994; 1 -0.704594 3.492262 0.559079; 1 -1.602637 4.443674 1.750330; 1 -0.291716 5.729178 3.125383; 1 1.430352 5.584828 2.724268; 1 0.913533 5.670818 4.416831; 1 3.009581 2.594266 5.697649; 1 2.276539 4.200437 5.722478; 1 3.437492 3.781931 4.455835; 1 0.977983 -0.410532 4.771962; 1 2.368287 0.516399 5.341385; 1 2.363212 -0.159383 3.704495; 1 -4.174271 2.815521 4.382020; 1 -4.632777 2.504994 6.068109; 1 -3.618730 1.342986 5.192217; 1 -2.944548 7.212504 6.276544; 1 -1.303620 7.351949 6.931720; 1 -2.663937 7.111035 8.024607; 1 0.608406 2.057975 8.444220; 1 -0.845220 1.822838 9.413049; 1 0.149543 3.271061 9.657542;
6	77 -0.788565 2.353181 4.943735; 6 -2.560308 2.995287 5.912225; 6 -1.515105 2.656180 6.903139; 6 0.555967 3.630001 3.941377; 6 -0.261227 3.084697 2.845913; 6 -0.048640 1.691594 2.860982; 6 1.029973 1.379328 3.823588; 6 1.465591 2.571794 4.419714; 6 2.601695 2.772770 5.376680; 6 1.585545 0.002468 4.038687; 6 -0.698769 0.669059 1.976015; 6 -1.145963 3.881985 1.934734; 6 0.712043 5.088013 4.250690; 1 -1.584767 1.678428 7.395592; 6 -0.921275 3.717078 7.717306; 6 0.050211 3.324305 8.799721; 6 -1.281786 5.013381 7.498488; 1 -0.837478 5.802907 8.103382; 6 -2.276325 5.384324 6.512135; 6 -2.624511 6.845289 6.354513; 6 -2.888591 4.413762 5.776224; 1 -3.678916 4.684373 5.075669; 6 -3.705622 2.011072 5.682968; 1 -1.629950 1.046421 1.544243; 1 -0.936217 -0.245728 2.529243; 1 -0.035441 0.384953 1.146489; 1 -1.907771 3.253099 1.464099; 1 -0.564338 4.356221 1.130547; 1 -1.665453 4.676911 2.479026; 1 -0.222040 5.629534 4.079741; 1 1.489484 5.535090 3.614220; 1 0.995871 5.242471 5.295493; 1 2.884238 1.837470 5.869376; 1 2.342987 3.494059 6.158316; 1 3.493267 3.156662 4.858944; 1 0.789418 -0.747575 4.091239; 1 2.161128 -0.058962 4.966575; 1 2.250725 -0.287913 3.212687; 1 -4.203222 2.201062 4.724798; 1 -4.459760 2.099963 6.480171; 1 -3.364340 0.968161 5.677246; 1 -3.420463 6.991519 5.616611; 1 -1.755452 7.435740 6.031016; 1 -2.962481 7.281803 7.304392; 1 0.903837 2.771336 8.385885; 1 -0.425878 2.659855 9.535005; 1 0.434874 4.198172 9.336162;
6-7_ts	77 12.204184 15.246799 20.437301; 1 10.589510 15.804780 20.607852; 6 13.001016 13.954629 16.903972; 1 13.597300 13.303840 16.265792; 6 11.948860 11.903211 17.904779; 1 12.165208 11.543461 18.918795; 1 12.595447 11.364494 17.204554; 6 12.134963 13.394988 17.804333; 6 11.317484 14.248154 18.651443; 1 10.348656 13.841793 18.942313; 6 14.103339 14.246562 20.968822; 6 12.947992 15.481284 22.534667; 6 14.538909 15.633085 20.846351; 6 13.814594 16.394581 21.788330; 6 11.468766 15.690592 18.484272; 6 13.213037 14.135947 22.107241; 6 12.357121 16.220838 17.483288; 1 12.410876 17.298904 17.344464; 6 13.115574 15.380494 16.713436; 6 10.602746 16.465530 19.387704; 1 9.528325 16.254655 19.318595; 1 10.792608 17.535253 19.462530; 6 15.536182 16.124352 19.839165; 1 15.257571 15.823536 18.822137; 1 16.538322 15.720616 20.039650; 1 15.612911 17.215646 19.849994; 6 14.741424 13.099749 20.238714; 1 14.142410 12.188802 20.323183; 1 15.736436 12.882212 20.652221; 1 14.860955 13.319693 19.173368; 6 12.123653 15.872713 23.729734; 1 11.670986 16.860920 23.602035; 1 12.742800 15.908972 24.637464; 1 11.314525 15.158511 23.906789; 6 13.917418 17.872612 22.034764; 1 14.289921 18.405318 21.154503; 1 14.601040 18.092267 22.867589; 1 12.945814 18.304993 22.294730; 6 14.045497 15.901824 15.643671; 1 15.080562 15.576519 15.813138; 1 14.040394 16.996032 15.605860; 1 13.758147 15.530604 14.650440; 6 12.749625 12.870012 22.767678; 1 11.780562 13.004364 23.257408; 1 13.465683 12.535767 23.534210; 1 12.639361 12.056270 22.044753; 1 10.908063 11.623338 17.689321;
7	77 11.980106 15.168902 20.460084; 1 10.586626 14.552097 20.929699; 6 12.976962 14.034831 16.800669; 1 13.591596 13.400305 16.164125; 6 11.807983 11.971381 17.648067; 1 11.987477 11.570008 18.653697; 1 12.465212 11.446867 16.947181; 6 12.033518 13.461387 17.616060; 6 11.201625 14.300486 18.442773; 1 10.250847 13.889220 18.763266; 6 14.063600 14.276173 21.010009; 6 12.786728 15.598509 22.443276; 6 14.323371 15.665575 20.690586; 6 13.548674 16.474172 21.570987; 6 11.380962 15.732886 18.400024; 6 13.153522 14.222479 22.110860; 6 12.376480 16.271350 17.508182; 1 12.487036 17.352577 17.456221; 6 13.141779 15.458757 16.712350; 6 10.672726 16.505652 19.406237; 1 9.620467 16.291996 19.578342; 1 10.905953 17.569921 19.449481; 6 15.294508 16.134026 19.647934; 1 15.170162 15.585985 18.708163; 1 16.331679 15.988156 19.981539; 1 15.167425 17.197630 19.426870; 6 14.730158 13.114407 20.333182; 1 14.252864 12.167221 20.598907; 1 15.788125 13.046500 20.622912; 1 14.691932 13.214394 19.242352; 6 12.016388 16.029344 23.659394; 1 11.516067 16.988373 23.496189; 1 12.681512 16.141500 24.528083; 1 11.246043 15.298842 23.920971; 6 13.554930 17.973903 21.645890; 1 13.784851 18.427606 20.677114; 1 14.309010 18.326780 22.363416; 1 12.584653 18.359922 21.971216; 6 14.137092 16.022719 15.725728; 1 15.147537 15.632071 15.903079; 1 14.183793 17.114855 15.782122; 1 13.870108 15.750986 14.695746; 6 12.758052 13.000342 22.890707; 1 11.758233 13.108263 23.319763; 1 13.459189 12.819928 23.718068; 1 12.748837 12.106270 22.260691; 1 10.769603 11.723645 17.389984;
7-8_ts	77 -0.704251 1.912223 3.745885; 6 -1.628130 -0.200366 3.375840; 6 -0.226718 -0.252710 3.234082; 6 0.132162 0.742956 2.214326; 6 -1.115639 1.215146 1.585316; 6 -2.178119 0.698651 2.344202; 6 -3.644062 0.933299 2.129274; 6 -1.188549 2.101209 0.379006; 6 1.505037 0.952565 1.648681; 6 0.738405 -1.130248 3.974828; 6 -2.467900 -1.005656 4.322917; 1 2.274430 0.759957 2.401034; 1 1.635855 1.981279 1.300801; 1 1.681534 0.276076 0.799580; 1 1.682341 -0.611576 4.163641; 1 0.965121 -2.037238 3.396512; 1 0.335976 -1.443762 4.942317; 1 -1.883972 -1.356558 5.177924; 1 -2.888617 -1.888089 3.820151; 1 -3.308820 -0.421888 4.710768; 1 -3.825615 1.824732 1.522897; 1 -4.173368 1.063159 3.078720; 1 -4.105535 0.079145 1.613839; 1 -0.391969 2.851200 0.389465; 1 -2.143903 2.631196 0.321924; 1 -1.077827 1.514761 -0.543857; 6 2.432113 5.593656 1.847993; 6 1.163791 5.038356

	2.079445; 1 0.457167 4.971829 1.252480; 6 0.769641 4.588716 3.346587; 6 1.691782 4.724911 4.401917; 1 1.408251 4.371500 5.390905; 6 2.956886 5.277226 4.207344; 6 3.319729 5.704400 2.918720; 1 4.307290 6.133005 2.754327; 6 2.812376 6.082681 0.467602; 1 2.226312 6.965516 0.179045; 1 2.631523 5.316728 -0.297001; 1 3.870695 6.359860 0.418876; 6 -0.595782 3.998065 3.562893; 6 3.926584 5.426234 5.359029; 1 4.108188 6.482771 5.597671; 1 4.901440 4.980725 5.124175; 1 3.547475 4.942274 6.265100; 1 -1.259924 4.310880 2.745441; 1 -1.020412 4.372418 4.506389; 1 0.295336 2.115185 4.991451; 77 -2.383120 4.275294 9.232043; 6 -4.015929 3.924852 10.715842; 6 -3.759473 5.349301 10.643686; 6 -2.814548 3.316337 11.233426; 6 -5.329998 3.241623 10.466126; 6 -2.455987 5.613124 11.207068; 6 -4.766293 6.393116 10.248870; 6 -1.868053 4.362379 11.557254; 6 -2.654191 1.862470 11.574763; 1 -5.902439 3.748281 9.683372; 1 -5.951618 3.226199 11.374223; 1 -5.188533 2.205004 10.145572; 6 -1.840143 6.969138 11.399165; 1 -4.283947 7.283337 9.833192; 1 -5.359588 6.714396 11.116694; 1 -5.461596 6.011528 9.495636; 6 -0.526206 4.162034 12.201901; 1 -3.249849 1.228357 10.911698; 1 -2.981910 1.665732 12.605360; 1 -1.612567 1.538175 11.492971; 1 -0.747427 6.927429 11.345599; 1 -2.103796 7.393124 12.378767; 1 -2.180516 7.677458 10.637377; 1 -0.095952 3.190474 11.941033; 1 -0.599694 4.202129 13.298314; 1 0.188323 4.932432 11.894483; 6 -1.450258 5.120113 7.495898; 6 -2.877298 4.915525 7.221665; 6 -0.797731 3.894618 7.837796; 6 -3.269619 3.752640 6.375124; 1 -3.469859 5.823944 7.108883; 6 -1.659571 2.718443 7.834801; 1 0.244505 3.865487 8.145260; 6 -2.626202 2.617690 6.703205; 1 -2.743932 1.675514 6.171092; 6 -4.249050 3.934550 5.252236; 1 -3.865909 4.632745 4.494914; 1 -4.470173 2.985515 4.752570; 1 -5.196671 4.354898 5.618679; 6 -1.087413 1.414725 8.348075; 1 -0.365381 1.579635 9.154997; 1 -1.875478 0.756126 8.731249; 1 -0.573022 0.878921 7.536485; 6 -0.749935 6.449487 7.413246; 1 -0.349637 6.608443 6.402836; 1 -1.441935 7.270654 7.630970; 1 0.086535 6.510615 8.118166;
8	77 -0.883357 2.422808 4.342890; 6 -1.566295 0.208790 3.756436; 6 -0.141271 0.347837 3.671432; 6 0.142555 1.313276 2.636134; 6 -1.128235 1.720237 2.062419; 6 -2.173736 1.045399 2.748437; 6 -3.632041 1.077051 2.389365; 6 -1.300181 2.597404 0.857969; 6 1.498880 1.584984 2.048957; 6 0.878847 -0.477175 4.405518; 6 -2.301823 -0.778834 4.615412; 1 2.280565 1.542116 2.812431; 1 1.552901 2.573728 1.588544; 1 1.743015 0.839126 1.278245; 1 1.813252 0.074468 4.540155; 1 1.113489 -1.397771 3.851709; 1 0.524361 -0.771159 5.398101; 1 -1.732555 -1.039929 5.511951; 1 -2.488776 -1.709350 4.061209; 1 -3.276641 -0.397593 4.938518; 1 -3.943324 2.064477 2.034237; 1 -4.267830 0.817021 3.241344; 1 -3.857799 0.356703 1.589439; 1 -0.487686 3.321902 0.769842; 1 -2.244821 3.150157 0.889758; 1 -1.304912 1.992619 -0.060035; 6 2.113645 5.755719 1.272256; 6 0.890497 5.370501 1.845500; 1 -0.028523 5.541496 1.286886; 6 0.811027 4.802073 3.124953; 6 2.017134 4.642340 3.836298; 1 1.983085 4.195947 4.826999; 6 3.247835 5.020138 3.300293; 6 3.284047 5.570588 2.008233; 1 4.240371 5.861942 1.576373; 6 2.149007 6.376692 -0.106754; 1 1.653985 7.356722 -0.117177; 1 1.631884 5.750859 -0.844834; 1 3.177304 6.522441 -0.453693; 6 -0.522453 4.464591 3.736923; 6 4.527352 4.844937 4.088275; 1 5.004666 5.811079 4.298473; 1 5.259817 4.241356 3.537242; 1 4.341078 4.350851 5.047425; 1 -1.322653 4.721585 3.030962; 1 -0.660248 5.092328 4.616510; 1 0.337100 2.798549 5.264555; 77 -2.039026 4.041661 8.696109; 6 -4.019045 3.947908 9.730870; 6 -3.605783 5.334947 9.661187; 6 -3.053651 3.268639 10.562413; 6 -5.302599 3.372415 9.203620; 6 -2.452370 5.514433 10.510331; 6 -4.368728 6.435656 8.979148; 6 -2.105045 4.240624 11.056953; 6 -3.140939 1.834413 10.999826; 1 -5.629869 3.886814 8.295156; 1 -6.113030 3.460612 9.943178; 1 -5.196018 2.311692 8.956840; 6 -1.760689 6.817454 10.790739; 1 -3.707962 7.248899 8.663201; 1 -5.123846 6.866318 9.651942; 1 -4.888434 6.068591 8.089460; 6 -0.994865 3.967040 12.031125; 1 -3.608437 1.210156 10.232571; 1 -3.742585 1.741072 11.915078; 1 -2.153511 1.413627 11.211157; 1 -0.695044 6.672918 10.996002; 1 -2.197376 7.321249 11.665060; 1 -1.842553 7.506794 9.944718; 1 -0.617985 2.944319 11.934751; 1 -1.335533 4.092513 13.069160; 1 -0.149377 4.646084 11.881286; 6 -0.621605 4.695555 7.211706; 6 -1.923519 4.601199 6.579759; 6 -0.193784 3.449993 7.772161; 6 -2.329516 3.418980 5.736884; 1 -2.374387 5.553728 6.299747; 6 -1.105214 2.343407 7.599605; 1 0.729333 3.369925 8.341463; 6 -1.842677 2.204943 6.294452; 1 -2.461376 1.310260 6.232029; 6 -3.715045 3.542655 5.139082; 1 -3.756165 4.320094 4.365741; 1 -4.048409 2.603538 4.690106; 1 -4.437137 3.819167 5.923212; 6 -0.780052 1.044337 8.305107; 1 -0.269516 1.219181 9.258249; 1 -1.685151 0.461216 8.512592; 1 -0.123386 0.419374 7.680669; 6 0.195434 5.957177 7.290230; 1 0.841773 6.051930 6.407350; 1 -0.450268 6.841749 7.323728; 1 0.836114 5.965337 8.179477;
8-9_ts	6 7.001659 6.955236 1.312182; 6 6.809444 7.599607 0.034286; 6 5.428586 8.015224 -0.069667; 6 4.771029 7.671499 1.150066; 6 5.744093 7.056059 2.022233; 6 8.207471 6.167199 1.738456; 1 9.130682 6.594987 1.335741; 1 8.146210 5.124709 1.390122; 1 8.308264 6.143760 2.827576; 6 7.817711 7.639316 -1.078420; 1 7.676845 8.511578 -1.723811; 1 7.738377 6.743815 -1.711217; 1 8.839667 7.678899 -0.690008; 6 4.788684 8.638508 -1.277364; 1 3.963682 9.302832 -1.000331; 1 4.379386 7.872840 -1.952483; 1 5.507710 9.228153 -1.854222; 6 3.316978 7.878903 1.462186; 1 3.151580 8.025760 2.534094; 1 2.714272 7.011509 1.156165; 1 2.913471 8.754604 0.943335; 6 5.448051 6.436783 3.359461; 1 6.335547 6.424558 3.998557; 1 5.104871 5.398504 3.245595; 1 4.665234 6.984712 3.893327; 6 8.096241 10.739771 1.422710; 6 6.761752 11.254783 1.608513; 1 6.241313 11.829082 0.845223; 6 6.110841 10.872233 2.829969; 6 6.946783 10.039278 3.671599; 1 6.434972 9.581593 4.519808; 6 8.439687 10.220297 3.836404; 6 9.038674 10.638102 2.591834; 1 9.993830 10.190514 2.309952; 6 8.752940 10.966768 0.077533; 1 9.476468 10.175966 -0.153132; 1 9.301054 11.920866 0.074991; 1 8.018274 10.995194 -0.734703; 6 4.695547 11.278862 3.158189; 1 4.088811 11.363057 2.249431; 1 4.661496 12.251634 3.667746; 1 4.221834 10.542429 3.816881; 6 9.055743 9.118904 4.679671; 1 10.147751 9.138747 4.634075; 1 8.724399 8.132352 4.319881; 1 8.762509 9.202131 5.734296; 77 6.576539 9.122589 1.691133; 1 11.744985 12.438273 1.349780; 6 11.852736 13.328812 1.976154; 6 11.428703 13.064501 3.392499; 1 11.255824 14.124604 1.521075; 1 12.907957 13.637775 1.930673; 6 10.855327 13.995455 4.302875; 6 11.638940 11.823009 4.127938; 6 10.822375 13.380655 5.627832; 6 10.526574 15.434035 4.017644; 6 11.338628 12.070731 5.531572; 6 12.439561 10.657647 3.615728; 77 9.435083 12.164260 4.026648; 6 10.370398 14.079157 6.877398; 1 10.335823 15.595296 2.952861; 1 11.357290 16.092773 4.310312; 1 9.636556 15.764560 4.560784; 6 11.590911 11.113259 6.659649; 1 12.248146 10.466367 2.555374; 1 12.220274 9.736140 4.163172; 1 13.516321 10.852451 3.722208; 6 6.971267 14.521560 4.535944; 6 6.807555 15.392047 5.619782; 1 11.168292 14.714043 7.287975; 1 10.083517 13.368827 7.659522; 1 9.512193 14.731762 6.685268; 1 11.365244 10.081163 6.375766; 1 10.982348 11.352885 7.537098; 1 12.644584 11.141597 6.974195; 6 6.655849 14.992502 3.251100; 6 7.421659 13.096216 4.771113; 1 7.041079 15.040257 6.623299; 6 6.336698 16.702302 5.446574; 6 6.183618 16.290399 3.044384; 1 6.797389 14.334018 2.396417; 1 6.596646 12.393664 4.670314; 1 7.799249 12.993001 5.790162; 1 8.009025 12.881309 3.549364; 6 6.146256 17.612993 6.638717; 6 6.033447 17.135634 4.154066; 6 5.831407 16.778626 1.657183; 1 6.980745 17.531552 7.344872; 1 5.231694 17.359851 7.191635; 1 6.064162 18.661143 6.332902; 1 5.676957 18.153454 4.003719; 1 6.206877 16.098049 0.886286; 1 6.250777 17.772950 1.462973; 1 4.743945 16.858070 1.526499;

8-12_ts	77 -1.056355 2.447298 4.061009; 6 -2.062161 0.467655 3.124642; 6 -0.631808 0.404902 2.960730; 6 -0.207206 1.496371 2.141655; 6 -1.387475 2.298551 1.853463; 6 -2.528817 1.631192 2.428919; 6 -3.959943 2.019947 2.195760; 6 -1.444820 3.441309 0.881196; 6 1.148630 1.654308 1.519159; 6 0.245594 -0.704731 3.467745; 6 -2.901395 -0.598354 3.771736; 1 1.942973 1.294091 2.178968; 1 1.373024 2.697516 1.289132; 1 1.207276 1.082041 0.581149; 1 1.277527 -0.368972 3.601473; 1 0.259400 -1.546259 2.760421; 1 -0.108008 -1.092830 4.428305; 1 -2.473213 -0.939579 4.721291; 1 -2.986750 -1.481943 3.122811; 1 -3.918289 -0.250193 3.973706; 1 -4.075678 3.105633 2.127790; 1 -4.618823 1.665103 2.992185; 1 -4.324051 1.590319 1.251353; 1 -0.521022 4.025069 0.900068; 1 -2.272582 4.120863 1.106698; 1 -1.585238 3.074580 -0.145800; 6 2.952944 5.301140 1.846637; 6 1.640119 5.188875 2.333619; 1 0.844847 5.745121 1.838606; 6 1.322966 4.404651 3.451910; 6 2.384782 3.734162 4.092083; 1 2.161532 3.116195 4.959365; 6 3.700264 3.829893 3.640957; 6 3.971891 4.613992 2.506571; 1 4.994710 4.690832 2.140581; 6 3.248543 6.162731 0.638674; 1 2.998879 7.215214 0.826310; 1 2.663313 5.847879 -0.234782; 1 4.307606 6.117184 0.364050; 6 -0.078316 4.347801 3.992296; 6 4.824589 3.114055 4.356994; 1 5.522331 3.824531 4.819974; 1 5.412336 2.494947 3.667430; 1 4.442459 2.462202 5.149618; 1 -0.721557 5.019342 3.409725; 1 -0.051177 4.734400 5.006498; 1 -0.130701 2.192126 5.442544; 77 -2.075324 4.246731 8.550145; 6 -3.689608 3.454791 9.895776; 6 -3.983679 4.835572 9.585696; 6 -2.445544 3.449191 10.633778; 6 -4.594702 2.275713 9.678789; 6 -2.972795 5.669735 10.195891; 6 -5.231432 5.331900 8.910849; 6 -2.026383 4.814725 10.842509; 6 -1.812833 2.236665 11.254891; 1 -5.235368 2.415174 8.803306; 1 -5.252810 2.116099 10.546379; 1 -4.026090 1.353633 9.523823; 6 -2.956752 7.171274 10.198125; 1 -5.055170 6.270447 8.376433; 1 -6.028062 5.515167 9.645790; 1 -5.609048 4.605583 8.185805; 6 -0.837656 5.256242 11.647501; 1 -1.995114 1.339440 10.656058; 1 -2.222226 2.053287 12.258379; 1 -0.729548 2.350614 11.357468; 1 -1.936200 7.564167 10.247737; 1 -3.505761 7.573981 11.061391; 1 -3.423868 7.580199 9.296979; 1 -0.035140 4.512918 11.620919; 1 -1.106803 5.408419 12.702721; 1 -0.426185 6.200217 11.276424; 6 -1.213796 5.227054 6.837513; 6 -2.373782 4.514960 6.360079; 6 -0.246716 4.367141 7.456441; 6 -2.335639 3.151018 5.714553; 1 -3.210993 5.145104 6.057496; 6 -0.603139 2.964975 7.517663; 1 0.665205 4.760052 7.898752; 6 -1.317649 2.324089 6.348673; 1 -1.575221 1.281123 6.537207; 6 -3.741750 2.575950 5.616638; 1 -4.347248 3.122973 4.884626; 1 -3.730933 1.522299 5.328456; 1 -4.246490 2.655570 6.590804; 6 0.316488 2.040132 8.287671; 1 0.767798 2.548949 9.145794; 1 -0.220178 1.160995 8.663278; 1 1.132834 1.673539 7.645837; 6 -1.029152 6.716681 6.718523; 1 -0.559544 6.968238 5.758679; 1 -1.993868 7.234089 6.761261; 1 -0.392458 7.108045 7.519865;
12	77 -1.592528 2.948754 3.856622; 6 -3.182969 1.949644 2.595914; 6 -1.860293 1.981560 1.938200; 6 -1.584077 3.339204 1.542004; 6 -2.579714 4.141560 2.172182; 6 -3.623994 3.284580 2.732269; 6 -4.945329 3.775148 3.247060; 6 -2.660286 5.638203 2.118616; 6 -0.489632 3.811647 0.635243; 6 -1.115105 0.766511 1.471988; 6 -3.940667 0.691970 2.895994; 1 0.388686 3.164751 0.684225; 1 -0.156245 4.820821 0.889937; 1 -0.839361 3.828088 -0.407563; 1 -0.049243 0.979507 1.355913; 1 -1.500690 0.426790 0.499806; 1 -1.219164 -0.060602 2.180403; 1 -3.289098 -0.074765 3.325828; 1 -4.369602 0.280283 1.971272; 1 -4.759671 0.863212 3.597970; 1 -4.829469 4.623770 3.929987; 1 -5.487887 2.991870 3.782696; 1 -5.585627 4.110583 2.418802; 1 -1.670051 6.086964 2.003860; 1 -3.114294 6.046108 3.026700; 1 -3.275785 5.961321 1.267025; 6 2.697494 5.163398 2.225053; 6 1.736719 4.727197 3.140063; 1 1.181360 5.464028 3.718730; 6 1.465728 3.361581 3.345563; 6 2.211301 2.436686 2.596738; 1 2.041380 1.372619 2.757592; 6 3.180587 2.839260 1.667571; 6 3.407843 4.206737 1.484658; 1 4.155966 4.534023 0.764153; 6 2.990635 6.637313 2.048965; 1 3.923932 6.922168 2.553322; 1 2.191466 7.258563 2.467332; 1 3.105779 6.901942 0.991020; 6 0.460848 2.911461 4.361444; 6 3.992322 1.812924 0.908038; 1 4.864074 1.484129 1.490194; 1 4.368516 2.218042 -0.037786; 1 3.401725 0.917822 0.681817; 1 0.568999 3.517176 5.269160; 1 0.682954 1.876779 4.649911; 1 -0.157608 1.597954 6.409145; 77 -2.190627 4.410949 8.483622; 6 -3.859162 3.841346 9.877629; 6 -4.236897 5.055970 9.184962; 6 -2.700640 4.156115 10.679008; 6 -4.641548 2.559750 9.917919; 6 -3.356453 6.116140 9.606178; 6 -5.450600 5.219115 8.313898; 6 -2.401712 5.559601 10.518953; 6 -2.044435 3.228437 11.660910; 1 -5.190345 2.394116 8.986355; 1 -5.376406 2.568547 10.737193; 1 -3.989258 1.694428 10.070383; 6 -3.455129 7.559127 9.201569; 1 -5.306723 6.004518 7.565109; 1 -6.331695 5.492164 8.912057; 1 -5.685696 4.293444 7.780865; 6 -1.335021 6.319814 11.253941; 1 -2.098064 2.187823 11.327561; 1 -2.536686 3.286977 12.642176; 1 -0.988218 3.472556 11.807811; 1 -2.473321 8.043430 9.190254; 1 -4.092061 8.126064 9.895837; 1 -3.887507 7.668703 8.201993; 1 -0.478722 5.682202 11.493317; 1 -1.717700 6.723090 12.202917; 1 -0.963470 7.165460 10.666523; 6 -1.242002 4.981837 6.650868; 6 -2.307490 4.065198 6.292551; 6 -0.273802 4.386270 7.529443; 6 -2.076568 2.604146 5.919971; 1 -3.180524 4.529159 5.832358; 6 -0.580892 3.035505 7.952297; 1 0.558976 4.962584 7.926534; 6 -1.040042 2.016161 6.907873; 1 -1.496784 1.173567 7.446466; 6 -3.388628 1.821797 6.071776; 1 -4.209486 2.274262 5.506218; 1 -3.272329 0.785974 5.732422; 1 -3.690857 1.795745 7.129028; 6 0.287478 2.438045 9.041751; 1 0.626692 3.197252 9.755308; 1 -0.253267 1.666293 9.602097; 1 1.179222 1.957345 8.610378; 6 -1.135357 6.395026 6.145570; 1 -0.628831 6.419886 5.170589; 1 -2.128438 6.839265 6.012182; 1 -0.567988 7.028069 6.837546;
12-13_ts	77 -1.773499 4.308289 3.500316; 6 -4.073679 4.451044 2.791850; 6 -3.299699 3.844828 1.737764; 6 -2.291983 4.766469 1.317823; 6 -2.417013 5.959906 2.147682; 6 -3.543131 5.764286 3.021342; 6 -4.133260 6.817199 3.910936; 6 -1.694067 7.257486 1.922167; 6 -1.391288 4.627089 0.124722; 6 -3.571673 2.496656 1.132069; 6 -5.306613 3.860576 3.416175; 1 -1.215459 3.577591 -0.130533; 1 -0.415208 5.092403 0.293406; 1 -1.832832 5.111980 -0.757970; 1 -2.673301 2.070180 0.677239; 1 -4.338411 2.567555 0.347657; 1 -3.935255 1.784594 1.879465; 1 -5.220204 2.775114 3.533967; 1 -6.198708 4.048835 2.801164; 1 -5.501189 4.283959 4.406001; 1 -3.359790 7.403242 4.415722; 1 -4.774683 6.380695 4.678268; 1 -4.748387 7.513684 3.323168; 1 -0.645926 7.095486 1.652841; 1 -1.708690 7.883283 2.819478; 1 -2.158865 7.831266 1.107308; 6 2.158659 1.415073 2.546352; 6 1.254715 2.474836 2.490083; 1 0.488491 2.480231 1.717513; 6 1.296115 3.544199 3.407731; 6 2.294083 3.509672 4.390535; 1 2.368952 4.336157 5.094861; 6 3.215201 2.453168 4.482391; 6 3.134989 1.412772 3.557038; 1 3.843609 0.587869 3.615019; 6 2.108613 0.292192 1.533828; 1 2.980266 0.314868 0.866116; 1 1.212252 0.357517 0.908095; 1 2.106511 -0.690530 2.021620; 6 0.328757 4.693916 3.309209; 6 4.268794 2.452232 5.567855; 1 4.858695 3.377345 5.555146; 1 4.961941 1.612376 5.452515; 1 3.818085 2.375808 6.566234; 1 0.408813 5.118952 2.301310; 1 0.626731 5.484023 4.005201; 1 -0.189436 5.925268 5.898785; 77 -2.458257 4.035879 8.581243; 6 -2.137372 1.931901 9.285784; 6 -3.557349 2.132720 9.096563; 6 -1.725248 2.821017 10.349161; 6 -1.291434 0.874405 8.635690; 6 -4.019388 3.088337 10.073356; 6 -4.432758 1.345386 8.162348; 6 -2.889524 3.515271 10.842481; 6 -0.356165 2.860933 10.965511; 1 -1.638546 0.647475 7.623248; 1 -1.313123 -0.063871 9.209974; 1 -0.246385 1.189004 8.557708; 6 -5.444392 3.512380 10.285616; 1 -5.297182 1.928897 7.830127; 1 -4.815632 0.438582 8.651604; 1 -3.883553 1.031050 7.269917; 6 -2.922551 4.456966 12.012290; 1 0.420890 2.634060 10.229793; 1 -0.270863 2.122099 11.774932; 1 -0.129529 3.843097 11.390921; 1 -5.508701 4.537558 10.664656; 1 -5.950133 2.862317 11.014142; 1 -6.020846 3.467921 9.356294; 1 -1.969161 4.979257 12.136033; 1 -3.123816 3.918443 12.949821; 1 -3.703117 5.216096 11.898796; 6 -

	3.168571 5.616149 7.303605; 6 -2.875985 4.460173 6.473981; 6 -2.035006 6.063875 8.064426; 6 -1.606113 4.275773 5.691185; 1 -3.747295 3.950904 6.062425; 6 -0.827241 5.295144 7.852133; 1 -2.124038 6.862729 8.797393; 6 -0.453527 4.982559 6.400540; 1 0.446794 4.359201 6.378893; 6 -1.397878 2.913485 5.192722; 1 -2.192265 2.191592 5.382211; 1 -1.263260 2.748038 3.759978; 1 -0.400009 2.494133 5.328020; 6 0.360615 5.593888 8.743167; 1 0.048546 5.886677 9.751886; 1 1.019631 4.722563 8.833727; 1 0.966836 6.413366 8.325848; 6 -4.531281 6.240185 7.464899; 1 -4.652922 7.109380 6.806041; 1 -5.324665 5.524318 7.221512; 1 -4.687539 6.581561 8.494586;
13	77 0.563180 2.308577 1.238754; 6 0.518226 3.373724 -1.719423; 6 0.960561 4.599043 -2.236930; 6 2.621977 1.668756 2.062710; 6 1.937822 2.178956 3.205191; 6 1.663690 3.583377 2.975963; 6 2.199873 3.943665 1.708868; 6 2.757814 2.751536 1.099939; 6 3.617024 2.721129 -0.133160; 6 2.264145 5.327540 1.133085; 6 1.033052 4.510781 3.974674; 6 1.709554 1.462074 4.505344; 6 3.231121 0.300801 1.929178; 1 0.713962 5.515858 -1.703470; 6 1.685742 4.685943 -3.437110; 6 2.138055 6.031338 -3.960553; 6 1.962408 3.511412 -4.136313; 1 2.523365 3.562130 -5.068365; 6 1.520678 2.264131 -3.662341; 6 1.814666 1.005145 -4.447945; 6 0.810260 2.214008 -2.464296; 1 0.475817 1.251565 -2.083879; 6 -0.291754 3.315837 -0.453668; 1 0.221092 4.023603 4.525304; 1 0.618958 5.401300 3.493171; 1 1.768567 4.849275 4.718359; 1 0.765165 1.754230 4.975065; 1 2.512740 1.693072 5.219990; 1 1.693266 0.376626 4.375707; 1 2.654837 -0.454352 2.472016; 1 4.254926 0.288421 2.329657; 1 3.281527 -0.011995 0.882968; 1 3.256087 3.417446 -0.893821; 1 3.627253 1.726297 -0.586929; 1 4.655836 2.989659 0.108405; 1 1.427541 5.948230 1.469655; 1 2.247016 5.305261 0.040965; 1 3.191895 5.831119 1.439794; 1 -1.246264 2.828130 -0.672607; 1 -0.522626 4.337110 -0.124918; 1 0.519102 1.125121 0.179767; 1 1.435996 0.115987 -3.933327; 1 2.892926 0.868876 -4.601248; 1 1.352935 1.035926 -5.443496; 1 2.760076 6.561433 -3.228034; 1 1.282914 6.682840 -4.182892; 1 2.722911 5.926624 -4.880309; 6 -1.987829 -1.102532 2.694800; 6 -1.114251 0.132052 2.921420; 1 -0.066220 -0.185374 3.000414; 6 -1.319813 1.134616 1.787324; 6 -2.082169 0.522006 0.663152; 1 -2.337087 1.198932 -0.148709; 6 -1.923571 -0.869343 0.279199; 6 -1.879710 -1.755137 1.405523; 1 -1.864197 -2.835221 1.276862; 6 -2.133012 -1.983432 3.918652; 1 -2.641472 -1.453703 4.732820; 1 -1.146887 -2.291445 4.301138; 1 -2.705520 -2.891381 3.698717; 6 -1.430168 2.532003 2.034520; 6 -1.848832 -1.342666 -1.146430; 1 -2.226535 -2.366145 -1.253092; 1 -0.808018 -1.328626 -1.498743; 1 -2.426847 -0.690109 -1.809631; 1 -1.374796 2.884394 3.064441; 1 -2.076759 3.135395 1.399531; 1 -5.155488 -0.697900 4.761855; 6 -5.692401 -1.393678 4.110396; 6 -5.609700 -0.973904 2.670853; 1 -5.267575 -2.389176 4.269293; 1 -6.737962 -1.422585 4.448209; 6 -5.551852 0.390276 2.198795; 6 -5.748672 -1.846982 1.531548; 6 -5.617863 0.340510 0.751806; 6 -5.599926 1.629794 3.046092; 6 -5.763624 -1.034856 0.349086; 6 -5.939298 -3.336145 1.580284; 77 -3.723337 -0.656225 1.450498; 6 -5.692131 1.536421 -0.155373; 1 -5.120461 1.469541 4.016511; 1 -6.637169 1.943489 3.236403; 1 -5.085204 2.465708 2.563103; 6 -5.957729 -1.528045 -1.056037; 1 -5.516799 -3.825968 0.697212; 1 -7.006294 -3.599171 1.621973; 1 -5.461401 -3.774357 2.461587; 1 -5.119142 2.377331 0.245982; 1 -6.731468 1.871794 -0.279417; 1 -5.297605 1.314250 -1.151687; 1 -5.486197 -0.862474 -1.785346; 1 -7.025318 -1.588865 -1.311888; 1 -5.531203 -2.526558 -1.195865; 1 -1.377262 0.596621 3.881153;
13-14_ts	77 0.466031 2.449730 1.413536; 6 0.332692 3.485600 -1.851100; 6 0.770060 4.743589 -2.277757; 6 2.435526 1.562678 2.170701; 6 1.758842 2.320436 3.214699; 6 1.835505 3.735842 2.867825; 6 2.431314 3.831921 1.592064; 6 2.770366 2.486412 1.136803; 6 3.538119 2.181996 -0.119538; 6 2.741295 5.090753 0.836415; 6 1.392668 4.864347 3.753479; 6 1.444437 1.822447 4.599101; 6 2.816993 0.111632 2.232308; 1 0.556022 5.616053 -1.662967; 6 1.462658 4.910542 -3.487940; 6 1.911155 6.286005 -3.928295; 6 1.714532 3.784028 -4.272102; 1 2.252713 3.898740 -5.211637; 6 1.288341 2.505408 -3.876765; 6 1.569466 1.303311 -4.749970; 6 0.604182 2.374129 -2.668174; 1 0.277143 1.387175 -2.345709; 6 -0.454468 3.339577 -0.574374; 1 0.475680 4.613470 4.296126; 1 1.197487 5.776354 3.181165; 1 2.159803 5.104558 4.504352; 1 0.568286 2.323697 5.022506; 1 2.290455 2.013345 5.274982; 1 1.250098 0.747834 4.614057; 1 2.077777 -0.481231 2.778769; 1 3.783305 -0.019910 2.741704; 1 2.909147 -0.321090 1.231881; 1 3.191741 2.786900 -0.962527; 1 3.433019 1.131642 -0.406108; 1 4.610764 2.383875 0.015302; 1 2.127492 5.930030 1.178770; 1 2.572794 4.968335 -0.237901; 1 3.794079 5.378145 0.970169; 1 -1.470017 2.985003 -0.773813; 1 -0.544801 4.299947 -0.066515; 1 0.011816 2.096674 -0.143896; 1 1.209233 0.379483 -4.286361; 1 2.644228 1.186785 -4.937530; 1 1.082318 1.397338 -5.728974; 1 2.521915 6.775064 -3.159325; 1 1.053455 6.943253 -4.121242; 1 2.504999 6.237929 -4.846669; 6 -1.947051 -1.094177 2.720202; 6 -1.111211 0.157906 2.991225; 1 -0.064869 -0.152006 3.110274; 6 -1.280962 1.179496 1.864654; 6 -2.023834 0.559178 0.724255; 1 -2.297645 1.238814 -0.080172; 6 -1.824868 -0.819345 0.313432; 6 -1.786017 -1.725016 1.425385; 1 -1.735665 -2.802039 1.280171; 6 -2.103021 -1.998805 3.925785; 1 -2.649472 -1.496423 4.732683; 1 -1.119996 -2.285006 4.332796; 1 -2.642876 -2.919072 3.675787; 6 -1.502152 2.578530 2.164514; 6 -1.703273 -1.265740 -1.118107; 1 -2.048126 -2.297737 -1.252067; 1 -0.655315 -1.217185 -1.447176; 1 -2.286964 -0.619972 -1.783694; 1 -1.540962 2.882946 3.211852; 1 -2.200670 3.139726 1.539102; 1 -5.158490 -0.792770 4.710460; 6 -5.674110 -1.488866 4.042316; 6 -5.568796 -1.053830 2.608700; 1 -5.238941 -2.480187 4.199771; 1 -6.725384 -1.534963 4.360238; 6 -5.528812 0.315916 2.151318; 6 -5.665582 -1.918392 1.458979; 6 -5.559474 0.278545 0.703140; 6 -5.624051 1.545106 3.009799; 6 -5.668292 -1.095935 0.284676; 6 -5.826487 -3.411614 1.490340; 77 -3.657666 -0.680847 1.437267; 6 -5.639750 1.480941 -0.195000; 1 -5.161030 1.385759 3.988283; 1 -6.672244 1.832787 3.181436; 1 -5.117814 2.397459 2.547171; 6 -5.819038 -1.579646 -1.129125; 1 -5.378763 -3.884400 0.610298; 1 -6.888288 -3.697773 1.511168; 1 -5.354632 -3.848678 2.375561; 1 -5.098552 2.331467 0.229807; 1 -6.683122 1.792790 -0.345056; 1 -5.213157 1.277614 -1.182360; 1 -5.344973 -0.897029 -1.841034; 1 -6.878555 -1.661619 -1.411832; 1 -5.367041 -2.567155 -1.267799; 1 -1.416652 0.601196 3.948471;
14	77 0.305034 2.503580 1.515414; 6 2.424639 1.670174 1.525212; 6 2.120495 2.665099 2.566915; 6 2.030519 3.991421 1.930028; 6 2.051468 3.769733 0.543659; 6 2.278027 2.336276 0.291363; 6 2.432364 1.739120 -1.077032; 6 1.914212 4.800160 -0.538085; 6 1.934347 5.301630 2.653533; 6 2.276518 2.436241 4.041513; 6 2.831133 0.245944 1.761726; 1 1.339564 5.212376 3.567612; 1 1.465867 6.070728 2.032058; 1 2.929839 5.669900 2.943096; 1 1.676335 3.145249 4.619115; 1 3.327021 2.560494 4.342898; 1 1.964466 1.426032 4.322278; 1 2.323537 -0.177641 2.633800; 1 3.913920 0.166703 1.940330; 1 2.590505 -0.388378 0.903310; 1 1.695979 2.146364 -1.777851; 1 2.305343 0.652846 -1.056960; 1 3.428323 1.950897 -1.492503; 1 1.467280 5.723764 -0.159518; 1 1.284510 4.438843 -1.358053; 1 2.893266 5.055099 -0.969170; 6 -1.942939 -1.034501 2.678744; 6 -1.236053 0.239478 3.144961; 1 -0.188854 0.002104 3.392212; 6 -1.342625 1.333818 2.087309; 6 -2.047599 0.830620 0.860684; 1 -2.329809 1.592873 0.132073; 6 -1.779466 -0.485346 0.311273; 6 -1.704223 -1.497027 1.326226; 1 -1.587585 -2.547429 1.067617; 6 -2.075408 -2.088764 3.759562; 1 -2.679112 -1.724446 4.598924; 1 -1.088426 -2.359033 4.167331; 1 -2.543594 -3.003756 3.379922; 6 -1.544051 2.739909 2.436916; 6 -1.602977 -0.772324 -1.154659; 1 -1.882573 -1.803194 -1.401604; 1 -0.554860 -0.625554 -1.451777; 1 -2.213510 -0.096111 -1.763500; 1 -1.548776 3.034798 3.486972; 1 -2.255548

	3.321194 1.839501; 1 -5.221250 -1.256066 4.554158; 6 -5.675662 -1.870687 3.771491; 6 -5.554892 -1.221245 2.422835; 1 -5.187938 -2.849641 3.796741; 1 -6.731167 -2.020139 4.039755; 6 -5.587117 0.201223 2.173510; 6 -5.565757 -1.908879 1.154835; 6 -5.575071 0.378438 0.735218; 6 -5.779529 1.282020 3.199142; 6 -5.587059 -0.921877 0.114876; 6 -5.637852 -3.396408 0.959380; 77 -3.640663 -0.570933 1.387296; 6 -5.704372 1.694720 0.021488; 1 -5.340031 1.002240 4.161310; 1 -6.847365 1.486366 3.369183; 1 -5.307446 2.218322 2.887694; 6 -5.665993 -1.196218 -1.359550; 1 -5.133455 -3.707654 0.039005; 1 -6.681047 -3.738594 0.892809; 1 -5.171768 -3.935063 1.789882; 1 -5.235150 2.503017 0.589649; 1 -6.760868 1.960733 -0.125208; 1 -5.232674 1.667741 -0.965879; 1 -5.212118 -0.389617 -1.943576; 1 -6.709573 -1.292839 -1.692341; 1 -5.151294 -2.125345 -1.625113; 1 -1.702881 0.582980 4.079317;
14-15_ts	77 0.214568 2.604051 1.832602; 6 2.120109 1.843033 0.611832; 6 2.361186 1.550645 1.978947; 6 2.312940 2.804441 2.720187; 6 2.087583 3.876536 1.802218; 6 1.860557 3.266341 0.501287; 6 1.694779 4.018700 -0.789476; 6 2.167907 5.347872 2.090145; 6 2.641243 2.952419 4.180250; 6 2.701436 0.209341 2.563968; 6 2.168761 0.878057 -0.536321; 1 2.279432 2.100218 4.763783; 1 2.189782 3.855271 4.601466; 1 3.727825 3.019220 4.334768; 1 2.337341 0.116521 3.591906; 1 3.788917 0.044549 2.589535; 1 2.259706 -0.608196 1.985143; 1 2.007943 -0.151691 -0.203142; 1 3.147110 0.909637 -1.038476; 1 1.410152 1.107173 -1.291468; 1 1.146285 4.953039 -0.637938; 1 1.144248 3.430010 -1.530439; 1 2.670504 4.273535 -1.228187; 1 1.907673 5.568933 3.129664; 1 1.485198 5.919580 1.454398; 1 3.184155 5.733360 1.913526; 6 -1.954709 0.208123 2.885651; 6 -1.763414 1.676521 2.650571; 1 -0.630599 2.411332 3.288502; 6 -1.725297 2.039616 1.243465; 6 -2.112474 0.919267 0.340662; 1 -2.357610 1.186710 -0.686519; 6 -1.533012 -0.395534 0.544119; 6 -1.434841 -0.747134 1.930425; 1 -1.081345 -1.729163 2.235249; 6 -2.045013 -0.216137 4.336119; 1 -2.834179 0.331311 4.864379; 1 -1.099825 -0.010028 4.860930; 1 -2.257461 -1.286325 4.431099; 6 -1.572746 3.457597 1.012371; 6 -1.093032 -1.310098 -0.567492; 1 -1.165661 -2.363767 -0.274427; 1 -0.051458 -1.107279 -0.846819; 1 -1.707757 -1.159998 -1.461957; 1 -2.094503 4.163444 1.662162; 1 -1.497518 3.797459 -0.021186; 1 -5.494470 0.299471 4.326203; 6 -5.661734 -0.728517 3.991684; 6 -5.422634 -0.872068 2.515841; 1 -5.002925 -1.378019 4.575634; 1 -6.697498 -0.994481 4.245360; 6 -5.660556 0.152111 1.526749; 6 -5.093058 -2.103980 1.837017; 6 -5.405216 -0.449806 0.233032; 6 -6.230564 1.519912 1.772931; 6 -5.090562 -1.845030 0.430413; 6 -4.870893 -3.436729 2.493251; 77 -3.500009 -0.403723 1.412519; 6 -5.627259 0.219442 -1.094148; 1 -5.939053 1.904987 2.754603; 1 -7.330198 1.506022 1.735381; 1 -5.882595 2.238935 1.025664; 6 -4.850351 -2.854950 -0.654335; 1 -4.182641 -4.062120 1.915780; 1 -5.814205 -3.993248 2.591883; 1 -4.453202 -3.326449 3.498573; 1 -5.421506 1.292188 -1.038774; 1 -6.668040 0.096744 -1.425560; 1 -4.982990 -0.201460 -1.872321; 1 -4.426166 -2.390522 -1.549672; 1 -5.786989 -3.346191 -0.954540; 1 -4.159406 -3.639631 -0.329813; 1 -2.398890 2.293723 3.294157;
15	77 -0.683994 1.281219 3.520580; 6 0.420459 3.093681 2.928048; 6 1.051762 1.990391 2.241653; 6 1.722493 1.182149 3.246866; 6 1.475033 1.752966 4.519647; 6 0.647058 2.934256 4.345316; 6 0.310177 3.929680 5.419330; 6 1.996652 1.264525 5.840542; 6 2.562931 -0.026979 2.951600; 6 1.212964 1.846026 0.754559; 6 -0.208413 4.290446 2.270310; 1 2.142742 -0.617262 2.130732; 1 2.651592 -0.684807 3.821841; 1 3.581690 0.261411 2.655418; 1 1.261066 0.794140 0.455462; 1 2.136229 2.331045 0.402404; 1 0.375541 2.299911 0.217369; 1 -0.673828 4.024635 1.317336; 1 0.548648 5.062343 2.072160; 1 -0.982093 4.733961 2.902935; 1 0.092357 3.435734 6.371602; 1 -0.568677 4.522630 5.151118; 1 1.142238 4.629058 5.593507; 1 2.269825 0.205345 5.805341; 1 1.254923 1.390784 6.636077; 1 2.894022 1.823330 6.142063; 6 -1.950562 -1.278520 1.726097; 6 -1.242208 -0.814479 2.966871; 1 -0.352496 -1.382585 3.237476; 6 -2.130809 -0.356162 3.977445; 6 -3.562560 -0.343017 3.562261; 1 -4.271082 -0.079038 4.346251; 6 -3.935686 0.043857 2.210486; 6 -3.051277 -0.491476 1.223049; 1 -3.247414 -0.363813 0.161067; 6 -1.092971 -2.021550 0.724887; 1 -0.631339 -2.908924 1.173417; 1 -0.277486 -1.377579 0.362468; 1 -1.675433 -2.348200 -0.143079; 6 -1.625511 0.196384 5.188102; 6 -5.107286 0.928271 1.887262; 1 -5.495043 0.732529 0.881169; 1 -4.806114 1.983425 1.936263; 1 -5.921653 0.782362 2.605516; 1 -0.800792 -0.297466 5.701205; 1 -2.332457 0.693448 5.848642; 1 -2.053638 1.889851 2.988184; 1 -1.949529 -5.078730 2.055279; 6 -2.829341 -5.147775 1.408891; 6 -3.967809 -4.326892 1.943025; 1 -2.529633 -4.824572 0.407634; 1 -3.110742 -6.208309 1.343384; 6 -4.241029 -4.082063 3.336906; 6 -5.065552 -3.803746 1.158884; 6 -5.490457 -3.345737 3.395205; 6 -3.478276 -4.630297 4.509026; 6 -6.012279 -3.219187 2.056879; 6 -5.221409 -3.937277 -0.329223; 77 -3.943128 -2.101956 2.345130; 6 -6.210865 -2.960982 4.656791; 1 -2.415554 -4.746896 4.276368; 1 -3.862527 -5.616860 4.809021; 1 -3.550248 -3.969764 5.378130; 6 -7.328201 -2.602526 1.679245; 1 -5.793852 -3.105410 -0.751814; 1 -5.748966 -4.865541 -0.592360; 1 -4.251320 -3.960872 -0.834642; 1 -5.507883 -2.731074 5.462584; 1 -6.858632 -3.778351 5.003931; 1 -6.843729 -2.080403 4.508588; 1 -7.618631 -1.813508 2.379785; 1 -8.133551 -3.351079 1.678778; 1 -7.294038 -2.159454 0.678782;
15-9_ts	77 5.799828 2.077308 5.603787; 77 7.843559 2.369835 1.745132; 6 9.107930 3.179123 0.266383; 6 9.430759 1.743384 0.253435; 6 8.261589 1.066814 -0.166692; 6 7.250136 2.058382 -0.539713; 6 7.778458 3.349609 -0.340672; 6 6.362657 2.638043 7.708168; 6 6.307258 1.197600 7.633288; 6 4.939071 0.798563 7.398961; 6 4.150290 1.984310 7.273803; 6 5.026800 3.123578 7.438230; 6 7.145686 4.662928 -0.696941; 6 10.770573 1.146555 0.569676; 6 10.106638 4.281883 0.468336; 6 5.894325 1.715907 -1.085968; 6 8.086457 -0.417591 -0.312437; 6 2.661152 2.042946 7.086450; 6 4.587005 4.556037 7.540247; 6 4.432308 -0.614125 7.345683; 6 7.534123 3.466579 8.152905; 6 7.448078 0.264548 7.928305; 1 7.462824 4.998984 -1.695531; 1 7.421717 5.446215 0.015205; 1 6.053464 4.596001 -0.703245; 1 11.279364 1.702430 1.363048; 1 11.426976 1.156408 -0.313341; 1 10.680526 0.107786 0.901907; 1 9.618758 5.207529 0.787635; 1 10.648903 4.493739 -0.465087; 1 10.845533 4.014532 1.229840; 1 5.440518 0.879314 -0.543482; 1 5.954622 1.420280 -2.143468; 1 5.207345 2.564471 -1.019282; 1 8.812402 -0.968667 0.292488; 1 8.215969 -0.734582 -1.357679; 1 7.086496 -0.737566 -0.000312; 1 2.138525 2.041425 8.054137; 1 2.291625 1.185601 6.514831; 1 2.358064 2.949889 6.554647; 1 5.380774 5.242153 7.230786; 1 4.315487 4.808932 8.575195; 1 3.714003 4.758352 6.912502; 1 3.549429 -0.702117 6.704316; 1 4.148416 -0.975710 8.344659; 1 5.191540 -1.300283 6.957038; 1 8.484559 2.997667 7.881309; 1 7.533839 3.604638 9.244885; 1 7.519250 4.460599 7.695705; 1 7.350911 -0.678442 7.381281; 1 7.488740 0.020754 8.999588; 1 8.408892 0.708291 7.652719; 6 5.292561 -0.190364 3.318695; 6 5.908181 1.137043 3.673542; 6 5.186784 2.382727 3.600757; 6 7.281620 1.272416 4.169900; 6 5.984799 3.540661 3.928547; 1 4.138510 2.417883 3.314131; 6 8.210018 2.384179 3.760881; 1 7.766329 0.328439 4.419323; 6 7.422703 3.565358 3.440076; 1 7.900437 4.544065 3.496860; 6 5.299446 4.886737 4.000313; 1 5.815326 5.562417 4.692644; 1 5.304420 5.370643 3.011851; 1 4.258100 4.797582 4.328382; 6 9.564155 2.422990 4.424494; 1 10.167959 3.243122 4.019683; 1 9.467232 2.578816 5.509813; 1 10.117820 1.488270 4.266767; 1 4.206814 -0.176234 3.465719; 1 5.711154 -0.996900 3.930838; 1 5.488585 -0.438587 2.266423;
9	77 5.799828 2.077308 5.603787; 77 7.843559 2.369835 1.745132; 6 9.107930 3.179123 0.266383; 6 9.430759

	1.743384 0.253435; 6 8.261589 1.066814 -0.166692; 6 7.250136 2.058382 -0.539713; 6 7.778458 3.349609 -0.340672; 6 6.362657 2.638043 7.708168; 6 6.307258 1.197600 7.633288; 6 4.939071 0.798563 7.398961; 6 4.150290 1.984310 7.273803; 6 5.026800 3.123578 7.438230; 6 7.145686 4.662928 -0.696941; 6 10.770573 1.146555 0.569676; 6 10.106638 4.281883 0.468336; 6 5.894325 1.715907 -1.085968; 6 8.086457 -0.417591 -0.312437; 6 2.661152 2.042946 7.086450; 6 4.587005 4.556037 7.540247; 6 4.432308 -0.614125 7.345683; 6 7.534123 3.466579 8.152905; 6 7.448078 0.264548 7.928305; 1 7.462824 4.998984 -1.695531; 1 7.421717 5.446215 0.015205; 1 6.053464 4.596001 -0.703245; 1 11.279364 1.702430 1.363048; 1 11.426976 1.156408 -0.313341; 1 10.680526 0.107786 0.901907; 1 9.618758 5.207529 0.787635; 1 10.648903 4.493739 -0.465087; 1 10.845533 4.014532 1.229840; 1 5.440518 0.879314 -0.543482; 1 5.954622 1.420280 -2.143468; 1 5.207345 2.564471 -1.019282; 1 8.812402 -0.968667 0.292488; 1 8.215969 -0.734582 -1.357679; 1 7.086496 -0.737566 -0.000312; 1 2.138525 2.041425 8.054137; 1 2.291625 1.185601 6.514831; 1 2.358064 2.949889 6.554647; 1 5.380774 5.242153 7.230786; 1 4.315487 4.808932 8.575195; 1 3.714003 4.758352 6.912502; 1 3.549429 -0.702117 6.704316; 1 4.148416 -0.975710 8.344659; 1 5.191540 -1.300283 6.957038; 1 8.484559 2.997667 7.881309; 1 7.533839 3.604638 9.244885; 1 7.519250 4.460599 7.695705; 1 7.350911 -0.678442 7.381281; 1 7.488740 0.020754 8.999588; 1 8.408892 0.708291 7.652719; 6 5.292561 -0.190364 3.318695; 6 5.908181 1.137043 3.673542; 6 5.186784 2.382727 3.600757; 6 7.281620 1.272416 4.169900; 6 5.984799 3.540661 3.928547; 1 4.138510 2.417883 3.314131; 6 8.210018 2.384179 3.760881; 1 7.766329 0.328439 4.419323; 6 7.422703 3.565358 3.440076; 1 7.900437 4.544065 3.496860; 6 5.299446 4.886737 4.000313; 1 5.815326 5.562417 4.692644; 1 5.304420 5.370643 3.011851; 1 4.258100 4.797582 4.328382; 6 9.564155 2.422990 4.424494; 1 10.167959 3.243122 4.019683; 1 9.467232 2.578816 5.509813; 1 10.117820 1.488270 4.266767; 1 4.206814 -0.176234 3.465719; 1 5.711154 -0.996900 3.930838; 1 5.488585 -0.438587 2.266423;
9-10_ts	77 5.865700 2.315267 5.420233; 77 7.636788 2.329497 2.005281; 6 9.020937 2.881927 0.304188; 6 9.300468 1.494501 0.566884; 6 8.101448 0.749745 0.321686; 6 7.093544 1.669404 -0.171593; 6 7.653507 2.981566 -0.181037; 6 6.332789 2.489437 7.629638; 6 6.307989 1.088273 7.292529; 6 4.956205 0.717396 6.942636; 6 4.152620 1.896971 6.997645; 6 4.998558 2.999082 7.394484; 6 7.007704 4.218898 -0.736204; 6 10.629995 0.924588 0.972141; 6 10.021455 4.003633 0.308773; 6 5.750699 1.292320 -0.729774; 6 7.963059 -0.745357 0.386309; 6 2.670490 1.968609 6.760743; 6 4.530126 4.385468 7.734491; 6 4.476969 -0.670073 6.627087; 6 7.472229 3.241589 8.256043; 6 7.459942 0.135150 7.443973; 1 7.234280 4.336886 -1.806434; 1 7.359767 5.120027 -0.225491; 1 5.919214 4.185313 -0.630606; 1 11.209214 1.635913 1.568996; 1 11.237028 0.667438 0.091894; 1 10.514830 0.012285 1.565968; 1 9.549756 4.961393 0.550397; 1 10.500971 4.113104 -0.674768; 1 10.816320 3.833072 1.041724; 1 5.391656 0.342055 -0.324465; 1 5.802661 1.175099 -1.822045; 1 4.992553 2.053168 -0.518146; 1 8.636320 -1.178940 1.132497; 1 8.201291 -1.211672 -0.580921; 1 6.944713 -1.046902 0.649587; 1 2.108984 1.776936 7.686617; 1 2.342667 1.229528 6.022480; 1 2.367916 2.954979 6.396161; 1 5.311813 5.129061 7.552778; 1 4.247127 4.454623 8.794663; 1 3.656697 4.675657 7.142667; 1 3.628455 -0.657642 5.934880; 1 4.148858 -1.197838 7.534676; 1 5.265586 -1.275198 6.168735; 1 8.440821 2.841179 7.940726; 1 7.435389 3.184771 9.354730; 1 7.452081 4.300931 7.981932; 1 7.399139 -0.690221 6.726947; 1 7.479422 -0.306120 8.450274; 1 8.418947 0.638615 7.287335; 6 5.941982 1.784955 3.164810; 6 5.151575 2.930855 3.561466; 6 7.521933 1.836910 4.247931; 6 6.013414 4.000647 4.011654; 6 8.327189 2.975360 3.854845; 6 7.407120 3.989064 3.376984; 1 7.802930 4.956615 3.065745; 6 5.281761 0.454300 2.883933; 1 5.030362 0.373872 1.819882; 1 5.951236 -0.378296 3.123795; 1 4.359821 0.328341 3.464952; 1 4.072223 2.981783 3.431462; 1 8.005753 0.879397 4.437083; 6 5.451997 5.359161 4.360241; 1 6.072857 5.875335 5.102654; 1 5.410424 6.004571 3.468584; 1 4.436930 5.284675 4.765064; 6 9.786013 3.133478 4.183286; 1 10.261403 3.874791 3.530789; 1 9.911793 3.476286 5.220644; 1 10.325943 2.185209 4.076291;
10	77 -0.769648 0.123399 1.487048; 77 0.703854 0.208960 -1.559673; 6 2.493735 0.375797 -3.153556; 6 2.454154 -0.957074 -2.616411; 6 1.187567 -1.540388 -2.947051; 6 0.425751 -0.544040 -3.694609; 6 1.243752 0.626639 -3.815295; 6 -0.125520 0.114935 3.653619; 6 -0.152978 -1.257422 3.155057; 6 -1.522319 -1.602005 2.906657; 6 -2.338155 -0.458045 3.215882; 6 -1.478170 0.586916 3.701185; 6 0.891215 1.865658 -4.588492; 6 3.590787 -1.662602 -1.932166; 6 3.695556 1.275963 -3.179925; 6 -0.855758 -0.780600 -4.443988; 6 0.851926 -2.999672 -2.817108; 6 -3.839761 -0.408864 3.162934; 6 -1.926356 1.915391 4.239096; 6 -2.025377 -2.954410 2.491642; 6 1.082035 0.814355 4.210677; 6 1.018313 -2.197828 3.129101; 1 1.228916 1.789875 -5.632309; 1 1.357844 2.758021 -4.159326; 1 -0.189632 2.032801 -4.603452; 1 4.211780 -0.968102 -1.357805; 1 4.245499 -2.159286 -2.663065; 1 3.226978 -2.428648 -1.241390; 1 3.415544 2.333308 -3.219353; 1 4.314219 1.072767 -4.066008; 1 4.333399 1.135053 -2.302385; 1 -1.449480 -1.580269 -3.994471; 1 -0.651535 -1.074207 -5.484123; 1 -1.481035 0.117324 -4.471413; 1 1.234207 -3.422326 -1.882463; 1 1.295925 -3.572931 -3.644288; 1 -0.225406 -3.176067 -2.841956; 1 -4.288082 -0.751461 4.106879; 1 -4.236887 -1.046302 2.366355; 1 -4.204162 0.607643 2.982742; 1 -1.160409 2.684486 4.101257; 1 -2.140545 1.852599 5.315619; 1 -2.838191 2.266738 3.746390; 1 -2.958200 -2.884510 1.923786; 1 -2.223607 -3.585907 3.369637; 1 -1.298462 -3.480316 1.865638; 1 1.987116 0.558573 3.651223; 1 1.254693 0.537076 5.260791; 1 0.969487 1.902229 4.170348; 1 0.870454 -3.002805 2.402668; 1 1.177549 -2.662601 4.113087; 1 1.944838 -1.680362 2.860840; 6 -1.092707 -0.402139 -0.662790; 6 -1.941638 0.658423 -0.222595; 6 1.072157 0.410755 0.616330; 6 -1.209597 1.832221 0.156748; 6 1.289194 1.648581 -0.060990; 6 0.048817 2.076576 -0.655663; 1 0.045876 2.963508 -1.293453; 6 -1.719961 -1.748030 -0.932984; 1 -2.001214 -1.855068 -1.989581; 1 -1.037267 -2.568054 -0.691017; 1 -2.634410 -1.888344 -0.339051; 1 -3.013406 0.539959 -0.052397; 1 1.945590 -0.108426 1.017398; 6 -1.906217 3.063214 0.693559; 1 -1.249725 3.634297 1.360831; 1 -2.200159 3.737099 -0.126512; 1 -2.812525 2.805371 1.252360; 6 2.598361 2.388859 -0.140916; 1 2.681617 2.991778 -1.051073; 1 2.673890 3.074403 0.715076; 1 3.455055 1.708243 -0.092382;
10-11_ts	77 -0.616586 0.014145 1.550770; 77 0.568424 0.414785 -1.441977; 6 2.421592 0.304234 -2.971431; 6 2.394504 -0.889224 -2.190333; 6 1.133678 -1.558350 -2.401671; 6 0.357394 -0.749064 -3.327007; 6 1.143591 0.422909 -3.632138; 6 0.116428 -0.370942 3.741364; 6 0.079828 -1.546722 2.891486; 6 -1.323171 -1.874242 2.607917; 6 -2.105893 -0.805274 3.103415; 6 -1.204535 0.148442 3.763968; 6 0.772267 1.480937 -4.631731; 6 3.518689 -1.415529 -1.344276; 6 3.584068 1.239131 -3.135757; 6 -0.874659 -1.177592 -4.071601; 6 0.827770 -2.964024 -1.968880; 6 -3.601976 -0.688325 3.075499; 6 -1.663817 1.393273 4.465042; 6 -1.805446 -3.151838 1.988693; 6 1.345333 0.229214 4.356122; 6 1.240345 -2.464051 2.636214; 1 1.133714 1.214713 -5.635577; 1 1.206072 2.451814 -4.373061; 1 -0.312067 1.608265 -4.690445; 1 4.140860 -0.606515 -0.949451; 1 4.172691 -2.079118 -1.927932; 1 3.141288 -1.989569 -0.493220; 1 3.256035 2.266225 -3.324708; 1 4.213329 0.940402 -3.986615; 1 4.224932 1.255697 -2.248911; 1 -1.445718 -1.924842 -3.516651; 1 -0.601498 -1.623354 -5.039441; 1 -1.542238 -0.334000 -4.271690; 1 1.159575 -3.152855 -0.942705; 1 1.334017 -3.693284 -2.618735; 1 -0.242766 -3.177018 -2.014197; 1 -4.043253 -1.026811 4.024012; 1 -4.037341 -1.292534 2.274173; 1 -3.920489 0.347158 2.918564; 1 -0.850873 2.116282

	4.573883; 1 -2.042081 1.158464 5.470364; 1 -2.471528 1.884339 3.914454; 1 -2.798012 -3.037523 1.544720; 1 -1.867086 -3.949700 2.742896; 1 -1.129882 -3.496556 1.199986; 1 2.202960 0.177737 3.675475; 1 1.630954 -0.293076 5.280090; 1 1.193099 1.283117 4.607656; 1 1.108989 -3.023075 1.705054; 1 1.347315 -3.193239 3.452453; 1 2.179502 -1.907806 2.563155; 6 -1.264561 0.060610 -0.417690; 6 -2.007013 1.312074 -0.174348; 6 0.957017 1.026205 0.757748; 6 -1.464391 2.512810 -0.522274; 6 1.054416 2.107225 -0.142527; 6 -0.076715 2.485394 -0.989020; 1 0.177631 3.266426 -1.710503; 6 -2.170932 -1.075390 -0.863807; 1 -2.563161 -0.904145 -1.876379; 1 -1.664331 -2.044952 -0.855069; 1 -3.044395 -1.157882 -0.197667; 1 -3.032049 1.255092 0.199899; 1 1.924171 0.774657 1.205583; 6 -2.178388 3.832941 -0.415736; 1 -1.734645 4.478660 0.357879; 1 -2.116535 4.394721 -1.359023; 1 -3.238767 3.701420 -0.173085; 6 2.356108 2.873560 -0.285612; 1 2.467075 3.307873 -1.284623; 1 2.371170 3.704412 0.433623; 1 3.229318 2.244409 -0.083143;
11	77 -0.556524 0.010193 1.312223; 77 0.350567 0.385067 -1.316051; 6 2.603872 0.367136 -2.236138; 6 2.396204 -0.892178 -1.610482; 6 1.242134 -1.519734 -2.212895; 6 0.738424 -0.634673 -3.243737; 6 1.567605 0.554414 -3.221429; 6 0.616901 -0.884498 3.128810; 6 -0.056549 -2.036136 2.548981; 6 -1.454085 -1.818993 2.622994; 6 -1.677945 -0.504551 3.183680; 6 -0.382078 0.037808 3.564043; 6 1.500940 1.676419 -4.219164; 6 3.296070 -1.514504 -0.583163; 6 3.737966 1.310283 -1.955431; 6 -0.279207 -0.977114 -4.293804; 6 0.865843 -2.963591 -2.036680; 6 -3.012804 0.055919 3.585643; 6 -0.157262 1.296582 4.350982; 6 -2.531607 -2.808751 2.288369; 6 2.094976 -0.781343 3.377400; 6 0.619927 -3.301742 2.108517; 1 2.129322 1.456208 -5.093856; 1 1.853239 2.622129 -3.795670; 1 0.480066 1.833810 -4.578645; 1 3.699267 -0.768512 0.108708; 1 4.150462 -2.014441 -1.062623; 1 2.767395 -2.266269 0.007213; 1 3.475951 2.344725 -2.194142; 1 4.622507 1.052479 -2.555387; 1 4.038139 1.279360 -0.902907; 1 -1.037871 -1.663995 -3.908828; 1 0.196277 -1.459443 -5.160658; 1 -0.799599 -0.084549 -4.653073; 1 0.918239 -3.276054 -0.989764; 1 1.549700 -3.608048 -2.608012; 1 -0.146438 -3.164562 -2.393625; 1 -3.314911 -0.316193 4.576242; 1 -3.795096 -0.222784 2.873599; 1 -2.988709 1.148032 3.633452; 1 0.815389 1.746521 4.125421; 1 -0.183598 1.099228 5.431634; 1 -0.923586 2.046835 4.132409; 1 -3.442685 -2.314918 1.937963; 1 -2.801957 -3.397292 3.177033; 1 -2.218170 -3.513420 1.513057; 1 2.673767 -1.210301 2.553818; 1 2.381756 -1.316309 4.294794; 1 2.410314 0.259690 3.494126; 1 0.038608 -3.830401 1.346706; 1 0.752982 -3.993280 2.953726; 1 1.613827 -3.106031 1.695884; 6 -1.514521 -0.190771 -0.536860; 6 -2.562883 0.826006 -0.776488; 6 -0.002311 1.796700 0.921351; 6 -2.216190 2.013030 -1.295283; 6 0.165688 2.382500 -0.373122; 6 -0.758940 2.183680 -1.500422; 1 -0.490362 2.800434 -2.362545; 6 -2.034719 -1.589468 -0.848313; 1 -2.237770 -1.700161 -1.924653; 1 -1.341163 -2.378305 -0.549275; 1 -2.990311 -1.768711 -0.334409; 1 -3.595886 0.589777 -0.509442; 1 0.518065 2.345062 1.721975; 6 -3.142821 3.169581 -1.556447; 1 -2.930162 4.017801 -0.888280; 1 -3.040019 3.548268 -2.583768; 1 -4.189891 2.883666 -1.408365; 6 1.227178 3.463697 -0.514070; 1 1.457120 3.669394 -1.564891; 1 0.858308 4.400787 -0.072388; 1 2.159650 3.201638 -0.003314;
11-2_ts	77 -0.461769 -0.154784 1.633009; 77 0.681178 -0.121875 -0.974836; 6 2.724822 -0.583784 -2.165468; 6 2.478522 -1.659868 -1.284429; 6 1.160182 -2.202992 -1.588666; 6 0.631857 -1.483707 -2.732364; 6 1.561144 -0.425537 -3.026709; 6 0.148906 -0.428714 3.861981; 6 0.106624 -1.729763 3.277790; 6 -1.250813 -2.005202 2.837891; 6 -2.035995 -0.846731 3.115563; 6 -1.163205 0.158452 3.702483; 6 1.468897 0.512437 -4.196862; 6 3.411828 -2.202042 -0.241734; 6 3.990074 0.216357 -2.271155; 6 -0.536920 -1.879789 -3.586856; 6 0.643609 -3.503923 -1.045281; 6 -3.522741 -0.711487 2.956668; 6 -1.613059 1.470356 4.279437; 6 -1.756054 -3.330769 2.345293; 6 1.333296 0.224677 4.512097; 6 1.234722 -2.719663 3.257745; 1 1.928444 0.066741 -5.091544; 1 1.982995 1.457523 -3.999440; 1 0.428227 0.746147 -4.437343; 1 4.143505 -1.452510 0.074260; 1 3.971192 -3.067736 -0.624955; 1 2.867185 -2.528999 0.647963; 1 3.795307 1.250737 -2.567151; 1 4.657891 -0.216931 -3.029248; 1 4.540461 0.239368 -1.325873; 1 -1.258102 -2.485377 -3.033934; 1 -0.196903 -2.473230 -4.448057; 1 -1.067885 -1.005065 -3.974254; 1 0.811767 -3.580184 0.032456; 1 1.154319 -4.351083 -1.525718; 1 -0.427402 -3.621976 -1.223859; 1 -4.042310 -0.992420 3.884251; 1 -3.905316 -1.354656 2.158721; 1 -3.807533 0.317528 2.717784; 1 -0.802374 2.204450 4.284049; 1 -1.957551 1.344617 5.316882; 1 -2.438088 1.897628 3.702881; 1 -2.662397 -3.216305 1.744141; 1 -2.000203 -4.000452 3.183317; 1 -1.014955 -3.844369 1.724684; 1 2.276847 -0.148617 4.099810; 1 1.353474 0.038835 5.595405; 1 1.319629 1.309717 4.366512; 1 1.160085 -3.398416 2.402919; 1 1.226240 -3.339372 4.166146; 1 2.208323 -2.223011 3.209180; 6 -1.287317 -0.061322 -0.274274; 6 -1.949142 1.241519 -0.294580; 6 0.930562 1.079229 0.937561; 6 -1.394621 2.381633 -0.798278; 6 1.055241 1.969579 -0.178143; 6 0.014194 2.410084 -1.045414; 1 0.351123 3.107199 -1.817903; 6 -2.249689 -1.180524 -0.652518; 1 -2.539146 -1.115020 -1.711717; 1 -1.830242 -2.173431 -0.472202; 1 -3.180393 -1.103735 -0.070277; 1 -2.998070 1.255595 0.015209; 1 1.866696 1.044411 1.507387; 6 -2.191655 3.640676 -1.060480; 1 -1.962081 4.446210 -0.347360; 1 -1.978305 4.040068 -2.062514; 1 -3.268740 3.448597 -1.005359; 6 2.403972 2.653102 -0.368685; 1 2.566813 2.955258 -1.409126; 1 2.439250 3.565304 0.243194; 1 3.237650 2.015737 -0.061159;
14-16_ts	77 0.427258 2.541808 1.415703; 6 -0.302117 3.427786 -0.473526; 6 -1.546539 4.083795 -0.589563; 6 2.416126 1.647806 2.218136; 6 1.714368 2.416777 3.229485; 6 1.786254 3.826262 2.857325; 6 2.418085 3.902276 1.595237; 6 2.785922 2.553698 1.181967; 6 3.653561 2.213303 0.000938; 6 2.734137 5.150611 0.824838; 6 1.312388 4.967645 3.710447; 6 1.360616 1.936009 4.610695; 6 2.821165 0.204531 2.304330; 1 -2.312076 3.942815 0.162933; 6 -1.816136 4.952104 -1.647421; 6 -0.867412 5.179237 -2.647154; 1 -1.083651 5.854466 -3.470682; 6 0.351989 4.503369 -2.583958; 6 0.619239 3.633970 -1.526717; 1 1.551318 3.079062 -1.521334; 1 0.393224 4.712899 4.247161; 1 1.105854 5.860594 3.113603; 1 2.065915 5.238500 4.464460; 1 0.480678 2.450735 5.009108; 1 2.191338 2.125696 5.305284; 1 1.154021 0.863614 4.630612; 1 2.122968 -0.382506 2.907243; 1 3.814192 0.104284 2.767679; 1 2.871345 -0.258059 1.313872; 1 3.606085 2.983437 -0.773961; 1 3.368991 1.259284 -0.454693; 1 4.707579 2.129692 0.303777; 1 2.216930 6.019301 1.241227; 1 2.436941 5.066340 -0.225694; 1 3.812108 5.362253 0.848550; 1 -0.187012 2.153317 -0.118566; 6 -1.891622 -1.122559 2.549973; 6 -1.054662 0.104508 2.912935; 1 0.000969 -0.194281 2.949493; 6 -1.298358 1.237374 1.913952; 6 -2.123844 0.749394 0.766888; 1 -2.441092 1.519677 0.065916; 6 -1.946360 -0.570192 0.187071; 6 -1.819836 -1.595214 1.181846; 1 -1.774986 -2.647520 0.909782; 6 -1.956273 -2.163576 3.649072; 1 -2.447883 -1.766908 4.545242; 1 -0.945370 -2.481448 3.950528; 1 -2.506910 -3.055648 3.330536; 6 -1.466553 2.602140 2.354029; 6 -1.922607 -0.845096 -1.291698; 1 -2.255535 -1.864441 -1.519604; 1 -0.903482 -0.727487 -1.686011; 1 -2.565252 -0.142832 -1.833940; 1 -1.369351 2.807412 3.421305; 1 -2.234088 3.216719 1.887815; 1 -4.947770 -1.143789 4.777304; 6 -5.512334 -1.750653 4.063190; 6 -5.512060 -1.131604 2.694868; 1 -5.068443 -2.750529 4.057439; 1 -6.537431 -1.846202 4.448325; 6 -5.510590 0.286599 2.418834; 6 -5.686750 -1.840249 1.451551; 6 -5.644893 0.437671 0.983984; 6 -5.548365 1.392356 3.435069; 6 -5.776156 -0.871830 0.397787; 6 -5.842194 -3.326575 1.299054; 77 -3.692871 -0.582661 1.449386; 6 -5.796816 1.744652 0.257749; 1 -5.019488 1.111438 4.350855; 1 -6.582553 1.644776 3.713428; 1 -5.076161 2.302813 3.054244; 6 -6.026418 -1.167348 -

	1.053402; 1 -5.461163 -3.675698 0.334077; 1 -6.899229 -3.624093 1.360666; 1 -5.305526 -3.870385 2.082289; 1 -5.240809 2.544052 0.756179; 1 -6.851667 2.050966 0.215189; 1 -5.431362 1.680866 -0.771923; 1 -5.594900 -0.398390 -1.701597; 1 -7.103439 -1.210860 -1.270824; 1 -5.594724 -2.128721 -1.349957; 1 -1.322432 0.437286 3.924492; 1 1.094488 4.639495 -3.367025; 1 -2.776737 5.461037 -1.685532;
16(19)	77 0.603933 2.371599 1.373040; 6 -0.166100 3.303029 -0.302622; 6 -0.762511 4.578697 -0.207778; 6 2.680307 1.753027 2.178857; 6 2.009305 2.281065 3.317795; 6 1.734692 3.686389 3.068274; 6 2.247317 4.022587 1.786186; 6 2.783869 2.815362 1.186979; 6 3.582420 2.766479 -0.086192; 6 2.301356 5.386672 1.163045; 6 1.125077 4.627664 4.067270; 6 1.774738 1.579960 4.625792; 6 3.284890 0.382062 2.057698; 1 -0.876024 5.042451 0.769937; 6 -1.210175 5.280941 -1.331401; 6 -1.085279 4.726472 -2.605704; 1 -1.435482 5.266559 -3.481956; 6 -0.500942 3.464923 -2.733336; 6 -0.051293 2.771936 -1.605229; 1 0.400514 1.793195 -1.743571; 1 0.299379 4.159873 4.614157; 1 0.735323 5.530082 3.588172; 1 1.867510 4.943626 4.813788; 1 0.833605 1.889910 5.091346; 1 2.579375 1.804233 5.340891; 1 1.740714 0.493849 4.503642; 1 2.719813 -0.362036 2.626694; 1 4.316228 0.377584 2.437919; 1 3.314522 0.048712 1.016974; 1 3.137972 3.402656 -0.857146; 1 3.628699 1.750364 -0.488578; 1 4.614794 3.107214 0.078038; 1 1.588078 6.073954 1.626610; 1 2.072958 5.349974 0.094496; 1 3.304057 5.821487 1.278760; 1 0.586300 1.127080 0.387189; 6 -1.948364 -1.114000 2.610958; 6 -1.079800 0.100575 2.940389; 1 -0.027224 -0.210598 2.997521; 6 -1.295769 1.187118 1.890344; 6 -2.059360 0.680735 0.719508; 1 -2.296905 1.432346 -0.029517; 6 -1.903259 -0.673707 0.221399; 6 -1.850318 -1.651099 1.268693; 1 -1.836868 -2.716329 1.048516; 6 -2.084070 -2.098773 3.753777; 1 -2.583987 -1.642339 4.616095; 1 -1.095225 -2.440832 4.098630; 1 -2.660490 -2.982761 3.459568; 6 -1.377147 2.562331 2.230696; 6 -1.836232 -1.023900 -1.239670; 1 -2.206619 -2.037816 -1.430640; 1 -0.798131 -0.970715 -1.595645; 1 -2.422073 -0.320439 -1.840456; 1 -1.287816 2.847983 3.278779; 1 -2.016607 3.214680 1.641129; 1 -5.112770 -0.884254 4.723807; 6 -5.654428 -1.518277 4.015716; 6 -5.575235 -0.974848 2.617907; 1 -5.233138 -2.525484 4.085352; 1 -6.698925 -1.572916 4.353708; 6 -5.514043 0.425048 2.265621; 6 -5.722109 -1.745343 1.407727; 6 -5.584962 0.501421 0.819882; 6 -5.552995 1.586758 3.217143; 6 -5.737828 -0.833420 0.300222; 6 -5.920920 -3.232141 1.328618; 77 -3.693826 -0.558495 1.420589; 6 -5.653977 1.770443 0.017771; 1 -5.072388 1.340666 4.168859; 1 -6.588001 1.888444 3.436676; 1 -5.034822 2.458383 2.806525; 6 -5.940150 -1.200554 -1.141861; 1 -5.511176 -3.645158 0.401451; 1 -6.989014 -3.492270 1.359423; 1 -5.436270 -3.748229 2.162805; 1 -5.085763 2.575099 0.492960; 1 -6.693155 2.113069 -0.086228; 1 -5.249194 1.634452 -0.989664; 1 -5.460706 -0.480518 -1.811864; 1 -7.008916 -1.224449 -1.398936; 1 -5.527722 -2.188871 -1.368996; 1 -1.349888 0.485510 3.932875; 1 -0.391705 3.013277 -3.717746; 1 -1.659736 6.264415 -1.206120;
16-17_ts	77 0.299649 1.182669 4.877062; 6 0.056907 -0.490418 8.918883; 6 -0.237858 0.128244 7.699456; 6 2.252614 2.246831 4.568215; 6 1.454102 3.044827 5.493335; 6 0.324881 3.538725 4.775190; 6 0.416467 3.083316 3.404106; 6 1.619382 2.326590 3.274439; 6 2.208255 1.806351 1.998598; 6 -0.524826 3.466835 2.296660; 6 -0.740491 4.451883 5.313444; 6 1.842656 3.381186 6.904003; 6 3.652670 1.766597 4.826317; 1 -1.204917 0.616932 7.593791; 6 0.676789 0.149910 6.625191; 6 1.909188 -0.497152 6.853872; 1 2.646702 -0.534888 6.056621; 6 2.215799 -1.112408 8.071465; 6 1.289260 -1.114556 9.115539; 1 1.522856 -1.594074 10.062941; 1 -0.855125 4.343106 6.395293; 1 -1.713940 4.254292 4.853725; 1 -0.490748 5.502962 5.110432; 1 0.975111 3.677208 7.500845; 1 2.561040 4.213010 6.922335; 1 2.304500 2.525404 7.403687; 1 3.792857 1.485615 5.872886; 1 4.383749 2.555008 4.594075; 1 3.901958 0.894679 4.213241; 1 1.441163 1.586661 1.252450; 1 2.783645 0.892109 2.166017; 1 2.889673 2.550865 1.562196; 1 -1.561731 3.517261 2.645719; 1 -0.489632 2.749317 1.471090; 1 -0.275618 4.453796 1.880971; 6 0.030789 -3.143730 3.432528; 6 0.102948 -2.057563 4.508370; 1 1.125212 -2.020261 4.904448; 6 -0.328918 -0.710336 3.931970; 6 -0.311611 -0.759880 2.431806; 1 -0.648312 0.151632 1.937006; 6 0.596114 -1.589119 1.656653; 6 0.770059 -2.902248 2.212985; 1 1.346222 -3.664595 1.693071; 6 0.020783 -4.555210 3.981572; 1 -0.878192 -4.742408 4.580082; 1 0.886035 -4.722877 4.641509; 1 0.056337 -5.303789 3.181925; 6 -1.509916 -0.060773 4.509371; 6 1.230034 -1.186982 0.350051; 1 1.335618 -2.050148 -0.317009; 1 2.230173 -0.762749 0.506565; 1 0.628243 -0.430748 -0.166439; 1 -1.993822 -0.592716 5.330647; 1 -2.221985 0.386079 3.816104; 1 -1.275281 1.062273 5.393116; 1 -3.283463 -4.990797 3.616160; 6 -3.206651 -5.290480 2.566979; 6 -2.992688 -4.104946 1.670242; 1 -2.388390 -6.012351 2.489784; 1 -4.136217 -5.814977 2.304073; 6 -3.533166 -2.782974 1.888833; 6 -2.381540 -4.139500 0.363809; 6 -3.199819 -1.993362 0.722052; 6 -4.430510 -2.366600 3.019274; 6 -2.516692 -2.840188 -0.223856; 6 -1.787399 -5.351125 -0.296045; 77 -1.300559 -2.598264 1.781324; 6 -3.652342 -0.582989 0.467281; 1 -4.177613 -2.890080 3.946286; 1 -5.485120 -2.584375 2.793343; 1 -4.353497 -1.293604 3.219382; 6 -2.075536 -2.444361 -1.603675; 1 -0.985967 -5.081175 -0.991158; 1 -2.546020 -5.903406 -0.869551; 1 -1.367352 -6.044092 0.439136; 1 -3.732532 -0.015100 1.398976; 1 -4.639466 -0.566679 -0.016013; 1 -2.957803 -0.047352 -0.187501; 1 -1.827948 -1.379624 -1.657808; 1 -2.865156 -2.633699 -2.345087; 1 -1.190110 -3.004961 -1.920475; 1 -0.681406 -0.478536 9.718669; 1 3.181690 -1.597902 8.199453; 1 -0.534106 -2.330385 5.358073;
17	77 -1.769896 3.192745 3.788348; 6 -3.422155 2.300843 2.429508; 6 -2.047159 2.187704 1.920005; 6 -1.625224 3.521060 1.485521; 6 -2.605819 4.427429 1.931847; 6 -3.729852 3.672863 2.504123; 6 -5.017448 4.293808 2.963944; 6 -2.587289 5.920229 1.781243; 6 -0.360935 3.828965 0.739907; 6 -1.399979 0.902179 1.492881; 6 -4.329434 1.136726 2.692070; 1 0.465945 3.201157 1.082604; 1 -0.054221 4.870148 0.874793; 1 -0.493092 3.656090 -0.337442; 1 -0.310524 0.987779 1.489906; 1 -1.721349 0.626080 0.477700; 1 -1.668416 0.080304 2.163486; 1 -3.806669 0.320444 3.198591; 1 -4.717617 0.740065 1.742947; 1 -5.188300 1.414994 3.308395; 1 -4.840101 5.200759 3.552309; 1 -5.598624 3.605415 3.584315; 1 -5.647552 4.581417 2.110240; 1 -1.576950 6.292759 1.591940; 1 -2.965161 6.419602 2.679533; 1 -3.221616 6.237564 0.941320; 6 2.425896 4.326057 4.058710; 6 1.027082 4.332382 4.060561; 1 0.519390 5.293606 4.091905; 6 0.267174 3.143113 4.013204; 6 1.009243 1.942619 3.959063; 1 0.480558 0.992447 3.926773; 6 2.407603 1.926894 3.950230; 6 3.128614 3.121334 4.003100; 1 4.215869 3.113442 3.999695; 1 -0.149109 1.669792 6.112685; 77 -2.141734 4.410899 8.304414; 6 -3.424219 3.645757 9.988589; 6 -4.171391 4.658646 9.280561; 6 -2.257744 4.293938 10.543521; 6 -3.857323 2.230365 10.243182; 6 -3.512229 5.930319 9.456921; 6 -5.499011 4.450141 8.608306; 6 -2.326761 5.705774 10.227592; 6 -1.269491 3.656406 11.477048; 1 -4.472928 1.844494 9.425137; 1 -4.450191 2.154929 11.167136; 1 -2.997925 1.561292 10.348368; 6 -4.012104 7.258190 8.964786; 1 -5.649849 5.155169 7.784907; 1 -6.324791 4.593324 9.319733; 1 -5.584635 3.439906 8.198561; 6 -1.370509 6.759298 10.707901; 1 -1.116609 2.599580 11.240466; 1 -1.622262 3.715582 12.516593; 1 -0.293224 4.147962 11.432230; 1 -3.188120 7.950097 8.763016; 1 -4.668754 7.737681 9.704964; 1 -4.588720 7.154283 8.040067; 1 -0.361714 6.356921 10.839071; 1 -1.688732 7.169515 11.677377; 1 -1.304385 7.595393 10.004675; 6 -1.479999 5.037333 6.365481; 6 -2.477938 4.008597 6.106806; 6 -0.359334 4.553732 7.120053; 6 -2.131886 2.569381 5.768057; 1 -3.455069 4.363514 5.781403; 6 -0.470090 3.193901 7.600599; 1 0.456748 5.214859 7.399371; 6 -0.986359 2.089443 6.681023; 1 -1.356176 1.277539 7.324978; 6 -3.350664 1.660017 5.925988; 1 -4.223257 2.040223

	5.383583; 1 -3.136354 0.650428 5.555136; 1 -3.628344 1.571659 6.987686; 6 0.591948 2.719880 8.572556; 1 0.947776 3.529681 9.218860; 1 0.212372 1.916340 9.214999; 1 1.457875 2.316774 8.026698; 6 -1.595480 6.456944 5.876124; 1 -1.214686 6.553922 4.850995; 1 -2.641815 6.783462 5.872357; 1 -1.027081 7.142198 6.515065; 1 2.934032 0.975053 3.904989; 1 2.966799 5.270156 4.097714;
17-18_ts	77 -1.100864 2.376965 4.037744; 6 -1.924969 0.321613 3.084040; 6 -0.506183 0.452071 2.824458; 6 -0.293670 1.622325 2.037681; 6 -1.590774 2.258821 1.838229; 6 -2.588107 1.414077 2.447010; 6 -4.070125 1.599568 2.299392; 6 -1.863306 3.379662 0.876434; 6 0.994226 2.061737 1.404295; 6 0.540860 -0.546232 3.232175; 6 -2.573089 -0.839237 3.785430; 1 1.862415 1.688168 1.955131; 1 1.073031 3.151538 1.374714; 1 1.067806 1.689142 0.372525; 1 1.532293 -0.088521 3.286555; 1 0.595500 -1.371950 2.508617; 1 0.322820 -0.984665 4.211033; 1 -1.968644 -1.191382 4.628455; 1 -2.708358 -1.693881 3.107022; 1 -3.561349 -0.577480 4.176029; 1 -4.351286 2.656311 2.330298; 1 -4.631981 1.082394 3.081117; 1 -4.406941 1.198653 1.332825; 1 -1.057214 4.117405 0.882381; 1 -2.791721 3.904699 1.122036; 1 -1.960800 2.996460 -0.150002; 6 0.283020 6.399956 3.098963; 6 -0.511916 5.276977 3.355503; 1 -1.561139 5.307239 3.072758; 6 0.002724 4.113090 3.963666; 6 1.362643 4.161539 4.337242; 1 1.814628 3.295497 4.816142; 6 2.161095 5.285128 4.100898; 6 1.629487 6.412153 3.469473; 1 2.250770 7.282832 3.273993; 1 -0.232640 2.022002 5.423675; 77 -2.044321 4.237911 8.473982; 6 -3.709865 3.594307 9.833607; 6 -3.914177 4.979495 9.478035; 6 -2.472034 3.533571 10.581632; 6 -4.686540 2.468451 9.646308; 6 -2.853671 5.766940 10.065845; 6 -5.124067 5.531888 8.778184; 6 -1.968298 4.875122 10.749139; 6 -1.924366 2.304922 11.250002; 1 -5.304058 2.614676 8.755254; 1 -5.366261 2.384523 10.507944; 1 -4.176383 1.506555 9.536079; 6 -2.739652 7.263477 10.017847; 1 -4.885483 6.439715 8.215705; 1 -5.911689 5.788463 9.500844; 1 -5.542854 4.808155 8.073349; 6 -0.757667 5.268416 11.546558; 1 -2.147076 1.401721 10.674153; 1 -2.363171 2.175722 12.249561; 1 -0.838568 2.359058 11.372625; 1 -1.695261 7.590170 10.048473; 1 -3.254786 7.729871 10.869897; 1 -3.185102 7.671831 9.105618; 1 -0.005672 4.473628 11.555334; 1 -1.022140 5.479378 12.592861; 1 -0.282839 6.168076 11.142485; 6 -1.083391 5.122176 6.746452; 6 -2.305169 4.508209 6.286907; 6 -0.200983 4.155571 7.385335; 6 -2.370959 3.139573 5.655140; 1 -3.084392 5.202267 5.970570; 6 -0.677983 2.831306 7.478109; 1 0.738208 4.517977 7.826508; 6 -1.429678 2.241102 6.310138; 1 -1.761309 1.216911 6.485726; 6 -3.809614 2.663302 5.522465; 1 -4.353900 3.233433 4.760539; 1 -3.861988 1.604372 5.253049; 1 -4.337086 2.796043 6.478703; 6 0.159421 1.843216 8.263200; 1 0.647541 2.324320 9.117032; 1 -0.449888 1.016771 8.647601; 1 0.946216 1.402346 7.630758; 6 -0.773892 6.590322 6.639155; 1 -0.229916 6.795803 5.711154; 1 -1.695931 7.182452 6.627468; 1 -0.159199 6.928379 7.481238; 1 3.206562 5.274602 4.404289; 1 -0.156159 7.270164 2.613840;
18	77 -0.860898 2.262912 4.329853; 6 -1.670312 0.116990 3.650812; 6 -0.236445 0.156154 3.642013; 6 0.166420 1.133781 2.655677; 6 -1.038627 1.636572 2.022713; 6 -2.162964 1.015550 2.631605; 6 -3.593343 1.151595 2.193317; 6 -1.068967 2.566898 0.846157; 6 1.567169 1.387358 2.173449; 6 0.682681 -0.753885 4.408666; 6 -2.519307 -0.825644 4.455009; 1 2.303579 1.144425 2.945223; 1 1.711739 2.437161 1.901803; 1 1.798903 0.776293 1.289476; 1 1.648507 -0.277651 4.598304; 1 0.873383 -1.681211 3.849709; 1 0.259798 -1.034306 5.377944; 1 -2.003786 -1.162221 5.359053; 1 -2.773178 -1.720294 3.869401; 1 -3.463836 -0.364536 4.763851; 1 -3.818501 2.163822 1.843523; 1 -4.292184 0.918237 3.001980; 1 -3.817845 0.462755 1.366015; 1 -0.261605 3.301647 0.893734; 1 -2.011785 3.118376 0.789212; 1 -0.956359 2.002218 -0.090313; 6 0.525145 6.708167 2.716337; 6 -0.816003 6.323307 2.674776; 1 -1.560365 6.988753 2.241236; 6 -1.213473 5.084321 3.186591; 6 -0.297752 4.179910 3.759473; 6 1.049341 4.595599 3.788768; 6 1.456890 5.832450 3.275981; 1 2.508587 6.109894 3.318070; 1 0.375499 2.492414 5.276203; 77 -2.007023 4.066426 8.611678; 6 -3.953512 3.944837 9.706270; 6 -3.607925 5.341804 9.541503; 6 -2.935743 3.362497 10.549841; 6 -5.224562 3.281635 9.257507; 6 -2.442728 5.625371 10.345144; 6 -4.438262 6.363319 8.816396; 6 -2.023173 4.405071 10.959249; 6 -2.944216 1.955107 11.074422; 1 -5.598689 3.717870 8.326438; 1 -6.017927 3.387539 10.013211; 1 -5.079780 2.211289 9.081406; 6 -1.807007 6.973546 10.526791; 1 -3.823469 7.181726 8.429220; 1 -5.192141 6.803727 9.484341; 1 -4.966248 5.918228 7.968200; 6 -0.876430 4.242753 11.915867; 1 -3.395149 1.263445 10.356604; 1 -3.522500 1.888711 12.006958; 1 -1.933295 1.595878 11.288296; 1 -0.733949 6.889678 10.726419; 1 -2.256162 7.516456 11.370915; 1 -1.928154 7.599209 9.637091; 1 -0.454960 3.233910 11.871472; 1 -1.195251 4.417457 12.953750; 1 -0.067548 4.948144 11.700271; 6 -0.645443 4.703426 7.059757; 6 -1.951324 4.526494 6.462440; 6 -0.157139 3.508687 7.677268; 6 -2.325994 3.283326 5.701138; 1 -2.439965 5.442551 6.132610; 6 -1.025986 2.356374 7.587690; 1 0.779799 3.496497 8.229057; 6 -1.782827 2.118954 6.309513; 1 -2.366946 1.197996 6.302388; 6 -3.738978 3.296817 5.156067; 1 -3.873784 4.038141 4.359612; 1 -4.031084 2.319529 4.762911; 1 -4.437550 3.558770 5.965434; 6 -0.627351 1.109864 8.348341; 1 -0.104372 1.355253 9.278899; 1 -1.500937 0.499543 8.607042; 1 0.042340 0.482590 7.739975; 6 0.108256 6.005305 7.066693; 1 0.692054 6.107500 6.144251; 1 -0.580735 6.855627 7.118314; 1 0.794781 6.066044 7.919108; 1 1.800447 3.940978 4.222913; 1 0.837827 7.670951 2.319708; 1 -2.265196 4.817129 3.129791;
18-9_ts	77 -1.041032 2.384970 4.284657; 6 -1.965827 0.474166 3.558462; 6 -0.540305 0.210319 3.688695; 6 0.126431 1.015012 2.727563; 6 -0.882536 1.712568 1.936959; 6 -2.162589 1.345579 2.410583; 6 -3.489927 1.731307 1.824935; 6 -0.576882 2.603146 0.768112; 6 1.595166 0.974793 2.409881; 6 0.084465 -0.822050 4.582779; 6 -3.041637 -0.360749 4.197770; 1 2.201327 0.816119 3.307751; 1 1.933180 1.900636 1.937053; 1 1.821149 0.154222 1.713034; 1 1.123583 -0.573074 4.818343; 1 0.082128 -1.812057 4.102285; 1 -0.454663 -0.917894 5.529891; 1 -2.805065 -0.613381 5.236087; 1 -3.164220 -1.308137 3.653678; 1 -4.012148 0.143326 4.191697; 1 -3.419784 2.652553 1.239046; 1 -4.244419 1.892993 2.600490; 1 -3.871858 0.945122 1.157203; 1 0.231731 3.304450 0.998531; 1 -1.449018 3.192751 0.472233; 1 -0.263526 2.012655 -0.104216; 6 0.912681 6.560121 2.483308; 6 -0.402711 6.526709 2.955615; 1 -1.063676 7.372361 2.779021; 6 -0.884601 5.415573 3.648373; 6 -0.070558 4.282314 3.865150; 6 1.263417 4.350338 3.406337; 6 1.746491 5.466238 2.720420; 1 2.779618 5.482009 2.380916; 1 -0.003846 3.534001 4.971430; 77 -2.025138 4.090990 8.625636; 6 -3.827020 3.630851 9.870120; 6 -3.777617 5.060322 9.654872; 6 -2.654406 3.285450 10.642793; 6 -4.965530 2.712722 9.527615; 6 -2.633145 5.592238 10.357479; 6 -4.842890 5.877355 8.979069; 6 -1.939974 4.499112 10.962689; 6 -2.352070 1.921900 11.195333; 1 -5.501866 3.052671 8.636775; 1 -5.694460 2.656187 10.350473; 1 -4.614705 1.695559 9.327975; 6 -2.266849 7.044520 10.466539; 1 -4.429248 6.783423 8.525300; 1 -5.613696 6.190633 9.697618; 1 -5.338876 5.309229 8.186933; 6 -0.718929 4.597511 11.831930; 1 -2.702754 1.132145 10.524394; 1 -2.845472 1.774529 12.166610; 1 -1.278573 1.773454 11.345756; 1 -1.186649 7.181956 10.580838; 1 -2.750247 7.514480 11.335249; 1 -2.577968 7.605877 9.579903; 1 -0.114302 3.687008 11.781206; 1 -0.991702 4.749352 12.886452; 1 -0.079217 5.435543 11.537058; 6 -0.868614 4.902228 7.005616; 6 -2.172311 4.557580 6.475963; 6 -0.188020 3.772485 7.571882; 6 -2.455699 3.280001 5.718673; 1 -2.803358 5.408189 6.215255; 6 -0.905773 2.520233 7.517710; 1 0.775556 3.879937 8.064505; 6 -1.719954 2.182170 6.296222; 1 -2.223575 1.217544 6.383259; 6 -3.917055 3.148894 5.335476; 1 -4.202165 3.875531 4.563385; 1 -

	4.150345 2.151136 4.956835; 1 -4.556200 3.332047 6.213153; 6 -0.303895 1.335439 8.243207; 1 0.249364 1.644573 9.136610; 1 -1.076069 0.623129 8.557656; 1 0.390621 0.794007 7.583611; 6 -0.289084 6.292060 7.005150; 1 0.253877 6.486981 6.072077; 1 -1.081792 7.044179 7.088419; 1 0.404739 6.432964 7.841794; 1 1.931023 3.519730 3.612912; 1 1.284303 7.431506 1.950695; 1 -1.907834 5.412614 4.000698;
7-20_ts	77 -1.132872 2.838306 4.500047; 6 -2.954245 1.570554 4.518191; 6 -1.825901 0.652633 4.593580; 6 -1.101685 0.742935 3.367948; 6 -1.800057 1.674434 2.501122; 6 -2.950331 2.153438 3.188543; 6 -4.008818 3.057384 2.626360; 6 -1.427364 1.992595 1.081187; 6 0.088607 -0.086250 2.976025; 6 -1.550064 -0.301233 5.721861; 6 -4.089008 1.648933 5.502027; 1 0.648940 -0.421915 3.853225; 1 0.779012 0.471584 2.336505; 1 -0.221873 -0.982068 2.419634; 1 -0.482756 -0.522005 5.812443; 1 -2.075574 -1.253220 5.561558; 1 -1.885038 0.104815 6.679938; 1 -3.745419 1.460971 6.523002; 1 -4.866563 0.907543 5.268785; 1 -4.559380 2.636559 5.492696; 1 -3.598913 3.748627 1.883629; 1 -4.484456 3.654900 3.409501; 1 -4.799781 2.473771 2.134551; 1 -0.349722 1.906850 0.917704; 1 -1.724216 3.007838 0.802831; 1 -1.920390 1.300040 0.384355; 6 0.847313 6.886292 2.770148; 6 -0.286584 6.715602 3.587973; 1 -0.913172 7.570266 3.839160; 6 -0.591203 5.445083 4.055978; 6 0.189246 4.337424 3.698878; 6 1.320524 4.466226 2.888751; 6 1.621636 5.767642 2.442481; 1 2.501850 5.908388 1.816438; 6 1.228525 8.261971 2.270737; 1 1.559938 8.910609 3.092604; 1 0.379570 8.764797 1.790780; 1 2.043624 8.212184 1.541212; 6 -1.720185 4.868653 4.864932; 6 2.193817 3.297981 2.499548; 1 2.241297 2.552110 3.301100; 1 3.215284 3.622697 2.273257; 1 1.811909 2.786416 1.605322; 1 -1.707456 5.147675 5.922494; 1 -0.903559 2.940189 6.073388; 1 0.440933 3.272704 4.738487; 1 -2.715073 5.076581 4.453960;
20	77 -0.431244 2.530743 4.621166; 6 -2.592915 3.613246 5.835155; 6 -1.265449 3.531506 6.245176; 6 0.937807 4.311324 3.761344; 6 -0.044010 3.959984 2.780410; 6 0.163186 2.577371 2.403025; 6 1.283853 2.081610 3.159945; 6 1.750223 3.152520 4.013082; 6 2.978400 3.109186 4.878646; 6 1.955761 0.748965 2.978459; 6 -0.542897 1.859181 1.287096; 6 -1.026835 4.909494 2.159846; 6 1.119551 5.677981 4.356451; 1 0.093897 1.741936 5.904086; 6 -0.856564 4.081928 7.463237; 6 0.556322 3.986397 7.987349; 6 -1.848713 4.745085 8.212351; 1 -1.566357 5.186415 9.167936; 6 -3.183111 4.855852 7.798072; 6 -4.196480 5.589684 8.649025; 6 -3.566545 4.268535 6.579256; 1 -4.601738 4.321330 6.243860; 6 -2.580172 2.847675 4.543091; 1 -1.597827 2.143148 1.229295; 1 -0.502087 0.774916 1.421386; 1 -0.082851 2.092726 0.316738; 1 -1.868572 4.380657 1.704326; 1 -0.546076 5.501373 1.368574; 1 -1.430976 5.611115 2.896479; 1 0.159030 6.145459 4.592483; 1 1.648919 6.338800 3.655975; 1 1.704093 5.640403 5.278981; 1 3.185177 2.094500 5.229214; 1 2.875370 3.748193 5.759645; 1 3.859499 3.452875 4.319067; 1 1.247825 -0.013992 2.643837; 1 2.404513 0.394344 3.910518; 1 2.755490 0.814180 2.227483; 1 -2.828198 3.426044 3.646502; 1 -3.158048 1.921464 4.547789; 1 -1.064428 1.077593 4.694597; 1 -5.091354 4.980056 8.826865; 1 -4.533796 6.517636 8.167463; 1 -3.778648 5.859757 9.624738; 1 1.229317 4.690913 7.478971; 1 0.968457 2.982531 7.832147; 1 0.601006 4.212758 9.058372;
20-21_ts	77 -0.286055 2.740808 4.669262; 6 -2.518681 3.655591 5.915792; 6 -1.176428 3.668103 6.295042; 6 1.140909 4.416280 3.846998; 6 0.353982 3.993890 2.739844; 6 0.612908 2.582506 2.507585; 6 1.568046 2.140276 3.467727; 6 1.863401 3.261563 4.350851; 6 2.988967 3.297052 5.346539; 6 2.243368 0.798603 3.508633; 6 0.063313 1.782105 1.360061; 6 -0.532838 4.860997 1.893560; 6 1.255067 5.818904 4.370880; 1 -0.130251 1.616210 5.789670; 6 -0.818771 4.185913 7.546796; 6 0.598791 4.173690 8.067419; 6 -1.853037 4.711493 8.343345; 1 -1.595549 5.121959 9.319728; 6 -3.200551 4.714109 7.957123; 6 -4.261440 5.311809 8.854640; 6 -3.541192 4.153189 6.716347; 1 -4.582633 4.106877 6.399510; 6 -2.563131 2.981521 4.572480; 1 -0.973877 2.051860 1.138025; 1 0.088396 0.709435 1.570483; 1 0.648908 1.955381 0.446291; 1 -1.408771 4.313003 1.530763; 1 0.005141 5.230428 1.009171; 1 -0.891683 5.733608 2.447296; 1 0.330223 6.383495 4.222290; 1 2.061993 6.359769 3.856836; 1 1.478134 5.830737 5.440466; 1 3.097401 2.338954 5.862793; 1 2.831438 4.064423 6.107848; 1 3.943142 3.515250 4.845869; 1 1.606607 0.013395 3.091620; 1 2.495633 0.509387 4.532338; 1 3.176361 0.815576 2.927632; 1 -2.657083 3.638242 3.703996; 1 -3.308412 2.182424 4.480701; 1 -1.576073 1.750528 4.497265; 1 -5.189334 4.728303 8.825073; 1 -4.517889 6.337063 8.552778; 1 -3.926404 5.355690 9.896794; 1 1.199565 4.990075 7.642375; 1 1.101422 3.235436 7.810006; 1 0.624582 4.291139 9.156597;
21	77 -0.054164 1.678383 5.483482; 6 -2.482190 3.309321 5.934741; 6 -1.192325 3.083676 6.474452; 6 1.669718 2.795824 4.422921; 6 0.874305 2.253375 3.402740; 6 0.842767 0.784937 3.566600; 6 1.664497 0.425218 4.653912; 6 2.063298 1.675081 5.301137; 6 3.059347 1.768564 6.417214; 6 2.037824 -0.954702 5.109488; 6 0.132789 -0.145077 2.628902; 6 0.213357 2.990757 2.276863; 6 2.060826 4.230120 4.613427; 1 -0.590585 0.668249 6.621650; 6 -0.829866 3.812947 7.627969; 6 0.492322 3.588011 8.328988; 6 -1.713031 4.756839 8.170921; 1 -1.410976 5.301395 9.065291; 6 -2.971521 5.007704 7.620555; 6 -3.895029 6.050205 8.208223; 6 -3.341289 4.255511 6.503191; 1 -4.330332 4.397876 6.067324; 6 -2.961142 2.522918 4.728994; 1 -0.858345 0.235194 2.362483; 1 0.002827 -1.136781 3.069530; 1 0.701483 -0.262479 1.695838; 1 -0.770925 2.572373 2.045188; 1 0.818452 2.925325 1.361556; 1 0.078714 4.049680 2.512562; 1 1.362677 4.906909 4.113557; 1 3.062725 4.417052 4.201966; 1 2.081732 4.503760 5.671483; 1 2.911991 0.969687 7.149023; 1 2.990098 2.723302 6.942157; 1 4.079084 1.675767 6.016323; 1 1.303441 -1.697357 4.785483; 1 2.100991 -1.010620 6.199757; 1 3.014981 -1.250158 4.702151; 1 -4.055425 2.524067 4.655957; 1 -2.641624 1.469746 4.780902; 1 -2.570244 2.934431 3.788322; 1 -4.947964 5.779542 8.067715; 1 -3.750173 7.033236 7.737929; 1 -3.722333 6.179513 9.282803; 1 1.323693 4.083052 7.807925; 1 0.733833 2.521219 8.381710; 1 0.472844 3.988677 9.349028;
21-22_ts	77 -0.270220 1.648165 3.708754; 6 -1.428887 -0.276332 2.949037; 6 -0.038599 -0.489077 3.070072; 6 0.626850 0.480487 2.186138; 6 -0.419648 1.123805 1.373470; 6 -1.657025 0.703981 1.875042; 6 -3.020247 1.093030 1.383037; 6 -0.140179 2.052259 0.228401; 6 2.080697 0.462624 1.810152; 6 0.655447 -1.537898 3.889652; 6 -2.519759 -1.048786 3.633666; 1 2.703145 0.102488 2.633682; 1 2.431438 1.462036 1.537528; 1 2.249088 -0.202438 0.950193; 1 1.623278 -1.182416 4.253889; 1 0.835176 -2.442178 3.290704; 1 0.061092 -1.827945 4.761007; 1 -2.162365 -1.517456 4.555175; 1 -2.906001 -1.849095 2.985490; 1 -3.369587 -0.409733 3.895077; 1 -2.974214 1.911037 0.659417; 1 -3.673733 1.409982 2.202474; 1 -3.510164 0.241694 0.890441; 1 0.553886 2.847032 0.520974; 1 -1.051762 2.528927 -0.142162; 1 0.313041 1.507212 -0.611046; 6 1.923053 6.058822 3.242925; 6 0.615933 5.834247 2.816954; 1 0.075052 6.640682 2.321485; 6 -0.032792 4.602070 3.003254; 6 0.628202 3.508914 3.630266; 6 1.956483 3.756246 4.090255; 6 2.567462 4.999998 3.888127; 1 3.587200 5.145776 4.243753; 6 2.623674 7.375886 3.000879; 1 3.200786 7.693555 3.877954; 1 1.909047 8.171841 2.763456; 1 3.329954 7.311452 2.161257; 6 -1.479675 4.535428 2.556154; 6 2.781743 2.714379 4.820894; 1 2.339837 2.461455 5.792172; 1 3.798722 3.081899 5.001347; 1 2.853492 1.777554 4.260792; 1 -1.690825 5.285775 1.784574; 1 -2.155984 4.735551 3.397202; 1 0.525404 1.471060 5.062935; 77 -2.112339 4.176294 8.466028; 6 -3.671808 3.730887 10.005591; 6 -3.717703 5.147150 9.699808; 6 -2.380314 3.480319 10.596299; 6 -4.808529 2.755890 9.890965; 6 -2.512485 5.767953 10.197880; 6 -4.911070 5.876666

	9.149772; 6 -1.681152 4.741258 10.733826; 6 -1.924395 2.168711 11.168789; 1 -5.464966 3.002025 9.050932; 1 -5.425455 2.752196 10.802408; 1 -4.447232 1.735075 9.733547; 6 -2.198164 7.235669 10.160190; 1 -4.615935 6.770753 8.591850; 1 -5.581784 6.198689 9.958853; 1 -5.489634 5.240701 8.473533; 6 -0.341482 4.932491 11.385554; 1 -2.369191 1.323224 10.636074; 1 -2.213771 2.084344 12.225833; 1 -0.837413 2.057318 11.115067; 1 -1.121357 7.415762 10.078531; 1 -2.547214 7.742952 11.071037; 1 -2.679559 7.728565 9.309939; 1 0.292190 4.048091 11.269269; 1 -0.446119 5.119207 12.464128; 1 0.197957 5.783580 10.957807; 6 -1.318094 4.925606 6.613242; 6 -2.655873 4.389708 6.362944; 6 -0.432824 3.938532 7.142157; 6 -2.801542 3.041407 5.758020; 1 -3.419421 5.123160 6.104625; 6 -1.014112 2.618796 7.320561; 1 0.580325 4.185785 7.446472; 6 -1.916580 2.132666 6.235389; 1 -1.945721 1.076797 5.985318; 6 -3.953396 2.758852 4.838338; 1 -3.915422 3.360289 3.921764; 1 -3.990671 1.702731 4.556290; 1 -4.900408 3.006949 5.339791; 6 -0.203381 1.565266 8.043056; 1 0.438551 2.009632 8.811094; 1 -0.851648 0.829039 8.532498; 1 0.437423 1.020535 7.334649; 6 -0.915647 6.348508 6.339132; 1 -0.516616 6.437431 5.321089; 1 -1.775234 7.022436 6.429374; 1 -0.140206 6.686255 7.035321; 1 -1.748147 3.551857 2.163462;
22	77 -0.905497 2.257283 4.302649; 6 -1.874193 0.193281 3.557133; 6 -0.442353 0.136638 3.535976; 6 0.018775 1.110327 2.572974; 6 -1.153516 1.698839 1.950655; 6 -2.309878 1.136449 2.549252; 6 -3.731185 1.354545 2.113465; 6 -1.100741 2.627711 0.772510; 6 1.417002 1.262263 2.042243; 6 0.416764 -0.850221 4.277186; 6 -2.773991 -0.720308 4.340666; 1 2.156604 0.837468 2.726582; 1 1.674010 2.314327 1.884520; 1 1.527290 0.743330 1.079170; 1 1.420723 -0.453950 4.452944; 1 0.523470 -1.783347 3.705909; 1 -0.009303 -1.107658 5.251508; 1 -2.283216 -1.089517 5.246252; 1 -3.060205 -1.598039 3.744165; 1 -3.700553 -0.223283 4.646713; 1 -3.900252 2.374705 1.755358; 1 -4.440962 1.168892 2.924279; 1 -3.995010 0.672859 1.292195; 1 -0.443154 3.481621 0.963300; 1 -2.089487 3.016855 0.516061; 1 -0.713838 2.100705 -0.110698; 6 1.230589 6.579088 3.021290; 6 -0.121109 6.334601 2.798700; 1 -0.716519 7.088883 2.284550; 6 -0.754092 5.151134 3.213892; 6 -0.031657 4.126967 3.885161; 6 1.352602 4.385747 4.121948; 6 1.942903 5.584076 3.691637; 1 3.002189 5.742542 3.890360; 6 1.902478 7.842831 2.535980; 1 2.688956 8.170855 3.225740; 1 1.183967 8.663474 2.430402; 1 2.376086 7.699001 1.554654; 6 -2.238826 5.078402 2.921045; 6 2.281233 3.437538 4.859052; 1 1.971266 3.295828 5.901398; 1 3.302756 3.834519 4.871216; 1 2.316046 2.444745 4.401232; 1 -2.490602 5.676525 2.037286; 1 -2.824265 5.480699 3.757382; 1 0.315979 2.228540 5.270567; 77 -2.043714 4.105612 8.592977; 6 -3.902181 3.772761 9.793654; 6 -3.756467 5.193549 9.560484; 6 -2.774206 3.367655 10.602323; 6 -5.086812 2.922903 9.431935; 6 -2.601676 5.664503 10.289880; 6 -4.749855 6.065529 8.844968; 6 -1.994769 4.539407 10.927593; 6 -2.567424 1.995408 11.177515; 1 -5.581572 3.288719 8.527278; 1 -5.836233 2.917113 10.238141; 1 -4.794137 1.884891 9.246259; 6 -2.146861 7.092091 10.389828; 1 -4.267833 6.936883 8.391074; 1 -5.517939 6.436764 9.538269; 1 -5.259954 5.518418 8.046965; 6 -0.793923 4.574023 11.829263; 1 -2.972104 1.221021 10.519143; 1 -3.070707 1.899013 12.150054; 1 -1.506865 1.774936 11.332499; 1 -1.062661 7.161434 10.524709; 1 -2.616757 7.602362 11.242980; 1 -2.403315 7.660562 9.490483; 1 -0.248732 3.625560 11.810453; 1 -1.085067 4.761708 12.872989; 1 -0.093427 5.363770 11.539397; 6 -0.811678 4.840773 6.974724; 6 -2.113712 4.526432 6.431237; 6 -0.189737 3.709077 7.592007; 6 -2.398682 3.237592 5.710622; 1 -2.706915 5.384182 6.114986; 6 -0.946915 2.476197 7.558625; 1 0.761571 3.798268 8.110794; 6 -1.736860 2.138846 6.324254; 1 -2.251481 1.178226 6.379582; 6 -3.845098 3.083413 5.279106; 1 -4.136757 3.780292 4.488035; 1 -4.055108 2.068541 4.932498; 1 -4.492636 3.278870 6.147315; 6 -0.398712 1.290838 8.323627; 1 0.122009 1.604325 9.234601; 1 -1.196991 0.598588 8.616458; 1 0.313737 0.724836 7.703738; 6 -0.188902 6.209497 6.935815; 1 0.371462 6.344675 6.003246; 1 -0.958026 6.988703 6.980717; 1 0.499897 6.358640 7.775406; 1 -2.580823 4.056814 2.756174;
22-9_ts	77 -1.118240 2.373939 4.281747; 6 -1.946835 0.378674 3.599343; 6 -0.499827 0.228488 3.597978; 6 0.007764 1.106531 2.605899; 6 -1.122664 1.727357 1.917782; 6 -2.316552 1.239089 2.487666; 6 -3.716643 1.492381 2.008667; 6 -0.980715 2.635461 0.730678; 6 1.428034 1.219993 2.130078; 6 0.278347 -0.758627 4.420428; 6 -2.893478 -0.536017 4.327168; 1 2.132131 0.771961 2.836391; 1 1.723970 2.262593 1.976253; 1 1.553255 0.698993 1.170006; 1 1.316664 -0.441208 4.556599; 1 0.296711 -1.748144 3.939604; 1 -0.157945 -0.887519 5.415568; 1 -2.511237 -0.818403 5.313102; 1 -3.045485 -1.465430 3.759870; 1 -3.877645 -0.080639 4.472046; 1 -3.782693 2.408035 1.413232; 1 -4.423010 1.588942 2.837888; 1 -4.069653 0.664729 1.376416; 1 -0.286715 3.458040 0.933192; 1 -1.938826 3.074498 0.438693; 1 -0.592790 2.085216 -0.138094; 6 1.448338 6.368857 2.648310; 6 0.053505 6.313477 2.618998; 1 -0.491862 7.103674 2.105389; 6 -0.680840 5.276718 3.213090; 6 0.009164 4.217457 3.863031; 6 1.428244 4.288505 3.933571; 6 2.114056 5.344416 3.321391; 1 3.200420 5.370468 3.389839; 6 2.204260 7.483842 1.964243; 1 3.166144 7.674994 2.452779; 1 1.631594 8.417995 1.967241; 1 2.417471 7.238989 0.914480; 6 -2.185084 5.340136 3.081597; 6 2.241396 3.300562 4.746162; 1 2.176892 3.539435 5.816728; 1 3.299129 3.339060 4.464384; 1 1.881473 2.277023 4.633946; 1 -2.479382 6.167348 2.425937; 1 -2.665613 5.493033 4.051714; 1 -0.180224 3.468238 5.059757; 77 -2.127771 4.075904 8.624777; 6 -3.922462 3.609817 9.875952; 6 -3.874914 5.040774 9.665299; 6 -2.747416 3.262772 10.643121; 6 -5.064311 2.693777 9.538917; 6 -2.728469 5.570615 10.365649; 6 -4.944700 5.858815 8.997852; 6 -2.031564 4.475464 10.963232; 6 -2.441459 1.897171 11.188396; 1 -5.603375 3.033544 8.649663; 1 -5.790200 2.641015 10.364745; 1 -4.717272 1.675175 9.340163; 6 -2.362472 7.022622 10.479564; 1 -4.535433 6.769549 8.549467; 1 -5.715062 6.164248 9.720256; 1 -5.440602 5.294484 8.202909; 6 -0.806334 4.571021 11.826994; 1 -2.792898 1.110082 10.514694; 1 -2.932007 1.744663 12.160324; 1 -1.367400 1.749735 11.335336; 1 -1.281558 7.160417 10.586433; 1 -2.839579 7.487176 11.354629; 1 -2.681068 7.588528 9.598585; 1 -0.200882 3.661498 11.768512; 1 -1.073835 4.717106 12.883655; 1 -0.169138 5.411323 11.533125; 6 -1.005603 4.896113 6.982051; 6 -2.306772 4.511852 6.475523; 6 -0.293730 3.790744 7.559710; 6 -2.556298 3.223394 5.727400; 1 -2.962830 5.342677 6.213007; 6 -0.978770 2.518918 7.522232; 1 0.669245 3.929005 8.045963; 6 -1.786935 2.149274 6.305310; 1 -2.261451 1.170837 6.396224; 6 -4.017820 3.029338 5.373787; 1 -4.352842 3.729540 4.598602; 1 -4.217237 2.014935 5.019634; 1 -4.641814 3.198699 6.264671; 6 -0.343841 1.356781 8.255814; 1 0.210856 1.688198 9.140390; 1 -1.096665 0.630862 8.585558; 1 0.356618 0.823428 7.595590; 6 -0.455995 6.296879 6.935307; 1 0.064320 6.474500 5.985438; 1 -1.262032 7.035040 7.014513; 1 0.253012 6.475734 7.751868; 1 -2.590457 4.409242 2.676729;
6-23_ts	6 6.652048 6.945402 1.741664; 6 6.635014 7.351558 0.354610; 6 5.382714 8.018025 0.088806; 6 4.637222 8.064107 1.310238; 6 5.432455 7.439793 2.338796; 6 7.585774 5.954452 2.372882; 1 8.563250 5.944054 1.887862; 1 7.167920 4.939524 2.295450; 1 7.753355 6.164414 3.431940; 6 7.645247 6.960471 -0.684963; 1 7.651159 7.660417 -1.525772; 1 7.422372 5.962553 -1.088026; 1 8.656544 6.933769 -0.272905; 6 4.914372 8.514289 -1.249366; 1 4.239661 9.370548 -1.149900; 1 4.369225 7.729375 -1.792556; 1 5.752433 8.824787 -1.880227; 6 3.253211 8.620064 1.481924; 1 3.086953 8.979721 2.501920; 1 2.493031 7.852557 1.279405; 1 3.062850 9.455549 0.800615; 6 4.990772 7.171139

	3.749455; 1 5.844606 7.127038 4.430931; 1 4.460543 6.210790 3.815635; 1 4.311502 7.947043 4.116020; 6 8.395174 10.517224 1.229026; 6 7.115370 11.196655 1.027996; 1 6.775966 11.575004 0.066329; 6 6.281761 11.233856 2.193706; 6 6.924202 10.543884 3.331828; 1 6.290844 10.396099 4.207130; 6 8.312158 11.018574 3.591670; 6 9.108574 10.982192 2.476962; 77 6.641697 9.215823 1.653502; 1 10.831668 13.575491 3.457367; 6 11.673538 13.319251 4.110349; 6 12.209210 11.937599 3.853087; 1 11.343215 13.443966 5.145355; 1 12.457281 14.068706 3.927633; 6 12.670542 11.017844 4.830182; 6 12.625141 11.476807 2.522045; 6 13.242364 9.893323 4.100833; 6 12.696170 11.201393 6.320361; 6 13.271439 10.243641 2.673391; 6 12.449096 12.265021 1.257230; 77 11.115989 9.851579 3.609391; 6 14.137733 8.855978 4.712787; 1 11.896706 11.866947 6.661062; 1 13.651313 11.635443 6.654397; 1 12.569950 10.246880 6.839920; 6 13.950513 9.421422 1.620451; 1 11.448660 12.707489 1.176953; 1 12.604971 11.644066 0.370019; 1 13.163800 13.099710 1.205501; 6 10.290467 8.085159 4.556941; 6 11.212293 7.289250 5.374375; 1 13.801862 8.565931 5.710530; 1 15.159708 9.254659 4.801075; 1 14.182134 7.950541 4.103642; 1 13.616896 9.690857 0.613497; 1 13.753963 8.354490 1.768564; 1 15.041041 9.560274 1.656308; 6 10.404852 7.912895 3.098482; 1 11.117830 7.375044 6.458074; 6 12.086516 6.386931 4.853414; 6 11.367882 6.943408 2.567466; 1 9.497775 8.043696 2.507085; 6 12.164610 6.232715 3.415554; 1 12.868433 5.505599 3.010819; 1 9.901771 11.709710 2.334745; 6 9.255996 10.242388 0.015678; 1 8.647621 10.001875 -0.862894; 1 9.877447 11.115774 -0.233491; 1 9.937609 9.407789 0.203743; 6 4.939249 11.908585 2.255511; 1 5.045857 12.947380 2.596962; 1 4.451409 11.928732 1.274234; 1 4.273641 11.397733 2.959704; 6 8.519382 11.789095 4.866806; 1 8.289448 11.164659 5.738333; 1 9.540166 12.148710 4.974176; 1 7.826819 12.644825 4.901018; 6 8.923694 8.230642 5.216679; 1 8.979817 8.846442 6.123392; 1 8.195106 8.675352 4.538139; 1 8.541357 7.242821 5.522727; 6 11.426403 6.717156 1.077827; 1 12.230460 6.024334 0.805613; 1 10.486465 6.293936 0.697190; 1 11.588767 7.658480 0.537842; 6 12.974236 5.530368 5.723779; 1 12.821314 4.461183 5.521990; 1 14.039730 5.733989 5.544581; 1 12.777812 5.701568 6.787705;
23	6 6.451947 6.858921 2.065085; 6 6.564443 6.972044 0.629242; 6 5.416265 7.687354 0.137214; 6 4.599386 8.041550 1.259815; 6 5.247684 7.559898 2.452178; 6 7.249675 5.947649 2.952096; 1 8.268605 5.802410 2.586350; 1 6.775588 4.956334 3.007938; 1 7.326048 6.334538 3.971359; 6 7.573511 6.274013 -0.236540; 1 7.827437 6.862626 -1.123385; 1 7.183273 5.306570 -0.583468; 1 8.501267 6.075739 0.304504; 6 5.090993 7.941560 -1.307310; 1 4.507715 8.859028 -1.435901; 1 4.500162 7.116922 -1.731407; 1 5.997156 8.037379 -1.912982; 6 3.266977 8.731045 1.198624; 1 3.059801 9.291650 2.115133; 1 2.453184 8.003212 1.070632; 1 3.210162 9.433767 0.361014; 6 4.661237 7.592429 3.835194; 1 5.439183 7.566036 4.602015; 1 4.002663 6.727736 3.999539; 1 4.064188 8.494976 4.000029; 6 8.519618 10.162960 0.938678; 6 7.286837 10.854899 0.570439; 1 6.983987 11.048775 -0.456945; 6 6.435795 11.171934 1.685845; 6 7.018695 10.691585 2.947308; 1 6.339596 10.753329 3.799396; 6 8.425822 11.232960 3.175885; 6 9.251781 10.904444 2.044009; 77 6.677691 9.075294 1.545075; 1 10.297392 13.633935 5.556644; 6 11.083145 13.010412 5.989723; 6 11.591719 11.979922 5.021676; 1 10.669745 12.544286 6.889416; 1 11.892996 13.681913 6.312053; 6 12.188600 10.742565 5.371539; 6 11.728425 12.132925 3.588573; 6 12.751949 10.141490 4.174107; 6 12.427122 10.256357 6.770012; 6 12.484431 11.002776 3.080049; 6 11.566366 13.422012 2.825798; 77 10.354529 10.300914 3.785162; 6 13.675445 8.960731 4.151997; 1 11.660642 10.617216 7.462654; 1 13.399210 10.616029 7.137866; 1 12.436843 9.166178 6.823655; 6 13.023583 10.888059 1.682900; 1 11.072808 14.192731 3.423676; 1 10.996835 13.307273 1.896795; 1 12.554567 13.813242 2.547534; 6 9.396764 8.600010 4.941346; 6 10.205758 7.992687 5.995016; 1 13.405253 8.214867 4.901474; 1 14.706960 9.281702 4.359237; 1 13.675543 8.461033 3.179028; 1 12.301476 11.241708 0.939994; 1 13.275913 9.853638 1.431758; 1 13.937940 11.488331 1.560038; 6 9.713995 8.214810 3.578575; 1 9.964778 8.260438 7.023543; 6 11.122462 7.011978 5.760276; 6 10.757010 7.216973 3.343582; 1 8.918720 8.254230 2.839342; 6 11.394767 6.629730 4.396319; 1 12.140838 5.857672 4.212110; 1 9.876006 11.684860 1.600085; 6 9.372616 9.612427 -0.184170; 1 8.760707 9.269495 -1.026401; 1 10.063518 10.382201 -0.563614; 1 9.984731 8.772153 0.153951; 6 5.126954 11.901701 1.556927; 1 5.277909 12.983840 1.674670; 1 4.661220 11.735607 0.578078; 1 4.421145 11.583849 2.332118; 6 8.302913 12.637408 3.759423; 1 7.977134 12.598177 4.806707; 1 9.235629 13.194981 3.721195; 1 7.547648 13.220145 3.208472; 6 7.979819 8.885581 5.417186; 1 7.949780 9.737659 6.106671; 1 7.316088 9.088731 4.582429; 1 7.587199 8.012605 5.961727; 6 11.105985 6.837962 1.928290; 1 11.777606 5.973216 1.894990; 1 10.214155 6.601191 1.337081; 1 11.602053 7.673558 1.416648; 6 11.845003 6.298723 6.878582; 1 11.657752 5.216918 6.843930; 1 12.934461 6.428676 6.810510; 1 11.525776 6.663026 7.860666;
23-24_ts	6 6.476367 6.802355 2.066515; 6 6.593015 6.914241 0.632173; 6 5.446961 7.630125 0.136948; 6 4.624074 7.980718 1.256473; 6 5.271624 7.505578 2.451113; 6 7.250640 5.868877 2.950744; 1 8.272761 5.714786 2.597978; 1 6.761338 4.883749 2.984001; 1 7.316883 6.237676 3.977397; 6 7.597609 6.209776 -0.234884; 1 7.918065 6.829844 -1.078028; 1 7.169068 5.286431 -0.650111; 1 8.490542 5.928314 0.327538; 6 5.128644 7.882656 -1.309529; 1 4.553721 8.804677 -1.443693; 1 4.532993 7.061630 -1.734105; 1 6.038403 7.969242 -1.911350; 6 3.287882 8.661960 1.188929; 1 3.076214 9.229596 2.100022; 1 2.479015 7.927627 1.066828; 1 3.226919 9.356409 0.344768; 6 4.684936 7.539882 3.834090; 1 5.464546 7.528806 4.600139; 1 4.036067 6.669407 4.007258; 1 4.078649 8.437313 3.993099; 6 8.485116 10.182294 0.872204; 6 7.216574 10.830576 0.560705; 1 6.863142 11.012524 -0.453023; 6 6.397274 11.116407 1.708431; 6 7.044306 10.661146 2.944581; 1 6.392632 10.701020 3.820086; 6 8.443251 11.253680 3.121161; 6 9.247187 10.924274 1.959322; 77 6.695853 9.029262 1.546739; 1 10.183078 13.491023 5.568140; 6 10.977714 12.891754 6.018629; 6 11.521365 11.867226 5.064344; 1 10.565064 12.424039 6.917770; 1 11.768758 13.584911 6.342313; 6 12.077589 10.615318 5.408868; 6 11.725794 12.054495 3.628281; 6 12.703794 10.041383 4.217483; 6 12.211212 10.052888 6.792249; 6 12.525509 10.932190 3.140512; 6 11.653615 13.373033 2.902904; 77 10.337877 10.360561 3.698504; 6 13.586731 8.829114 4.219183; 1 11.484224 10.495315 7.480108; 1 13.213545 10.251366 7.200142; 1 12.062024 8.969566 6.805753; 6 13.139481 10.847124 1.772987; 1 11.062717 14.113211 3.448287; 1 11.227358 13.279452 1.898486; 1 12.664579 13.788994 2.787909; 6 9.269294 8.250734 5.130364; 6 10.047112 7.523353 6.073233; 1 13.230361 8.063498 4.911006; 1 14.607547 9.101597 4.525858; 1 13.654943 8.370762 3.227882; 1 12.460667 11.226025 1.002578; 1 13.397589 9.816982 1.509141; 1 14.064065 11.441672 1.715644; 6 9.566927 8.100689 3.749672; 1 9.815123 7.632741 7.131338; 6 11.006571 6.608449 5.683211; 6 10.593654 7.183203 3.345578; 1 8.812267 8.384130 3.015443; 6 11.267936 6.445732 4.298247; 1 12.021056 5.724652 3.984918; 1 9.834805 11.724179 1.494024; 6 9.303593 9.681515 -0.299429; 1 8.668377 9.284984 -1.100228; 1 9.908066 10.498248 -0.725607; 1 10.000001 8.896246 0.004273; 6 5.062523 11.804827 1.627168; 1 5.185975 12.890630 1.742725; 1 4.567206 11.627215 0.665009; 1 4.394561 11.465433 2.426495; 6 8.279947 12.682974 3.635346; 1 7.949450 12.688022 4.682472; 1 9.202746 13.257534 3.574779; 1 7.518198 13.226890 3.054250; 6 7.994584 8.875335 5.656130; 1 8.201266 9.713401 6.330242; 1 7.367315 9.235038 4.845832; 1 7.428078 8.128367 6.232006; 6 10.929964

	7.015147 1.886213; 1 11.462910 6.075744 1.702323; 1 10.031589 7.036961 1.264363; 1 11.566232 7.840226 1.543935; 6 11.759260 5.773803 6.692042; 1 11.646969 4.702499 6.481795; 1 12.835922 5.989210 6.667457; 1 11.405016 5.957447 7.711288;
24	6 6.931909 6.939793 1.821628; 6 7.013474 7.518854 0.499633; 6 5.717013 8.060745 0.176703; 6 4.836837 7.810163 1.285759; 6 5.585052 7.135446 2.306978; 6 7.946891 6.049038 2.468834; 1 8.952174 6.301466 2.137369; 1 7.750172 4.996299 2.215164; 1 7.931274 6.141425 3.557677; 6 8.176126 7.420874 -0.443074; 1 8.155279 8.225368 -1.184307; 1 8.161993 6.466584 -0.989244; 1 9.122709 7.496717 0.093394; 6 5.318221 8.664921 -1.139691; 1 4.517807 9.402428 -1.022763; 1 4.952725 7.892468 -1.831228; 1 6.161330 9.164233 -1.625258; 6 3.373910 8.144335 1.338310; 1 3.032745 8.308778 2.364686; 1 2.770040 7.326208 0.921143; 1 3.141736 9.045066 0.761136; 6 5.041711 6.602238 3.601565; 1 5.808744 6.596104 4.381532; 1 4.681236 5.570342 3.484981; 1 4.201569 7.201930 3.966242; 6 7.566965 11.155868 1.504378; 6 6.191895 11.282718 1.923860; 1 5.404972 11.619789 1.251142; 6 5.865453 10.780281 3.229054; 6 7.001538 10.200117 3.918176; 1 6.778605 9.642665 4.828024; 6 8.331600 10.871018 3.864096; 6 8.640250 11.353141 2.517206; 77 6.627650 9.171305 2.004498; 1 10.820925 6.146729 1.255131; 6 11.564049 6.936460 1.108673; 6 11.450527 8.042113 2.120951; 1 11.461899 7.305728 0.083741; 1 12.550402 6.454664 1.181354; 6 11.785392 9.393677 1.891501; 6 11.202349 7.840340 3.542932; 6 11.686493 10.089770 3.170766; 6 12.248096 10.011744 0.603213; 6 11.387560 9.089222 4.198222; 6 11.053036 6.511219 4.229092; 77 9.623817 9.568495 2.896722; 6 12.216735 11.474253 3.420992; 1 11.872621 9.465672 -0.268255; 1 13.347038 10.023156 0.532495; 1 11.908151 11.048193 0.510270; 6 11.464065 9.301473 5.683163; 1 10.569291 5.766523 3.590797; 1 10.457224 6.593703 5.144118; 1 12.032143 6.095863 4.515542; 1 11.913768 12.171688 2.632572; 1 13.316244 11.473125 3.454754; 1 11.861647 11.877737 4.373675; 1 10.799579 8.617369 6.220645; 1 11.183342 10.320302 5.962895; 1 12.486630 9.128248 6.051289; 1 9.259846 12.245339 2.387128; 6 8.722634 11.643224 5.108110; 1 8.752533 10.990688 5.990266; 1 9.707966 12.107971 5.000339; 1 7.999037 12.446785 5.318608; 6 4.468006 10.817572 3.786894; 1 4.290001 9.970640 4.458665; 1 4.319414 11.735661 4.371669; 1 3.709398 10.800514 2.995790; 6 7.920020 11.480985 0.070708; 1 8.176489 12.546917 -0.027526; 1 8.792943 10.903523 -0.248799; 1 7.092792 11.271799 -0.616493;
24-25_ts	77 2.567985 4.549300 2.578901; 6 0.352120 4.809122 1.978770; 6 1.074525 5.905482 1.395920; 6 1.651596 6.678747 2.459470; 6 1.305670 6.041827 3.708182; 6 0.493931 4.881296 3.413156; 6 4.034390 3.596935 3.881317; 6 4.597696 4.726987 3.172328; 6 4.577009 4.549061 1.740455; 6 4.371727 3.250137 1.184502; 77 2.627712 1.500930 2.372249; 6 4.578560 2.069890 2.005757; 6 4.348256 3.110125 -0.318583; 6 4.305945 2.199389 3.436699; 6 5.031968 1.336240 4.449611; 6 2.659534 -0.682802 2.302410; 6 1.691401 -0.321708 3.330799; 6 0.565767 0.269458 2.679765; 6 0.815253 0.253582 1.247134; 6 2.076304 -0.347265 1.015997; 6 2.669488 -0.681725 -0.322607; 6 -0.173824 0.596024 0.167177; 6 -0.765568 0.519320 3.330214; 6 1.776199 -0.697103 4.783237; 6 3.854298 -1.577124 2.486153; 6 5.177152 5.948730 3.832755; 6 -0.234248 4.049050 4.425880; 6 1.589344 6.578601 5.081862; 6 2.390606 7.975858 2.297324; 6 1.119672 6.242446 -0.066890; 6 -0.577054 3.909103 1.226645; 1 4.806059 5.388897 1.086823; 1 5.223424 1.281977 1.614503; 1 3.871019 3.727767 4.951359; 1 5.092705 6.838077 3.197401; 1 6.244976 5.792423 4.040563; 1 4.685713 6.154970 4.789706; 1 3.809570 3.937468 -0.792070; 1 3.876133 2.172787 -0.621255; 1 5.376896 3.118242 -0.710855; 1 -0.170269 3.621228 0.253954; 1 -1.538652 4.414589 1.050649; 1 -0.773578 2.996831 1.786860; 1 1.079781 5.340612 -0.685172; 1 2.033062 6.785663 -0.329143; 1 0.268337 6.876141 -0.355166; 1 3.001370 7.983868 1.388440; 1 3.052780 8.174976 3.144753; 1 1.690418 8.820321 2.225981; 1 5.223372 0.330630 4.064655; 1 4.449156 1.238822 5.374471; 1 6.003880 1.777827 4.721601; 1 2.496823 7.189583 5.100105; 1 1.717343 5.771177 5.809116; 1 0.761886 7.210806 5.433686; 1 -0.285492 3.005759 4.114662; 1 -1.260365 4.415921 4.577051; 1 0.270870 4.074791 5.396007; 1 2.811294 -0.705244 5.136974; 1 1.359825 -1.700523 4.960283; 1 1.222222 0.006650 5.412233; 1 4.686395 -1.291439 1.832743; 1 3.599648 -2.620940 2.250186; 1 4.220058 -1.558685 3.516426; 1 2.318437 0.002000 -1.102593; 1 2.396428 -1.700831 -0.637018; 1 3.763280 -0.633051 -0.305743; 1 0.289306 1.148155 -0.658229; 1 -1.003417 1.198266 0.542791; 1 -0.611009 -0.317868 -0.261951; 1 -1.329857 1.325812 2.852413; 1 -0.659566 0.771769 4.389542; 1 -1.398775 -0.380135 3.275771;
25	77 2.377428 4.409111 2.644126; 6 0.106051 4.864895 2.142982; 6 0.895781 5.874101 1.509932; 6 1.587481 6.606154 2.536159; 6 1.244036 6.024232 3.807716; 6 0.330457 4.935755 3.571441; 6 4.021521 3.772791 3.879983; 6 4.442248 4.678166 2.833198; 6 4.209915 4.106163 1.527480; 6 4.455588 2.654007 1.315290; 77 2.801373 1.569532 2.265471; 6 4.854299 1.887817 2.470878; 6 4.939098 2.258000 -0.061069; 6 4.268627 2.313450 3.718241; 6 4.541048 1.538875 4.987897; 6 2.756910 -0.748986 2.057912; 6 1.868944 -0.402423 3.136578; 6 0.809281 0.397495 2.603199; 6 1.029978 0.538510 1.173143; 6 2.224102 -0.182763 0.846740; 6 2.714985 -0.437723 -0.548909; 6 0.052849 1.035443 0.144944; 6 -0.451831 0.711195 3.352770; 6 1.950693 -0.906072 4.548639; 6 3.941668 -1.668742 2.153197; 6 5.118060 5.999675 3.077079; 6 -0.460021 4.236687 4.641033; 6 1.639068 6.548750 5.159212; 6 2.370660 7.868170 2.315925; 6 0.896139 6.206460 0.045140; 6 -1.003401 4.126250 1.457962; 1 4.356788 4.748921 0.659088; 1 5.475627 0.997432 2.392549; 1 4.025847 4.148023 4.903946; 1 4.958101 6.699357 2.250295; 1 6.202586 5.845379 3.171539; 1 4.773414 6.470298 4.005175; 1 4.221380 2.558875 -0.833452; 1 5.091236 1.177974 -0.144773; 1 5.894804 2.752032 -0.293709; 1 -0.731796 3.821278 0.444780; 1 -1.889220 4.774633 1.379277; 1 -1.306802 3.236811 2.010686; 1 0.696087 5.321116 -0.565639; 1 1.860345 6.614771 -0.274747; 1 0.127734 6.955808 -0.197113; 1 2.952980 7.832090 1.389077; 1 3.063002 8.071988 3.136912; 1 1.694313 8.731701 2.239097; 1 4.709091 0.475987 4.787880; 1 3.702583 1.621473 5.689242; 1 5.434579 1.929441 5.498425; 1 2.606398 7.060360 5.131505; 1 1.713248 5.741311 5.894281; 1 0.899175 7.269583 5.536244; 1 -0.853734 3.279868 4.294974; 1 -1.316378 4.848242 4.964351; 1 0.151217 4.037623 5.527136; 1 2.977011 -1.150540 4.837242; 1 1.350878 -1.819512 4.674044; 1 1.574318 -0.167104 5.262746; 1 4.692702 -1.444239 1.389075; 1 3.640769 -2.717305 2.015390; 1 4.433227 -1.598795 3.128733; 1 2.597729 0.444279 -1.185941; 1 2.146657 -1.256402 -1.014626; 1 3.770179 -0.725461 -0.568533; 1 0.516564 1.732439 -0.560947; 1 -0.793561 1.544779 0.605518; 1 -0.352208 0.196024 -0.439311; 1 -1.025672 1.505488 2.873972; 1 -0.246799 1.020151 4.381624; 1 -1.102534 -0.175154 3.398799;
25-26_ts	6 6.470086 6.808344 1.184315; 6 7.067046 7.584967 0.117785; 6 6.061169 8.503229 -0.348873; 6 4.841054 8.254238 0.382904; 6 5.088860 7.193751 1.311931; 6 7.112540 5.647163 1.888625; 1 8.195541 5.770843 1.955250; 1 6.916789 4.706690 1.353895; 1 6.729816 5.530228 2.907559; 6 8.391230 7.336080 -0.538389; 1 8.833814 8.262449 -0.915267; 1 8.289348 6.643935 -1.388180; 1 9.103216 6.894219 0.161406; 6 6.203356 9.407136 -1.539600; 1 5.523518 10.262196 -1.487813; 1 5.977786 8.863365 -2.468089; 1 7.221078 9.799173 -1.625351; 6 3.518893 8.926870 0.144927; 1 2.922040 8.980871 1.061218; 1 2.924148 8.380420 -0.601120; 1 3.647086 9.948159 -0.226346; 6 4.081088 6.579403 2.240018; 1 4.556486 6.169804 3.136670; 1 3.539379 5.755555 1.753325; 1 3.334597 7.310683 2.567491; 6 7.095905 11.121108 1.948602; 6 6.029699 10.761076 2.861778; 1 5.014011 11.149554 2.813026; 6

	6.398735 9.738588 3.809239; 6 7.739684 9.192453 3.621990; 1 7.919399 8.332968 4.268394; 6 8.629591 11.117173 3.985558; 6 8.443546 11.432938 2.591318; 77 6.539294 8.995861 1.818464; 1 10.102254 6.271940 3.088686; 6 10.963620 6.609612 2.502882; 6 11.310867 8.039318 2.798075; 1 10.735002 6.453469 1.446067; 1 11.804338 5.948141 2.756685; 6 11.652474 9.049131 1.868129; 6 11.490007 8.551336 4.147998; 6 11.973964 10.243447 2.627170; 6 11.755115 8.930969 0.375101; 6 11.948783 9.898671 4.040435; 6 11.371071 7.735770 5.404036; 77 9.862342 9.942186 3.215376; 6 12.529049 11.511681 2.042035; 1 11.204330 8.067145 -0.004742; 1 12.803327 8.817352 0.060003; 1 11.359836 9.819045 -0.128522; 6 12.417066 10.775859 5.166677; 1 10.524187 7.042391 5.356708; 1 11.223640 8.372205 6.282171; 1 12.273458 7.131926 5.583892; 1 12.047085 11.754451 1.089592; 1 13.609714 11.426970 1.854987; 1 12.376787 12.362992 2.712933; 1 11.901897 10.539390 6.102823; 1 12.241801 11.834195 4.951070; 1 13.495511 10.648624 5.339904; 1 8.845703 12.393852 2.255433; 6 8.248176 11.754714 5.274247; 1 8.502566 11.131189 6.137209; 1 8.813276 12.695164 5.356206; 1 7.183136 12.016976 5.309218; 6 5.450695 9.252312 4.875754; 1 5.617434 8.192918 5.098729; 1 5.596803 9.813298 5.809573; 1 4.405672 9.380342 4.569985; 6 6.760289 12.018315 0.774402; 1 6.797281 13.079031 1.066789; 1 7.480815 11.884350 -0.040875; 1 5.759907 11.814266 0.378974;
26	6 -1.858378 -2.550782 -1.433437; 6 -1.345157 -1.666257 -2.462209; 6 -2.396627 -0.732456 -2.775267; 6 -3.556210 -1.062995 -1.983495; 6 -3.226000 -2.191745 -1.167041; 6 -1.157605 -3.765921 -0.895002; 1 -0.072022 -3.640257 -0.908271; 1 -1.394751 -4.656579 -1.494688; 1 -1.452541 -3.979702 0.137680; 6 -0.049436 -1.820031 -3.205103; 1 0.325541 -0.855099 -3.559463; 1 -0.164519 -2.475405 -4.082033; 1 0.725889 -2.256110 -2.568646; 6 -2.350763 0.289267 -3.875330; 1 -3.002132 1.142716 -3.665900; 1 -2.679158 -0.148827 -4.828753; 1 -1.337215 0.674860 -4.021734; 6 -4.901339 -0.399476 -2.071578; 1 -5.433104 -0.435533 -1.115082; 1 -5.541562 -0.889643 -2.819744; 1 -4.813790 0.652521 -2.360261; 6 -4.157656 -2.905056 -0.229566; 1 -3.615696 -3.374091 0.597564; 1 -4.714586 -3.699988 -0.746733; 1 -4.894441 -2.221430 0.204895; 6 -1.260197 1.665718 -0.339733; 6 -2.003320 1.144562 0.793545; 1 -2.999819 1.505706 1.043057; 6 -1.451559 0.008930 1.475273; 6 -0.191903 -0.441556 0.929225; 1 0.072693 -1.448022 1.265381; 6 0.804378 2.439864 1.001606; 6 0.211385 2.036548 -0.235166; 77 -1.720745 -0.435450 -0.615205; 1 1.604464 -2.903714 0.638775; 6 2.550442 -2.608433 0.172932; 6 2.962996 -1.223762 0.572886; 1 2.429020 -2.697461 -0.910114; 1 3.305522 -3.345007 0.482697; 6 3.500867 -0.220259 -0.262862; 6 2.955853 -0.728237 1.941807; 6 3.840822 0.915830 0.574438; 6 3.742458 -0.298477 -1.742454; 6 3.564970 0.568462 1.944525; 6 2.582708 -1.544109 3.146455; 77 1.529996 0.742757 0.815236; 6 4.571115 2.141876 0.099599; 1 3.117446 -1.061581 -2.214102; 1 4.791442 -0.545256 -1.962123; 1 3.522066 0.654086 -2.235286; 6 3.929555 1.376452 3.157978; 1 1.672895 -2.128245 2.970483; 1 2.399960 -0.909117 4.018348; 1 3.379255 -2.254014 3.412914; 1 4.231839 2.453212 -0.893668; 1 5.652869 1.956440 0.031484; 1 4.427923 2.987831 0.778810; 1 3.228813 1.204456 3.980529; 1 3.930845 2.450025 2.944787; 1 4.934542 1.115559 3.519713; 1 0.576062 2.516269 -1.153118; 6 1.001498 3.721881 1.720666; 1 1.683479 3.632753 2.572006; 1 1.377346 4.495999 1.033508; 1 0.031136 4.082772 2.091791; 6 -2.177414 -0.660288 2.614713; 1 -1.992469 -1.740210 2.616819; 1 -1.822951 -0.264580 3.577067; 1 -3.260483 -0.495345 2.562778; 6 -2.008409 2.676232 -1.201902; 1 -1.907458 3.695632 -0.797753; 1 -1.610503 2.696807 -2.223120; 1 -3.076506 2.440048 -1.260955;
6-27_ts	6 6.742961 6.723461 2.609052; 6 6.928022 6.805878 1.181957; 6 5.638447 7.039364 0.560003; 6 4.661493 7.138137 1.592195; 6 5.342685 7.004595 2.859391; 6 7.756784 6.270649 3.620531; 1 8.772976 6.543712 3.320307; 1 7.732083 5.177988 3.748686; 1 7.574418 6.720835 4.600894; 6 8.198813 6.486387 0.448164; 1 8.273308 7.036064 -0.494667; 1 8.250642 5.414556 0.210518; 1 9.080081 6.735210 1.046327; 6 5.378067 7.095586 -0.918002; 1 4.525976 7.741103 -1.153647; 1 5.154402 6.097216 -1.320485; 1 6.244521 7.479137 -1.464868; 6 3.186180 7.342638 1.403701; 1 2.743849 7.888129 2.243065; 1 2.657274 6.382008 1.325899; 1 2.970269 7.908208 0.491587; 6 4.674944 6.946935 4.204550; 1 5.355956 7.257679 5.002039; 1 4.341124 5.924849 4.432092; 1 3.795801 7.597255 4.247131; 6 7.916458 10.329528 1.468984; 6 6.592465 10.756373 1.076909; 1 6.314608 10.937983 0.042036; 6 5.635694 10.853038 2.134589; 6 6.188477 10.468446 3.425557; 1 5.469906 10.377797 4.239086; 6 7.579205 10.792798 3.819650; 6 8.470828 10.808959 2.777272; 1 9.535351 10.701890 2.934122; 6 8.927503 10.060416 0.377057; 1 9.666772 9.313222 0.687999; 1 9.475648 10.981315 0.132427; 1 8.446228 9.696708 -0.536782; 6 4.226020 11.340259 1.941285; 1 3.858654 11.114304 0.934359; 1 4.176097 12.428130 2.082616; 1 3.547290 10.879243 2.667183; 6 7.880098 10.737035 5.290256; 1 8.936505 10.887227 5.504115; 1 7.574383 9.749925 5.672736; 1 7.308312 11.487296 5.846715; 77 6.315028 8.823985 1.970021; 1 11.403194 14.501946 0.872022; 6 11.416303 15.069907 1.807586; 6 11.109846 14.194443 2.987620; 1 10.685820 15.878355 1.713660; 1 12.411449 15.529316 1.904596; 6 10.604785 14.653093 4.282499; 6 11.364281 12.805728 3.112128; 6 10.730553 13.548371 5.234820; 6 10.511803 16.087289 4.715831; 6 11.161301 12.423716 4.517038; 6 11.996944 11.919442 2.076751; 77 9.095225 13.302778 3.445902; 6 10.482456 13.670947 6.709321; 1 10.370471 16.758301 3.867440; 1 11.440206 16.380821 5.228344; 1 9.680290 16.249497 5.407688; 6 11.527423 11.076675 5.073340; 1 11.817267 12.294974 1.065086; 1 11.616334 10.892812 2.117537; 1 13.086745 11.858543 2.218128; 6 7.631126 14.555880 2.480883; 6 7.088217 14.028516 3.743720; 1 9.586606 14.267796 6.911443; 1 11.323117 14.167215 7.217467; 1 10.344910 12.694521 7.184659; 1 11.116304 10.248321 4.484703; 1 11.185476 10.952669 6.105184; 1 12.619470 10.947834 5.075232; 6 7.902280 16.000707 2.469150; 6 7.135303 14.064174 1.129657; 1 6.402582 13.183110 3.673570; 6 6.792121 14.941968 4.847260; 6 7.612268 16.828858 3.508864; 1 8.265886 16.430742 1.534841; 1 7.859169 14.310987 0.342205; 1 6.180433 14.545359 0.860893; 1 6.988822 12.986756 1.116164; 6 6.151699 14.378319 6.090399; 6 7.055705 16.273148 4.724423; 6 7.835701 18.320136 3.433873; 1 6.821889 13.670830 6.597116; 1 5.234059 13.823275 5.848345; 1 5.890388 15.165758 6.805712; 1 6.820540 16.950603 5.544757; 1 8.202293 18.623198 2.447171; 1 8.567418 18.658310 4.181518; 1 6.907316 18.873091 3.632179;
27	6 7.089709 6.952891 2.115296; 6 6.911123 7.201880 0.703552; 6 5.503461 7.407818 0.444911; 6 4.812045 7.338544 1.691880; 6 5.788976 7.097914 2.730462; 6 8.342979 6.455280 2.776892; 1 9.238961 6.864303 2.299930; 1 8.412489 5.357983 2.724517; 1 8.379733 6.735988 3.833484; 6 7.976982 7.055391 -0.344671; 1 7.783467 7.692667 -1.212743; 1 8.033534 6.017555 -0.702734; 1 8.962814 7.322881 0.047085; 6 4.878557 7.597916 -0.907644; 1 3.967287 8.202013 -0.850948; 1 4.604004 6.633419 -1.359036; 1 5.563004 8.096879 -1.600438; 6 3.328475 7.454381 1.892837; 1 3.087087 7.870376 2.876002; 1 2.837943 6.472547 1.825837; 1 2.867558 8.101134 1.139139; 6 5.473706 6.833161 4.176157; 1 6.319853 7.082996 4.822575; 1 5.233102 5.772648 4.337572; 1 4.614779 7.420125 4.516224; 6 7.787696 10.741154 1.218254; 6 6.390286 11.094513 1.161688; 1 5.886294 11.349740 0.232557; 6 5.671090 11.011717 2.401320; 6 6.502708 10.568330 3.499564; 1 5.959524 10.320242 4.412564; 6 7.959009 10.920273 3.694662; 6 8.631718 11.063990 2.424743; 1 9.619657 10.612230 2.322741; 6 8.538322 10.653617 -0.093924; 1 9.358378 9.928192 -0.037521; 1 8.984053 11.627039 -0.347276; 1 7.882742 10.354966 -0.919179; 6 4.205172 11.340113

	2.522877; 1 3.675329 11.144768 1.583721; 1 4.055186 12.398803 2.776693; 1 3.735020 10.741770 3.311277; 6 8.523627 10.089538 4.836011; 1 9.609577 10.115884 4.877875; 1 8.220075 9.039168 4.712757; 1 8.138581 10.429277 5.806252; 77 6.410236 9.078885 1.860716; 1 11.596683 12.030878 1.290947; 6 11.824437 13.032258 1.668309; 6 11.185746 13.276887 3.006225; 1 11.480189 13.752320 0.919808; 1 12.920201 13.118605 1.729835; 6 10.791178 14.530763 3.542725; 6 11.068962 12.296615 4.071152; 6 10.481111 14.356411 4.953777; 6 10.963500 15.848887 2.846728; 6 10.668208 12.998753 5.282307; 6 11.778932 10.966983 4.034089; 77 8.974688 12.905909 3.491994; 6 10.141508 15.443677 5.932733; 1 10.791178 15.764695 1.769274; 1 11.990547 16.216945 2.986351; 1 10.285400 16.611670 3.233042; 6 10.593402 12.423469 6.667513; 1 11.457272 10.329413 3.203997; 1 11.643134 10.401477 4.959842; 1 12.859216 11.129278 3.913445; 6 7.513924 14.545684 2.541120; 6 6.903346 13.770407 3.585442; 1 10.278450 16.434973 5.491958; 1 10.788039 15.386654 6.817684; 1 9.101846 15.382430 6.276883; 1 10.308582 11.368468 6.661317; 1 9.862095 12.958687 7.281529; 1 11.565640 12.495764 7.178297; 6 7.790035 15.934054 2.852993; 6 7.314843 14.282548 1.060205; 1 6.287496 12.932084 3.301637; 6 6.562155 14.387612 4.854909; 6 7.445112 16.516715 4.042075; 1 8.199261 16.555182 2.058470; 1 8.174191 14.650478 0.487330; 1 6.426622 14.821264 0.694603; 1 7.189631 13.227123 0.838419; 6 5.867653 13.547146 5.895081; 6 6.833404 15.711863 5.063113; 6 7.656606 17.989356 4.298606; 1 6.498733 12.700578 6.192531; 1 4.932791 13.122545 5.505194; 1 5.626214 14.130095 6.790132; 1 6.551874 16.182381 6.004051; 1 8.072269 18.496297 3.421539; 1 8.341732 18.157168 5.140935; 1 6.712457 18.486139 4.558681;
27-9_ts	6 7.064713 6.944853 2.087764; 6 6.866307 7.209086 0.680717; 6 5.457656 7.434937 0.447239; 6 4.786729 7.366422 1.705035; 6 5.777128 7.101672 2.725323; 6 8.323189 6.427543 2.723859; 1 9.215187 6.836535 2.239459; 1 8.383234 5.330526 2.655441; 1 8.377727 6.693799 3.783430; 6 7.912138 7.053956 -0.386276; 1 7.712823 7.698801 -1.247488; 1 7.948406 6.017496 -0.751058; 1 8.908140 7.305480 -0.010054; 6 4.812759 7.647297 -0.892546; 1 3.913165 8.266379 -0.814426; 1 4.513818 6.691853 -1.347794; 1 5.493445 8.141092 -1.592751; 6 3.308441 7.500517 1.931994; 1 3.089216 7.912565 2.922049; 1 2.803398 6.525912 1.866531; 1 2.843653 8.158731 1.190678; 6 5.480988 6.827397 4.173302; 1 6.341956 7.055396 4.808044; 1 5.223900 5.769659 4.327822; 1 4.638436 7.425919 4.533644; 6 7.830109 10.717169 1.242529; 6 6.440938 11.102011 1.178385; 1 5.948720 11.375369 0.247953; 6 5.711499 11.024801 2.414053; 6 6.529021 10.560524 3.514639; 1 5.974793 10.314689 4.421817; 6 7.990357 10.890388 3.723786; 6 8.676003 11.017043 2.454917; 1 9.651052 10.534390 2.359658; 6 8.586426 10.619725 -0.066066; 1 9.389516 9.875632 -0.008469; 1 9.056864 11.583776 -0.310907; 1 7.929854 10.340844 -0.897482; 6 4.250260 11.377556 2.527784; 1 3.724169 11.197827 1.583387; 1 4.114839 12.436590 2.788884; 1 3.764440 10.780486 3.307673; 6 8.536198 10.037644 4.858449; 1 9.622439 10.060137 4.913028; 1 8.231386 8.989577 4.717439; 1 8.142380 10.363622 5.830455; 77 6.413224 9.082830 1.863381; 1 11.651819 11.981191 1.338769; 6 11.867055 12.990430 1.702057; 6 11.213787 13.249820 3.029937; 1 11.522892 13.695412 0.939284; 1 12.961275 13.088288 1.772803; 6 10.792765 14.504788 3.541417; 6 11.091273 12.280844 4.110333; 6 10.473689 14.347802 4.956834; 6 10.931984 15.817232 2.828553; 6 10.679017 13.002481 5.312304; 6 11.815629 10.958988 4.104512; 77 9.030274 12.845853 3.514327; 6 10.126451 15.460046 5.902652; 1 10.808401 15.706101 1.746690; 1 11.931569 16.242096 3.003022; 1 10.200792 16.551528 3.172929; 6 10.606690 12.433472 6.699385; 1 11.534433 10.322353 3.259401; 1 11.643746 10.390197 5.022108; 1 12.898833 11.131328 4.031631; 6 7.469309 14.640852 2.496716; 6 6.922197 13.813148 3.519809; 1 9.704083 16.318483 5.377097; 1 11.019311 15.811353 6.440524; 1 9.394836 15.144729 6.653594; 1 10.255627 11.398168 6.700823; 1 9.929299 13.013521 7.334068; 1 11.595373 12.443731 7.182519; 6 7.694546 16.019420 2.819006; 6 7.404097 14.321358 1.017603; 1 6.386412 12.925383 3.226444; 6 6.577317 14.376428 4.803860; 6 7.341785 16.562618 4.032976; 1 8.072023 16.672570 2.034669; 1 8.288172 14.709215 0.498938; 1 6.525678 14.808664 0.566570; 1 7.341333 13.253991 0.826178; 6 5.944271 13.486315 5.842475; 6 6.791258 15.712388 5.039180; 6 7.487998 18.040049 4.310093; 1 6.618802 12.662998 6.105168; 1 5.018806 13.031172 5.466285; 1 5.700846 14.042326 6.753835; 1 6.513382 16.143909 5.999258; 1 7.884507 18.576169 3.441898; 1 8.160614 18.227430 5.157913; 1 6.521102 18.489398 4.571656;
7-33_ts	6 6.860135 6.752854 1.208600; 6 7.123945 7.541697 0.042574; 6 5.907036 8.236744 -0.314482; 6 4.877031 7.838804 0.602920; 6 5.471644 6.967976 1.579134; 6 7.724253 5.677292 1.802169; 1 8.776817 5.801834 1.545685; 1 7.407078 4.692879 1.428568; 1 7.650368 5.649937 2.893160; 6 8.359592 7.531425 -0.812365; 1 8.724787 8.542659 -1.020285; 1 8.144020 7.055504 -1.778989; 1 9.173262 6.973767 -0.346579; 6 5.723311 9.080129 -1.544112; 1 4.933534 9.826110 -1.410815; 1 5.446202 8.460212 -2.408825; 1 6.642620 9.612316 -1.805635; 6 3.430332 8.233805 0.526848; 1 2.954841 8.224207 1.511968; 1 2.869399 7.539236 -0.114000; 1 3.306131 9.236810 0.106161; 6 4.748798 6.230492 2.670618; 1 5.396025 6.065140 3.537398; 1 4.405761 5.245637 2.323293; 1 3.867474 6.780455 3.015105; 6 7.642239 10.907880 1.861563; 6 6.200121 11.046637 2.034525; 1 5.551313 11.580769 1.343689; 6 5.670433 10.315644 3.154384; 6 6.738589 9.557973 3.852277; 1 6.389704 8.870012 4.622776; 6 7.854959 10.471820 4.227632; 6 8.379519 11.156472 3.157324; 77 6.514175 8.965658 1.782192; 1 9.709147 7.800408 1.710230; 6 10.794877 7.692585 1.757051; 6 11.342943 8.418361 2.944317; 1 11.209468 8.085125 0.824044; 1 11.031965 6.618170 1.795983; 6 12.467216 9.269404 2.967545; 6 10.835290 8.291274 4.309236; 6 12.643167 9.754754 4.337172; 6 13.379961 9.587289 1.822051; 6 11.663343 9.060073 5.169790; 6 9.720714 7.371300 4.716868; 77 10.719392 10.574139 3.766130; 6 13.848043 10.490408 4.849263; 1 12.869902 9.504224 0.857336; 1 14.232697 8.893552 1.807568; 1 13.781147 10.600202 1.903218; 6 11.624477 9.096844 6.671105; 1 8.864896 7.457910 4.040459; 1 9.370723 7.589301 5.730426; 1 10.053476 6.322910 4.704777; 6 12.387631 13.108131 2.949721; 6 12.459219 13.809471 4.170346; 1 14.208710 11.224597 4.125018; 1 14.669309 9.790429 5.064381; 1 13.619035 11.031943 5.771326; 1 10.625972 8.872031 7.057709; 1 11.912381 10.080518 7.052178; 1 12.318549 8.358736 7.098621; 6 13.473693 13.237643 2.072573; 1 11.629550 13.722069 4.868021; 6 13.560531 14.592031 4.511684; 6 14.604947 14.008945 2.388786; 1 13.429628 12.742015 1.103601; 6 14.637408 14.677408 3.612074; 1 15.506664 15.280199 3.871432; 1 8.748911 12.166740 3.307410; 6 11.170533 12.303637 2.575448; 1 11.272441 11.967274 1.534740; 1 10.488999 11.723971 4.849818; 1 10.309376 12.984193 2.597569; 6 15.752813 14.118052 1.409171; 1 15.427246 14.556468 0.456778; 1 16.558285 14.745578 1.805040; 1 16.180770 13.134289 1.176489; 6 13.604428 15.345640 5.822965; 1 12.740525 15.104024 6.450890; 1 14.511060 15.108384 6.394151; 1 13.604684 16.432055 5.662342; 6 7.937523 10.843184 5.685288; 1 8.253871 9.989048 6.296170; 1 8.634360 11.662039 5.864465; 1 6.935858 11.130506 6.039446; 6 4.233311 10.359915 3.594537; 1 3.935717 9.415979 4.064237; 1 4.085077 11.157341 4.335540; 1 3.558235 10.557940 2.754468; 6 8.279312 11.416034 0.588466; 1 8.586321 12.467014 0.688777; 1 9.182836 10.843781 0.353770; 1 7.591898 11.342489 -0.261501;
33	6 7.092831 6.631687 1.251198; 6 6.696055 7.306062 0.037935; 6 5.348003 7.783474 0.205817; 6 4.922353 7.450366 1.534051; 6 6.010235 6.779342 2.194802; 6 8.193117 5.613405 1.349807; 1 8.999948 5.810936 0.641426; 1 7.791014

	4.617048 1.112105; 1 8.630593 5.555221 2.348040; 6 7.466338 7.297692 -1.252261; 1 7.195786 8.143979 -1.890305; 1 7.265295 6.379188 -1.821832; 1 8.544944 7.349924 -1.078885; 6 4.507385 8.446608 -0.848521; 1 3.783647 9.143158 -0.413057; 1 3.938894 7.702652 -1.424221; 1 5.120914 9.008584 -1.559036; 6 3.553242 7.677167 2.106310; 1 3.582994 7.794866 3.193712; 1 2.895136 6.823968 1.889263; 1 3.076573 8.569449 1.688949; 6 5.932037 6.123463 3.544824; 1 6.919875 6.018806 4.000291; 1 5.496207 5.117246 3.464378; 1 5.307430 6.696084 4.237503; 6 7.995648 10.651719 1.105547; 6 6.573020 10.954289 1.272979; 1 5.922495 11.281520 0.463879; 6 6.062016 10.675630 2.593523; 6 7.123432 10.149850 3.463412; 1 6.786707 9.752613 4.422233; 6 8.287351 11.132358 3.503171; 6 8.793886 11.344359 2.192556; 77 6.751034 8.900959 1.687748; 1 9.672040 7.967329 1.145646; 6 10.758373 7.922746 1.255937; 6 11.190260 8.568457 2.536452; 1 11.210616 8.415662 0.390376; 1 11.064846 6.867441 1.228372; 6 12.319562 9.419530 2.698557; 6 10.603905 8.339407 3.842155; 6 12.432446 9.753503 4.105794; 6 13.302500 9.813351 1.634563; 6 11.381023 9.047990 4.806644; 6 9.474945 7.410172 4.163287; 77 10.480225 10.680990 3.472046; 6 13.622199 10.392339 4.763721; 1 12.836062 9.858606 0.645807; 1 14.127549 9.088856 1.577739; 1 13.740608 10.792782 1.841994; 6 11.236527 8.969805 6.301137; 1 8.652617 7.541972 3.457144; 1 9.083470 7.599686 5.167164; 1 9.808681 6.362543 4.134296; 6 12.414985 13.211654 3.441906; 6 12.397596 13.647700 4.780784; 1 14.114675 11.113498 4.109318; 1 14.359672 9.622961 5.035344; 1 13.342968 10.925265 5.676131; 1 10.193103 8.843625 6.603125; 1 11.613532 9.874772 6.784571; 1 11.804668 8.117895 6.701364; 6 13.588751 13.430217 2.707123; 1 11.496392 13.490983 5.370067; 6 13.500830 14.262065 5.372189; 6 14.720325 14.040083 3.272134; 1 13.616029 13.136729 1.658609; 6 14.664748 14.444164 4.606609; 1 15.535211 14.916178 5.059914; 1 9.041467 12.357483 1.877770; 6 11.199960 12.610440 2.794772; 1 11.371901 12.512294 1.716589; 1 10.310634 11.602403 4.743845; 1 10.368900 13.310287 2.927638; 6 15.959279 14.277478 2.437120; 1 15.800114 15.081020 1.705495; 1 16.812880 14.565061 3.059940; 1 16.242520 13.381992 1.870744; 6 13.453104 14.734846 6.808477; 1 12.526047 14.423720 7.301078; 1 14.293355 14.337125 7.391425; 1 13.510895 15.829519 6.872129; 6 7.968986 12.293370 4.445413; 1 7.948766 11.962727 5.490858; 1 8.698892 13.101947 4.363341; 1 6.976806 12.709410 4.212083; 6 4.641778 10.939820 3.010983; 1 4.311359 10.219418 3.766940; 1 4.558571 11.941511 3.454496; 1 3.948455 10.894254 2.162923; 6 8.576497 10.708844 -0.290991; 1 8.901276 11.731667 -0.536697; 1 9.455954 10.063343 -0.380471; 1 7.849144 10.396153 -1.048271;
33-24_ts	6 7.093902 6.643469 1.219886; 6 6.719540 7.324438 0.005007; 6 5.367894 7.799001 0.149460; 6 4.913936 7.450251 1.464456; 6 5.989923 6.779469 2.143180; 6 8.194232 5.626999 1.337731; 1 9.010922 5.823383 0.640385; 1 7.797783 4.628563 1.098689; 1 8.618169 5.573275 2.342471; 6 7.515691 7.331697 -1.269395; 1 7.269152 8.194070 -1.895500; 1 7.314857 6.427361 -1.861515; 1 8.591147 7.366623 -1.073328; 6 4.549235 8.470578 -0.916861; 1 3.812967 9.159669 -0.490445; 1 3.996978 7.732048 -1.515256; 1 5.177310 9.043533 -1.605777; 6 3.532788 7.673453 2.008549; 1 3.538013 7.772326 3.098105; 1 2.874744 6.828379 1.761216; 1 3.070914 8.576115 1.596244; 6 5.894353 6.115402 3.488131; 1 6.872164 6.046219 3.972165; 1 5.497977 5.093956 3.394847; 1 5.229970 6.663880 4.163240; 6 7.917127 10.692645 1.102672; 6 6.504813 10.968996 1.357287; 1 5.803143 11.309290 0.597347; 6 6.070377 10.630649 2.692172; 6 7.180712 10.088456 3.484611; 1 6.893199 9.640229 4.437824; 6 8.354375 11.066009 3.515238; 6 8.791162 11.322294 2.166374; 77 6.737707 8.903097 1.679569; 1 9.784187 7.935168 1.100913; 6 10.865455 7.858969 1.241411; 6 11.276902 8.526001 2.517385; 1 11.353596 8.318942 0.376486; 1 11.137272 6.793555 1.245617; 6 12.420406 9.335850 2.710710; 6 10.578408 8.423926 3.796753; 6 12.450177 9.735944 4.112082; 6 13.469933 9.680379 1.693884; 6 11.366065 9.106038 4.797070; 6 9.442407 7.500318 4.110555; 77 10.468301 10.601889 3.309303; 6 13.585292 10.466336 4.772072; 1 13.078313 9.625971 0.673408; 1 14.325738 8.991983 1.754507; 1 13.860447 10.691432 1.846905; 6 11.147030 9.073169 6.282856; 1 8.663927 7.569969 3.349714; 1 8.985023 7.756237 5.070945; 1 9.795064 6.460608 4.178648; 6 12.418967 13.440878 3.457134; 6 12.444273 13.945487 4.768010; 1 14.046126 11.199710 4.107030; 1 14.366441 9.754798 5.076183; 1 13.256463 11.000473 5.667619; 1 10.083299 9.030565 6.533799; 1 11.562371 9.961561 6.767677; 1 11.629130 8.194128 6.736744; 6 13.579695 13.550543 2.682132; 1 11.550336 13.865122 5.384418; 6 13.589347 14.540743 5.300166; 6 14.746979 14.143133 3.185920; 1 13.571102 13.176803 1.659653; 6 14.736799 14.626587 4.496602; 1 15.638215 15.082657 4.902943; 1 9.001121 12.357120 1.878284; 6 11.168187 12.847100 2.878149; 1 11.310269 12.576212 1.832923; 1 10.648584 12.097762 3.977910; 1 10.335993 13.557207 2.923775; 6 15.976850 14.278948 2.316351; 1 15.890794 15.137185 1.636341; 1 16.878830 14.429177 2.918828; 1 16.130135 13.390107 1.693736; 6 13.598617 15.094146 6.707459; 1 12.698168 14.803400 7.257957; 1 14.468676 14.738787 7.272633; 1 13.644802 16.191141 6.704417; 6 8.058918 12.181931 4.519212; 1 8.106996 11.806062 5.549207; 1 8.761963 13.016085 4.442475; 1 7.047362 12.591252 4.367360; 6 4.668038 10.849792 3.189660; 1 4.391651 10.096523 3.935597; 1 4.586851 11.833505 3.672500; 1 3.932692 10.817671 2.377005; 6 8.426699 10.794606 -0.318779; 1 8.726445 11.828102 -0.554034; 1 9.310396 10.163861 -0.463053; 1 7.668921 10.491195 -1.049895;
28	77 -0.736220 2.373666 4.964609; 6 -2.519808 2.945504 5.943182; 6 -1.452972 2.665315 6.930943; 6 0.605260 3.634968 3.933090; 6 -0.228620 3.105852 2.837985; 6 -0.047767 1.713143 2.846194; 6 1.004592 1.375730 3.832147; 6 1.488962 2.563322 4.413172; 6 2.636493 2.733132 5.362678; 6 1.522854 -0.014146 4.055730; 6 -0.722480 0.704727 1.964362; 6 -1.110662 3.925878 1.945787; 6 0.776570 5.090623 4.245444; 1 -1.458892 1.698976 7.444835; 6 -0.917669 3.788376 7.690316; 6 -1.343038 5.062972 7.461640; 1 -0.932351 5.887114 8.040423; 6 -2.382012 5.335859 6.498056; 6 -2.964724 4.325045 5.793224; 1 -3.797123 4.530264 5.122657; 1 -1.640017 1.105702 1.524724; 1 -0.989200 -0.199235 2.521776; 1 -0.062847 0.396267 1.140582; 1 -1.875698 3.311013 1.462301; 1 -0.527609 4.415600 1.152159; 1 -1.625496 4.711629 2.507919; 1 -0.165573 5.633163 4.129313; 1 1.518574 5.543083 3.571691; 1 1.116128 5.236804 5.274408; 1 2.880842 1.794405 5.869060; 1 2.409086 3.474180 6.135428; 1 3.541585 3.071601 4.837026; 1 0.708008 -0.744830 4.087424; 1 2.074540 -0.090369 4.996863; 1 2.200274 -0.316685 3.244299; 1 -3.275625 2.175199 5.759964; 1 -2.729501 6.358903 6.373666; 1 -0.185816 3.583989 8.469584;
28-29_ts	6 7.455560 7.139224 1.107581; 6 6.151638 7.186461 0.484183; 6 5.152111 6.838776 1.470401; 6 5.815649 6.635790 2.715700; 6 7.230969 6.857732 2.509933; 6 8.784192 7.208811 0.410681; 1 8.740808 7.855511 -0.470597; 1 9.111304 6.212897 0.076988; 1 9.561087 7.609082 1.068738; 6 5.896834 7.371560 -0.985143; 1 4.923364 7.835244 -1.171896; 1 5.907375 6.404298 -1.506848; 1 6.660045 8.005208 -1.445304; 6 3.681218 6.682471 1.211595; 1 3.087279 6.921128 2.099578; 1 3.436426 5.650718 0.921122; 1 3.343226 7.336345 0.402023; 6 5.170490 6.226909 4.008552; 1 5.728927 6.599513 4.872461; 1 5.122122 5.132286 4.098681; 1 4.146774 6.606497 4.087185; 6 8.306965 6.629012 3.533869; 1 9.195158 7.229831 3.318919; 1 8.614261 5.573986 3.549224; 1 7.967270 6.887018 4.541754; 6 6.085800 10.743658 0.939671; 6 5.223776 10.672455 2.119668; 1 4.139815 10.707155 2.076065; 6 5.942574 10.470429 3.334745; 6 7.384827 10.372485 3.128683; 1 7.976494 10.037900 3.974683; 6 8.002713 11.372611 2.214159; 6 7.328755 11.536377 1.055802; 1 7.610834 12.235878 0.275066; 77 6.327163 8.837944 1.994484; 1 12.758802 12.438736

	3.851032; 6 12.471948 13.424032 4.231202; 6 11.038307 13.449529 4.673739; 1 12.654813 14.151091 3.434079; 1 13.149556 13.668045 5.063546; 6 10.206753 14.641216 4.797354; 6 10.246902 12.355812 5.117672; 6 8.975679 14.267400 5.509858; 6 10.716397 16.046505 4.677557; 6 8.988718 12.873506 5.680449; 6 10.705547 10.928768 5.230029; 77 9.198794 13.386428 3.341915; 6 7.934566 15.242080 5.972440; 1 11.420268 16.150686 3.848596; 1 11.234311 16.337237 5.603517; 1 9.904983 16.756965 4.506050; 6 7.965170 12.049122 6.405023; 1 11.462886 10.690128 4.477320; 1 9.880084 10.221579 5.104927; 1 11.147486 10.735865 6.218499; 6 9.407359 14.200458 1.361961; 6 8.103437 14.594400 1.921205; 1 7.749847 16.009657 5.213834; 1 8.255263 15.756954 6.889933; 1 6.982189 14.747229 6.185356; 1 7.819154 11.072104 5.936096; 1 6.992422 12.549104 6.432480; 1 8.273132 11.865597 7.445019; 6 10.382252 15.250456 1.101686; 1 7.210474 14.047483 1.629634; 6 7.892775 16.005328 2.207995; 6 10.128143 16.552663 1.410385; 1 11.314339 14.977393 0.609236; 6 8.860361 16.937893 1.980068; 1 8.670399 17.989371 2.184766; 1 6.911483 16.315463 2.564425; 1 9.450263 13.366371 0.660101; 1 10.866547 17.322109 1.195424; 1 5.585914 10.732249 -0.026318; 1 9.078588 11.701686 2.348398; 1 5.448867 10.337877 4.292320;
29	6 7.223438 7.048683 1.880647; 6 6.830863 7.430005 0.541339; 6 5.391496 7.569431 0.503365; 6 4.892366 7.341322 1.820817; 6 6.019754 7.056792 2.682072; 6 8.582465 6.577396 2.312682; 1 9.377254 7.076413 1.750062; 1 8.697545 5.493762 2.158206; 1 8.758178 6.778987 3.373661; 6 7.741472 7.470433 -0.653656; 1 7.394340 8.189796 -1.401794; 1 7.793814 6.486335 -1.140812; 1 8.758945 7.754130 -0.369798; 6 4.569555 7.855970 -0.720438; 1 3.650753 8.397237 -0.472966; 1 4.275050 6.926178 -1.228471; 1 5.123933 8.459948 -1.445107; 6 3.449509 7.345659 2.237407; 1 3.333785 7.650438 3.281919; 1 3.003108 6.345596 2.137854; 1 2.854305 8.030947 1.625446; 6 5.930354 6.638214 4.122769; 1 6.848227 6.880230 4.666129; 1 5.769269 5.554026 4.206987; 1 5.101452 7.134674 4.636618; 6 7.662716 10.871645 1.305152; 6 6.268121 11.215006 1.459062; 1 5.635769 11.547018 0.641197; 6 5.750967 10.987583 2.776884; 6 6.719518 10.456644 3.707658; 1 6.321388 10.107224 4.659138; 6 8.153347 10.895312 3.708904; 6 8.673428 11.124769 2.383296; 1 9.658613 10.740439 2.121898; 77 6.431241 9.135005 1.944080; 1 11.770732 12.054060 1.536965; 6 12.027222 13.016897 1.989944; 6 11.283054 13.238129 3.275944; 1 11.800378 13.794638 1.254878; 1 13.114944 13.021384 2.153668; 6 10.853206 14.480300 3.812512; 6 10.987299 12.211443 4.265084; 6 10.329264 14.250798 5.146756; 6 11.040787 15.834524 3.199541; 6 10.432681 12.865725 5.441454; 6 11.535223 10.811006 4.239158; 77 8.995182 12.884611 3.599393; 6 9.908956 15.337850 6.088406; 1 11.296530 15.765544 2.138157; 1 11.853412 16.378403 3.701992; 1 10.134887 16.443395 3.281994; 6 10.155260 12.198372 6.758394; 1 11.572815 10.402496 3.224859; 1 10.941315 10.125581 4.851132; 1 12.560450 10.794307 4.634384; 6 7.479304 14.030321 2.415617; 6 6.957123 13.800843 3.747116; 1 9.223704 16.044544 5.609678; 1 10.782320 15.907073 6.437498; 1 9.403750 14.934510 6.970862; 1 9.780372 11.179062 6.622379; 1 9.406658 12.747106 7.337308; 1 11.066416 12.132194 7.371082; 6 7.779797 15.389976 2.025368; 1 6.301618 12.958847 3.928383; 6 6.765654 14.944139 4.611691; 6 7.591363 16.438672 2.881431; 1 8.113471 15.572010 1.005577; 6 7.073568 16.211121 4.201637; 1 6.885331 17.060663 4.854263; 1 6.316227 14.781709 5.589486; 1 7.209860 13.358701 1.610745; 1 7.787459 17.457177 2.553891; 1 8.039430 10.862502 0.283429; 1 8.752955 10.339844 4.429030; 1 4.702666 11.136050 3.017918;
29-30_ts	6 7.131324 6.972771 1.822351; 6 6.735664 7.431219 0.508235; 6 5.305952 7.650267 0.507028; 6 4.820167 7.393054 1.823943; 6 5.945478 7.009514 2.648534; 6 8.472136 6.417177 2.208905; 1 9.279980 6.890444 1.642633; 1 8.526382 5.333922 2.021357; 1 8.681265 6.579099 3.270680; 6 7.625590 7.473729 -0.702226; 1 7.298622 8.235395 -1.416943; 1 7.624766 6.507145 -1.226133; 1 8.659698 7.701291 -0.427863; 6 4.477298 8.037761 -0.684041; 1 3.601750 8.626653 -0.392077; 1 4.111185 7.150895 -1.221613; 1 5.053493 8.634706 -1.397526; 6 3.387641 7.459071 2.270197; 1 3.308595 7.719303 3.330131; 1 2.881669 6.492235 2.133068; 1 2.822271 8.206134 1.703740; 6 5.861598 6.533886 4.071745; 1 6.800858 6.706853 4.604907; 1 5.647340 5.456360 4.114751; 1 5.069091 7.048918 4.623529; 6 7.801222 10.772033 1.409362; 6 6.434931 11.195521 1.604895; 1 5.810897 11.609878 0.818559; 6 5.927916 10.941416 2.925288; 6 6.881271 10.308838 3.807386; 1 6.478732 9.937010 4.748884; 6 8.344024 10.662456 3.807726; 6 8.858682 10.914821 2.471274; 1 9.803253 10.456921 2.170909; 77 6.461536 9.095772 1.991579; 1 12.037908 12.011087 1.686067; 6 12.203771 13.006322 2.109806; 6 11.414444 13.206742 3.370576; 1 11.932309 13.739014 1.344101; 1 13.282948 13.107104 2.298817; 6 10.835630 14.405884 3.841289; 6 11.180140 12.179129 4.396815; 6 10.319489 14.181272 5.192864; 6 10.851050 15.738681 3.152306; 6 10.570918 12.842721 5.561596; 6 11.850473 10.834088 4.433163; 77 9.231380 12.595246 3.709256; 6 9.752603 15.258519 6.070195; 1 11.039096 15.633931 2.079758; 1 11.639796 16.385163 3.564838; 1 9.900491 16.267255 3.268393; 6 10.358527 12.207731 6.904824; 1 11.929327 10.395051 3.433984; 1 11.302812 10.127721 5.064319; 1 12.868734 10.919689 4.838651; 6 7.362743 14.393633 2.094657; 6 6.984460 14.011723 3.392339; 1 9.087582 15.925702 5.514295; 1 10.555760 15.877296 6.496765; 1 9.182002 14.840363 6.905017; 1 10.049788 11.162170 6.809578; 1 9.585578 12.727608 7.478623; 1 11.282873 12.224847 7.501487; 6 7.457837 15.754704 1.770356; 1 6.789145 12.966951 3.623307; 6 6.678533 14.994501 4.349402; 6 7.145568 16.722790 2.722369; 1 7.755138 16.053037 0.768440; 6 6.748092 16.343223 4.014569; 1 6.487244 17.102873 4.747000; 1 6.374461 14.691536 5.347573; 1 7.532724 13.636602 1.338688; 1 7.194600 17.776996 2.460605; 1 8.155293 10.780130 0.378963; 1 8.918250 10.028415 4.485373; 1 4.897183 11.149853 3.197008;
28-31_ts	6 6.645640 6.704095 1.334910; 6 5.864268 6.910559 0.131232; 6 4.739204 7.725107 0.467738; 6 4.841212 8.064790 1.865362; 6 5.993000 7.377684 2.424401; 6 7.833289 5.791571 1.452213; 1 8.452658 5.816059 0.550313; 1 7.508353 4.752215 1.601301; 1 8.466150 6.067333 2.299493; 6 6.149013 6.296455 -1.209398; 1 5.759952 6.911489 -2.027089; 1 5.686065 5.303117 -1.296654; 1 7.223592 6.169705 -1.372107; 6 3.638204 8.143190 -0.463739; 1 3.203293 9.102808 -0.168396; 1 2.824567 7.403956 -0.469886; 1 3.995151 8.243518 -1.493737; 6 3.818125 8.824498 2.661419; 1 4.276886 9.333550 3.513930; 1 3.045051 8.149766 3.055017; 1 3.315201 9.582908 2.053277; 6 6.304278 7.229668 3.885175; 1 7.368253 7.060384 4.063085; 1 5.752203 6.379053 4.311742; 1 6.019167 8.121636 4.450083; 6 8.755389 9.543183 0.456684; 6 7.772622 10.127364 -0.454464; 1 7.731789 9.925072 -1.520573; 6 6.794336 10.924896 0.214789; 6 6.993643 10.974062 1.667014; 1 6.193344 11.425867 2.249239; 6 8.392909 11.222911 2.108668; 6 9.316970 10.478558 1.470981; 77 6.703693 8.927051 0.955251; 1 10.273556 13.195070 6.388435; 6 11.059068 12.492987 6.684699; 6 11.707166 11.853960 5.492796; 1 10.601726 11.743389 7.338140; 1 11.792878 13.051010 7.285478; 6 12.440636 10.580383 5.501181; 6 11.751102 12.328912 4.165029; 6 13.122910 10.433554 4.200937; 6 12.796679 9.807372 6.734909; 6 12.648000 11.464845 3.373047; 6 11.120310 13.584390 3.636250; 77 10.829868 10.192113 4.184613; 6 14.131378 9.373160 3.875095; 1 11.977960 9.814084 7.458758; 1 13.684842 10.243394 7.215521; 1 13.013919 8.762297 6.499722; 6 13.042420 11.739531 1.950918; 1 10.323837 13.941309 4.295834; 1 10.685342 13.431966 2.642454; 1 11.862070 14.390958 3.544712; 6 9.214965 9.057443 4.960537; 6 9.190280

	8.743205 6.382957; 1 13.835303 8.404742 4.291095; 1 15.118797 9.623111 4.290578; 1 14.249480 9.247849 2.794363; 1 12.192933 12.084573 1.351455; 1 13.450973 10.847886 1.465900; 1 13.809283 12.526062 1.898983; 6 10.057861 8.213325 4.085475; 1 8.514419 9.305622 7.025743; 6 9.955849 7.743639 6.905278; 6 10.812398 7.132169 4.704526; 1 9.720101 8.038456 3.062273; 6 10.783840 6.925081 6.051925; 1 11.352872 6.109889 6.493760; 1 10.390108 10.624232 1.557406; 1 8.291393 9.462571 4.545851; 1 5.959463 11.386632 -0.303574; 1 8.627036 12.028572 2.798012; 1 9.422898 8.794165 0.036293; 1 9.919378 7.524850 7.970358; 1 11.381431 6.462326 4.061101;
31	6 6.708009 6.847727 2.067917; 6 6.401480 7.075864 0.668976; 6 5.160131 7.803255 0.592631; 6 4.731989 8.089560 1.932503; 6 5.692399 7.507020 2.841474; 6 7.727088 5.877247 2.588371; 1 8.612621 5.832008 1.949326; 1 7.296389 4.865423 2.624951; 1 8.065773 6.131272 3.594813; 6 7.146915 6.477049 -0.490342; 1 7.025808 7.074441 -1.399370; 1 6.787796 5.461906 -0.711697; 1 8.218597 6.408996 -0.280979; 6 4.419653 8.160459 -0.664449; 1 3.860498 9.095094 -0.553563; 1 3.697192 7.376444 -0.932243; 1 5.100553 8.280120 -1.512544; 6 3.457049 8.778625 2.328400; 1 3.565307 9.307439 3.280345; 1 2.636550 8.056423 2.446908; 1 3.142090 9.510063 1.577355; 6 5.549226 7.450344 4.336012; 1 6.516605 7.316092 4.826570; 1 4.906539 6.609022 4.632119; 1 5.096876 8.364244 4.733487; 6 8.607906 10.023876 0.801441; 6 7.389478 10.730343 0.409806; 1 7.029109 10.828531 -0.610543; 6 6.635707 11.204932 1.540161; 6 7.261028 10.872480 2.817025; 1 6.650465 11.028571 3.706152; 6 8.709274 11.287060 2.890448; 6 9.464688 10.814660 1.761971; 77 6.786387 9.094758 1.608706; 1 9.706825 13.160354 5.532969; 6 10.552627 12.679222 6.034060; 6 11.351942 11.834676 5.083037; 1 10.147268 12.082440 6.856308; 1 11.168666 13.476990 6.474788; 6 12.052909 10.635294 5.384671; 6 11.642603 12.164318 3.697189; 6 12.832017 10.248049 4.219162; 6 12.149455 9.968609 6.723593; 6 12.605072 11.197801 3.190879; 6 11.316091 13.472845 3.032073; 77 10.477745 10.278223 3.580101; 6 13.819775 9.121363 4.199888; 1 11.371474 10.321924 7.406601; 1 13.122148 10.178659 7.190841; 1 12.047111 8.881985 6.643556; 6 13.297508 11.281969 1.860892; 1 10.405801 13.921980 3.439838; 1 11.175854 13.363262 1.951993; 1 12.131026 14.194120 3.184107; 6 9.233356 8.585273 4.285473; 6 9.499541 7.997756 5.580958; 1 13.390469 8.202921 4.612528; 1 14.706062 9.372341 4.799926; 1 14.160390 8.900282 3.183990; 1 12.620629 11.633815 1.075429; 1 13.687085 10.309230 1.546709; 1 14.144717 11.982602 1.897257; 6 10.037928 8.152459 3.157683; 1 8.858540 8.273670 6.416707; 6 10.486722 7.067960 5.755615; 6 11.069535 7.165431 3.395835; 1 9.589883 8.157861 2.171453; 6 11.290598 6.646249 4.640777; 1 12.045159 5.876936 4.789641; 1 10.140478 11.493917 1.235418; 1 8.218338 8.913685 4.098359; 1 5.663966 11.678823 1.431002; 1 8.814767 12.323264 3.219025; 1 9.119519 9.477928 0.008995; 1 10.645889 6.610906 6.729813; 1 11.639533 6.796425 2.544823;
31-32_ts	6 6.973563 6.779659 1.378601; 6 6.254467 7.299811 0.234417; 6 5.040447 7.906200 0.710539; 6 5.033261 7.816421 2.145296; 6 6.215993 7.088896 2.555877; 6 8.189662 5.902338 1.300595; 1 8.876622 6.234407 0.515872; 1 7.903865 4.865603 1.070345; 1 8.740852 5.891975 2.243085; 6 6.639694 7.092722 -1.203076; 1 6.234661 7.879459 -1.847368; 1 6.263084 6.131516 -1.581501; 1 7.726707 7.090339 -1.329308; 6 3.953821 8.494403 -0.143656; 1 3.420960 9.296086 0.377153; 1 3.211493 7.731675 -0.418300; 1 4.352522 8.911501 -1.073771; 6 3.912893 8.233789 3.055354; 1 4.287083 8.521993 4.042452; 1 3.193440 7.415333 3.203023; 1 3.359674 9.086759 2.649941; 6 6.449508 6.551712 3.937610; 1 7.492267 6.275535 4.101469; 1 5.836202 5.652792 4.098920; 1 6.176143 7.276328 4.709998; 6 8.592868 10.155512 0.755683; 6 7.326441 10.787195 0.399206; 1 6.942457 10.885322 -0.613082; 6 6.550572 11.168577 1.551991; 6 7.213691 10.850804 2.811041; 1 6.597608 10.936679 3.706376; 6 8.633541 11.376757 2.888822; 6 9.411530 10.963591 1.734245; 77 6.845149 9.074789 1.534666; 1 9.922442 13.434692 5.345550; 6 10.753073 12.954416 5.873088; 6 11.454073 11.954129 5.001050; 1 10.344929 12.487917 6.774610; 1 11.440323 13.751324 6.194195; 6 11.999057 10.712074 5.380491; 6 11.752345 12.145014 3.576200; 6 12.708795 10.141218 4.230132; 6 11.994962 10.110396 6.754450; 6 12.626272 11.044682 3.147805; 6 11.583242 13.445572 2.840898; 77 10.436671 10.471782 3.517761; 6 13.542037 8.893974 4.289490; 1 11.217989 10.551879 7.385643; 1 12.960377 10.273203 7.256227; 1 11.819673 9.031328 6.725811; 6 13.348332 10.966633 1.834094; 1 10.634909 13.931563 3.091096; 1 11.606776 13.303971 1.756407; 1 12.391984 14.143778 3.099600; 6 9.533844 8.191323 3.933950; 6 8.907645 8.537703 5.144871; 1 13.041957 8.098578 4.849697; 1 14.503378 9.089987 4.786896; 1 13.762419 8.508507 3.289366; 1 12.738758 11.362372 1.015871; 1 13.608118 9.934773 1.579556; 1 14.282300 11.548082 1.859266; 6 10.484403 7.153676 3.914967; 1 8.120196 9.281700 5.137092; 6 9.251628 7.868302 6.327957; 6 10.800700 6.468722 5.085546; 1 10.963250 6.888897 2.976584; 6 10.184717 6.832822 6.294226; 1 10.429711 6.294746 7.206976; 1 10.016777 11.708213 1.207768; 1 9.128994 8.581572 2.991866; 1 5.545415 11.572710 1.462189; 1 8.646019 12.428902 3.195757; 1 9.130476 9.665648 -0.056259; 1 8.775772 8.140807 7.266371; 1 11.518507 5.652710 5.066571;
34	77 -0.042052 1.687101 5.466097; 6 -2.361237 2.687411 7.180970; 6 -1.325489 3.041831 6.280847; 6 1.881162 2.730834 4.697791; 6 1.091670 2.430215 3.576764; 6 0.859606 0.970780 3.564819; 6 1.609769 0.374618 4.620227; 6 2.111326 1.465606 5.421482; 6 2.971320 1.335672 6.641951; 6 1.787212 -1.090114 4.893894; 6 0.110914 0.249299 2.485217; 6 0.584993 3.373393 2.527248; 6 2.426652 4.062475 5.117937; 1 -0.830215 0.566770 6.315997; 6 -1.245018 4.417252 5.941452; 6 -2.127557 5.366061 6.452521; 1 -2.033318 6.410890 6.164042; 6 -3.136751 4.974283 7.338837; 6 -3.249986 3.631716 7.701060; 1 -4.031707 3.318952 8.389870; 1 -0.784298 0.802338 2.185614; 1 -0.204726 -0.744034 2.814208; 1 0.741744 0.125938 1.593288; 1 -0.451347 3.148857 2.256354; 1 1.188909 3.298048 1.612173; 1 0.621315 4.412698 2.864592; 1 1.922015 4.886932 4.606911; 1 3.497999 4.133899 4.884051; 1 2.312586 4.220097 6.194883; 1 2.735621 0.425047 7.198700; 1 2.833504 2.185869 7.315987; 1 4.033669 1.296467 6.360975; 1 0.944612 -1.673580 4.512318; 1 1.862692 -1.289577 5.966554; 1 2.701417 -1.471420 4.417618; 1 -2.467696 1.648879 7.476963; 1 -0.470118 4.748611 5.254293; 1 -3.827046 5.711762 7.741832;
34-35_ts	77 0.260814 1.482834 3.180134; 6 -1.032526 -0.398957 2.811443; 6 0.290544 -0.641339 2.385087; 6 0.610867 0.395534 1.400721; 6 -0.637207 1.106810 1.067615; 6 -1.614382 0.668455 1.974081; 6 -3.042706 1.118563 2.063269; 6 -0.782228 2.100484 -0.045774; 6 1.868915 0.457980 0.586801; 6 1.220734 -1.722495 2.851056; 6 -1.802108 -1.169931 3.842683; 1 2.736990 0.151122 1.176254; 1 2.054266 1.471994 0.222177; 1 1.797069 -0.209012 -0.284919; 1 2.257120 -1.374128 2.869892; 1 1.172629 -2.594046 2.182962; 1 0.970097 -2.060984 3.860330; 1 -1.133641 -1.696695 4.528737; 1 -2.451559 -1.918442 3.366464; 1 -2.442497 -0.513458 4.439911; 1 -3.196316 2.081438 1.568545; 1 -3.366955 1.222809 3.103601; 1 -3.711755 0.388322 1.586528; 1 0.061607 2.796318 -0.075403; 1 -1.695983 2.692794 0.055066; 1 -0.826013 1.587987 -1.016925; 6 0.998951 6.306039 3.484919; 6 -0.093010 5.783757 2.786374; 1 -0.811210 6.454403 2.318598; 6 -0.258849 4.402095 2.680869; 6 0.633722 3.481301 3.283696; 6 1.724075 4.048878 3.985917; 6 1.909262 5.430282 4.080662; 1 2.765827 5.824631 4.623893; 1 1.464256 1.331119 4.230730; 77 -1.789851 3.991820 8.738380; 6 -3.808761 4.093052 9.669092; 6 -3.443273 5.421908 9.228014; 6 -2.855934 3.723763 10.693083; 6 -5.026532 3.316099 9.258492; 6 -2.348405 5.896438 10.046021; 6 -4.202235

	6.247762 8.227608; 6 -1.987183 4.852247 10.946635; 6 -2.893990 2.456272 11.499759; 1 -5.309130 3.533179 8.224057; 1 -5.889569 3.557849 9.896991; 1 -4.854786 2.237794 9.328134; 6 -1.727824 7.262108 9.974550; 1 -3.544618 6.944289 7.698424; 1 -4.984469 6.841164 8.721745; 1 -4.687679 5.617461 7.477203; 6 -0.915737 4.916542 11.996843; 1 -3.298101 1.624593 10.915722; 1 -3.527257 2.577496 12.390062; 1 -1.896240 2.165664 11.842981; 1 -0.671453 7.241016 10.261050; 1 -2.236283 7.966572 10.648547; 1 -1.787545 7.678265 8.964405; 1 -0.492262 3.928201 12.199742; 1 -1.311199 5.303408 12.947134; 1 -0.093138 5.572542 11.694380; 6 -0.283342 4.203724 7.223045; 6 -1.563729 3.958843 6.565063; 6 0.091004 3.165181 8.125732; 6 -1.803169 2.584422 6.053104; 1 -1.976738 4.787349 5.994766; 6 -0.885671 2.078457 8.190594; 1 0.980856 3.219232 8.745242; 6 -1.447135 1.603226 6.899398; 1 -1.475317 0.543770 6.657585; 1 0.299729 5.108701 7.090865; 1 -0.739097 1.343068 8.979129; 1 -2.163082 2.423392 5.040251; 1 2.439797 3.389956 4.467409; 1 -1.116776 4.025749 2.128181; 1 1.139299 7.381887 3.560386;
35	77 -1.159711 2.332675 4.314185; 6 -1.782084 0.107589 3.732162; 6 -0.409221 0.343431 3.407179; 6 -0.367624 1.396831 2.415662; 6 -1.741235 1.741039 2.083087; 6 -2.604260 0.968241 2.906178; 6 -4.106233 0.958158 2.883904; 6 -2.144150 2.677183 0.982133; 6 0.843980 1.853684 1.652923; 6 0.770798 -0.439675 3.911137; 6 -2.307341 -0.955073 4.653790; 1 1.759663 1.696212 2.230383; 1 0.786350 2.920215 1.416887; 1 0.948683 1.303427 0.706919; 1 1.679141 0.168680 3.919078; 1 0.961216 -1.313977 3.272670; 1 0.609724 -0.802887 4.930356; 1 -1.564432 -1.245021 5.402658; 1 -2.580410 -1.860377 4.093590; 1 -3.206638 -0.625507 5.186221; 1 -4.508744 1.865197 2.424233; 1 -4.526306 0.885042 3.893532; 1 -4.490492 0.101200 2.312765; 1 -1.512911 3.569374 0.962182; 1 -3.180074 3.010917 1.090318; 1 -2.056421 2.179569 0.006103; 6 -0.388171 6.969018 2.839933; 6 -1.675567 6.429266 2.864388; 1 -2.521884 7.022463 2.522568; 6 -1.888032 5.128751 3.330889; 6 -0.832439 4.311880 3.785125; 6 0.455284 4.883101 3.748304; 6 0.676547 6.184760 3.285420; 1 1.688797 6.584811 3.275825; 1 0.157695 2.629795 5.130584; 77 -2.259885 4.083822 8.641112; 6 -4.016574 3.572028 9.910189; 6 -4.033830 4.998276 9.667265; 6 -2.815021 3.295926 10.669081; 6 -5.116017 2.599109 9.592910; 6 -2.905914 5.595682 10.345554; 6 -5.144070 5.753047 8.991706; 6 -2.157135 4.548224 10.963588; 6 -2.426817 1.954630 11.224722; 1 -5.644955 2.877702 8.676488; 1 -5.858346 2.556212 10.404320; 1 -4.726159 1.586798 9.449643; 6 -2.607940 7.065517 10.426688; 1 -4.776987 6.661906 8.505137; 1 -5.912083 6.054056 9.718343; 1 -5.632612 5.143128 8.226486; 6 -0.927926 4.718338 11.809640; 1 -2.759892 1.142068 10.572684; 1 -2.877992 1.793965 12.214205; 1 -1.342248 1.863422 11.338820; 1 -1.533707 7.255812 10.516453; 1 -3.095768 7.522154 11.300071; 1 -2.964494 7.597866 9.539858; 1 -0.281680 3.836673 11.762669; 1 -1.190631 4.874531 12.865979; 1 -0.334098 5.580709 11.490268; 6 -1.166730 4.872587 6.958882; 6 -2.461409 4.453447 6.474762; 6 -0.411571 3.828056 7.573233; 6 -2.615584 3.135853 5.775207; 1 -3.131691 5.248934 6.159392; 6 -1.087257 2.549685 7.587243; 1 0.550643 4.006613 8.042932; 6 -1.865963 2.098364 6.388874; 1 -2.262691 1.087257 6.449415; 1 1.307214 4.301337 4.089876; 1 -0.218956 7.981487 2.481461; 1 -2.904922 4.743036 3.344358; 1 -0.625479 1.764418 8.182861; 1 -0.820183 5.900018 6.930703; 1 -3.603060 2.937024 5.361201;
35-30_ts	77 -0.951956 2.393352 4.285780; 6 -2.050932 0.589315 3.599223; 6 -0.655382 0.180238 3.687143; 6 0.063767 0.912288 2.703762; 6 -0.890575 1.712927 1.945959; 6 -2.188874 1.483120 2.460528; 6 -3.488884 2.016861 1.932050; 6 -0.531482 2.570477 0.767983; 6 1.514067 0.736281 2.350859; 6 -0.107898 -0.895657 4.580500; 6 -3.202072 -0.077011 4.301914; 1 2.120301 0.501144 3.231454; 1 1.929376 1.637023 1.890489; 1 1.647647 -0.085009 1.631291; 1 0.958800 -0.749988 4.774967; 1 -0.230080 -1.888245 4.122368; 1 -0.618293 -0.918102 5.548236; 1 -2.899104 -0.508782 5.260362; 1 -3.610539 -0.894889 3.692029; 1 -4.020047 0.623911 4.498735; 1 -3.345497 2.937376 1.358743; 1 -4.190338 2.240476 2.742477; 1 -3.978869 1.287873 1.269930; 1 0.338966 3.200062 0.979834; 1 -1.354930 3.232685 0.486830; 1 -0.288106 1.951667 -0.106871; 6 1.137647 6.525879 2.525582; 6 -0.151090 6.555808 3.065546; 1 -0.762401 7.449394 2.959310; 6 -0.671992 5.447434 3.733835; 6 0.073302 4.255601 3.860278; 6 1.383530 4.257663 3.332789; 6 1.905514 5.369525 2.669082; 1 2.918342 5.332465 2.274309; 1 0.162833 3.454683 4.965512; 77 -2.048306 4.135049 8.598521; 6 -3.773260 3.491630 9.853896; 6 -3.877305 4.919529 9.642649; 6 -2.557741 3.270874 10.607320; 6 -4.812236 2.461852 9.512861; 6 -2.787031 5.568511 10.333860; 6 -5.032212 5.618921 8.982578; 6 -1.974589 4.554113 10.926983; 6 -2.089258 1.943812 11.134536; 1 -5.356305 2.728048 8.601628; 1 -5.551344 2.357265 10.321634; 1 -4.362797 1.477852 9.348544; 6 -2.576907 7.051182 10.448886; 1 -4.724332 6.565438 8.527547; 1 -5.823526 5.844833 9.711517; 1 -5.473708 5.001860 8.194734; 6 -0.758071 4.779162 11.778478; 1 -2.377236 1.126365 10.467320; 1 -2.525178 1.737022 12.122348; 1 -1.000630 1.914129 11.242299; 1 -1.515225 7.302825 10.537626; 1 -3.085120 7.457638 11.335396; 1 -2.970356 7.582247 9.576904; 1 -0.059711 3.939427 11.712766; 1 -1.029997 4.895270 12.837712; 1 -0.217293 5.682863 11.479660; 6 -1.014684 5.010116 6.934201; 6 -2.276542 4.518584 6.428414; 6 -0.192078 4.000474 7.530382; 6 -2.371306 3.202492 5.709013; 1 -3.002385 5.279340 6.144717; 6 -0.785609 2.683842 7.520297; 1 0.754930 4.233784 8.007307; 6 -1.552568 2.189892 6.329449; 1 -1.953524 1.185808 6.463156; 1 2.002896 3.375671 3.466238; 1 1.540158 7.395094 2.012069; 1 -1.675505 5.493938 4.135503; 1 -0.278902 1.924572 8.113798; 1 -3.380758 2.951888 5.381912; 1 -0.730283 6.057407 6.930601;
30	77 5.813432 1.878882 5.659285; 77 7.765285 2.372904 1.642039; 6 9.185431 3.156706 0.312487; 6 9.369181 1.701539 0.162052; 6 8.163003 1.194768 -0.362493; 6 7.264968 2.316390 -0.662949; 6 7.910221 3.524147 -0.328676; 6 6.322506 2.752182 7.650270; 6 6.337874 1.310344 7.772026; 6 4.988390 0.820247 7.607406; 6 4.143098 1.939040 7.331623; 6 4.964225 3.128638 7.326549; 6 7.434391 4.922888 -0.588796; 6 10.621692 0.941999 0.485369; 6 10.275848 4.125153 0.667530; 6 5.908167 2.164643 -1.286914; 6 7.836977 -0.243721 -0.641306; 6 2.653888 1.898920 7.140474; 6 4.457471 4.537999 7.200303; 6 4.547517 -0.607771 7.756485; 6 7.457128 3.686022 7.961517; 6 7.521279 0.484774 8.192631; 1 7.842515 5.312009 -1.533570; 1 7.743917 5.605701 0.208183; 1 6.343258 4.970713 -0.656006; 1 11.136953 1.370558 1.350262; 1 11.325788 0.958479 -0.359897; 1 10.407857 -0.105722 0.717249; 1 9.865278 5.060232 1.059563; 1 10.881191 4.369220 -0.217972; 1 10.945746 3.709709 1.426049; 1 5.357741 1.323593 -0.851972; 1 5.986720 1.976756 -2.367611; 1 5.300407 3.063652 -1.150167; 1 8.492752 -0.919217 -0.084532; 1 7.948673 -0.476904 -1.710216; 1 6.804508 -0.482463 -0.364611; 1 2.126129 2.005417 8.099490; 1 2.329360 0.953970 6.693035; 1 2.310118 2.708051 6.488958; 1 5.229741 5.205906 6.808119; 1 4.142359 4.931117 8.177542; 1 3.595820 4.598816 6.528268; 1 3.680373 -0.830607 7.126505; 1 4.263523 -0.830609 8.795359; 1 5.344043 -1.305533 7.480855; 1 8.424780 3.232214 7.727364; 1 7.468421 3.960629 9.027206; 1 7.384015 4.611525 7.382578; 1 7.471356 -0.529637 7.784863; 1 7.571755 0.398605 9.287529; 1 8.459779 0.931382 7.851974; 6 6.079683 0.712463 3.890481; 6 5.211553 1.824847 3.611471; 6 7.402127 1.104393 4.341726; 6 5.832834 3.116643 3.832727; 1 4.173078 1.691637 3.324105; 6 8.136002 2.252810 3.692277; 1 8.025200 0.298713 4.726959; 6 7.256838 3.376394 3.402921; 1 7.595723 4.402745 3.535518; 1 9.151711 2.410833 4.051654; 1 5.741222 -0.317190 3.827215; 1 5.161774 3.973584 3.800594;

34-36_ts	6 7.272700 7.144698 1.118700; 6 6.552485 7.716611 0.001948; 6 5.166796 7.885534 0.380283; 6 5.031018 7.479552 1.740865; 6 6.330913 7.056988 2.214710; 6 8.675083 6.608731 1.101248; 1 9.306072 7.162667 0.399992; 1 8.689581 5.550224 0.801008; 1 9.144506 6.687155 2.084960; 6 7.108043 7.920330 -1.379419; 1 6.623671 8.758132 -1.890946; 1 6.956476 7.023902 -1.997019; 1 8.181758 8.125019 -1.349470; 6 4.061890 8.360170 -0.519078; 1 3.283217 8.887904 0.041060; 1 3.579816 7.517028 -1.034855; 1 4.434633 9.041445 -1.289986; 6 3.755650 7.450027 2.532879; 1 3.944010 7.577183 3.602982; 1 3.232874 6.491005 2.405772; 1 3.066438 8.241290 2.220391; 6 6.612221 6.447356 3.558648; 1 7.653136 6.590484 3.858316; 1 6.413802 5.366183 3.541834; 1 5.981062 6.884428 4.338766; 6 7.721336 10.957075 0.939062; 6 6.455371 11.335463 1.574166; 1 5.638976 11.828254 1.055040; 6 6.363653 10.913702 2.935861; 6 7.554355 10.194642 3.391229; 1 7.490923 9.689529 4.351815; 6 8.863146 10.801768 3.022239; 6 8.952343 11.186620 1.735835; 77 6.646800 9.207577 1.671128; 1 11.058354 13.036179 4.485021; 6 11.966717 12.770154 5.036507; 6 12.682671 11.616678 4.396422; 1 11.677906 12.546721 6.067050; 1 12.606209 13.663622 5.055944; 6 13.260889 10.510555 5.037613; 6 12.948955 11.521940 2.949776; 6 13.794453 9.627344 3.983776; 6 13.334902 10.225962 6.508074; 6 13.701069 10.356090 2.706867; 6 12.585290 12.586959 1.957208; 77 11.741338 9.578903 3.531121; 6 14.665003 8.431221 4.235947; 1 12.653716 10.863236 7.078807; 1 14.351377 10.404376 6.885442; 1 13.073866 9.186700 6.728125; 6 14.322685 9.920312 1.412682; 1 11.579693 12.980926 2.136470; 1 12.617643 12.208189 0.931965; 1 13.281913 13.435316 2.020309; 6 10.777976 8.251479 4.747307; 6 10.135408 8.707864 5.921925; 1 14.308051 7.858003 5.095841; 1 15.700384 8.741589 4.439684; 1 14.677748 7.761819 3.371428; 1 13.788770 10.330506 0.550890; 1 14.316749 8.831196 1.316572; 1 15.367736 10.255903 1.351834; 6 10.740344 6.856167 4.525059; 1 10.137237 9.771668 6.153617; 6 9.485975 7.838878 6.804160; 6 10.114970 5.978115 5.414809; 1 11.220848 6.450286 3.639034; 6 9.477069 6.464529 6.558987; 1 8.984161 5.783687 7.248717; 1 9.805034 11.706978 1.309484; 1 11.429645 8.519982 2.372572; 1 5.467032 11.052950 3.532315; 1 9.606347 11.034919 3.784300; 1 7.783413 11.100440 -0.137789; 1 8.996228 8.237218 7.690823; 1 10.121486 4.909082 5.210318;
36	6 7.470398 7.201206 1.200511; 6 6.882837 7.857293 0.049765; 6 5.466885 7.994201 0.278787; 6 5.177771 7.475860 1.583904; 6 6.412557 7.000892 2.161471; 6 8.858818 6.638633 1.270574; 1 9.588472 7.300339 0.794123; 1 8.908895 5.663402 0.763468; 1 9.174347 6.494643 2.305182; 6 7.603298 8.168532 -1.231542; 1 7.148609 9.014664 -1.756525; 1 7.583921 7.307276 -1.914298; 1 8.651753 8.418958 -1.045306; 6 4.461970 8.538963 -0.695575; 1 3.636605 9.045372 -0.184821; 1 4.025899 7.734027 -1.304377; 1 4.917014 9.257756 -1.383637; 6 3.815645 7.373617 2.208954; 1 3.870789 7.407553 3.300976; 1 3.325019 6.428367 1.935617; 1 3.159424 8.187603 1.884274; 6 6.540997 6.276192 3.470828; 1 7.520058 6.438507 3.927081; 1 6.405441 5.194257 3.329557; 1 5.786077 6.609925 4.189162; 6 7.881026 10.989152 1.450487; 6 6.470242 11.326018 1.671396; 1 5.817908 11.773671 0.927262; 6 5.993390 10.883560 2.951837; 6 7.026396 10.218722 3.743554; 1 6.697375 9.682906 4.630295; 6 8.306794 11.002617 3.848550; 6 8.779419 11.407225 2.576405; 77 6.705881 9.222573 1.825375; 1 10.537022 13.724250 4.449185; 6 11.398508 13.268054 4.950706; 6 11.808536 11.981102 4.292354; 1 11.124611 13.113842 5.998121; 1 12.212645 14.005861 4.928060; 6 12.186673 10.773597 4.929179; 6 11.951295 11.794204 2.858330; 6 12.530529 9.804210 3.892568; 6 12.309305 10.524478 6.403674; 6 12.429533 10.471061 2.617308; 6 11.803821 12.879689 1.831683; 77 10.323244 10.193568 3.563597; 6 13.202197 8.484022 4.142739; 1 11.756103 11.265896 6.987328; 1 13.361597 10.573517 6.716806; 1 11.924263 9.538108 6.676608; 6 12.860344 9.909390 1.291603; 1 11.011699 13.588919 2.094008; 1 11.574973 12.474667 0.841651; 1 12.733952 13.459198 1.745805; 6 9.718706 8.756581 4.944102; 6 9.077576 9.088590 6.154591; 1 12.771741 7.971699 5.007091; 1 14.275883 8.623464 4.333908; 1 13.104943 7.815362 3.282031; 1 12.286516 10.339712 0.465694; 1 12.727312 8.824720 1.255707; 1 13.922835 10.120173 1.103742; 6 10.070111 7.401425 4.784308; 1 8.786894 10.118507 6.344541; 6 8.800725 8.133402 7.138072; 6 9.801034 6.438123 5.763802; 1 10.579142 7.089543 3.877261; 6 9.158505 6.797409 6.949280; 1 8.943653 6.052755 7.711692; 1 9.242753 12.386430 2.456331; 1 9.764960 9.102608 2.570955; 1 4.948619 10.961418 3.239102; 1 8.369043 11.682346 4.700916; 1 8.260505 11.123670 0.438586; 1 8.304061 8.439268 8.057041; 1 10.095886 5.403942 5.595035;
36-32_ts	6 7.324359 6.964351 1.513840; 6 6.745793 7.416930 0.263104; 6 5.368408 7.765097 0.504422; 6 5.105077 7.587204 1.902562; 6 6.310601 7.092244 2.525770; 6 8.660074 6.294205 1.654339; 1 9.424625 6.787917 1.046447; 1 8.603707 5.244394 1.330158; 1 9.005427 6.302959 2.689973; 6 7.420773 7.349343 -1.077580; 1 7.014352 8.090603 -1.772986; 1 7.290816 6.359334 -1.537460; 1 8.495853 7.532872 -0.989937; 6 4.370865 8.193529 -0.533575; 1 3.625514 8.879802 -0.119064; 1 3.828950 7.327742 -0.940066; 1 4.855635 8.698687 -1.374483; 6 3.781741 7.774240 2.588323; 1 3.912390 8.070632 3.633475; 1 3.194844 6.844269 2.582707; 1 3.177624 8.544603 2.098488; 6 6.406303 6.610997 3.943884; 1 7.437689 6.586320 4.298001; 1 5.996206 5.593905 4.027516; 1 5.840975 7.250329 4.627993; 6 8.284663 10.558140 0.950191; 6 6.932435 11.103457 0.827372; 1 6.436386 11.334918 -0.111209; 6 6.257240 11.179264 2.093533; 6 7.076295 10.725595 3.211692; 1 6.540447 10.582380 4.148122; 6 8.452607 11.353214 3.271567; 6 9.117621 11.233948 2.000470; 77 6.802838 9.165077 1.669140; 1 10.524399 13.333027 5.539621; 6 11.398076 12.753594 5.856932; 6 11.827216 11.781429 4.796791; 1 11.134632 12.246145 6.789555; 1 12.197524 13.473182 6.087054; 6 12.312985 10.468484 4.974218; 6 11.819075 12.049102 3.368310; 6 12.683234 9.923548 3.668814; 6 12.509672 9.752322 6.277756; 6 12.441939 10.921643 2.687266; 6 11.578003 13.408833 2.771456; 77 10.356552 10.370768 3.501059; 6 13.428751 8.635730 3.453798; 1 11.990585 10.257258 7.097108; 1 13.575928 9.703821 6.539297; 1 12.134315 8.724922 6.233271; 6 12.847500 10.874690 1.241953; 1 10.665198 13.871728 3.162259; 1 11.487626 13.366090 1.682310; 1 12.413501 14.084453 3.001074; 6 9.539885 8.711853 4.612257; 6 8.573252 8.979484 5.606887; 1 13.175042 7.889978 4.212144; 1 14.515521 8.796585 3.510421; 1 13.213056 8.201625 2.472255; 1 12.142429 11.417395 0.605070; 1 12.895475 9.845316 0.874472; 1 13.839552 11.327133 1.094401; 6 10.252068 7.495966 4.722027; 1 7.996915 9.893221 5.556193; 6 8.383448 8.112072 6.682311; 6 10.051085 6.620622 5.792145; 1 10.966996 7.237579 3.947247; 6 9.120273 6.927944 6.786156; 1 8.962619 6.251930 7.622326; 1 9.666643 12.092114 1.609482; 1 9.390738 8.967806 3.309219; 1 5.212002 11.465195 2.174142; 1 8.478460 12.303520 3.813350; 1 8.786414 10.288916 0.021260; 1 7.655716 8.366076 7.449876; 1 10.622203 5.696360 5.844349;
32	77 2.314316 4.393065 2.584673; 6 0.076633 4.914185 2.145719; 6 0.879392 5.953905 1.577757; 6 1.605270 6.591285 2.645091; 6 1.242999 5.944096 3.880111; 6 0.303004 4.896999 3.580881; 6 4.085351 3.818657 3.699918; 6 4.374868 4.698506 2.593170; 6 4.067178 4.114611 1.312216; 6 4.295574 2.660600 1.130979; 77 2.763158 1.567209 2.209073; 6 4.817073 1.908083 2.246813; 6 4.317358 2.365292 3.518680; 6 2.761243 -0.741562 2.167478; 6 1.832132 -0.352144 3.195683; 6 0.772889 0.386206 2.579286; 6 1.040619 0.464812 1.153824; 6 2.268359 -0.244339 0.908038; 6 2.871412 -0.513266 -0.441239; 6 0.085294 0.919426 0.087789; 6 -0.524395 0.715347 3.255440; 6 1.913378 -0.740091 4.644115; 6 3.968390 -1.614262 2.363454; 6 -0.503627 4.155287 4.607896; 6 1.681274 6.359894 5.255344; 6 2.493474 7.794498 2.502237; 6 0.891029 6.374899 0.136328; 6 -1.027273 4.211193 1.413598; 1 4.116253 4.743532 0.425719; 1 5.440476 1.025534 2.133053; 1 4.155563 4.213834 4.711438; 1 -0.744665 3.969299 0.385513; 1 -

	1.922441 4.849474 1.367617; 1 -1.319255 3.284360 1.909218; 1 0.657792 5.536576 -0.526576; 1 1.868525 6.768493 -0.161433; 1 0.149742 7.164673 -0.054946; 1 3.047272 7.779851 1.557417; 1 3.224481 7.858046 3.314561; 1 1.906256 8.723697 2.520531; 1 2.694163 6.776252 5.251610; 1 1.674724 5.512599 5.947531; 1 1.013509 7.129403 5.668610; 1 -0.953116 3.252261 4.193450; 1 -1.321313 4.782274 4.994597; 1 0.110496 3.853938 5.462596; 1 2.951618 -0.820375 4.982634; 1 1.435478 -1.714265 4.824413; 1 1.415000 -0.005250 5.283079; 1 4.716855 -1.453333 1.581010; 1 3.694760 -2.678697 2.336839; 1 4.451490 -1.428048 3.328608; 1 2.612892 0.273874 -1.155746; 1 2.510968 -1.466167 -0.854496; 1 3.964169 -0.571036 -0.394590; 1 0.611878 1.359687 -0.764949; 1 -0.611697 1.669488 0.463213; 1 -0.511126 0.076620 -0.293450; 1 -1.069881 1.501794 2.731952; 1 -0.375251 1.038134 4.289116; 1 -1.177180 -0.170229 3.278142; 1 4.536605 1.778370 4.408181; 1 4.505525 2.309240 0.122635; 1 4.694219 5.729559 2.717563;
TS for 32/benzene analog of 2	6 6.504093 6.786489 1.148289; 6 7.057297 7.598064 0.085323; 6 6.023169 8.518931 -0.322324; 6 4.828625 8.227265 0.436938; 6 5.123133 7.150463 1.333078; 6 7.176004 5.611649 1.799981; 1 8.262351 5.717504 1.793264; 1 6.929233 4.679318 1.272219; 1 6.860998 5.491767 2.841466; 6 8.365998 7.389436 -0.615909; 1 8.797197 8.335753 -0.954753; 1 8.244611 6.741539 -1.497178; 1 9.096883 6.914068 0.041830; 6 6.120351 9.485055 -1.469172; 1 5.468333 10.352205 -1.324510; 1 5.825952 9.005253 -2.413406; 1 7.142010 9.856646 -1.589634; 6 3.490906 8.887651 0.261171; 1 2.927165 8.915241 1.199298; 1 2.877198 8.347751 -0.474015; 1 3.593935 9.917320 -0.094106; 6 4.154652 6.494690 2.274990; 1 4.665085 6.069198 3.144309; 1 3.611365 5.675426 1.782111; 1 3.407345 7.204319 2.644961; 6 7.120410 11.070756 1.855905; 6 6.048575 10.776870 2.786739; 1 5.034572 11.163883 2.734922; 6 6.443655 9.804149 3.771817; 6 7.768502 9.225739 3.609460; 1 7.943773 8.393211 4.289139; 6 8.648251 11.202637 3.851652; 6 8.465214 11.446613 2.448898; 77 6.554994 8.974841 1.808430; 1 10.023607 6.317938 3.118461; 6 10.892951 6.630660 2.531391; 6 11.281474 8.048655 2.828193; 1 10.656326 6.485111 1.474647; 1 11.715235 5.944714 2.780643; 6 11.678652 9.040888 1.900721; 6 11.446878 8.566493 4.179343; 6 12.023686 10.228494 2.658608; 6 11.795781 8.911834 0.409732; 6 11.953130 9.894965 4.073858; 6 11.261265 7.768553 5.438083; 77 9.886280 9.984316 3.187518; 6 12.635529 11.473347 2.079948; 1 11.201340 8.079807 0.024612; 1 12.840041 8.738136 0.110385; 1 11.457932 9.819814 -0.099768; 6 12.414828 10.768087 5.205578; 1 10.386246 7.112319 5.374961; 1 11.120767 8.418710 6.307023; 1 12.131964 7.127845 5.643309; 1 12.190331 11.722868 1.111609; 1 13.717794 11.351524 1.925826; 1 12.491218 12.334731 2.739257; 1 11.865251 10.556371 6.127696; 1 12.278466 11.828953 4.976085; 1 13.483338 10.608191 5.410362; 1 8.870625 12.375563 2.041414; 1 6.850057 11.626147 0.958632; 1 5.740670 9.445530 4.521237; 1 8.249566 11.615133 4.779983;
TS for 30/benzene analog of 10	77 5.892629 2.348536 5.407714; 77 7.761356 2.321828 2.044014; 6 8.795243 3.043614 0.189652; 6 9.450018 1.791576 0.497437; 6 8.475952 0.749750 0.414944; 6 7.211790 1.354788 0.058063; 6 7.412413 2.767589 -0.142988; 6 6.302086 2.261947 7.627907; 6 6.021397 0.922425 7.176645; 6 4.639312 0.851362 6.756733; 6 4.076178 2.157828 6.887383; 6 5.104302 3.041566 7.390204; 6 6.421377 3.740735 -0.714167; 6 10.913520 1.613368 0.786398; 6 9.479124 4.372644 0.030833; 6 5.937141 0.608200 -0.217549; 6 8.728694 -0.718894 0.601508; 6 2.647820 2.535945 6.615337; 6 4.903420 4.473220 7.801618; 6 3.912406 -0.388832 6.321613; 6 7.543472 2.728136 8.332986; 6 6.961510 -0.245520 7.277421; 1 6.473644 3.769035 -1.813101; 1 6.602623 4.756576 -0.350098; 1 5.395174 3.475905 -0.441077; 1 11.343121 2.504784 1.253529; 1 11.480921 1.423043 -0.136115; 1 11.092573 0.768689 1.459581; 1 8.791077 5.200477 0.225794; 1 9.867588 4.496643 -0.990207; 1 10.323777 4.476887 0.718924; 1 5.844729 -0.280909 0.414740; 1 5.892778 0.273205 -1.263236; 1 5.059120 1.235741 -0.035778; 1 9.540023 -0.901850 1.313476; 1 9.011446 -1.199527 -0.346735; 1 7.839918 -1.237263 0.974418; 1 2.019351 2.381876 7.504428; 1 2.218614 1.937721 5.804980; 1 2.556840 3.588982 6.332206; 1 5.834687 5.042767 7.730355; 1 4.552564 4.538780 8.841539; 1 4.161354 4.974159 7.172223; 1 3.111528 -0.159221 5.611316; 1 3.452117 -0.903791 7.177794; 1 4.586234 -1.103180 5.838552; 1 8.428153 2.180241 7.993917; 1 7.464518 2.585596 9.421414; 1 7.730689 3.791165 8.153292; 1 6.762769 -0.991898 6.501406; 1 6.865007 -0.749053 8.249404; 1 8.004067 0.070580 7.172484; 6 5.994698 2.036922 3.133079; 6 5.383378 3.260655 3.606729; 6 7.490508 1.674835 4.233308; 6 6.423530 4.083652 4.177536; 6 8.494671 2.680896 3.959078; 6 7.812893 3.893332 3.571249; 1 8.387938 4.789933 3.346624; 1 4.340793 3.521823 3.448670; 1 7.779985 0.632736 4.347583; 1 5.372330 1.191285 2.849528; 1 6.179944 5.069055 4.570513; 1 9.558725 2.541735 4.128010;
benzene analog of 10	77 -0.725837 0.136105 1.480886; 77 0.741578 0.288389 -1.538570; 6 2.507387 0.377478 -3.140456; 6 2.447129 -0.936027 -2.554645; 6 1.154655 -1.494594 -2.833604; 6 0.409638 -0.512750 -3.618664; 6 1.259057 0.627520 -3.809290; 6 -0.128436 0.089733 3.652843; 6 -0.114237 -1.269761 3.116866; 6 -1.469572 -1.630084 2.811459; 6 -2.317522 -0.512566 3.135043; 6 -1.491756 0.536760 3.668890; 6 0.928602 1.847827 -4.620552; 6 3.575781 -1.630374 -1.846692; 6 3.706195 1.283904 -3.143467; 6 -0.922207 -0.752586 -4.271482; 6 0.716139 -2.905908 -2.564500; 6 -3.816610 -0.488430 3.030489; 6 -1.982476 1.850191 4.208010; 6 -1.938487 -2.969117 2.317312; 6 1.049242 0.797695 4.260461; 6 1.077101 -2.184478 3.098304; 1 1.275781 1.738692 -5.657979; 1 1.402412 2.745726 -4.211115; 1 -0.149713 2.028145 -4.650781; 1 4.190441 -0.925762 -1.277481; 1 4.237718 -2.137718 -2.563194; 1 3.205808 -2.385235 -1.147172; 1 3.415364 2.337379 -3.207848; 1 4.365149 1.076022 -3.998862; 1 4.306672 1.161970 -2.236043; 1 -1.578612 -1.352466 -3.633763; 1 -0.806712 -1.289215 -5.224304; 1 -1.441568 0.187356 -4.481221; 1 1.222501 -3.323478 -1.689073; 1 0.935767 -3.560450 -3.420508; 1 -0.360992 -2.963988 -2.378589; 1 -4.289923 -0.870137 3.946666; 1 -4.172993 -1.107484 2.200630; 1 -4.193593 0.526899 2.870390; 1 -1.215095 2.625734 4.131231; 1 -2.261174 1.763143 5.267932; 1 -2.865760 2.204387 3.666977; 1 -2.802479 -2.874652 1.651767; 1 -2.239063 -3.615180 3.154685; 1 -1.151609 -3.489800 1.764445; 1 1.973417 0.575457 3.718367; 1 1.198934 0.494914 5.306942; 1 0.915522 1.883560 4.245181; 1 0.966329 -2.972184 2.347060; 1 1.219646 -2.671384 4.073981; 1 1.998488 -1.640047 2.868832; 6 -1.026211 -0.329936 -0.668083; 6 -1.918296 0.685934 -0.213255; 6 1.125440 0.492262 0.636942; 6 -1.185369 1.852592 0.189369; 6 1.299273 1.728937 -0.052631; 6 0.055647 2.163967 -0.622068; 1 0.029725 3.047418 -1.260433; 1 -2.986331 0.548331 -0.046018; 1 2.010485 -0.013774 1.025147; 1 2.261006 2.200095 -0.254221; 1 -1.708861 2.691559 0.648461; 1 -1.435756 -1.313917 -0.900964;
benzene analog of 11	77 -0.430259 0.072190 1.365505; 77 0.396678 0.429294 -1.295747; 6 2.590878 0.283082 -2.307642; 6 2.336323 -0.960243 -1.663325; 6 1.104088 -1.504961 -2.192933; 6 0.618579 -0.602064 -3.220566; 6 1.526980 0.528460 -3.250482; 6 0.637125 -0.801161 3.279005; 6 0.108714 -1.967325 2.594202; 6 -1.300138 -1.839177 2.502805; 6 -1.674323 -0.567717 3.086177; 6 -0.461815 0.045882 3.615819; 6 1.480753 1.659701 -4.238579; 6 3.241211 -1.621971 -0.665906; 6 3.777595 1.172824 -2.071163; 6 -0.485616 -0.881941 -4.199856; 6 0.554066 -2.875086 -1.918032; 6 -3.078404 -0.113134 3.367741; 6 -0.408579 1.286340 4.461141; 6 -2.254417 -2.845563 1.926832; 6 2.071563 -0.607498 3.681236; 6 0.915755 -3.169487 2.200464; 1 2.082195 1.426110 -5.128396; 1 1.875229 2.587883 -3.812466; 1 0.458711 1.856950 -4.574179; 1 3.676201 -0.896868 0.028971; 1 4.072205 -2.133340 -1.172954; 1 2.707129 -2.369689 -0.076520; 1 3.537160 2.224070 -2.258232; 1 4.613784 0.908022 -2.734274; 1 4.139607 1.094447 -

	1.041214; 1 -1.264066 -1.507692 -3.754312; 1 -0.101733 -1.406268 -5.087321; 1 -0.964390 0.041310 -4.538614; 1 0.788954 -3.205378 -0.902683; 1 0.970832 -3.616240 -2.614626; 1 -0.534586 -2.895806 -2.027276; 1 -3.441223 -0.525204 4.321376; 1 -3.768954 -0.436103 2.583252; 1 -3.140753 0.976802 3.430150; 1 0.543603 1.814203 4.344482; 1 -0.520728 1.043347 5.527109; 1 -1.206300 1.985444 4.193440; 1 -3.115720 -2.358476 1.458780; 1 -2.641579 -3.519569 2.704171; 1 -1.775308 -3.467843 1.164193; 1 2.758393 -0.970207 2.909791; 1 2.304503 -1.148941 4.609948; 1 2.299737 0.448523 3.853511; 1 0.461335 -3.711873 1.366023; 1 0.995889 -3.875476 3.040129; 1 1.935022 -2.897080 1.912821; 6 -1.415667 -0.116741 -0.449011; 6 -2.501466 0.849598 -0.702087; 6 0.160683 1.849396 0.960688; 6 -2.155040 2.036279 -1.224623; 6 0.247034 2.410176 -0.348685; 6 -0.707622 2.241175 -1.452497; 1 -0.450958 2.859082 -2.315051; 1 -3.529768 0.602646 -0.433206; 1 0.730108 2.399446 1.724282; 1 1.050289 3.129667 -0.527595; 1 -1.719077 -1.161320 -0.600842; 1 -2.856173 2.848361 -1.410826;
benzene analog of 2	77 -0.498548 11.975002 5.427764; 77 -0.828568 9.442240 4.278322; 6 -1.394833 7.891947 2.697612; 6 -0.107155 11.343820 3.572146; 6 -0.360440 14.172370 5.696357; 6 -0.662048 7.645639 1.410111; 6 -2.574160 8.691388 2.854595; 6 0.039745 9.970129 6.000479; 6 -0.056883 6.195999 4.199180; 6 1.250337 10.747128 6.100512; 6 0.629734 13.743369 8.084422; 6 2.382514 10.608602 5.191058; 6 -1.089680 7.261908 3.967641; 6 -2.094471 7.701529 4.914352; 6 -0.427864 13.603110 7.027362; 6 0.914447 10.383314 3.234360; 6 -3.006670 8.575971 4.221236; 6 -1.713069 12.989447 7.199488; 6 2.219436 10.322546 3.883665; 6 -2.448062 13.156762 5.976427; 6 -1.619233 13.877638 5.041719; 6 0.725192 15.069074 5.172877; 6 -2.047155 14.386696 3.694859; 6 -2.228402 12.346459 8.455663; 6 -3.879060 12.766158 5.754211; 6 -2.252803 7.199587 6.321286; 6 -4.267828 9.153207 4.792413; 6 -3.276874 9.460501 1.772192; 1 -0.655934 11.781616 2.733025; 1 0.394604 7.425756 1.586995; 1 -0.713819 8.510833 0.741736; 1 -1.093840 6.789133 0.873448; 1 0.828507 6.354792 3.577120; 1 -0.457994 5.199162 3.962654; 1 0.276147 6.182657 5.240724; 1 1.533849 11.031522 7.116586; 1 0.621440 12.899097 8.780888; 1 0.473354 14.657792 8.673842; 1 1.629483 13.800071 7.644609; 1 3.079457 10.097573 3.255078; 1 1.704002 14.786681 5.570709; 1 0.540169 16.117186 5.451624; 1 0.791550 15.021159 4.082343; 1 -1.191750 14.496943 3.021684; 1 -2.533821 15.368743 3.776584; 1 -2.756820 13.705680 3.214278; 1 -2.924539 11.531125 8.234375; 1 -2.762052 13.071140 9.087421; 1 -1.414523 11.929272 9.056363; 1 -4.081597 12.566183 4.698399; 1 -4.555296 13.572938 6.072388; 1 -4.147097 11.870575 6.321295; 1 -1.285755 6.940534 6.762400; 1 -2.884176 6.300535 6.353476; 1 -2.717227 7.951551 6.967193; 1 -4.179295 9.328742 5.868186; 1 -5.112384 8.465527 4.639754; 1 -4.529335 10.103385 4.319138; 1 -3.765783 10.356135 2.168553; 1 -4.052220 8.851403 1.285495; 1 -2.580640 9.784609 0.992698; 1 3.380022 10.681552 5.621200; 1 0.930164 10.076396 2.185923; 1 -0.385404 9.612906 6.942980;

8. Kinetic modeling of thermolysis of complex 1 (0.1 M) at 50 °C (Fig. 1B) using computed activation parameters (Fig. 2).

Table S17. Calculated time dependence of concentrations of **1**, **2** and all intermediates.

50 C Reaction time, s	Concentrations, M										
	**1**	**7**	**8**	**9**	**10**	**11**	**12**	**13**	**15**	**2**	**mesitylene**
0	0.1	0	0	0	0	0	0	0	0	0	0
0.00242406	0.1	2.33E-11	4.41E-08	7.56584E-09	1.48E-13	4.38819E-08	9.48E-08	1.01E-08	1.39E-12	2.48E-11	7.82E-15
0.00407301	0.1	6.17E-11	9.87E-08	1.14917E-08	3.92E-13	6.6652E-08	1.44E-07	1.72E-08	3.17E-12	6.52E-11	2.27E-14
0.00572195	0.099999	1.31E-10	1.73E-07	1.45066E-08	8.34E-13	8.41381E-08	1.82E-07	2.33E-08	5.66E-12	1.38E-10	5.50E-14
0.00704998	0.099999	2.12E-10	2.43E-07	1.64467E-08	1.35E-12	9.5391E-08	2.06E-07	2.77E-08	8.08E-12	2.22E-10	9.80E-14
0.008378	0.099999	3.21E-10	3.21E-07	1.80099E-08	2.04E-12	1.04458E-07	2.26E-07	3.15E-08	1.08E-11	3.34E-10	1.64E-13
0.00970603	0.099999	4.60E-10	4.06E-07	1.92716E-08	2.92E-12	1.11775E-07	2.41E-07	3.50E-08	1.39E-11	4.77E-10	2.58E-13
0.01261835	0.099998	8.65E-10	6.07E-07	2.13534E-08	5.48E-12	1.2385E-07	2.68E-07	4.18E-08	2.13E-11	8.92E-10	5.87E-13
0.01553068	0.099998	1.43E-09	8.24E-07	2.26516E-08	9.03E-12	1.31379E-07	2.84E-07	4.77E-08	2.97E-11	1.47E-09	1.14E-12
0.01844301	0.099997	2.17E-09	1.05E-06	2.34323E-08	1.37E-11	1.35908E-07	2.94E-07	5.29E-08	3.89E-11	2.22E-09	2.00E-12
0.02135534	0.099997	3.09E-09	1.29E-06	2.38946E-08	1.95E-11	1.38589E-07	2.99E-07	5.78E-08	4.88E-11	3.16E-09	3.25E-12
0.0261677	0.099996	5.02E-09	1.68E-06	2.43109E-08	3.15E-11	1.41003E-07	3.05E-07	6.53E-08	6.66E-11	5.13E-09	6.45E-12
0.03098006	0.099995	7.48E-09	2.08E-06	2.44752E-08	4.68E-11	1.41956E-07	3.07E-07	7.25E-08	8.59E-11	7.62E-09	1.14E-11
0.03579241	0.099994	1.05E-08	2.49E-06	2.45369E-08	6.53E-11	1.42314E-07	3.07E-07	7.96E-08	1.07E-10	1.06E-08	1.83E-11
0.04060477	0.099994	1.40E-08	2.89E-06	2.45621E-08	8.69E-11	1.4246E-07	3.08E-07	8.67E-08	1.29E-10	1.42E-08	2.78E-11
0.05581332	0.099991	2.85E-08	4.16E-06	2.46014E-08	1.76E-10	1.42688E-07	3.08E-07	1.09E-07	2.08E-10	2.90E-08	8.03E-11
0.07102186	0.099988	4.82E-08	5.42E-06	2.46069E-08	2.96E-10	1.4272E-07	3.08E-07	1.31E-07	3.01E-10	4.90E-08	1.74E-10
0.08623041	0.099986	7.32E-08	6.68E-06	2.46172E-08	4.45E-10	1.4278E-07	3.08E-07	1.53E-07	4.08E-10	7.43E-08	3.20E-10
0.10143895	0.099983	1.03E-07	7.94E-06	2.46316E-08	6.25E-10	1.42863E-07	3.09E-07	1.75E-07	5.28E-10	1.05E-07	5.31E-10
0.15031025	0.099975	2.34E-07	1.19E-05	2.46764E-08	1.40E-09	1.43123E-07	3.09E-07	2.44E-07	1.01E-09	2.39E-07	1.78E-09
0.19918155	0.099966	4.17E-07	1.59E-05	2.47181E-08	2.47E-09	1.43365E-07	3.10E-07	3.13E-07	1.62E-09	4.26E-07	4.19E-09
0.24805285	0.099958	6.51E-07	1.98E-05	2.4759E-08	3.84E-09	1.43602E-07	3.10E-07	3.81E-07	2.37E-09	6.65E-07	8.14E-09
0.29692415	0.099949	9.34E-07	2.36E-05	2.48003E-08	5.48E-09	1.43842E-07	3.11E-07	4.47E-07	3.26E-09	9.57E-07	1.40E-08
0.34579545	0.099941	1.27E-06	2.74E-05	2.48411E-08	7.40E-09	1.44078E-07	3.11E-07	5.13E-07	4.27E-09	1.30E-06	2.21E-08
0.58620242	0.0999	3.57E-06	4.53E-05	2.50335E-08	2.07E-08	1.45194E-07	3.14E-07	8.25E-07	1.10E-08	3.71E-06	1.06E-07
0.8266094	0.099858	6.92E-06	6.21E-05	2.52127E-08	3.99E-08	1.46234E-07	3.16E-07	1.12E-06	2.05E-08	7.27E-06	2.90E-07
1.06701637	0.099817	1.12E-05	7.78E-05	2.53816E-08	6.44E-08	1.47213E-07	3.18E-07	1.39E-06	3.25E-08	1.19E-05	6.11E-07
1.30742334	0.099776	1.63E-05	9.26E-05	2.55399E-08	9.37E-08	1.48131E-07	3.20E-07	1.65E-06	4.67E-08	1.75E-05	1.10E-06
1.54783031	0.099735	2.22E-05	0.000106	2.56882E-08	1.27E-07	1.48991E-07	3.22E-07	1.89E-06	6.28E-08	2.41E-05	1.78E-06
2.07983119	0.099644	3.73E-05	0.000134	2.59841E-08	2.14E-07	1.50708E-07	3.26E-07	2.37E-06	1.04E-07	4.18E-05	4.10E-06
2.61183207	0.099552	5.49E-05	0.000158	2.62408E-08	3.15E-07	1.52197E-07	3.29E-07	2.78E-06	1.52E-07	6.31E-05	7.72E-06
3.14383295	0.099461	7.43E-05	0.000178	2.64632E-08	4.25E-07	1.53487E-07	3.32E-07	3.14E-06	2.05E-07	8.77E-05	1.28E-05
3.67583383	0.09937	9.49E-05	0.000196	2.6656E-08	5.43E-07	1.54605E-07	3.34E-07	3.45E-06	2.61E-07	0.000115	1.94E-05
4.20783471	0.09928	0.000116	0.000211	2.68231E-08	6.65E-07	1.55574E-07	3.36E-07	3.71E-06	3.19E-07	0.000145	2.77E-05
4.73983559	0.099189	0.000138	0.000224	2.6968E-08	7.89E-07	1.56414E-07	3.38E-07	3.94E-06	3.77E-07	0.000177	3.77E-05
5.83705301	0.099002	0.000183	0.000246	2.72088E-08	1.05E-06	1.57811E-07	3.41E-07	4.33E-06	4.99E-07	0.000248	6.37E-05
6.93427044	0.098815	0.000226	0.000262	2.73883E-08	1.29E-06	1.58852E-07	3.43E-07	4.61E-06	6.16E-07	0.000325	9.68E-05
8.03148786	0.098629	0.000267	0.000274	2.75219E-08	1.53E-06	1.59627E-07	3.45E-07	4.81E-06	7.25E-07	0.000406	0.00013672

9.12870528	0.098443	0.000305	0.000283	2.76213E-08	1.74E-06	1.60204E-07	3.46E-07	4.96E-06	8.26E-07	0.00049	0.00018299
10.2259227	0.098258	0.000339	0.000289	2.76953E-08	1.93E-06	1.60633E-07	3.47E-07	5.07E-06	9.18E-07	0.000577	0.00023503
11.3231401	0.098072	0.000369	0.000294	2.77504E-08	2.11E-06	1.60952E-07	3.48E-07	5.15E-06	1.00E-06	0.000665	0.00029229
13.1665062	0.097762	0.000413	0.000299	2.78135E-08	2.36E-06	1.61318E-07	3.48E-07	5.23E-06	1.12E-06	0.000815	0.00039868
15.0098722	0.097453	0.000448	0.000301	2.7852E-08	2.56E-06	1.61542E-07	3.49E-07	5.28E-06	1.21E-06	0.000967	0.00051567
16.8532382	0.097144	0.000475	0.000302	2.78753E-08	2.71E-06	1.61676E-07	3.49E-07	5.30E-06	1.28E-06	0.00112	0.00064104
18.6966042	0.096837	0.000496	0.000303	2.78893E-08	2.83E-06	1.61758E-07	3.49E-07	5.30E-06	1.34E-06	0.001273	0.00077297
20.5399703	0.09653	0.000513	0.000302	2.78977E-08	2.93E-06	1.61807E-07	3.50E-07	5.30E-06	1.38E-06	0.001427	0.00090997
22.3833363	0.096225	0.000525	0.000302	2.79029E-08	3.00E-06	1.61837E-07	3.50E-07	5.29E-06	1.42E-06	0.00158	0.00105083
25.7673675	0.095667	0.00054	0.000301	2.79083E-08	3.08E-06	1.61868E-07	3.50E-07	5.27E-06	1.46E-06	0.001861	0.00131633
29.1513987	0.095111	0.000548	0.000299	2.79102E-08	3.13E-06	1.61879E-07	3.50E-07	5.24E-06	1.48E-06	0.00214	0.00158734
32.5354299	0.09456	0.000551	0.000297	2.79105E-08	3.15E-06	1.61881E-07	3.50E-07	5.21E-06	1.49E-06	0.002417	0.0018612
35.9194611	0.094011	0.000552	0.000296	2.79107E-08	3.15E-06	1.61882E-07	3.50E-07	5.18E-06	1.49E-06	0.002693	0.00213624
39.3034923	0.093466	0.000552	0.000294	2.79109E-08	3.15E-06	1.61883E-07	3.50E-07	5.15E-06	1.49E-06	0.002968	0.00241137
42.6875235	0.092923	0.00055	0.000292	2.79108E-08	3.14E-06	1.61883E-07	3.50E-07	5.12E-06	1.49E-06	0.003241	0.00268592
48.0198746	0.092075	0.000547	0.00029	2.79111E-08	3.12E-06	1.61885E-07	3.50E-07	5.08E-06	1.48E-06	0.003667	0.00311637
53.3522257	0.091235	0.000542	0.000287	2.79112E-08	3.09E-06	1.61885E-07	3.50E-07	5.03E-06	1.46E-06	0.00409	0.00354362
58.6845768	0.090402	0.000537	0.000284	2.79107E-08	3.07E-06	1.61882E-07	3.50E-07	4.98E-06	1.45E-06	0.004509	0.00396738
64.0169279	0.089577	0.000533	0.000282	2.79107E-08	3.04E-06	1.61882E-07	3.50E-07	4.94E-06	1.44E-06	0.004925	0.00438746
69.349279	0.088759	0.000528	0.000279	2.79112E-08	3.01E-06	1.61885E-07	3.50E-07	4.89E-06	1.42E-06	0.005336	0.00480375
74.6816301	0.087949	0.000523	0.000277	2.7911E-08	2.99E-06	1.61884E-07	3.50E-07	4.85E-06	1.41E-06	0.005744	0.00521623
85.813436	0.086282	0.000513	0.000271	2.79108E-08	2.93E-06	1.61883E-07	3.50E-07	4.76E-06	1.39E-06	0.006583	0.00606527
96.9452419	0.084646	0.000503	0.000266	2.79104E-08	2.87E-06	1.6188E-07	3.50E-07	4.67E-06	1.36E-06	0.007406	0.00689823
108.077048	0.083041	0.000494	0.000261	2.79107E-08	2.82E-06	1.61882E-07	3.50E-07	4.58E-06	1.33E-06	0.008213	0.0077154
119.208854	0.081467	0.000484	0.000256	2.79114E-08	2.77E-06	1.61886E-07	3.50E-07	4.49E-06	1.31E-06	0.009006	0.00851708
130.34066	0.079922	0.000475	0.000251	2.79111E-08	2.71E-06	1.61884E-07	3.50E-07	4.41E-06	1.28E-06	0.009783	0.00930356
188.918711	0.072265	0.00043	0.000227	2.79065E-08	2.45E-06	1.61858E-07	3.50E-07	3.98E-06	1.16E-06	0.013636	0.01320277
247.496763	0.065341	0.000389	0.000206	2.78717E-08	2.22E-06	1.61656E-07	3.49E-07	3.60E-06	1.05E-06	0.01712	0.01672815
306.074815	0.059081	0.000351	0.000186	2.78577E-08	2.01E-06	1.61574E-07	3.49E-07	3.26E-06	9.49E-07	0.02027	0.01991577
364.652867	0.053421	0.000318	0.000168	2.7915E-08	1.81E-06	1.61907E-07	3.50E-07	2.94E-06	8.58E-07	0.023118	0.02279807
423.230918	0.048302	0.000287	0.000152	2.80077E-08	1.64E-06	1.62444E-07	3.51E-07	2.66E-06	7.76E-07	0.025694	0.02540427
567.418671	0.03771	0.000224	0.000119	2.80168E-08	1.28E-06	1.62497E-07	3.51E-07	2.08E-06	6.05E-07	0.031024	0.03079772
711.606423	0.029448	0.000175	9.26E-05	2.74449E-08	1.00E-06	1.59181E-07	3.44E-07	1.62E-06	4.73E-07	0.035182	0.03500498
855.794175	0.022994	0.000137	7.23E-05	2.63672E-08	7.81E-07	1.5293E-07	3.30E-07	1.27E-06	3.69E-07	0.038429	0.03829128
999.981927	0.017951	0.000107	5.65E-05	2.54373E-08	6.09E-07	1.47536E-07	3.19E-07	9.90E-07	2.88E-07	0.040967	0.04085895
1144.16968	0.014013	8.33E-05	4.41E-05	2.56964E-08	4.76E-07	1.49039E-07	3.22E-07	7.72E-07	2.25E-07	0.042948	0.04286417
1288.35743	0.010939	6.51E-05	3.44E-05	2.81743E-08	3.71E-07	1.63411E-07	3.53E-07	6.03E-07	1.76E-07	0.044495	0.04442941
1433.5286	0.008526	5.07E-05	2.68E-05	3.29823E-08	2.89E-07	1.91297E-07	4.13E-07	4.70E-07	1.37E-07	0.045709	0.04565833
1578.69977	0.006645	3.95E-05	2.09E-05	3.86297E-08	2.26E-07	2.24052E-07	4.84E-07	3.66E-07	1.07E-07	0.046656	0.04661599
1723.87093	0.005179	3.08E-05	1.63E-05	4.2061E-08	1.76E-07	2.43954E-07	5.27E-07	2.85E-07	8.31E-08	0.047393	0.04736242
1869.0421	0.004036	2.40E-05	1.27E-05	3.92578E-08	1.37E-07	2.27695E-07	4.92E-07	2.23E-07	6.48E-08	0.047969	0.0479443
2014.21327	0.003146	1.87E-05	9.90E-06	2.63142E-08	1.07E-07	1.52622E-07	3.30E-07	1.74E-07	5.06E-08	0.048417	0.04839801
2162.15516	0.002439	1.45E-05	7.69E-06	1.71847E-10	8.30E-08	9.96713E-10	2.15E-09	1.35E-07	3.92E-08	0.048773	0.04875809
2310.09705	0.00189	1.13E-05	5.97E-06	0	6.44E-08	0	-4.71E-07	1.05E-07	3.05E-08	0.049049	0.04903788
2310.25343	0.00189	1.13E-05	5.72E-06	0	6.43E-08	0	-3.16E-07	9.88E-08	3.04E-08	0.04905	0.04903814

2310.37472	0.00189	1.13E-05	5.42E-06	0	6.43E-08	0	-1.20E-07	9.40E-08	3.04E-08	0.04905	0.04903834
2310.496	0.00189	1.12E-05	5.17E-06	3.58391E-09	6.41E-08	2.07867E-08	4.49E-08	9.01E-08	3.03E-08	0.04905	0.04903855
2310.61729	0.001889	1.12E-05	5.03E-06	1.2071E-08	6.40E-08	7.00117E-08	1.51E-07	8.78E-08	3.02E-08	0.04905	0.04903875
2310.73857	0.001889	1.12E-05	4.96E-06	1.7007E-08	6.38E-08	9.86405E-08	2.13E-07	8.68E-08	3.01E-08	0.04905	0.04903895

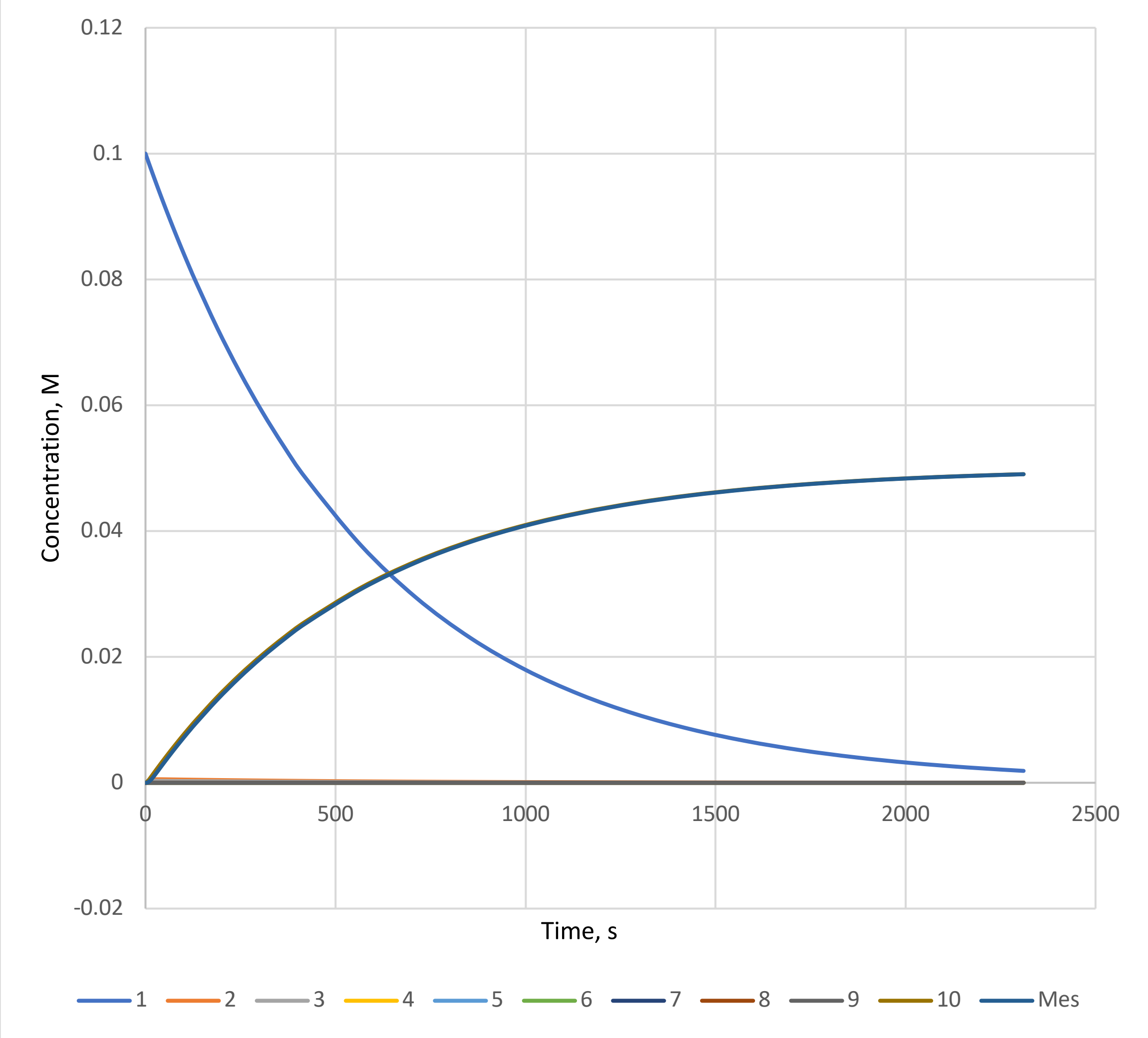

Figure S13. Calculated time dependence of concentrations of **1**, **2, Mes** and all intermediates (Table S17).

Table S18. Calculated initial rates for thermolysis of **1** (0.01- 0.1 M) without added mesitylene at 50 °C (from Table S17).

c(**1**), M	Initial rate, s^{-1}
0.01	$1.71 \cdot 10^{-4}$
0.025	$4.26 \cdot 10^{-4}$
0.05	$8.56 \cdot 10^{-4}$
0.1	$1.71 \cdot 10^{-3}$

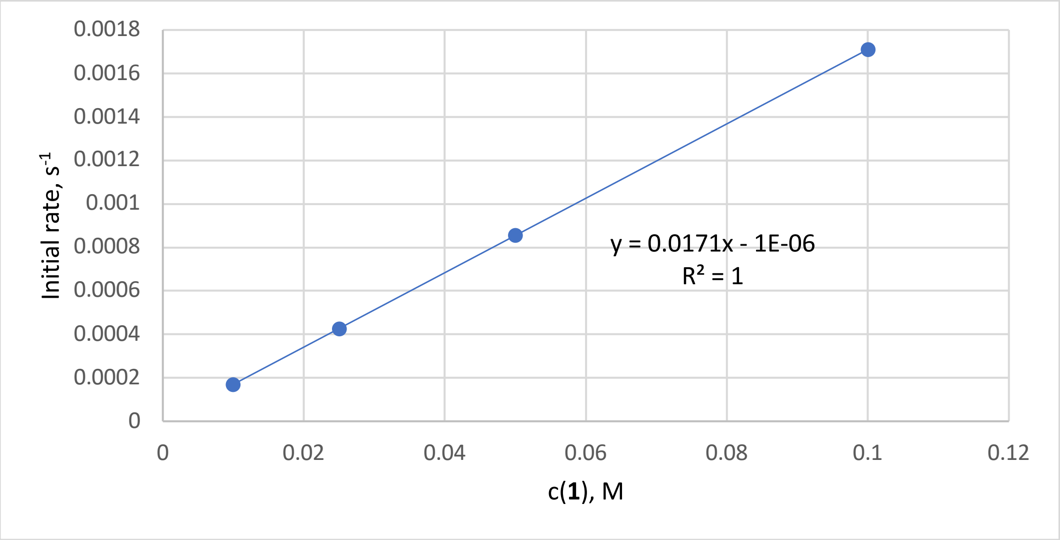

Figure S14. Calculated plot of initial rate vs concentration of **1** (Table S11).

Table S19. Calculated initial rates for thermolysis of **1** (0.1 M) in the presence of various concentration of mesitylene (0-1M) at 50 °C.

c(mes), M	Initial rate, s^{-1}
0	$1.71 \cdot 10^{-3}$
0.05	$1.71 \cdot 10^{-3}$
0.5	$1.71 \cdot 10^{-3}$
1	$1.71 \cdot 10^{-3}$

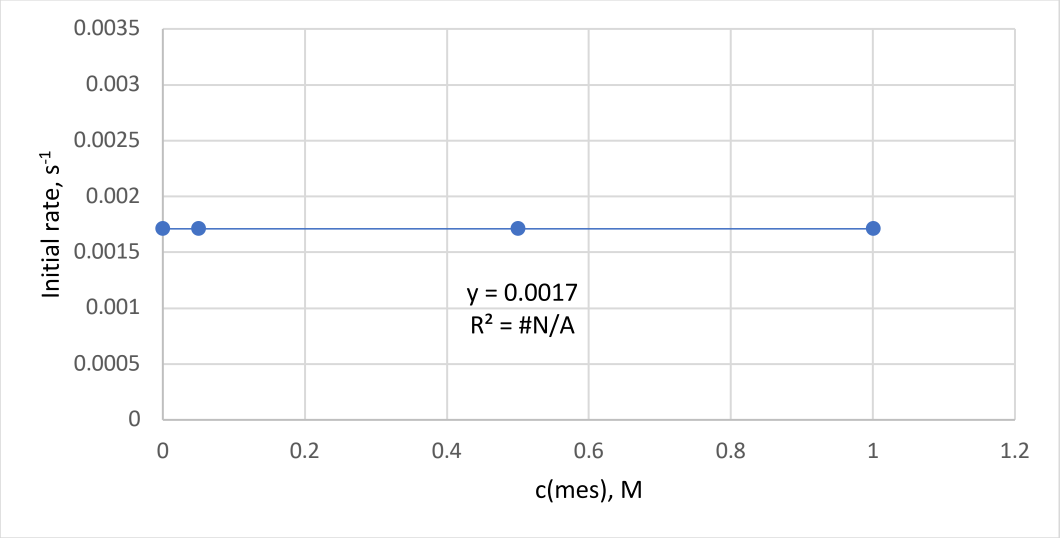

Figure S15. Calculated plot of initial rate vs concentration of mesitylene (Table S13).

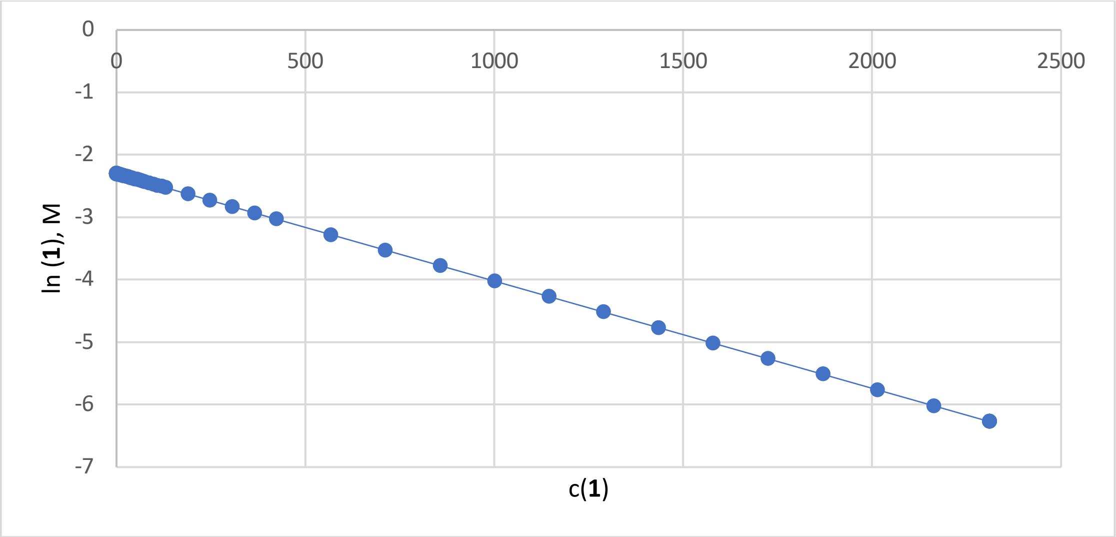

Figure S16. Calculated plot of ln c(**1**) vs time for thermolysis of **1** at 50 °C (Table S10).

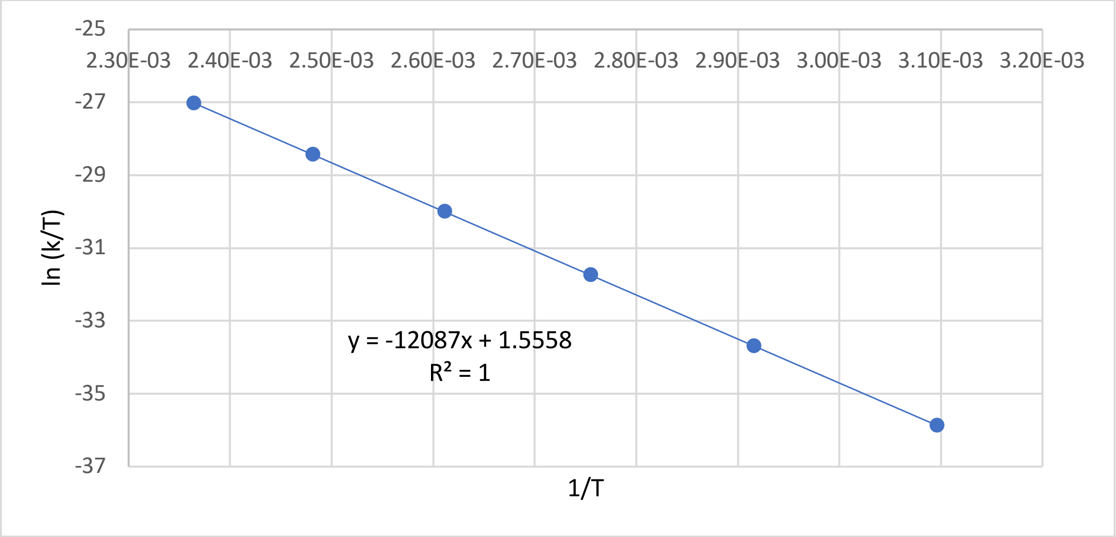

Figure S17. Calculated Eyring plot for determining the activation parameters $\Delta H^{\neq}$ and $\Delta S^{\neq}$ for thermolysis of **1** at 50-150 °C.

Table S20. Calculated activation parameters for thermolysis of **1** (Fig. S17).

$\Delta H^{\neq}$, kcal/mol	$\Delta S^{\neq}$, cal/(mol·K)	$\Delta G_{323}^{\neq}$, kcal/mol
24.00	3.09	23.01

9. ^{1}H and ^{13}C NMR spectra

^{1}H NMR spectrum of **mesitylene-d$_3$** in $CDCl_3$.

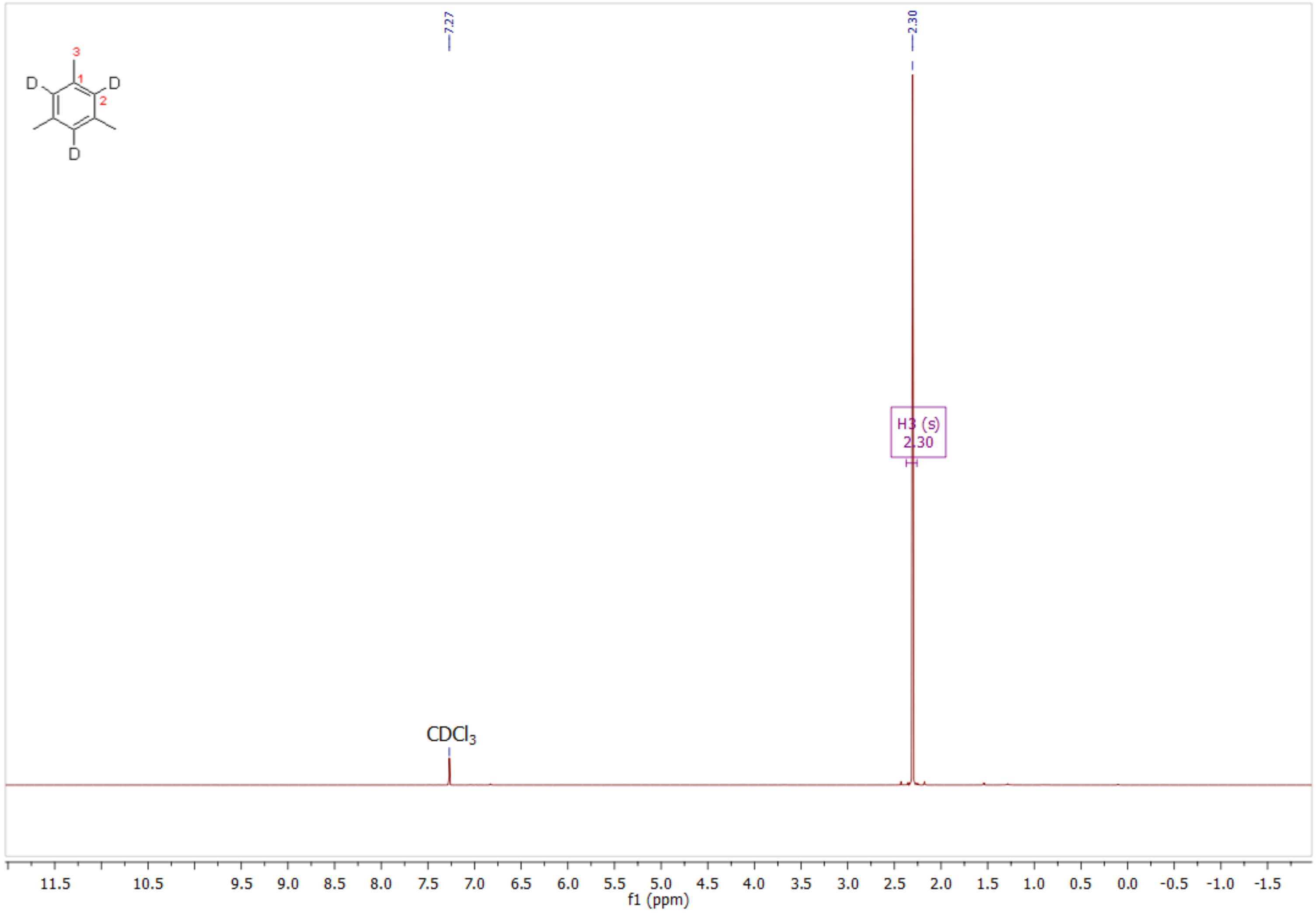

^{2}H NMR spectrum of **mesitylene-d$_3$** in $CHCl_3$.

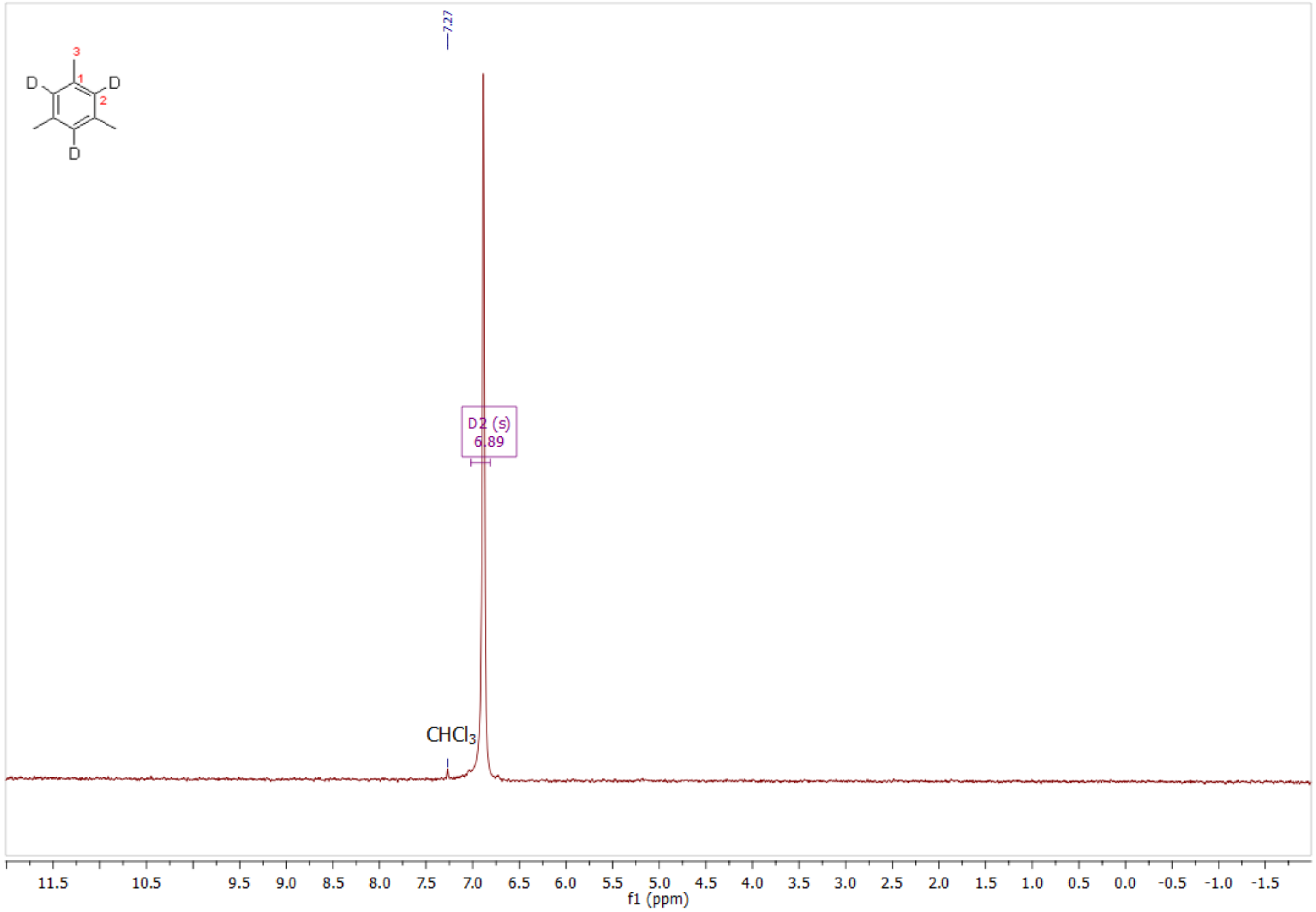

^{1}H NMR spectrum of **mesitylene-d$_9$** in $CDCl_3$.

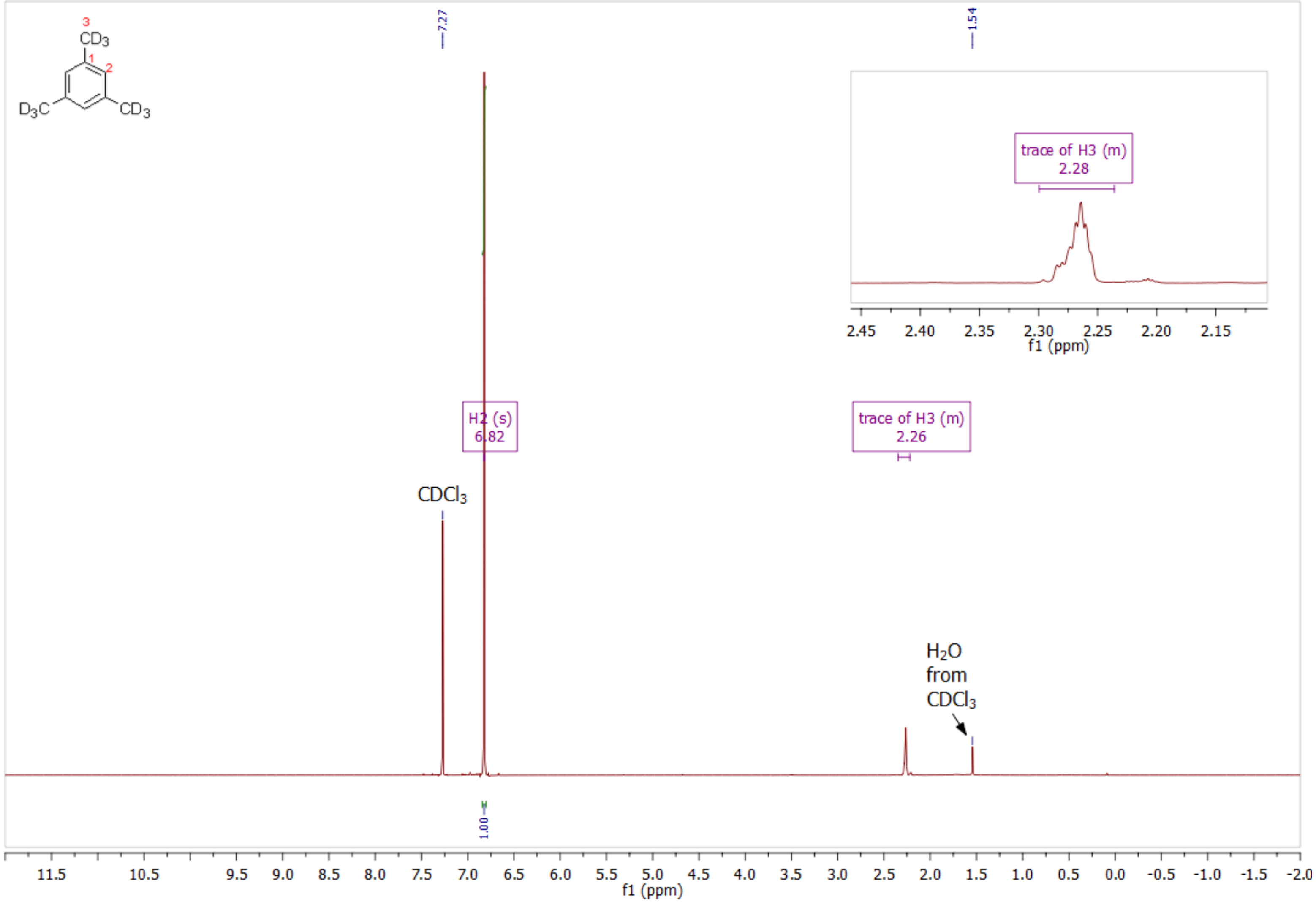

^{2}H NMR spectrum of **mesitylene-d$_3$** in $CHCl_3$.

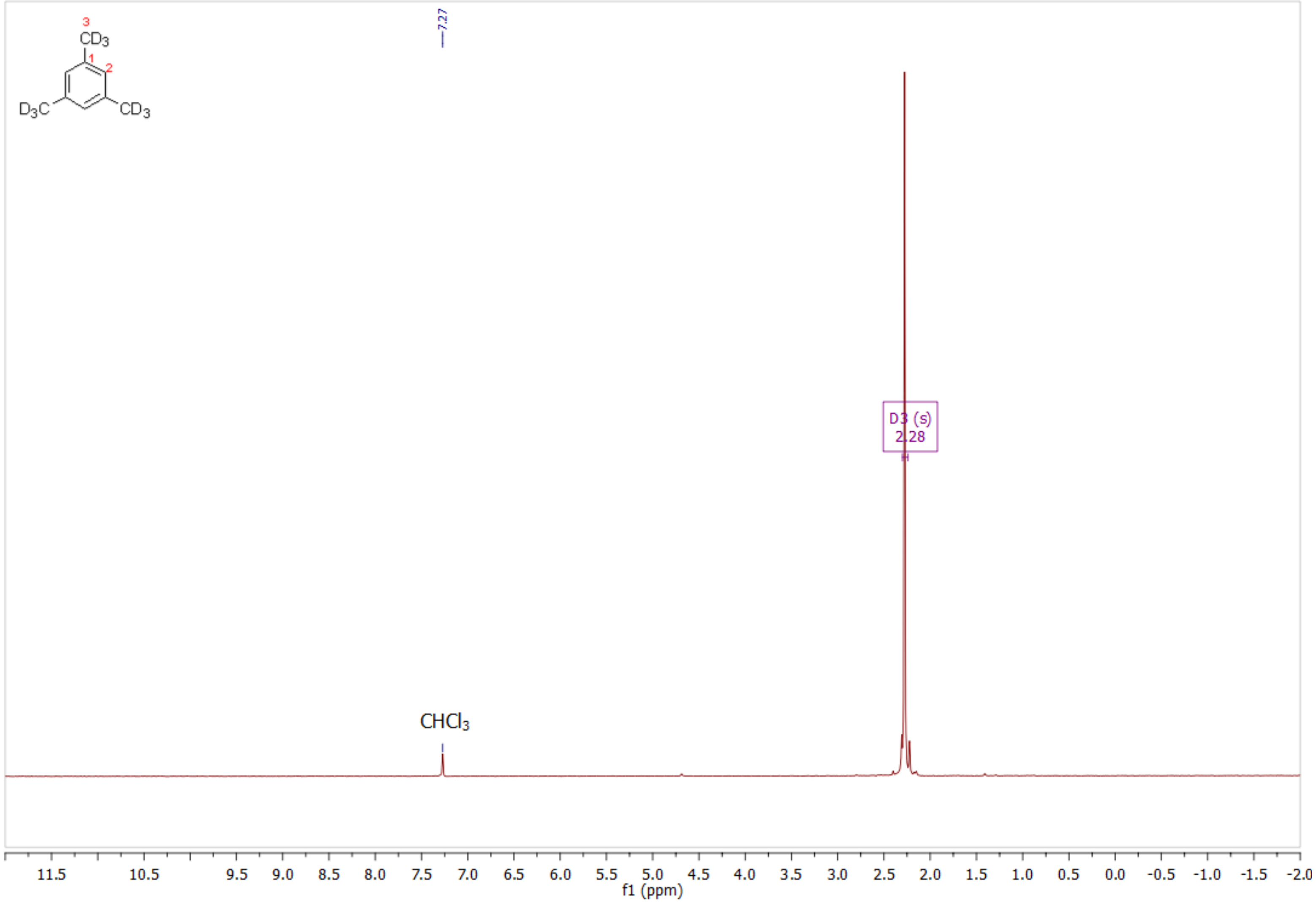

^{1}H NMR spectrum of **[Cp*Ir(η[6]-mesitylene-d$_3$)][BF$_4$]$_2$** in TFA-d.

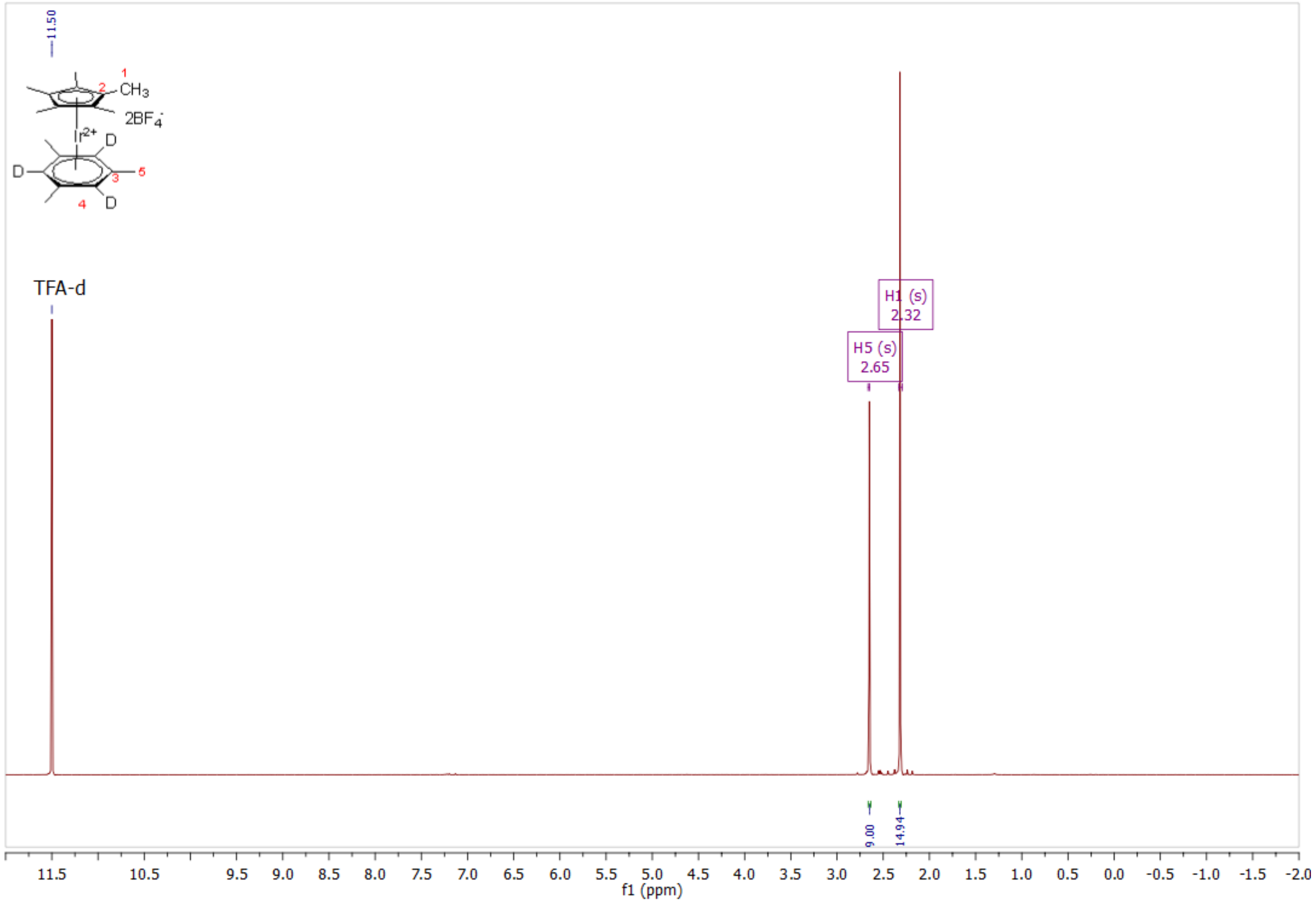

^{2}H NMR spectrum of **mesitylene-d$_3$** in TFA.

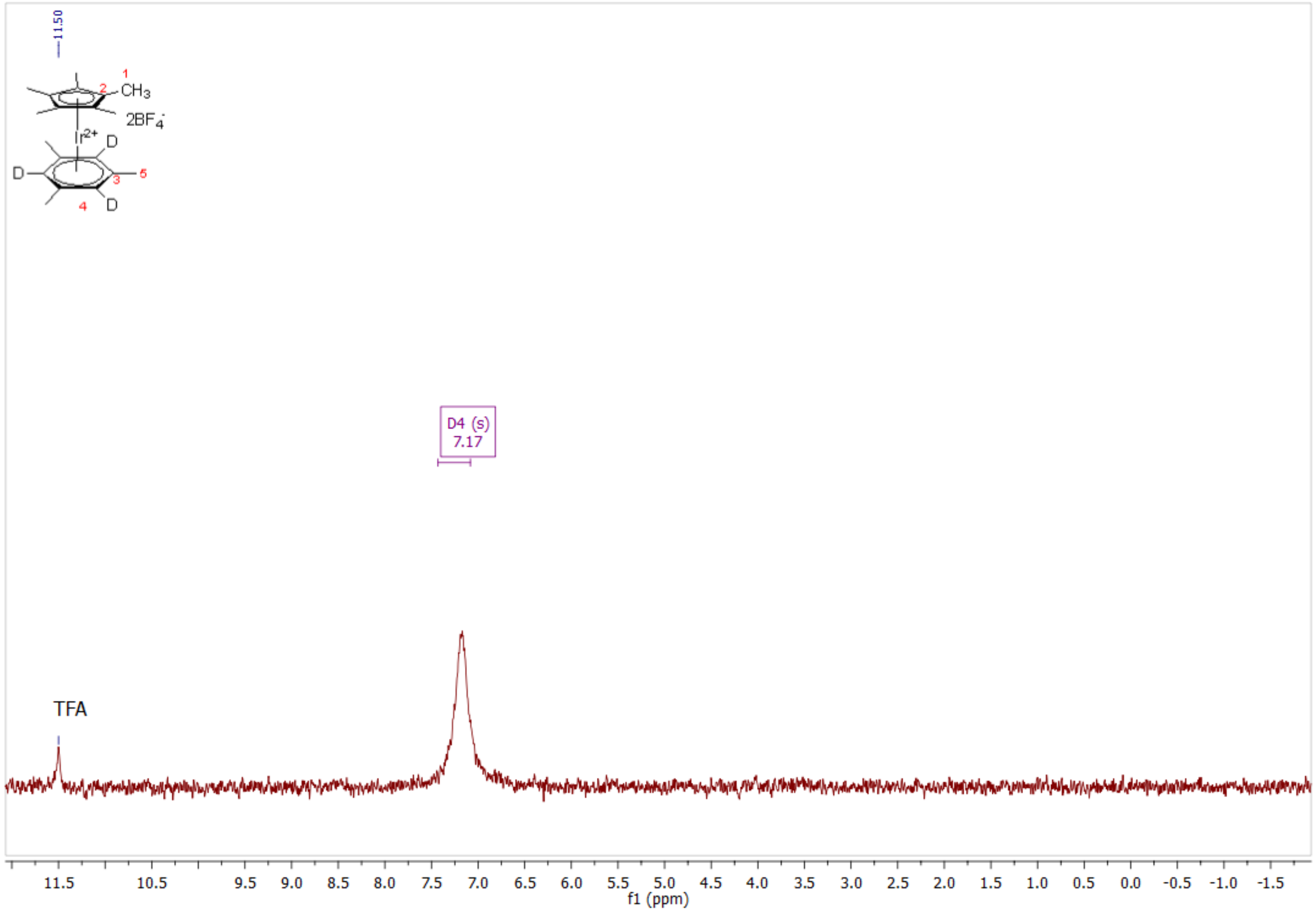

^{1}H NMR spectrum of **[Cp*Ir(η[6]-mesitylene-d3)][BF4]2** in TFA-d.

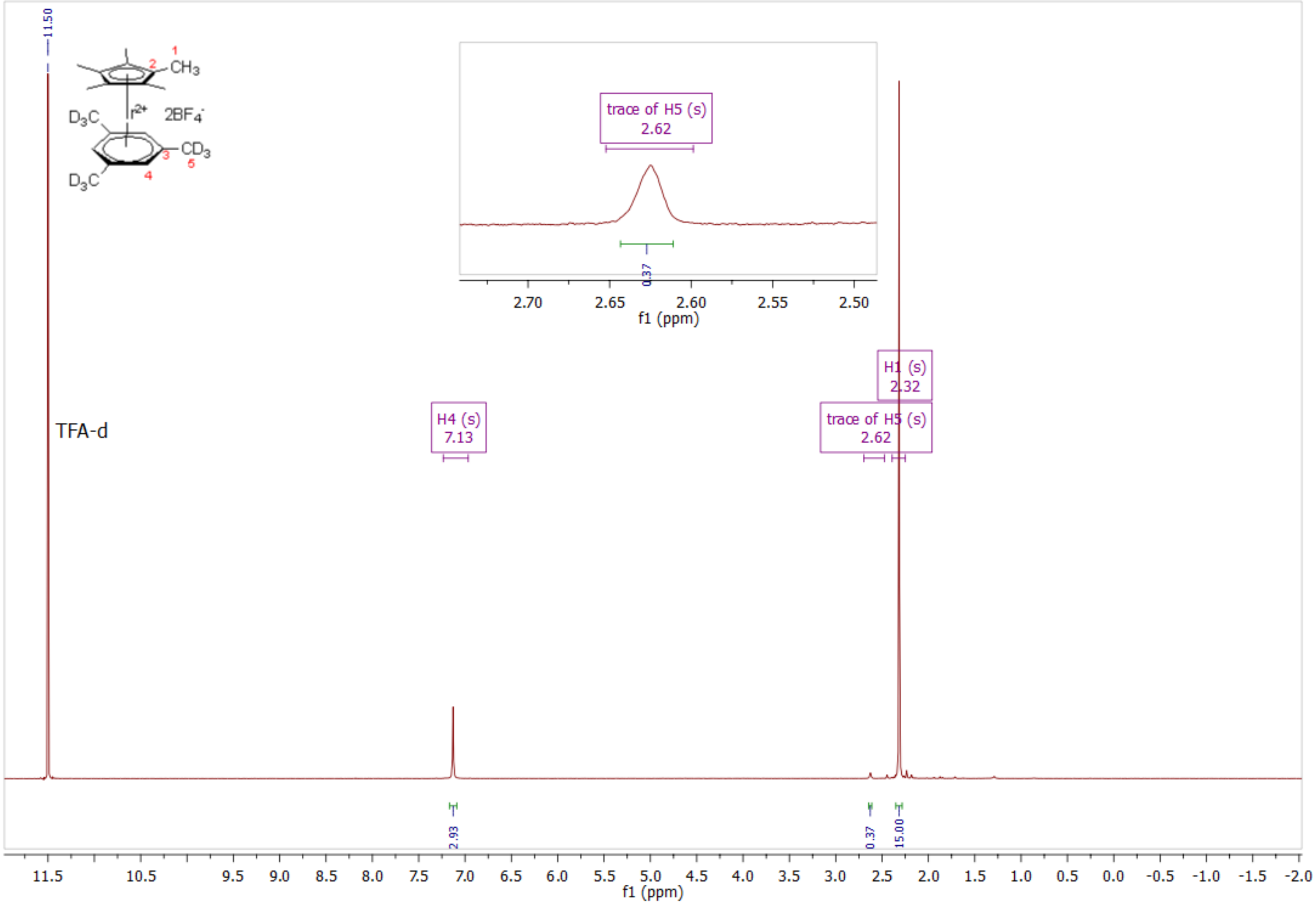

^{1}H NMR spectrum of **[Cp*Ir(η[6]-C_6Me_6)][BF_4]$_2$** in TFA-d.

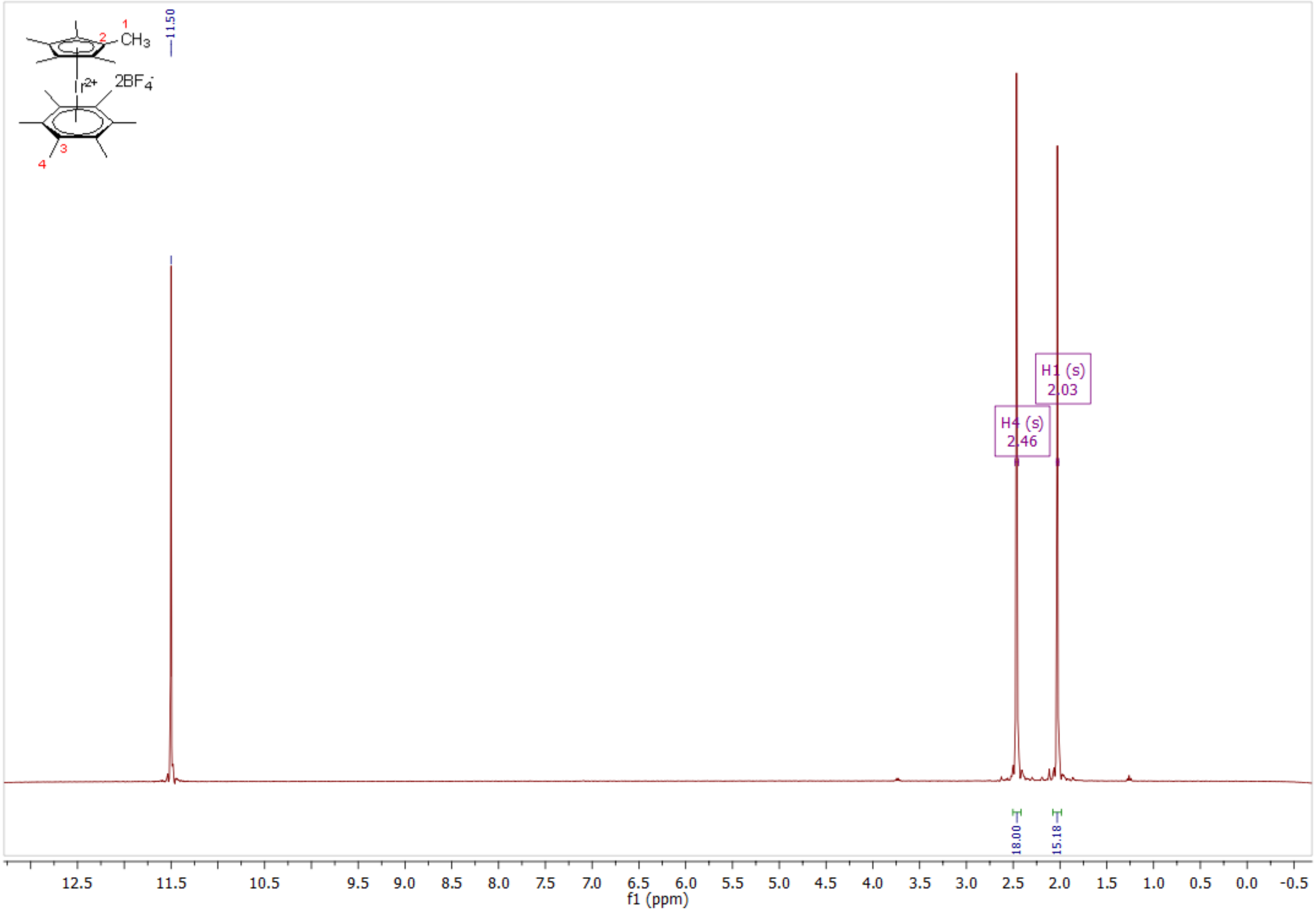

^{13}C NMR spectrum of **[Cp*Ir(η[6]-C_6Me_6)][BF_4]$_2$** in TFA-d.

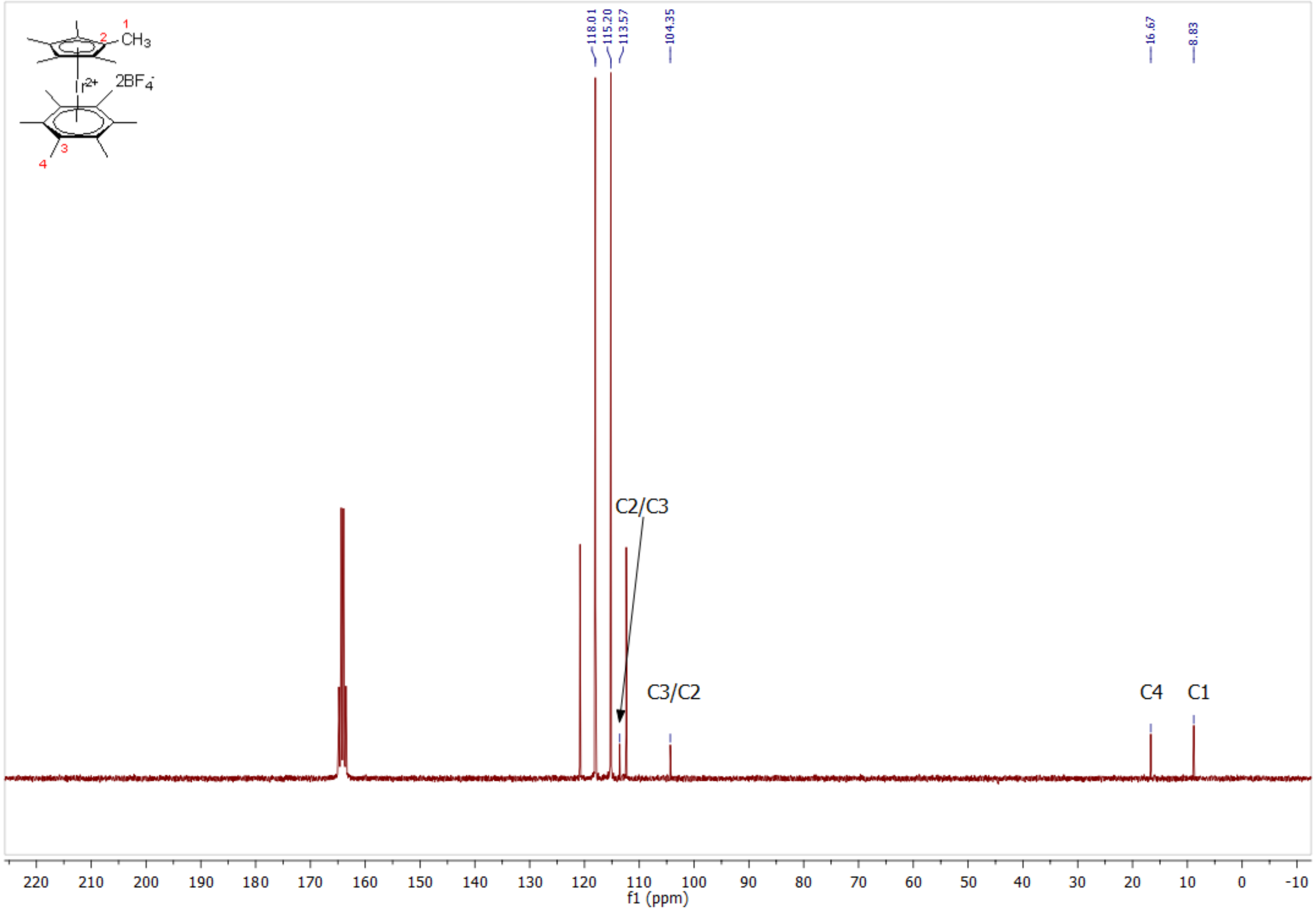

^{1}H NMR spectrum of **[Cp*Ir(η[6]-naphthalene)][BF4]2** in TFA-d.

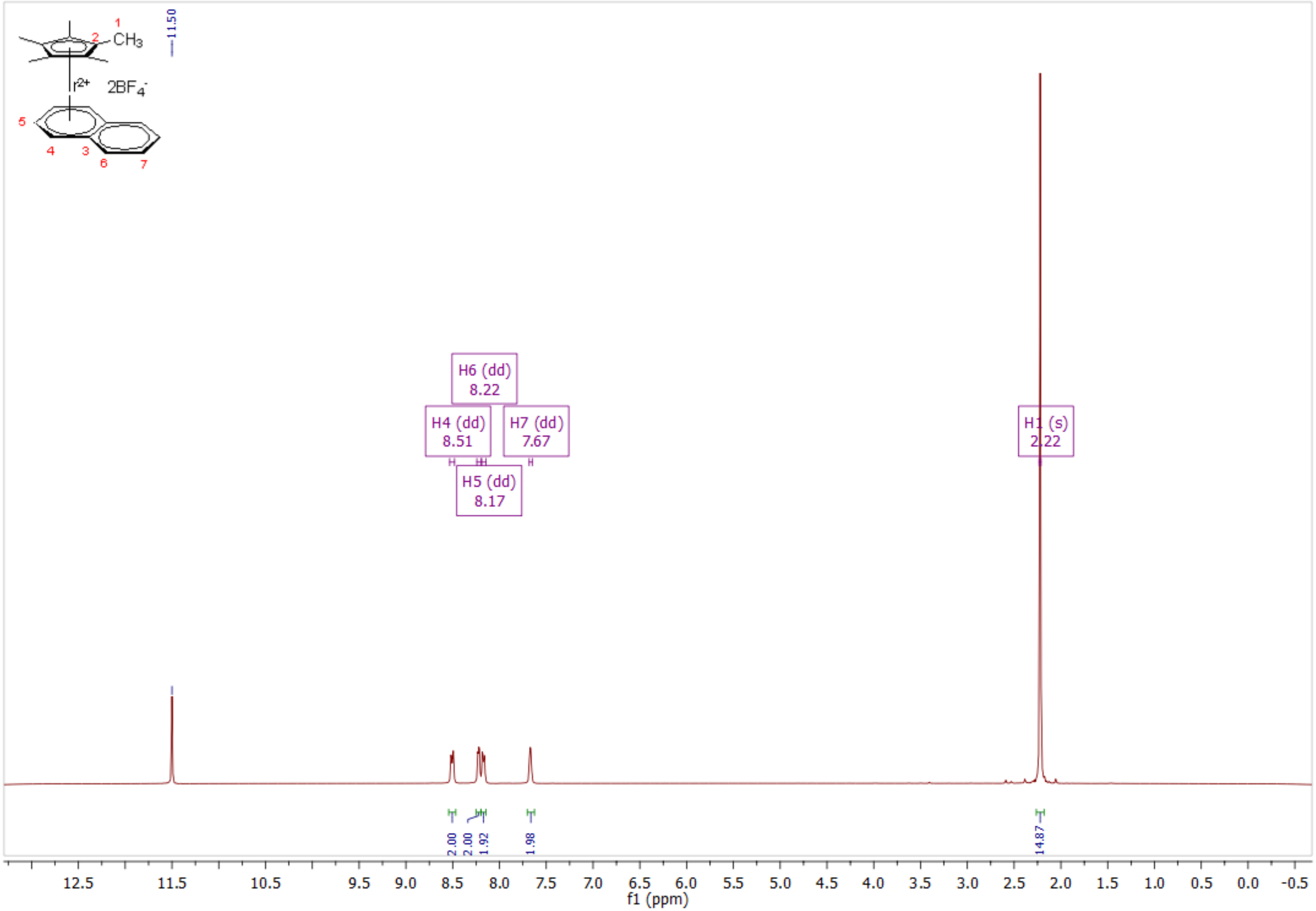

$^{13}C\{[^{1}H\}]$ NMR spectrum of **[Cp*Ir(η^6-napthalene)][BF$_4$]$_2$** in TFA-d.

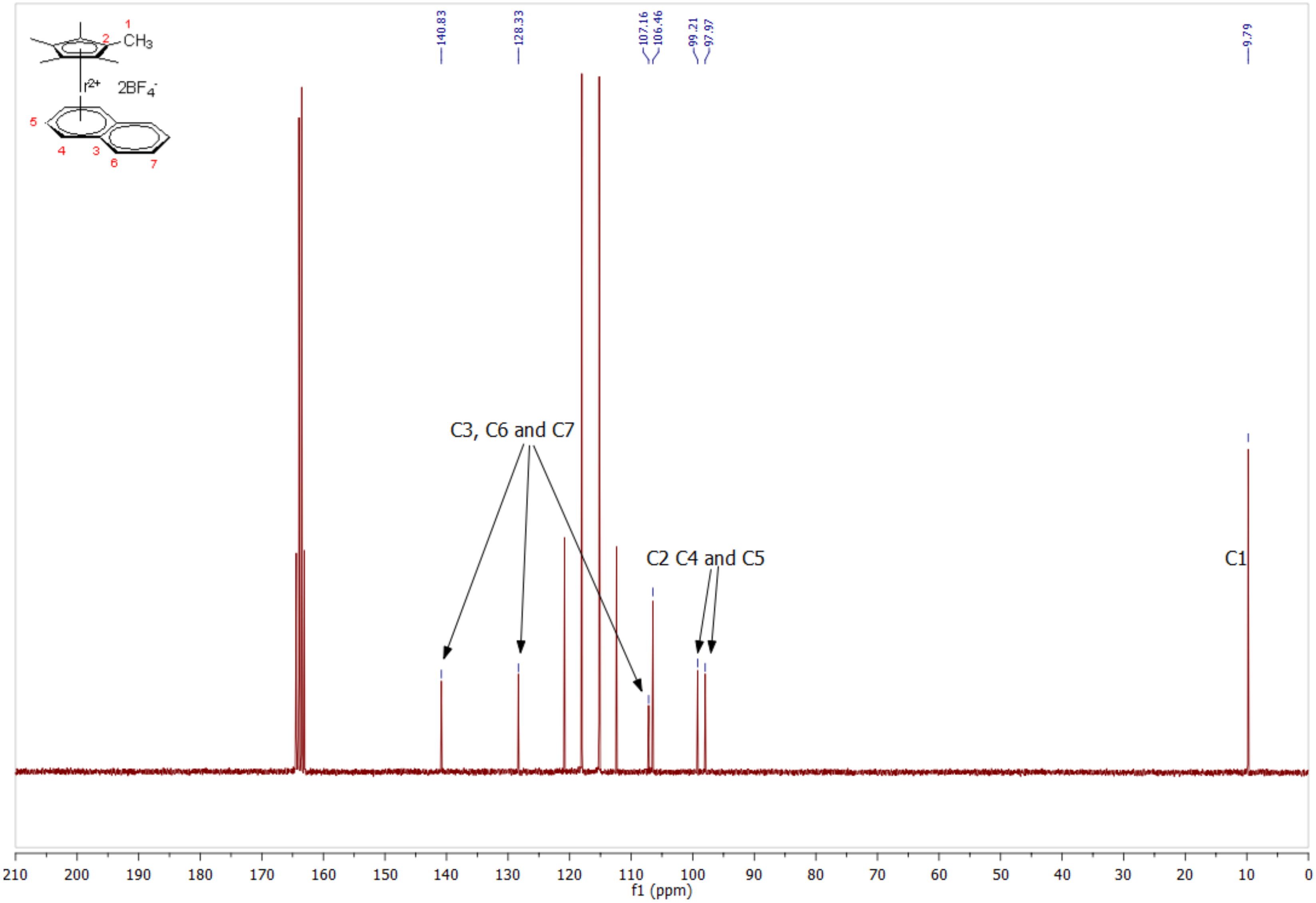

^{1}H NMR spectrum of **[Cp*Ir(η^{6}-2,6-dimethylnaphthalene)][BF$_4$]$_2$** in TFA-d.

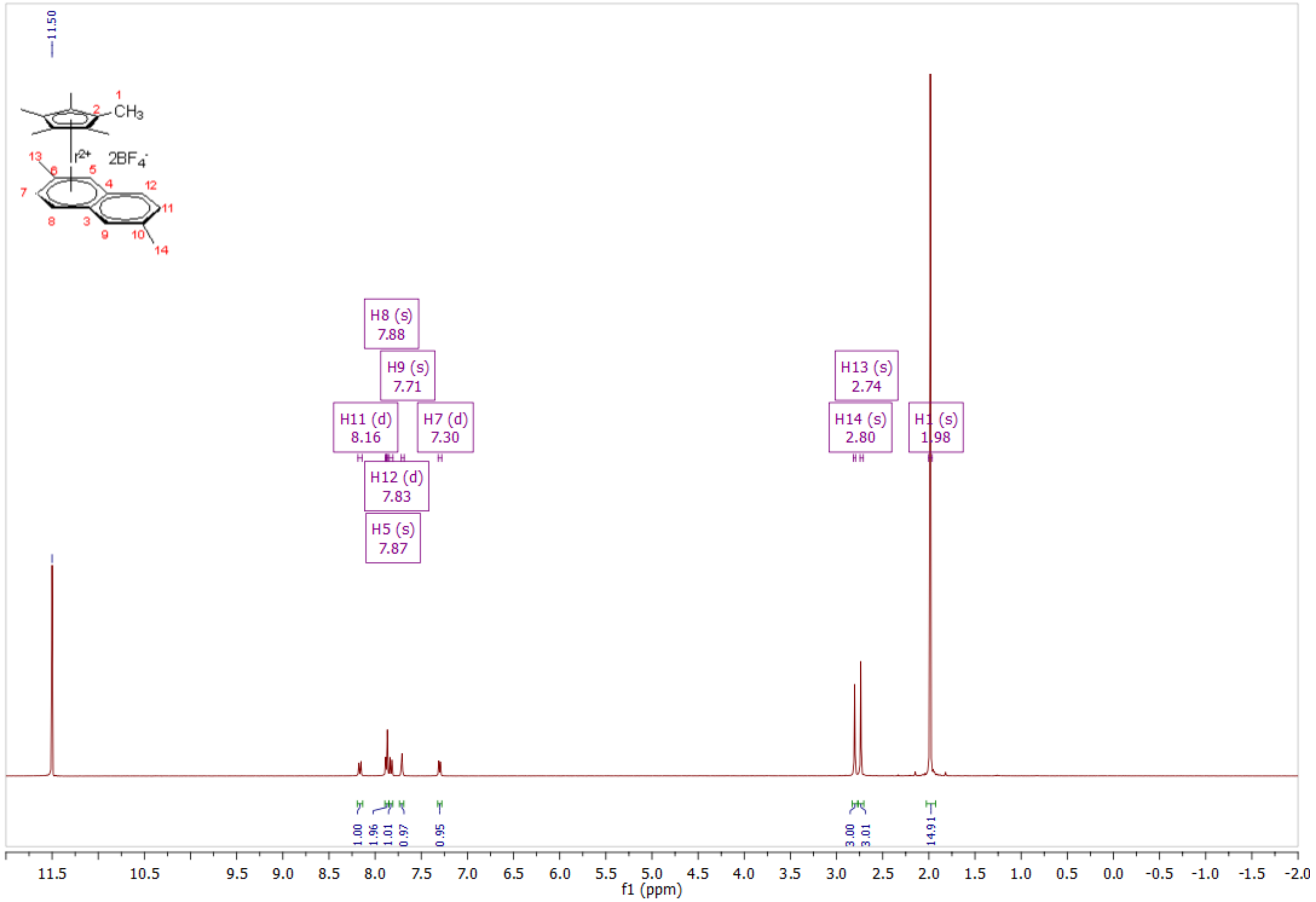

$^{13}C[\{^1H\}]$ NMR spectrum of **[Cp*Ir(η⁶-2,6-dimethylnapthalene)][BF₄]₂** in TFA-d.

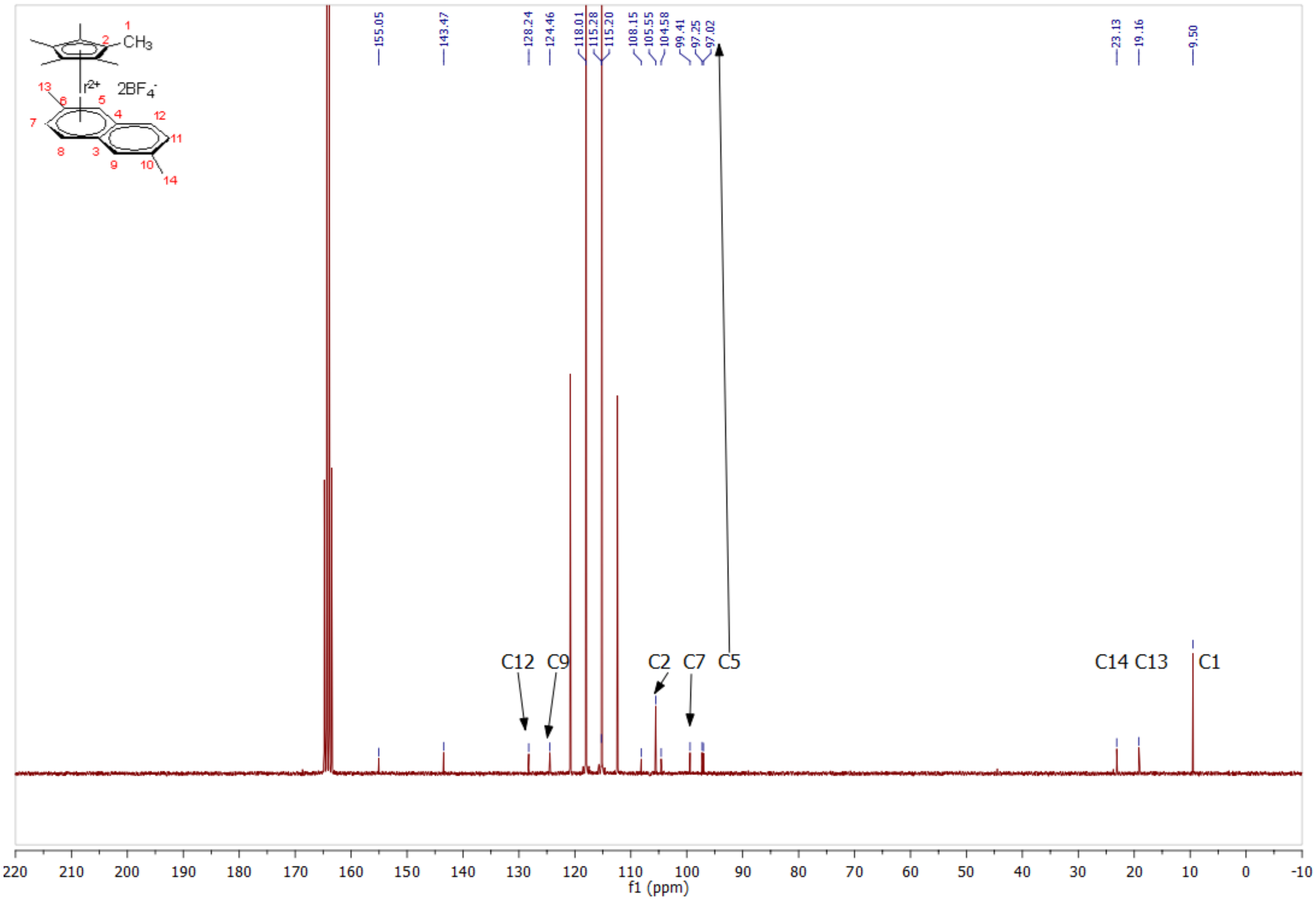

HMBC NMR spectrum of **[Cp*Ir(η[6]-2,6-dimethylnapthalene)][BF$_4$]$_2$** in TFA-d.

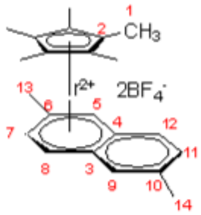

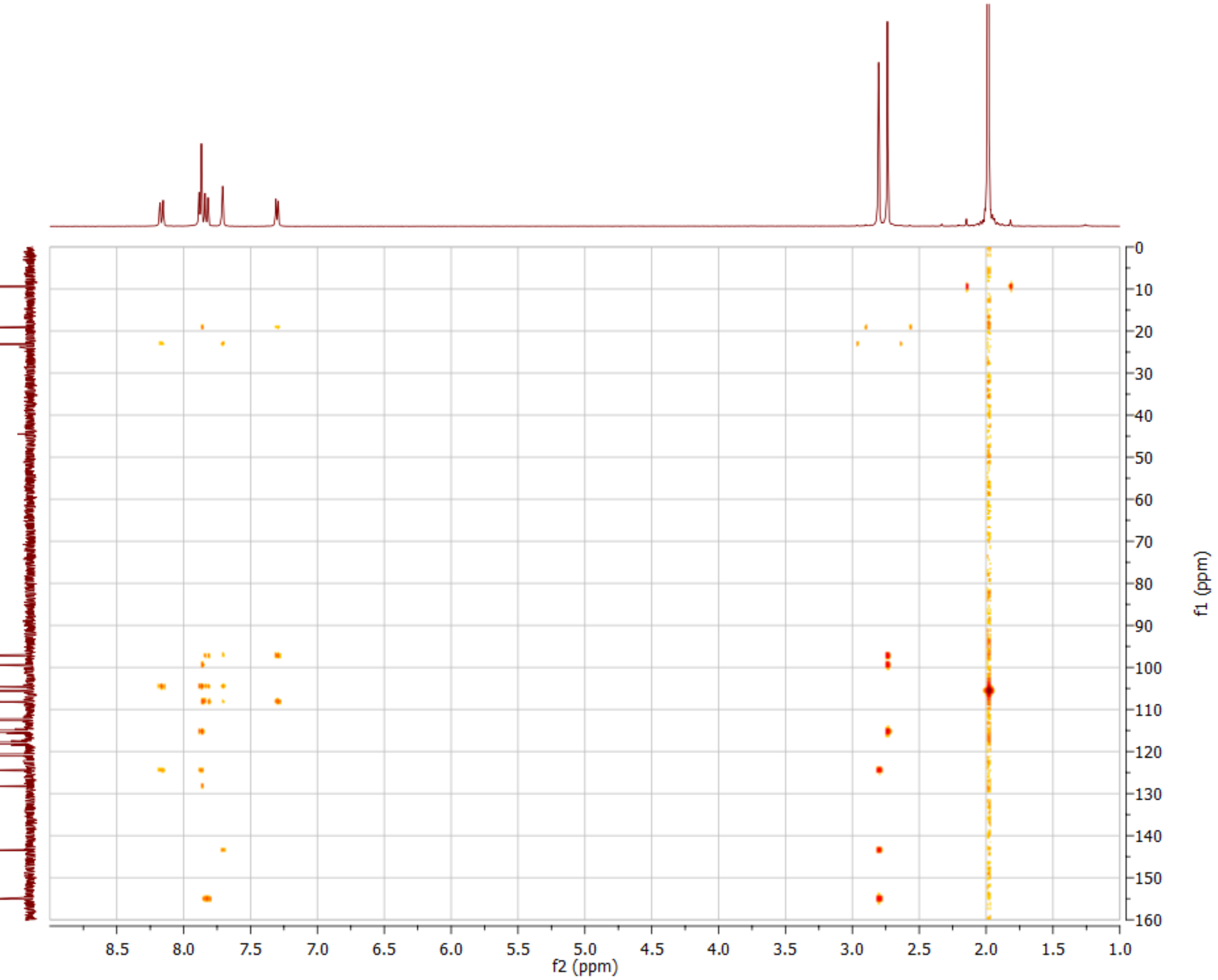

^{2}H NMR spectrum of **mesitylene-d$_3$** in TFA.

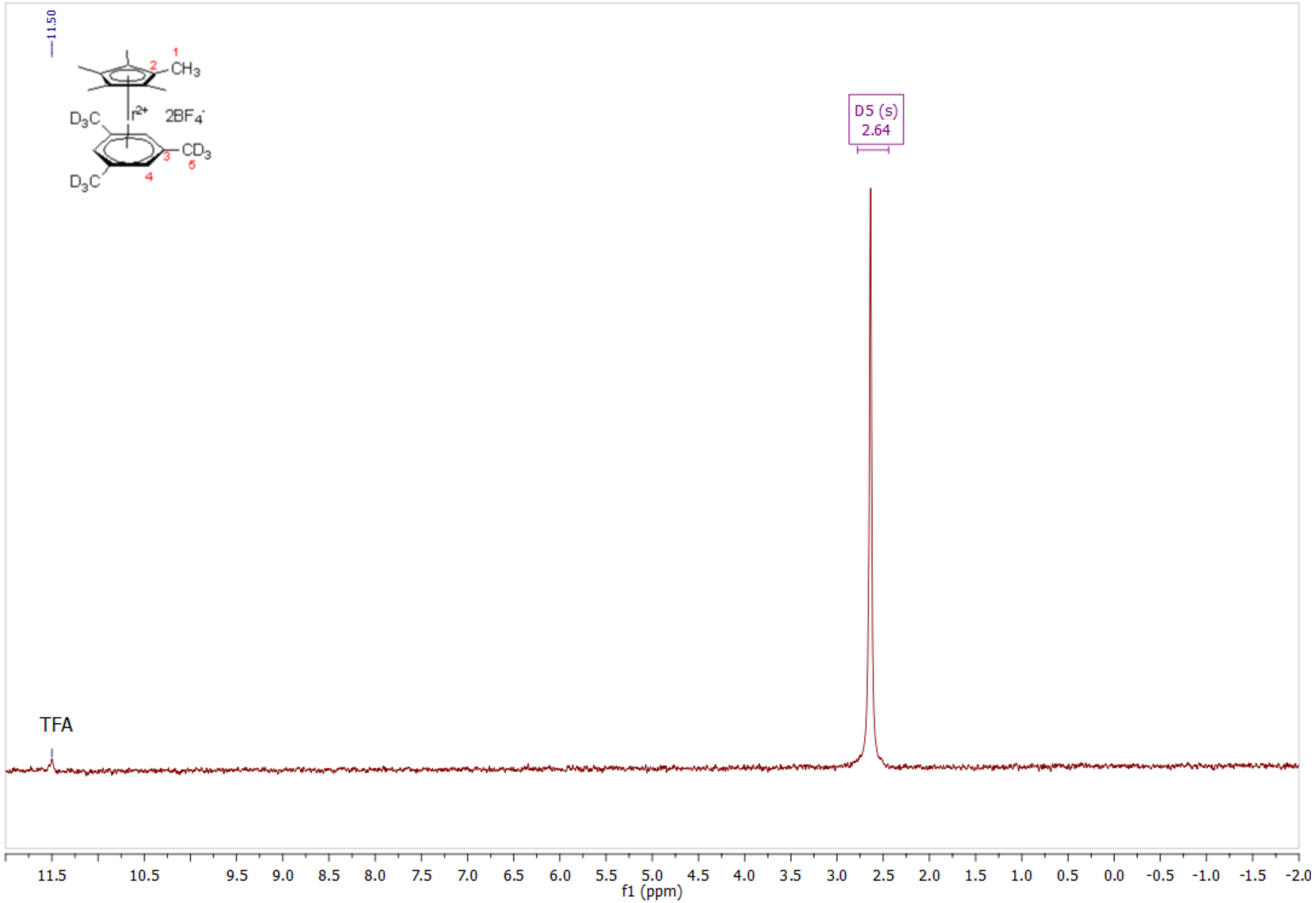

^{1}H NMR spectrum of **1-d₃** in C_6D_6.

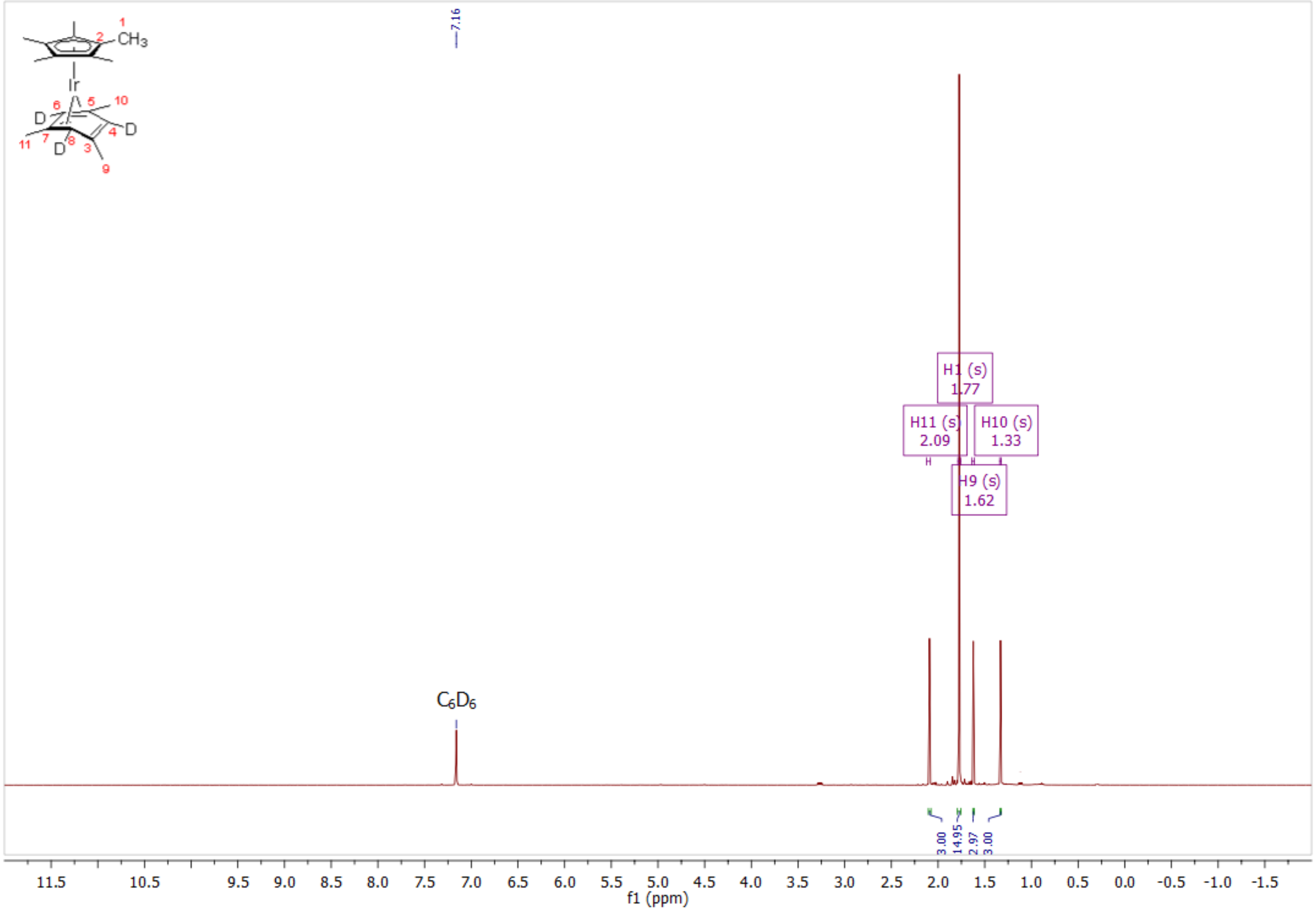

^{2}H NMR spectrum of **1-d₃** in C_6H_6.

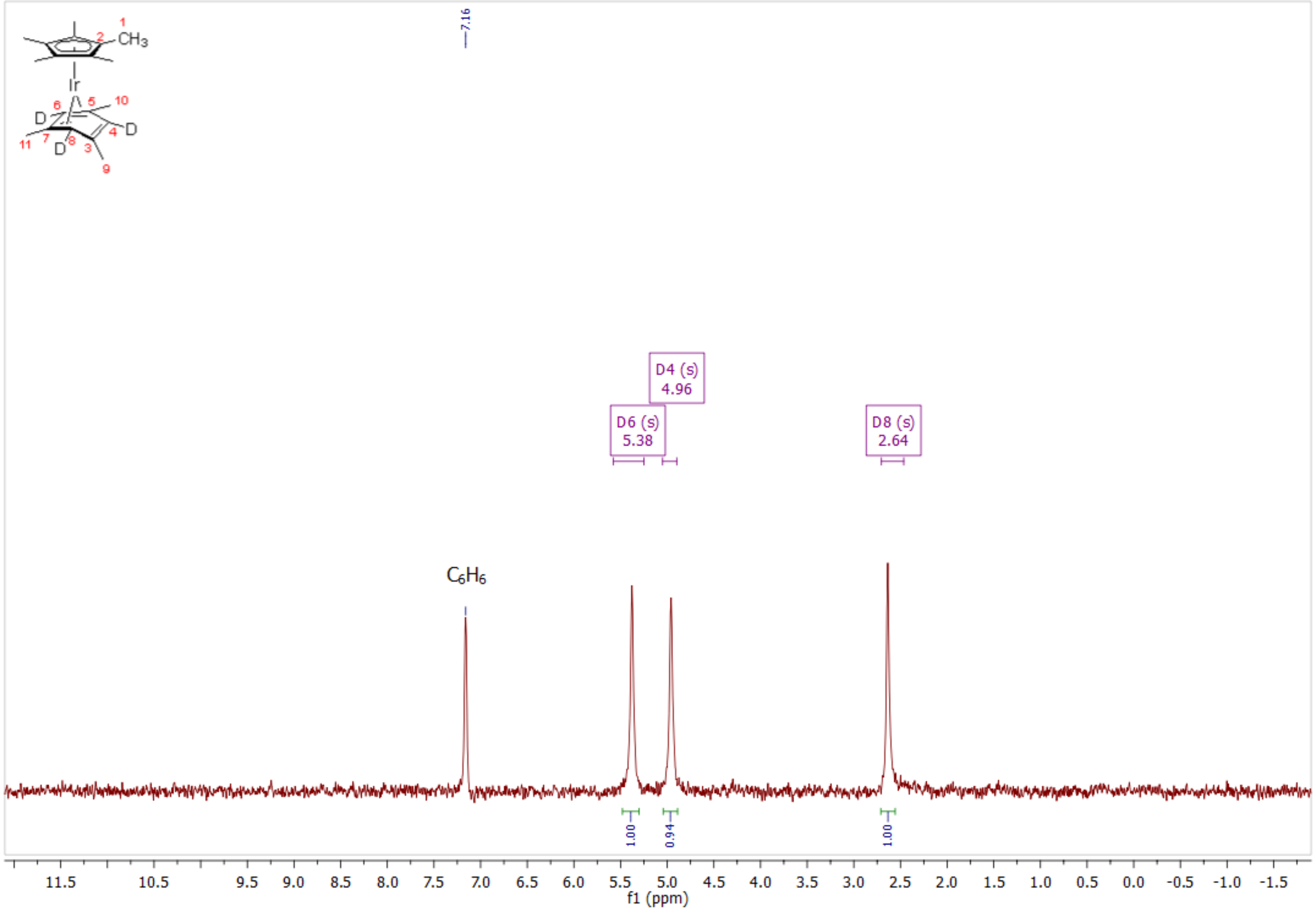

^1H NMR spectrum of **1-d₉** in C_6D_6.

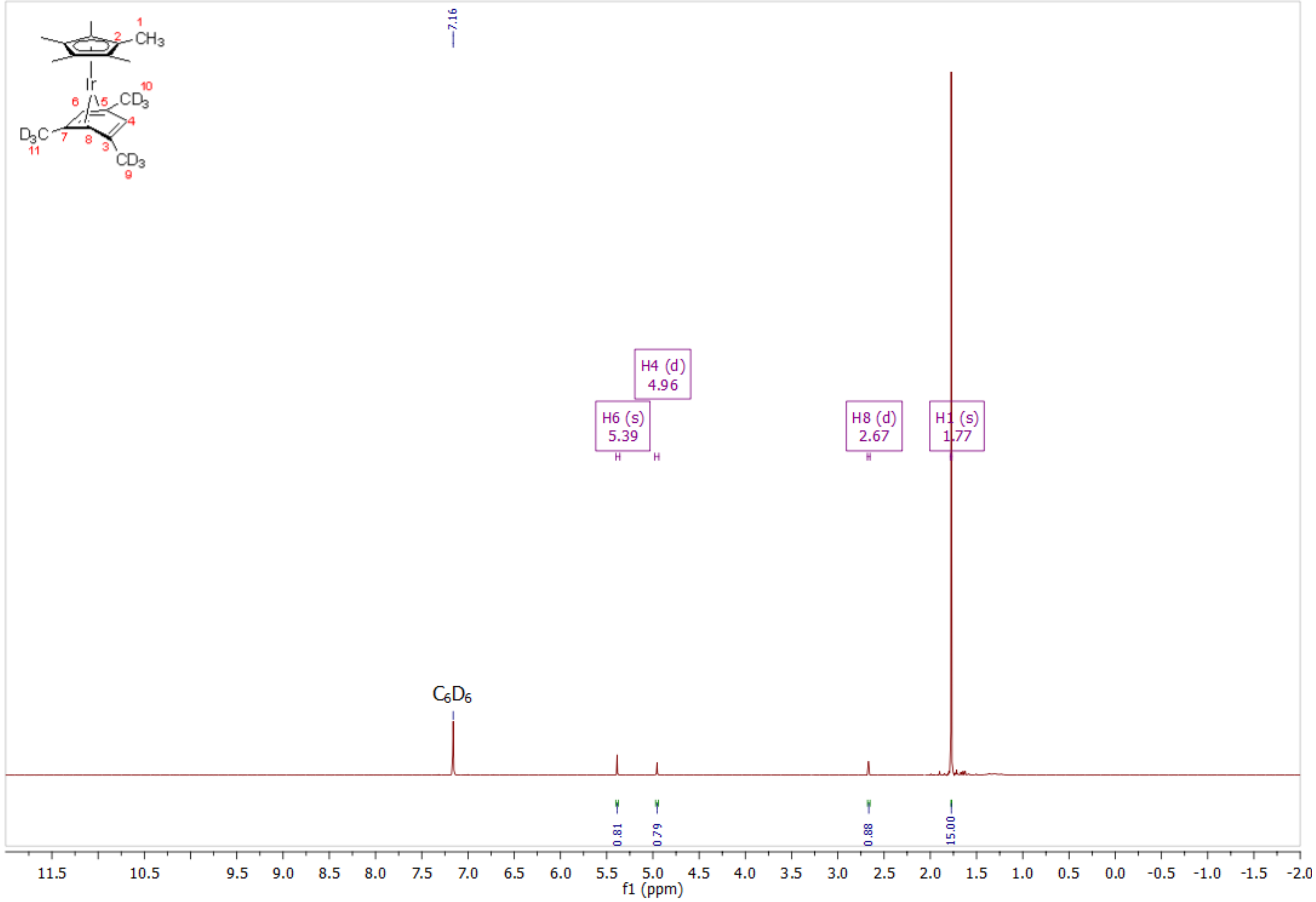

^{2}H NMR spectrum of **1-d₉** in C_6H_6.

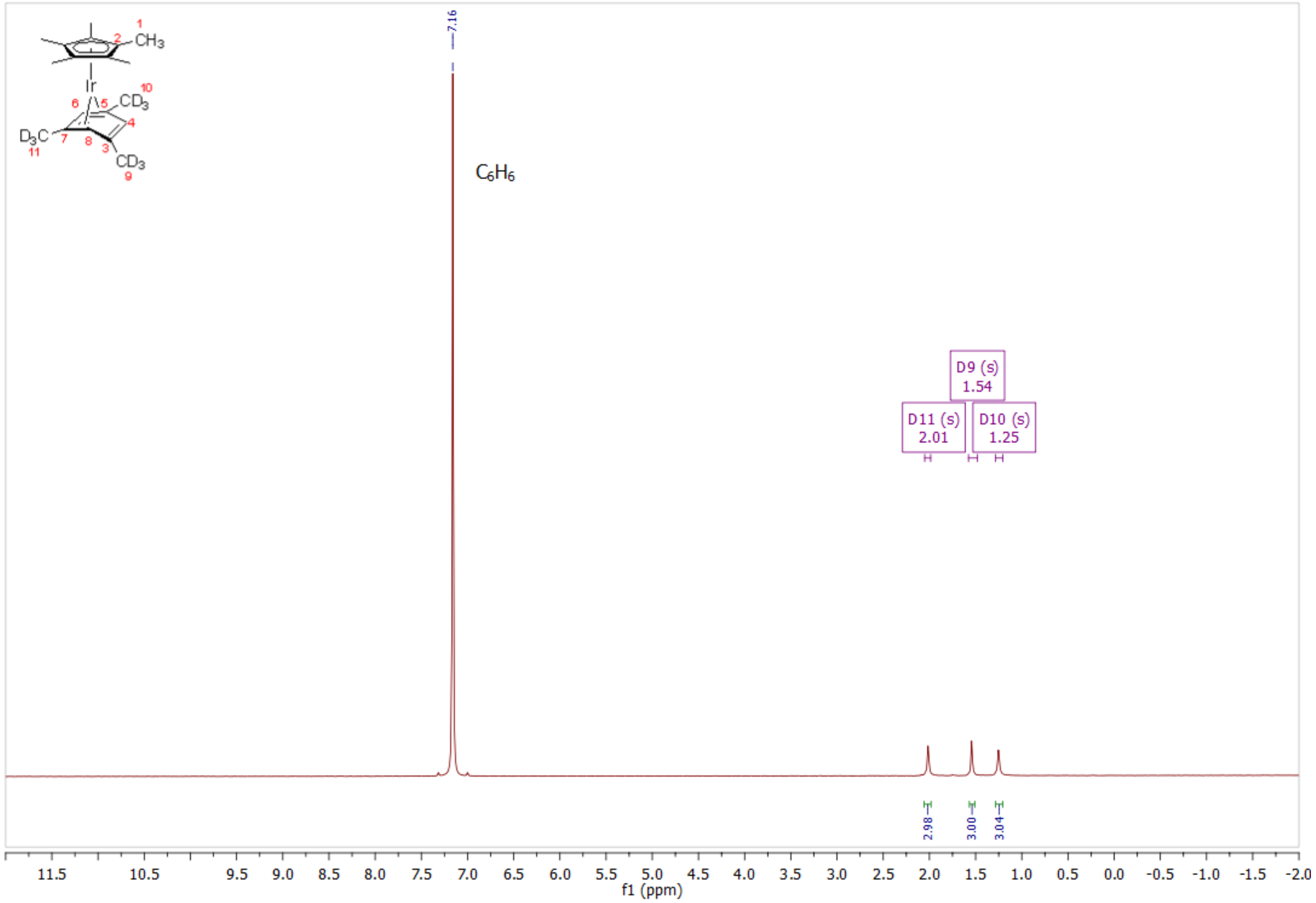

^{1}H NMR spectrum of **[Cp*Ir(η[4]-C_6Me_6)]** in C_6D_6.

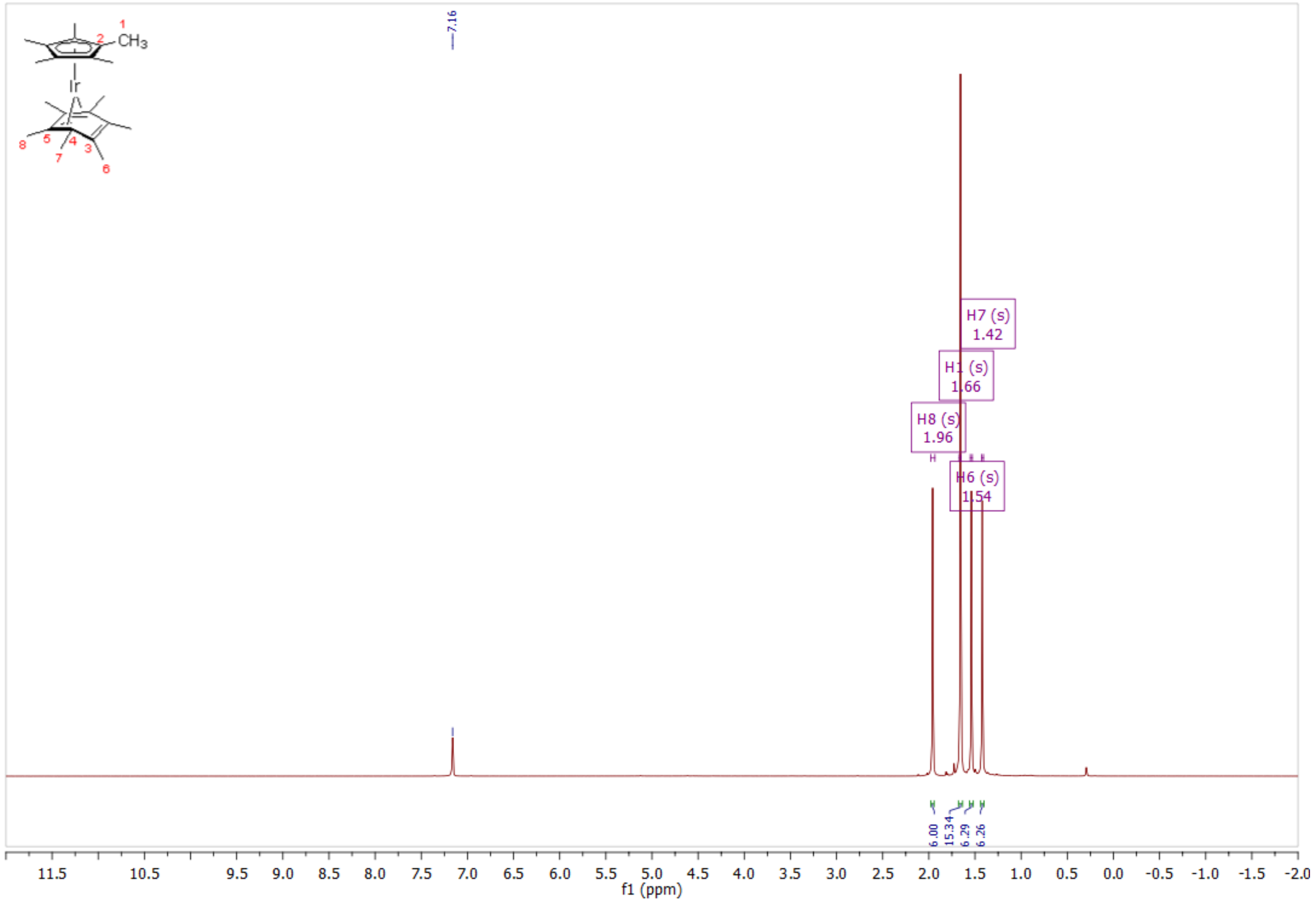

$^{13}C[\{^1H\}]$ NMR spectrum of **[Cp*Ir(η^4-C_6Me_6)]** in C_6D_6.

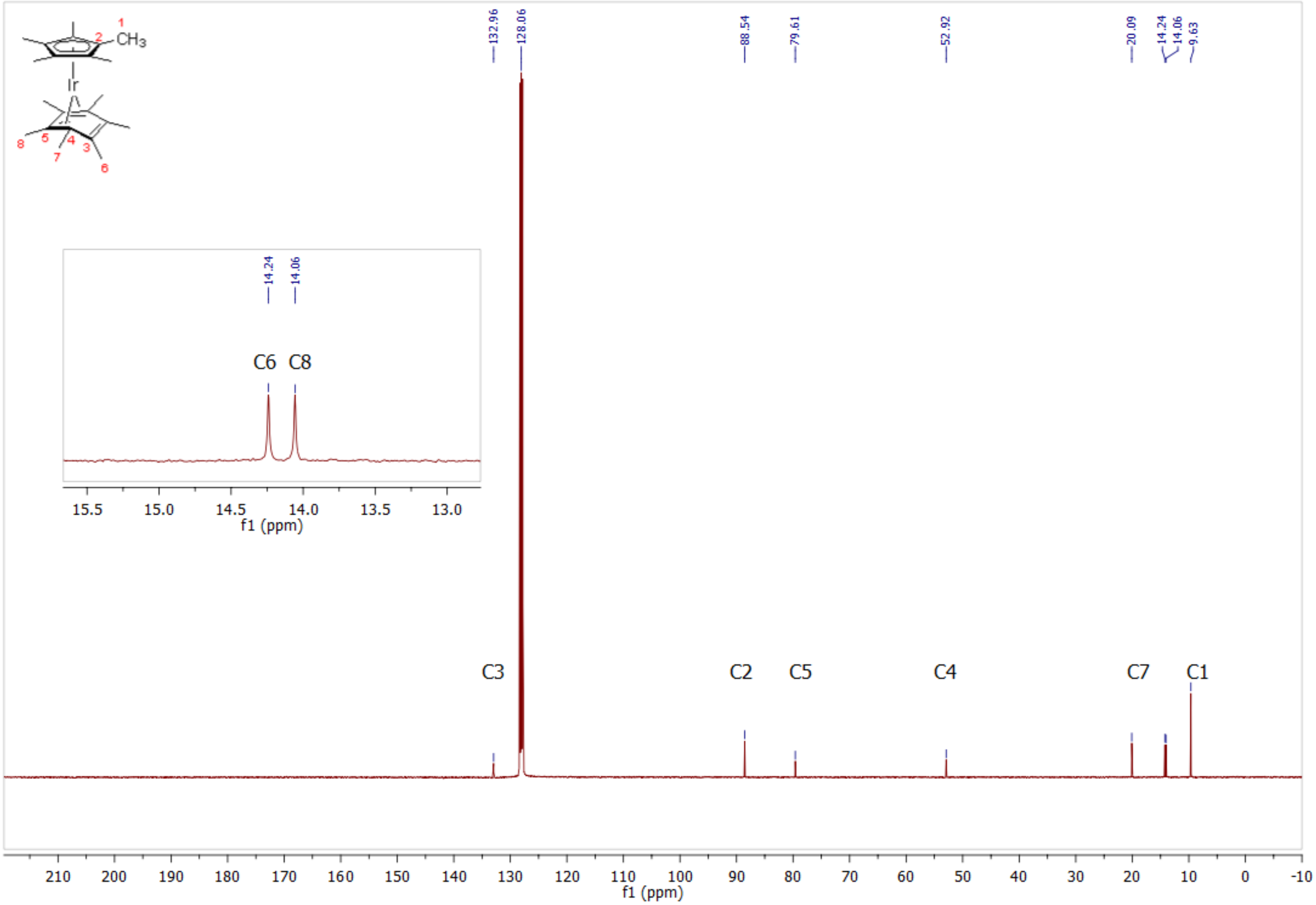

HMBC NMR spectrum of **[Cp*Ir(η^4-C_6Me_6)]** in C_6D_6.

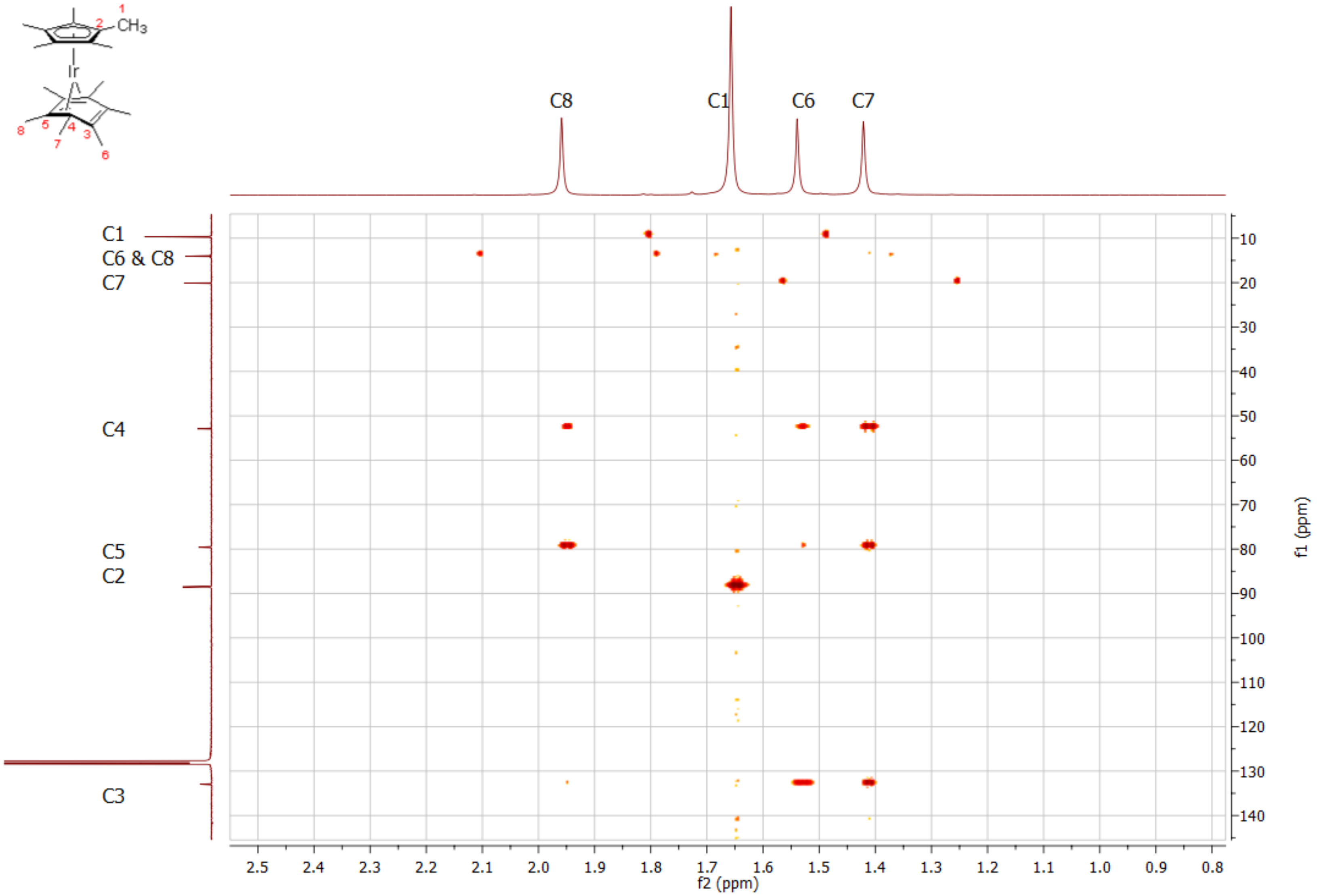

^{1}H NMR spectrum of **[Cp*Ir(η[4]-naphthalene)]** in C_6D_6.

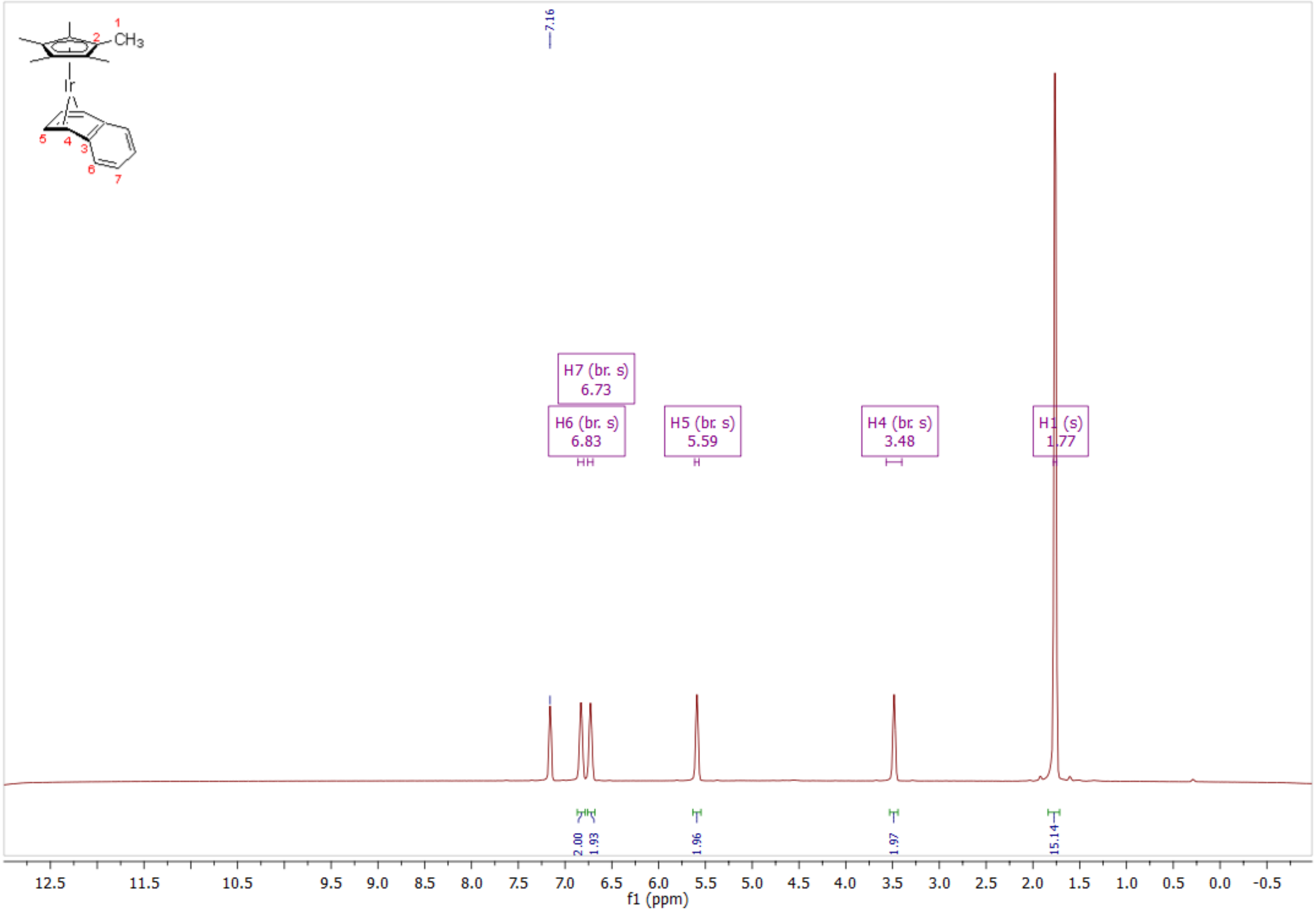

$^{13}C\{^{1}H\}$ NMR spectrum of **[Cp*Ir(η^4-naphthalene)]** in C_6D_6.

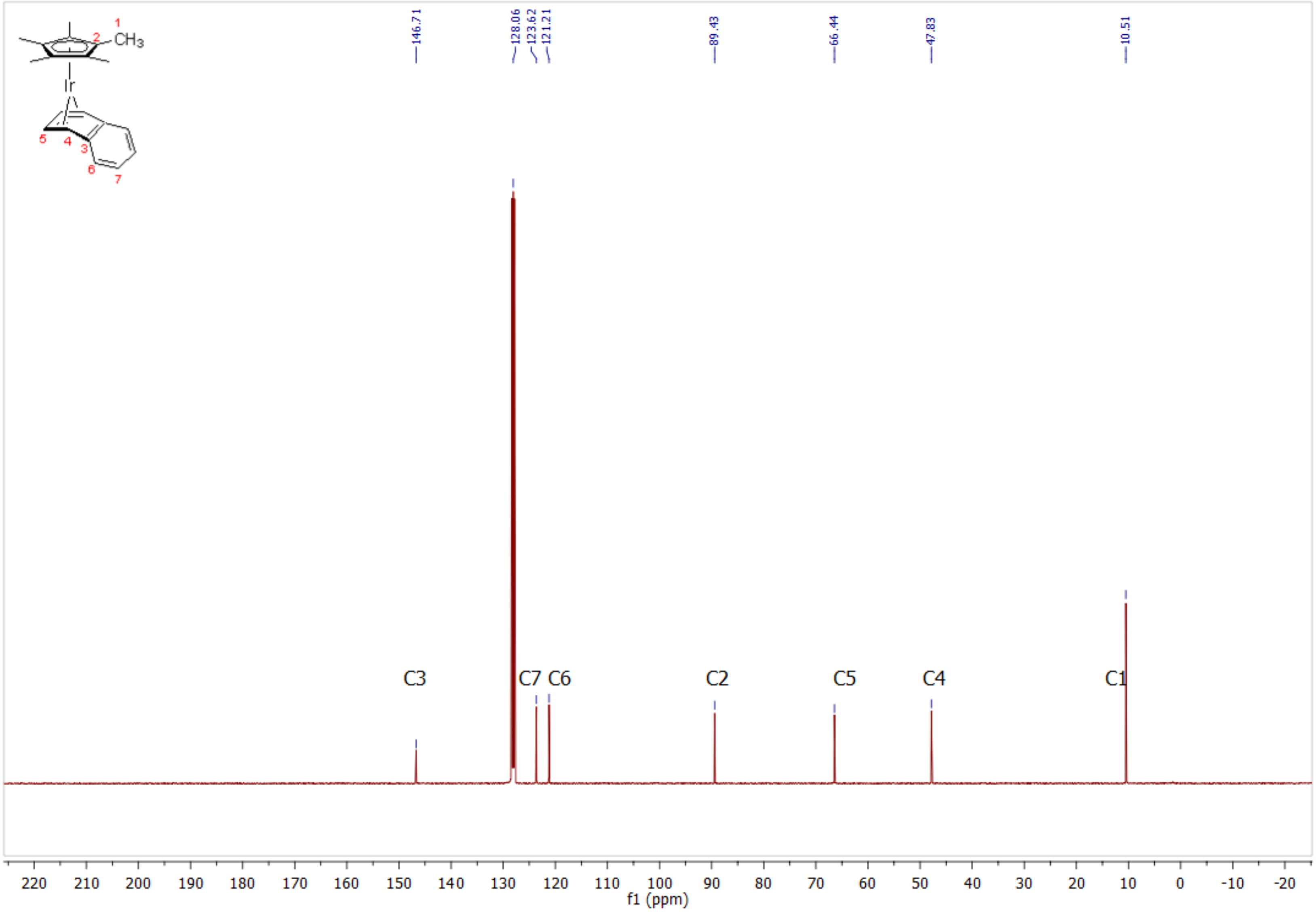

HSQC NMR spectrum of **[Cp*Ir(η⁴-naphthalene)]** in C_6D_6.

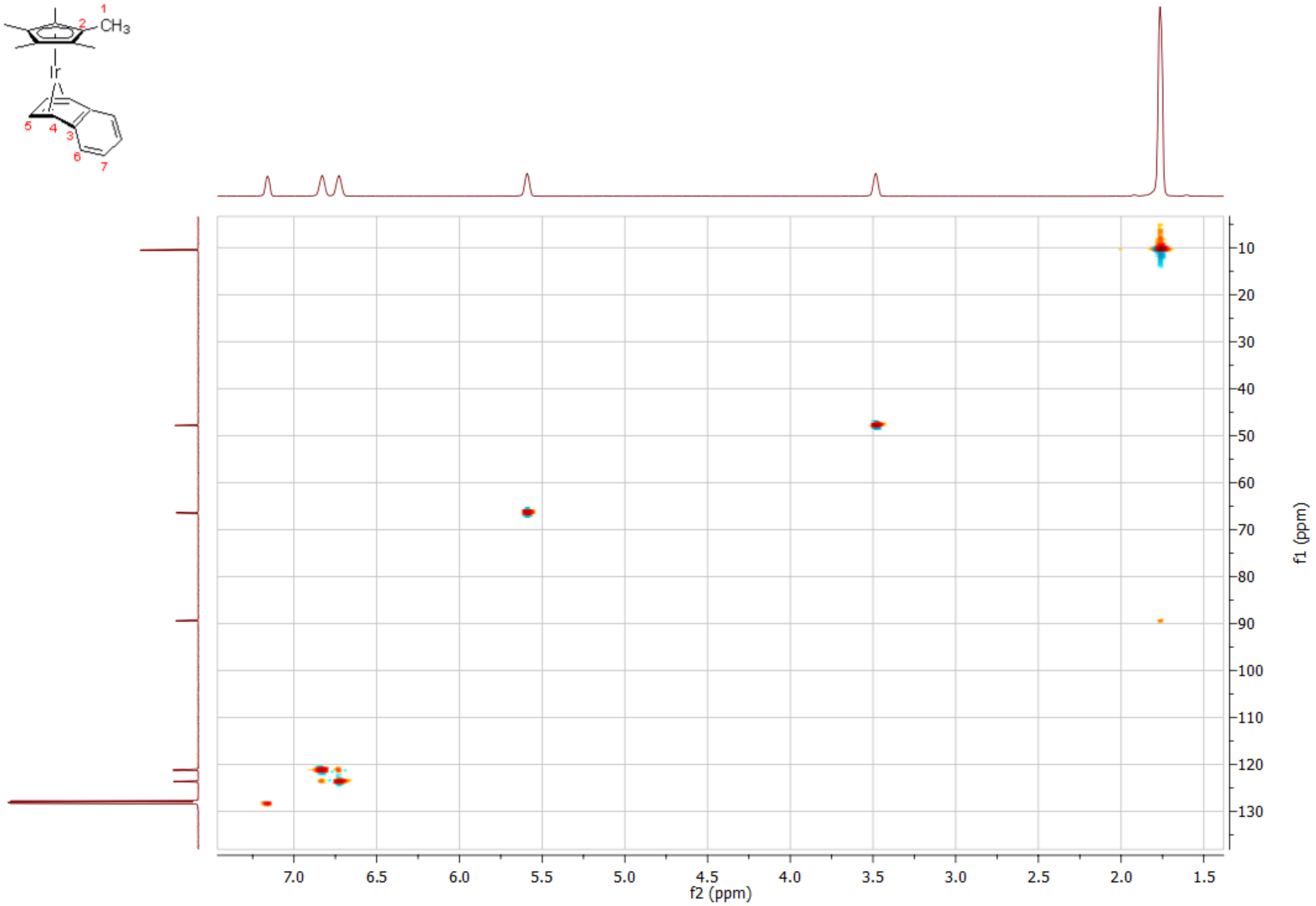

HMBC NMR spectrum of **[Cp*Ir(η^4-naphthalene)]** in C_6D_6.

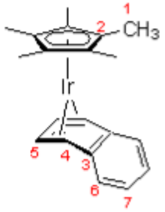

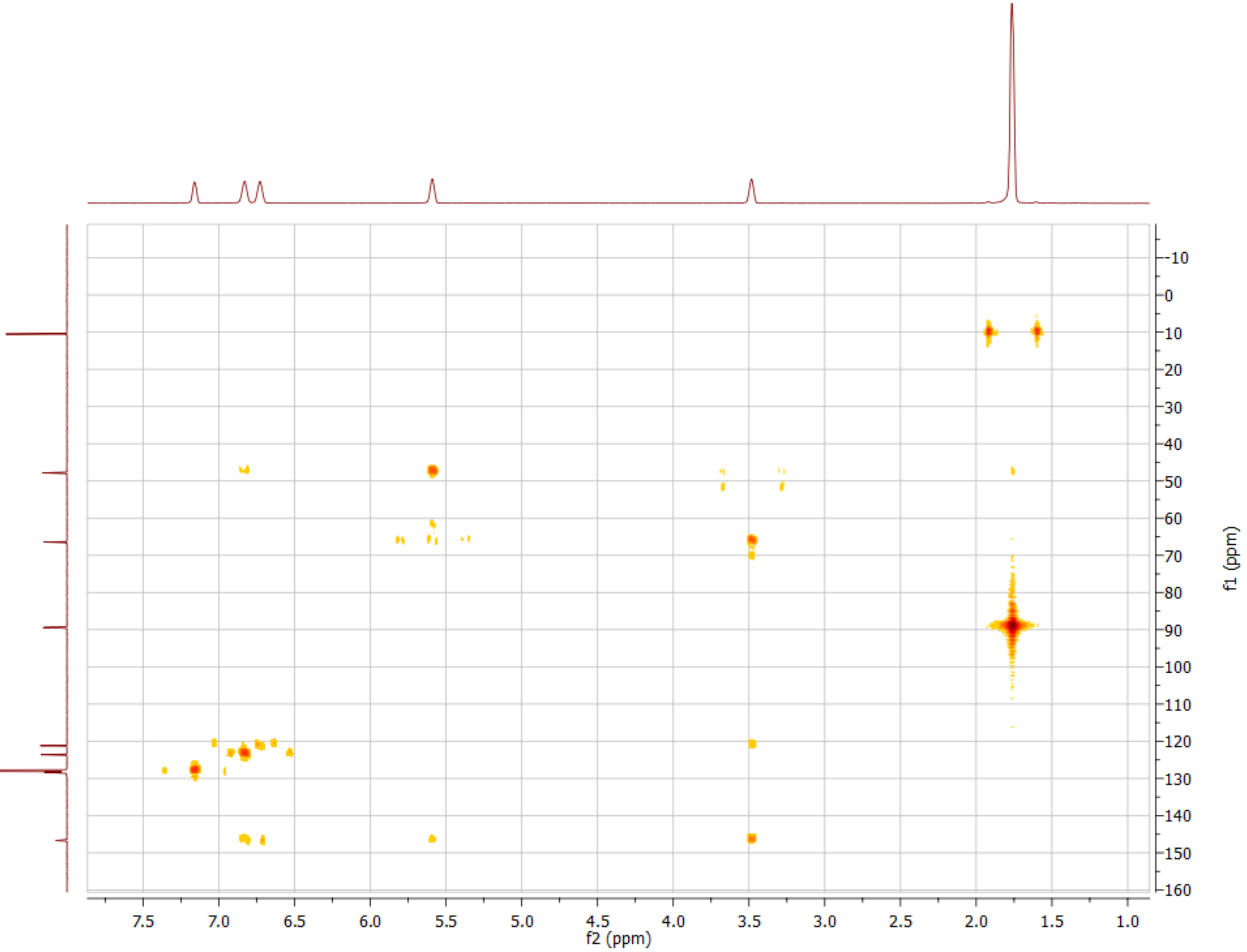

^{1}H NMR spectrum of **[Cp*Ir(η[4]-2,6-dimethylnaphthalene)]** in C_6D_6.

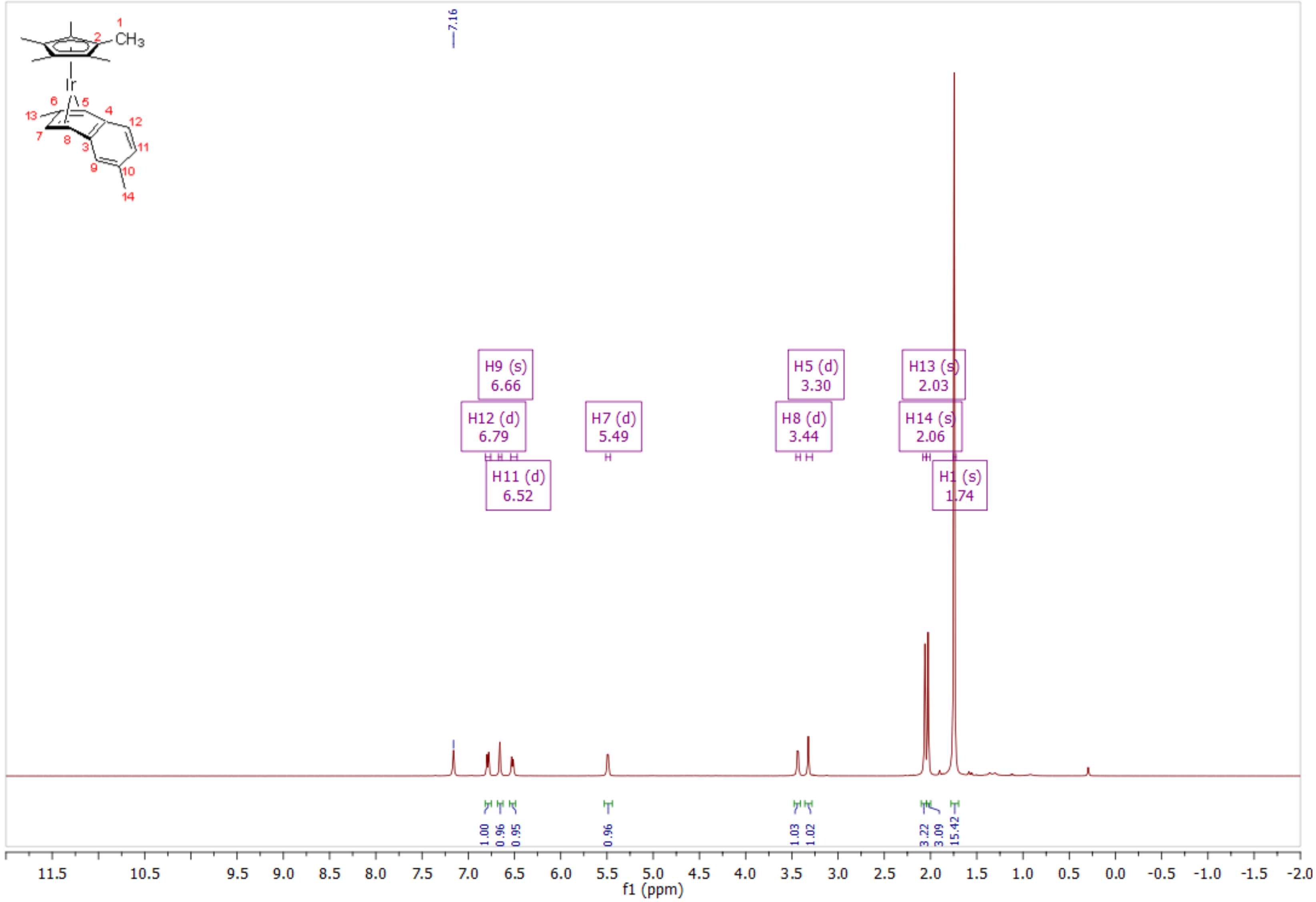

$^{13}C\{^{1}H\}$ NMR spectrum of **[Cp*Ir(η⁴-2,6-dimethylnaphthalene)]** in C_6D_6.

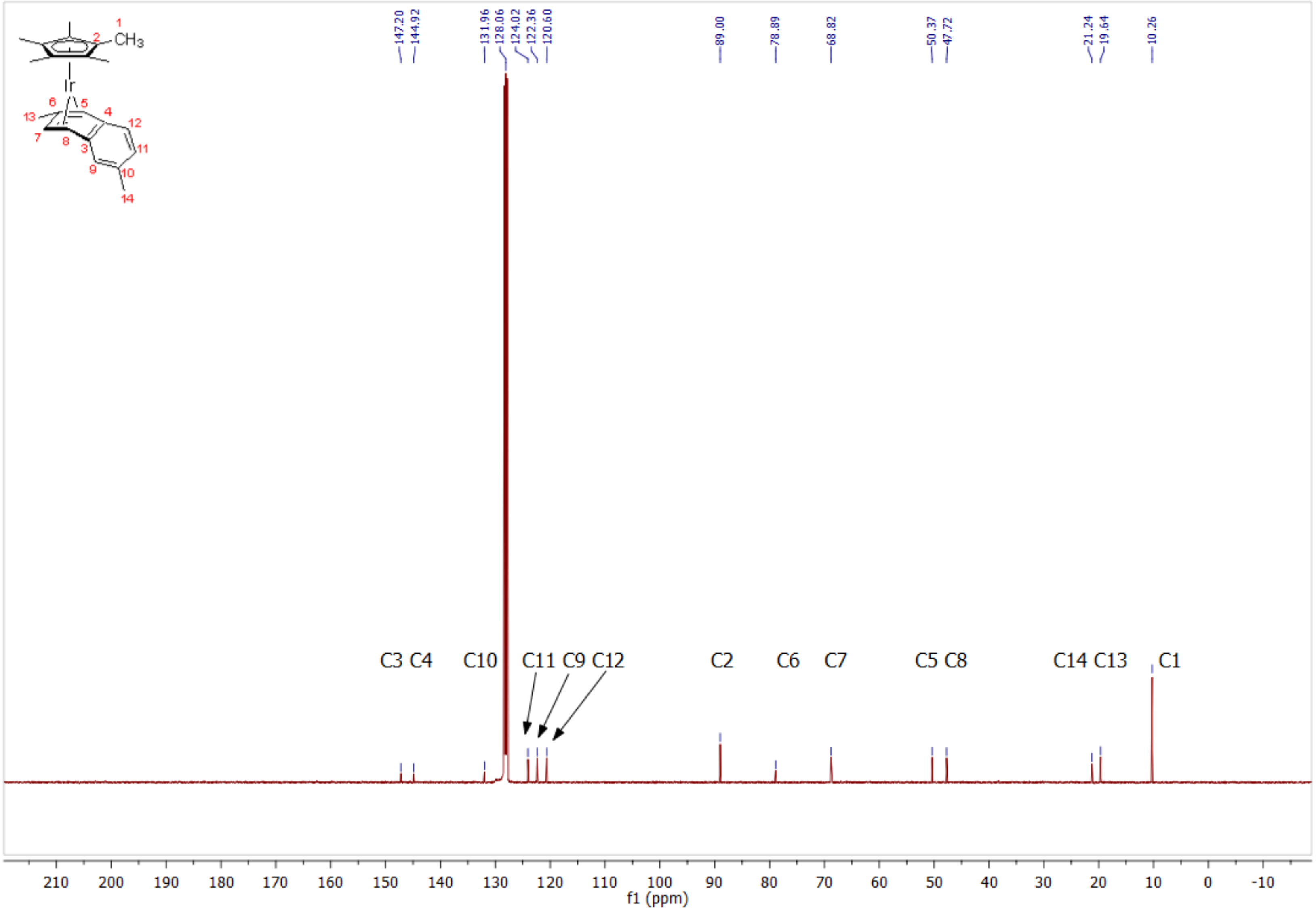

HSQC NMR spectrum of **[Cp*Ir(η[4]-2,6-dimethylnaphthalene)]** in C_6D_6.

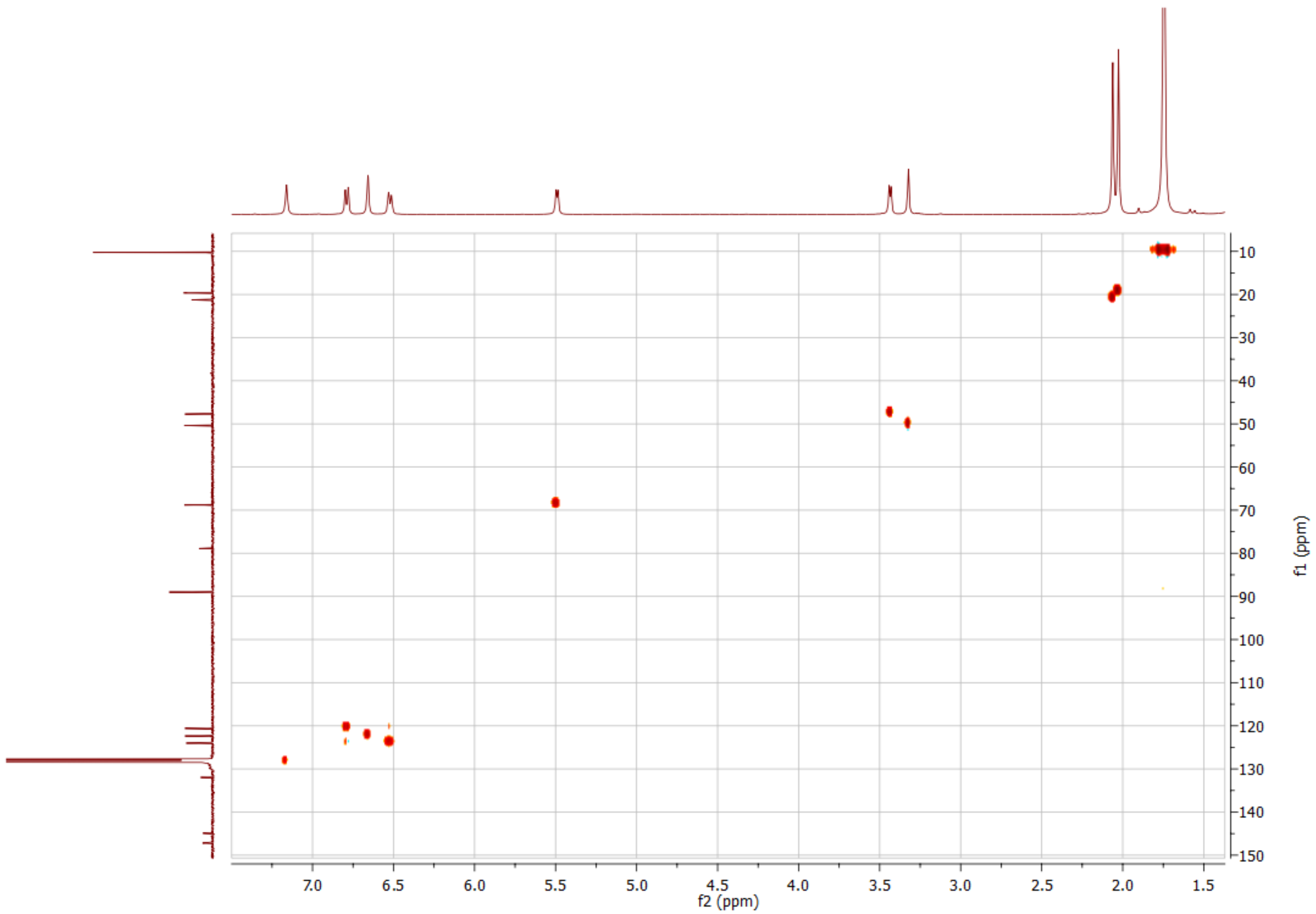

HMBC NMR spectrum of **[Cp*Ir(η⁴-2,6-dimethylnaphthalene)]** in C_6D_6.

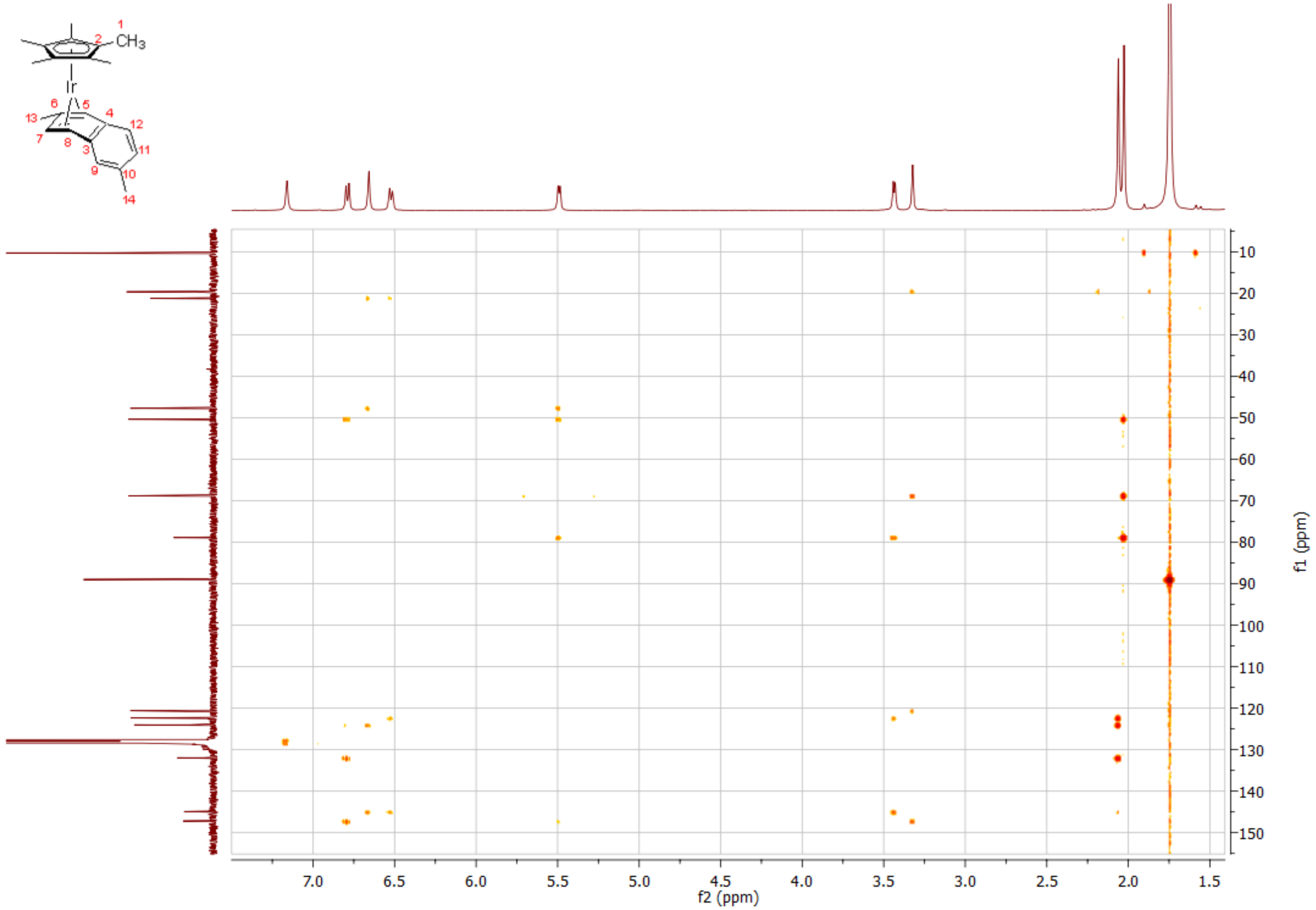

^1H NMR spectrum of **2-d₃** in C_6D_6.

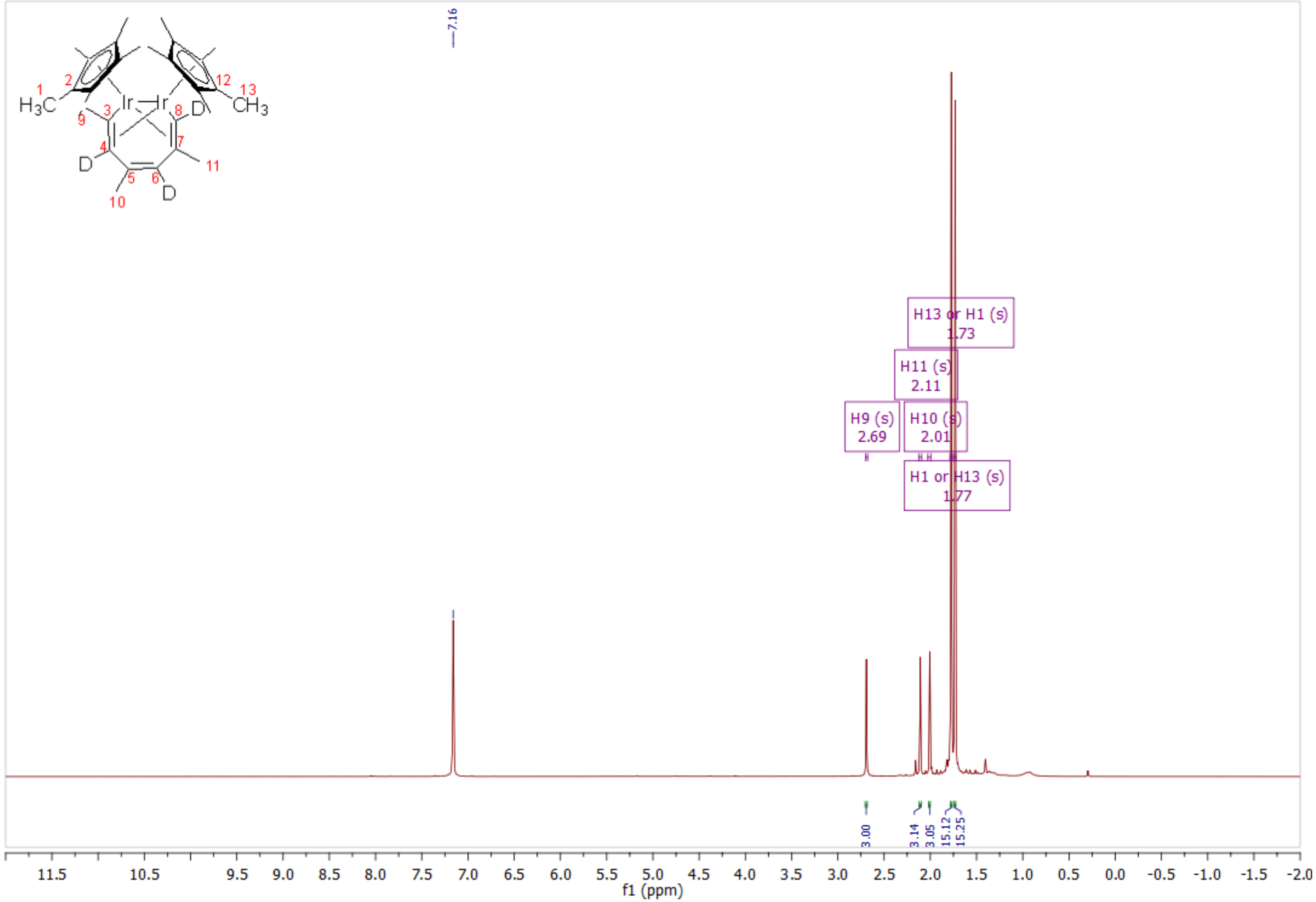

^{2}H NMR spectrum of **2-d₃** in C_6H_6.

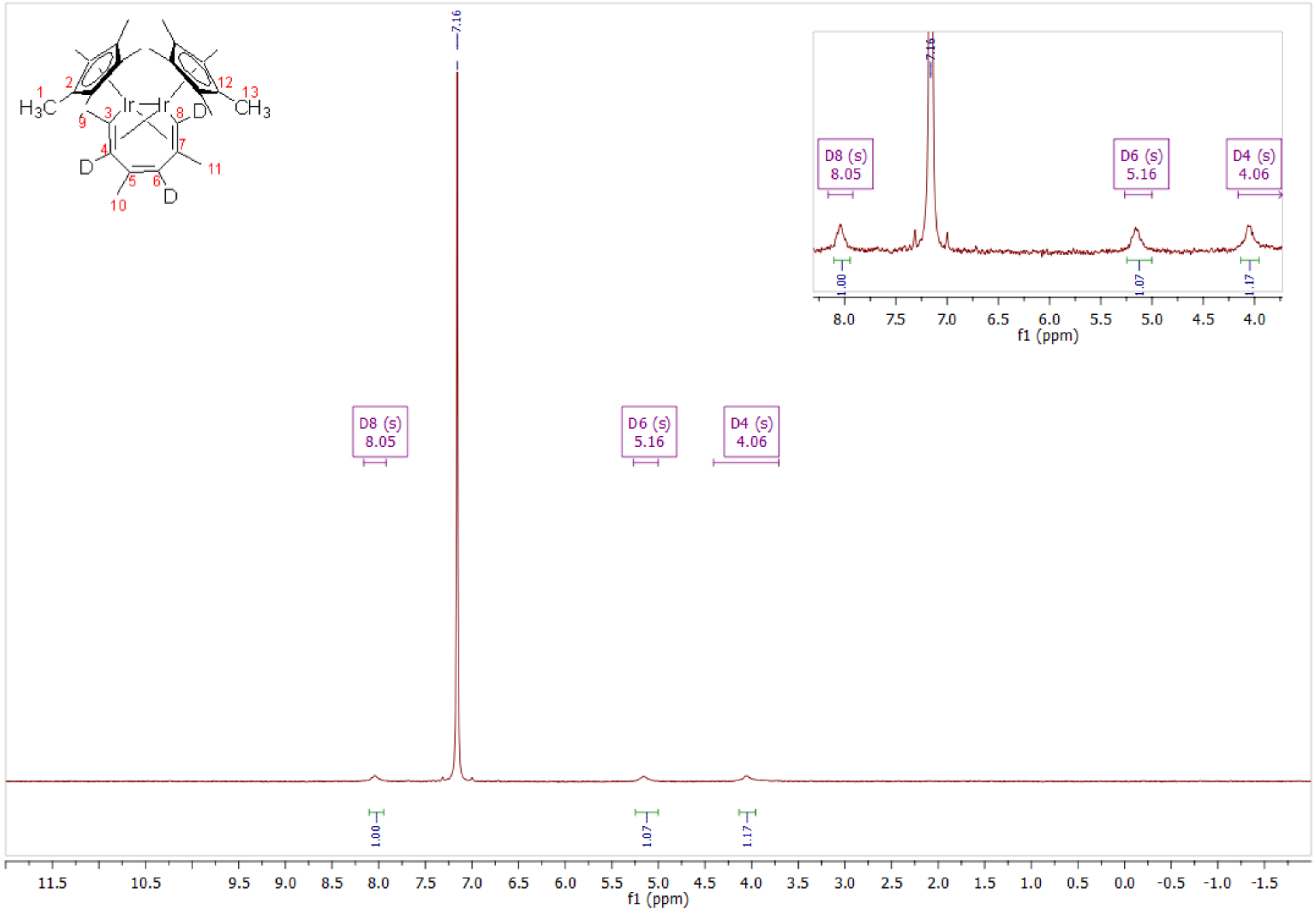

^{1}H NMR spectrum of **2-d₉** in C_6D_6.

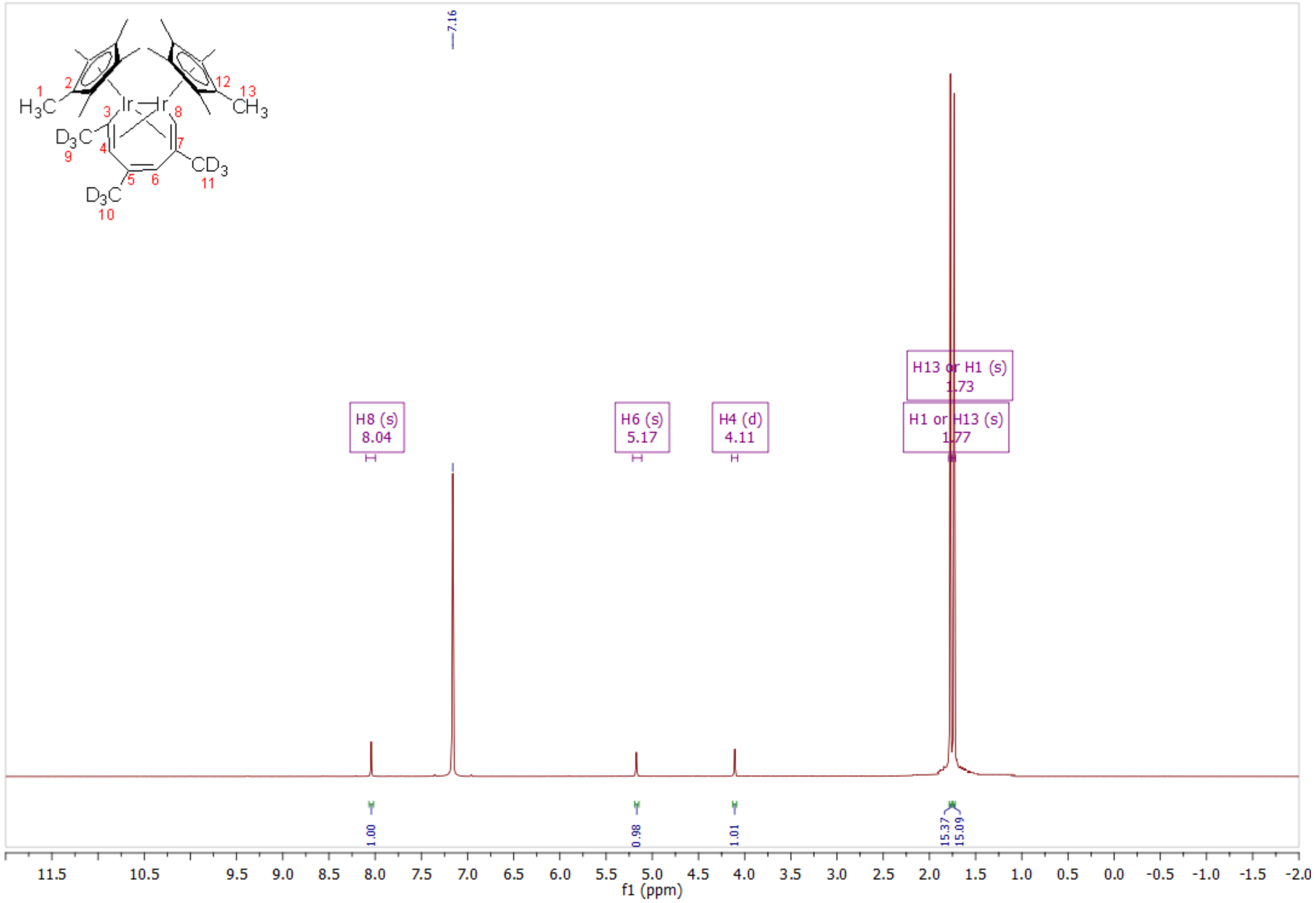

^{2}H NMR spectrum of **2-d$_9$** in C_6H_6.

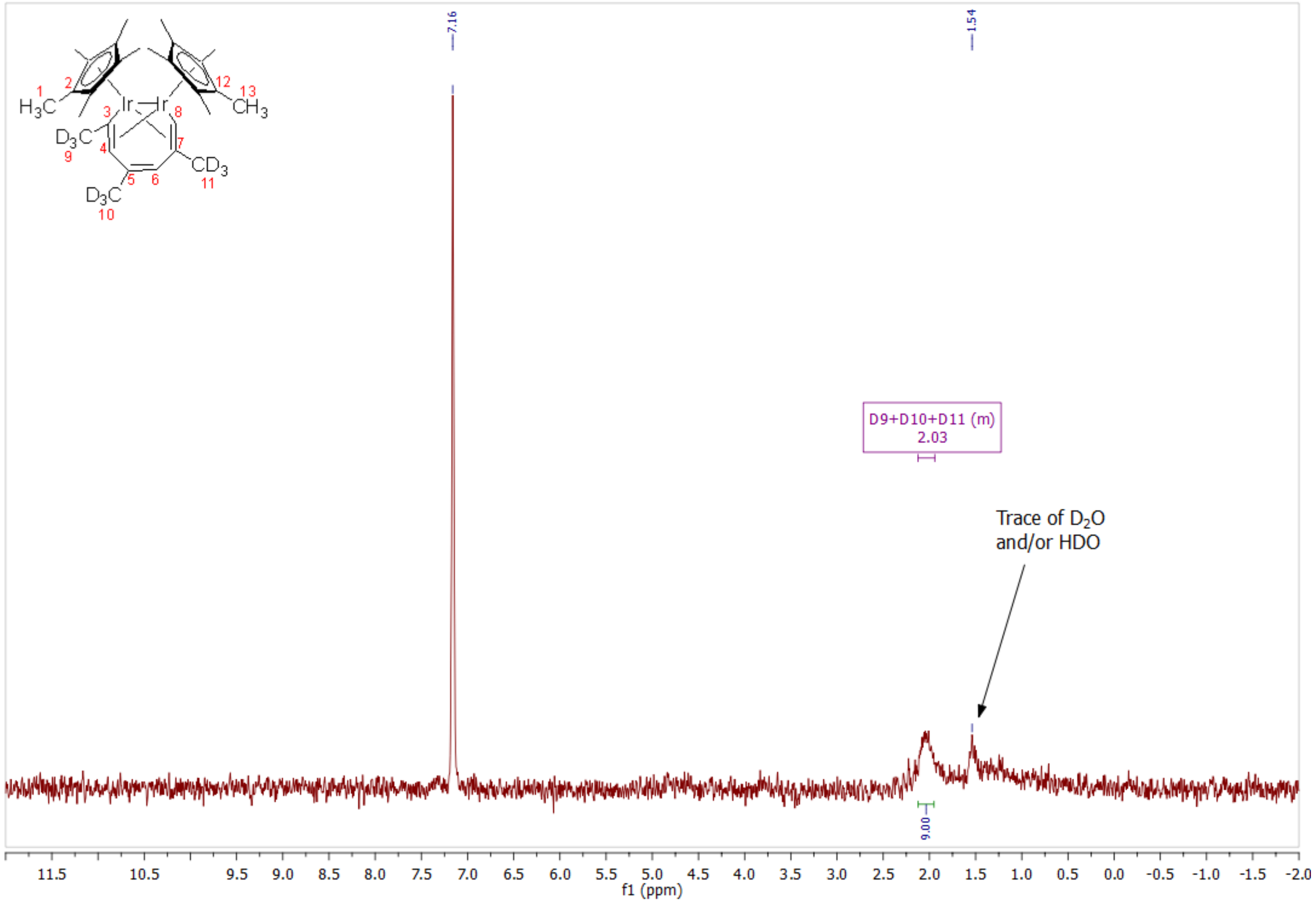

^{1}H NMR spectrum of the reaction mixture of thermal cleavage of **1** in C_6D_6 at full conversion of **1**.

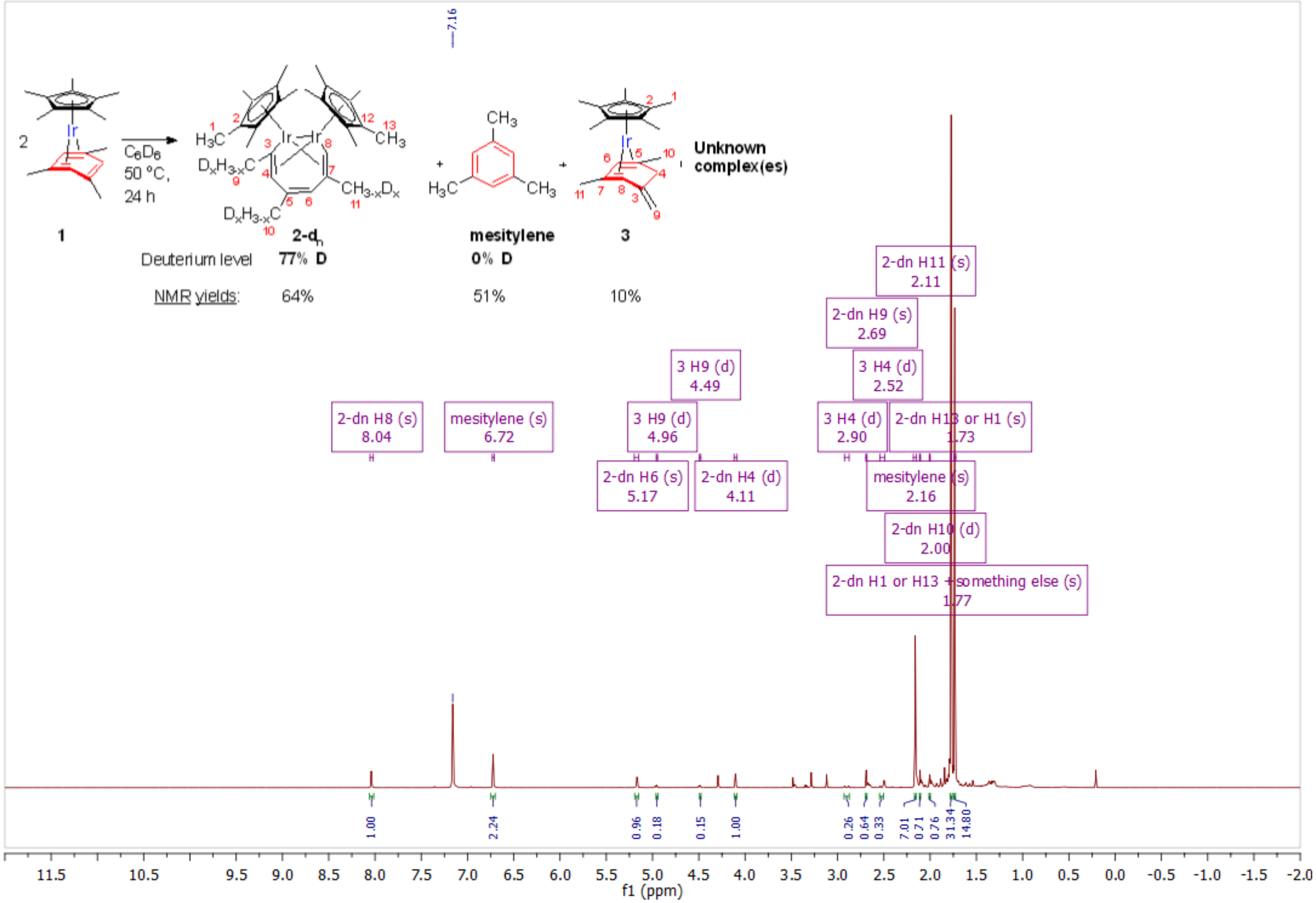

A low field region (8 to 4 ppm) of the ^{1}H NMR spectrum of the reaction mixture of thermal cleavage of **1** in C_6D_6 at full conversion of **1**.

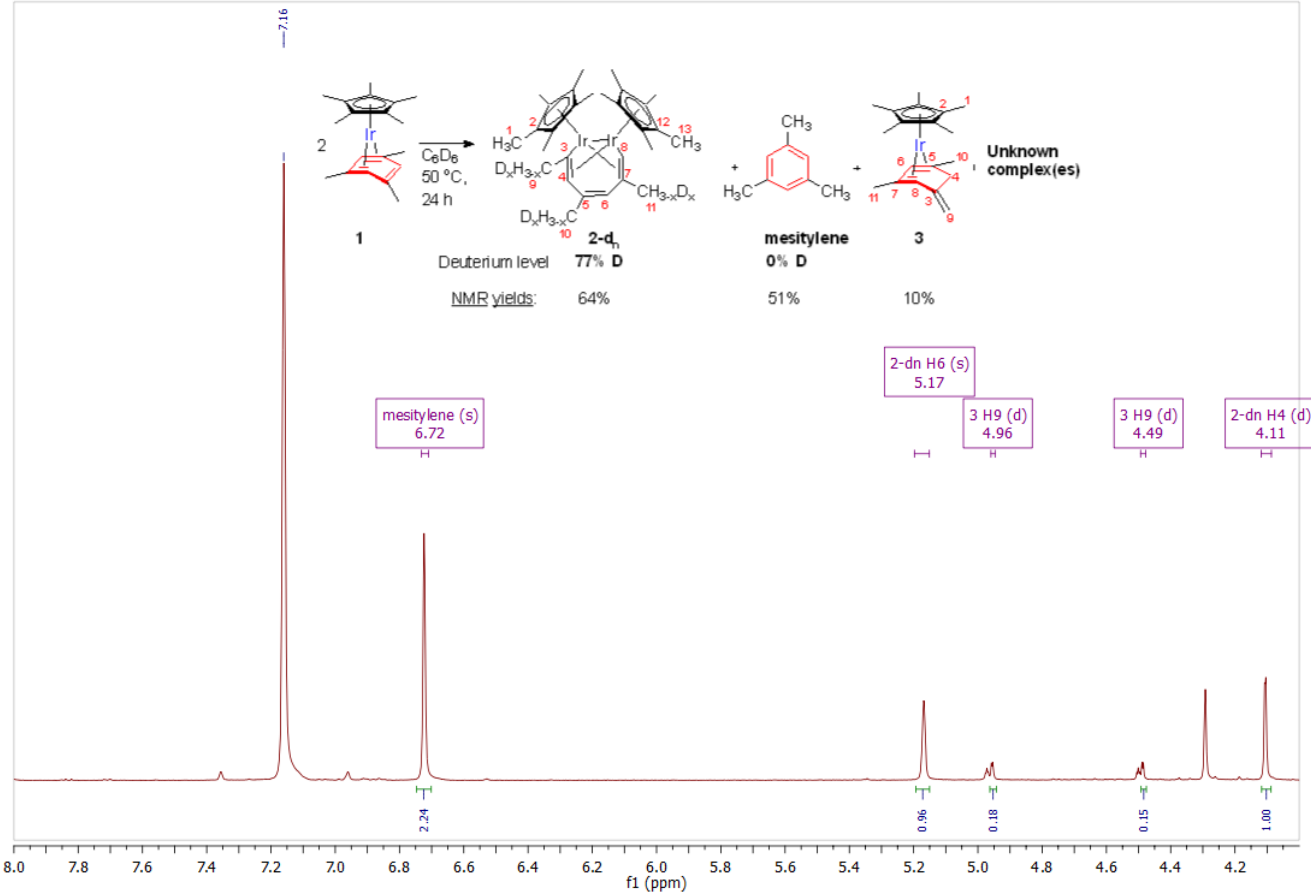

A high field region (4 to 0 ppm) of the ^{1}H NMR spectrum of the reaction mixture of thermal cleavage of **1** in C_6D_6 at full conversion of **1**.

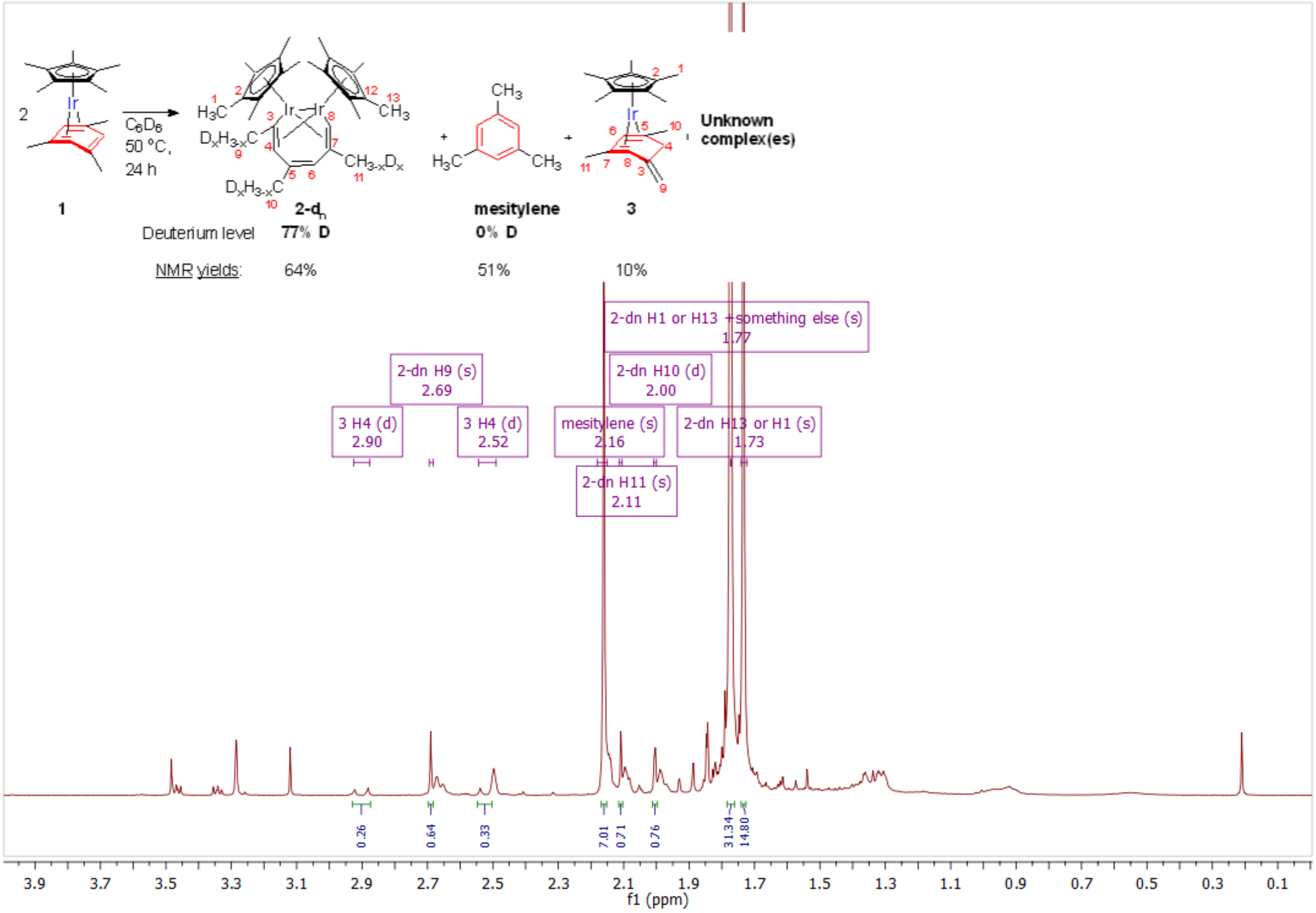

Overlaid ^{1}H NMR spectra of **2** in C_6D_6 and the reaction mixture of thermal cleavage of **1** in C_6D_6 at full conversion of **1**.

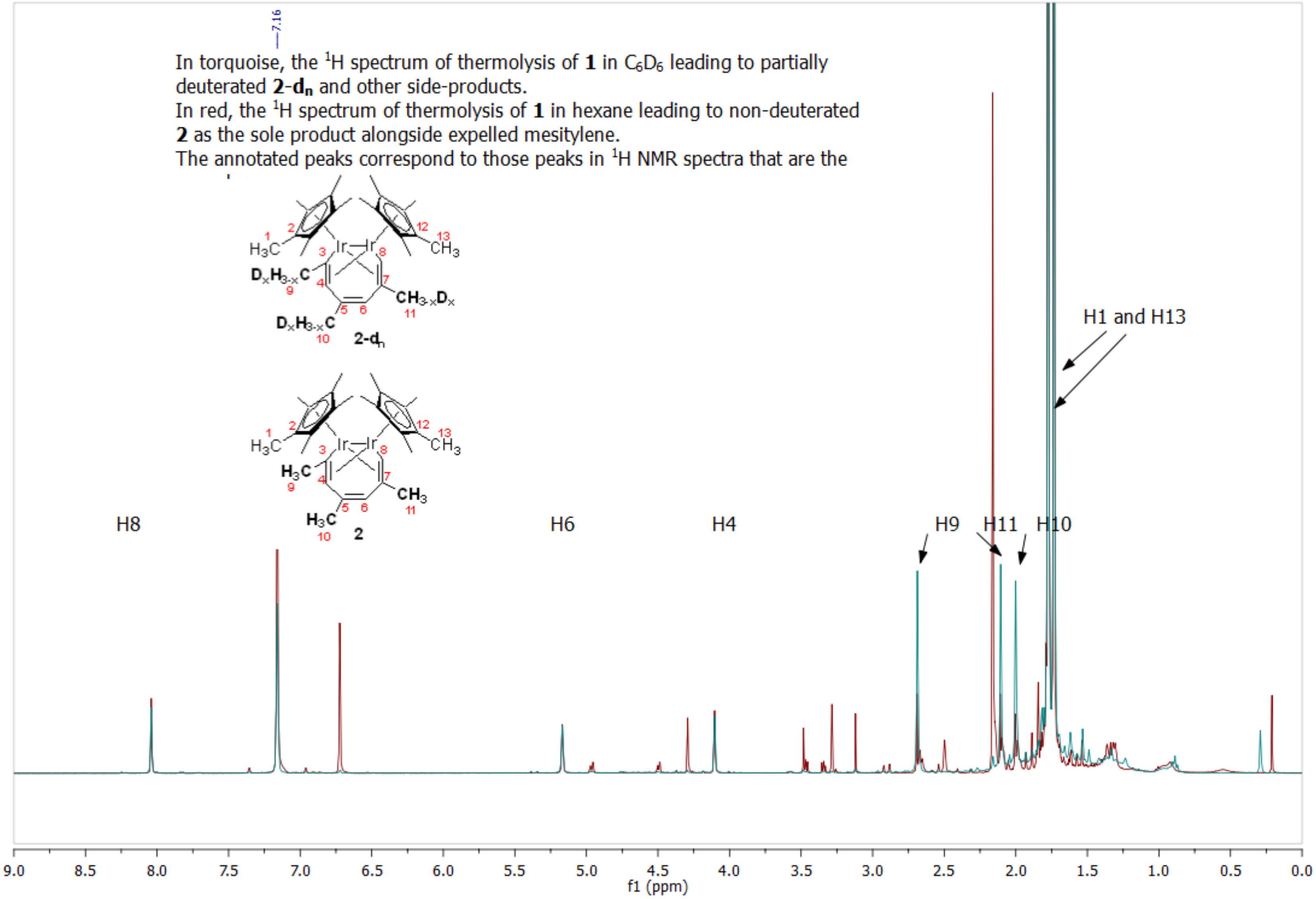

A 8.25 to 1.9 ppm region of the overlaid ^{1}H NMR spectra of **2** in C_6D_6 and the reaction mixture of thermal cleavage of **1** in C_6D_6.

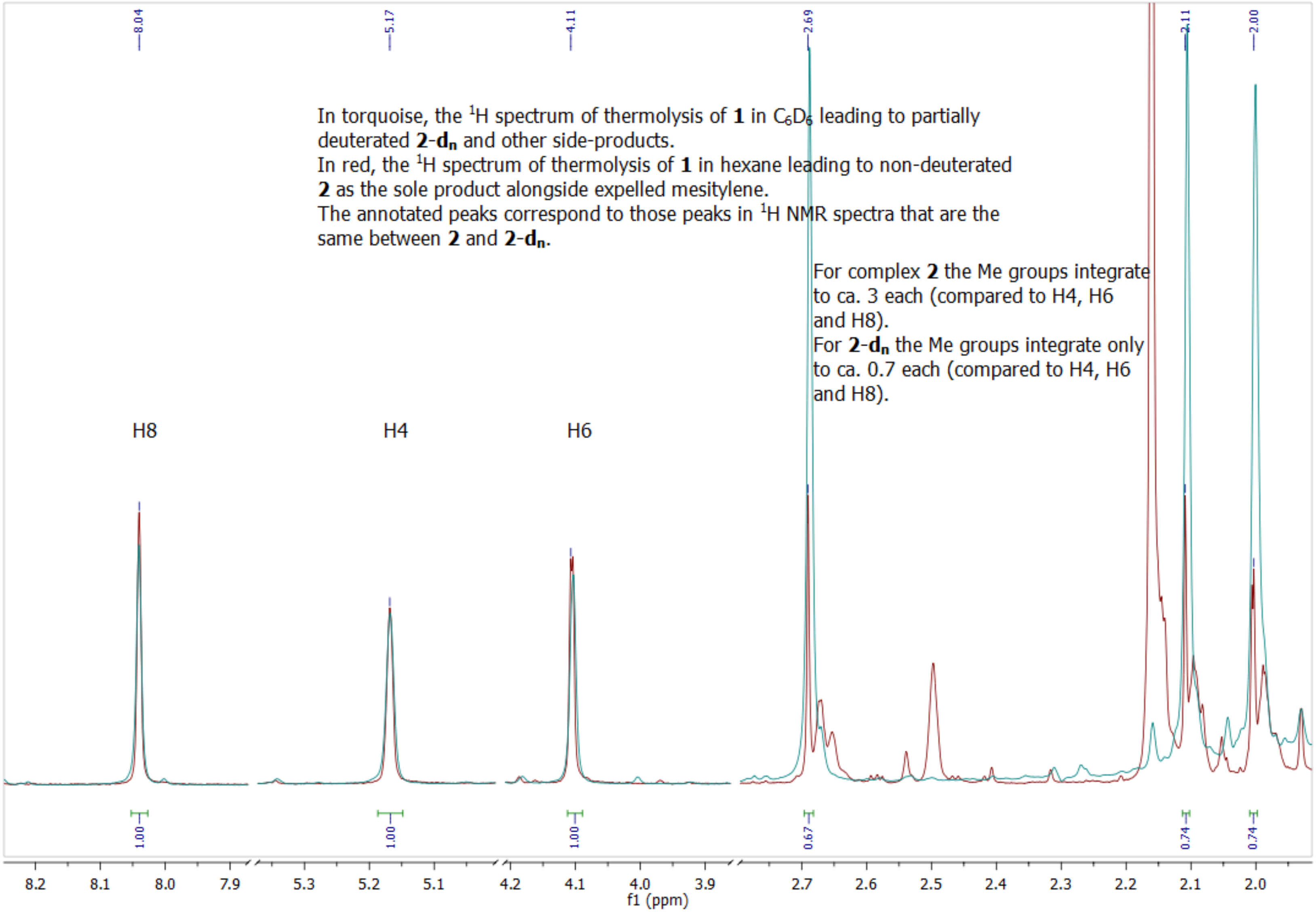

^{1}H NMR spectrum of the reaction mixture of thermal cleavage of **1** in THF-d_8 at full conversion of **1**.

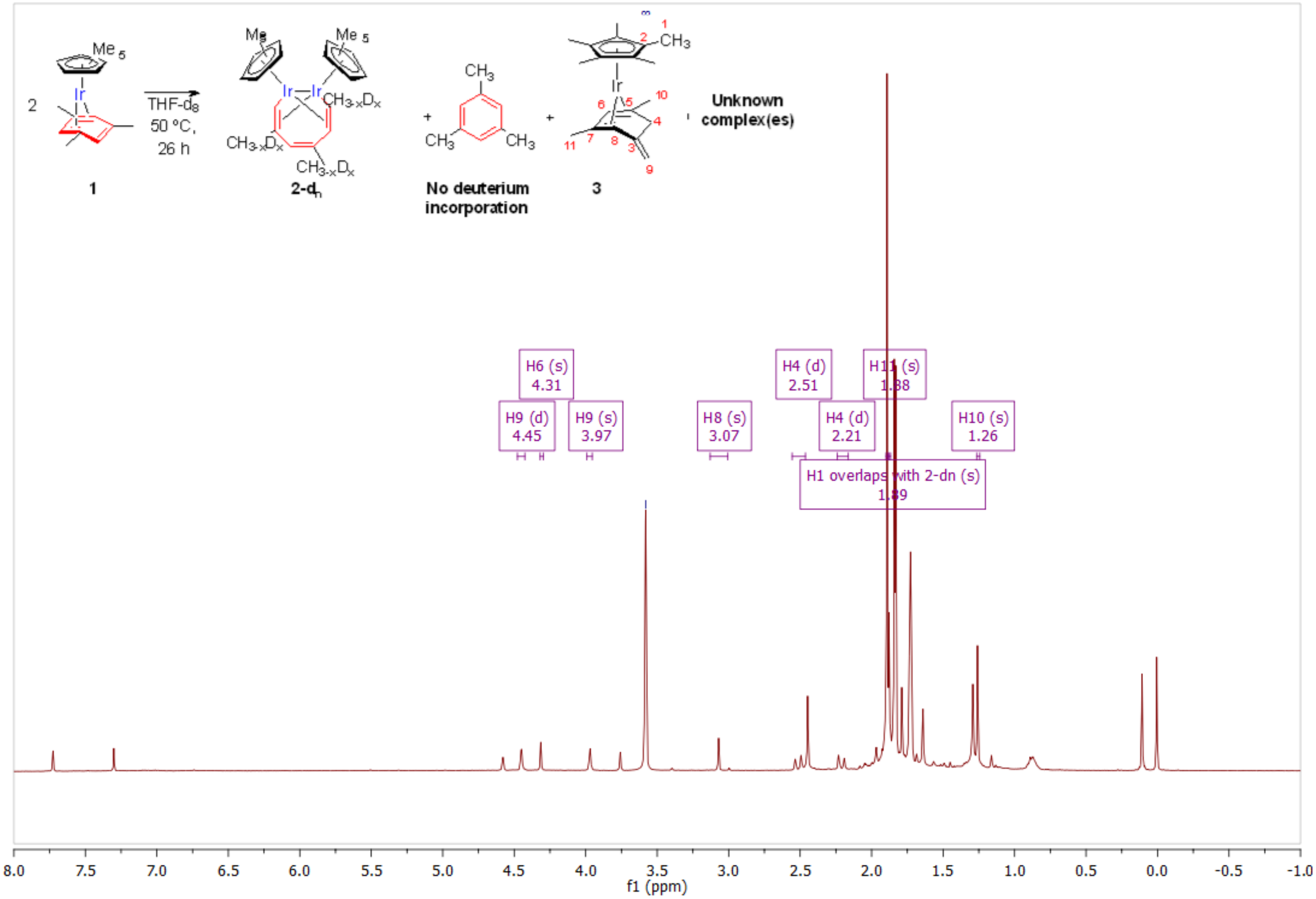

$^{13}C\{^1H\}$ NMR spectrum of the reaction mixture of thermal cleavage of **1** in THF-d_8 at full conversion of **1**.

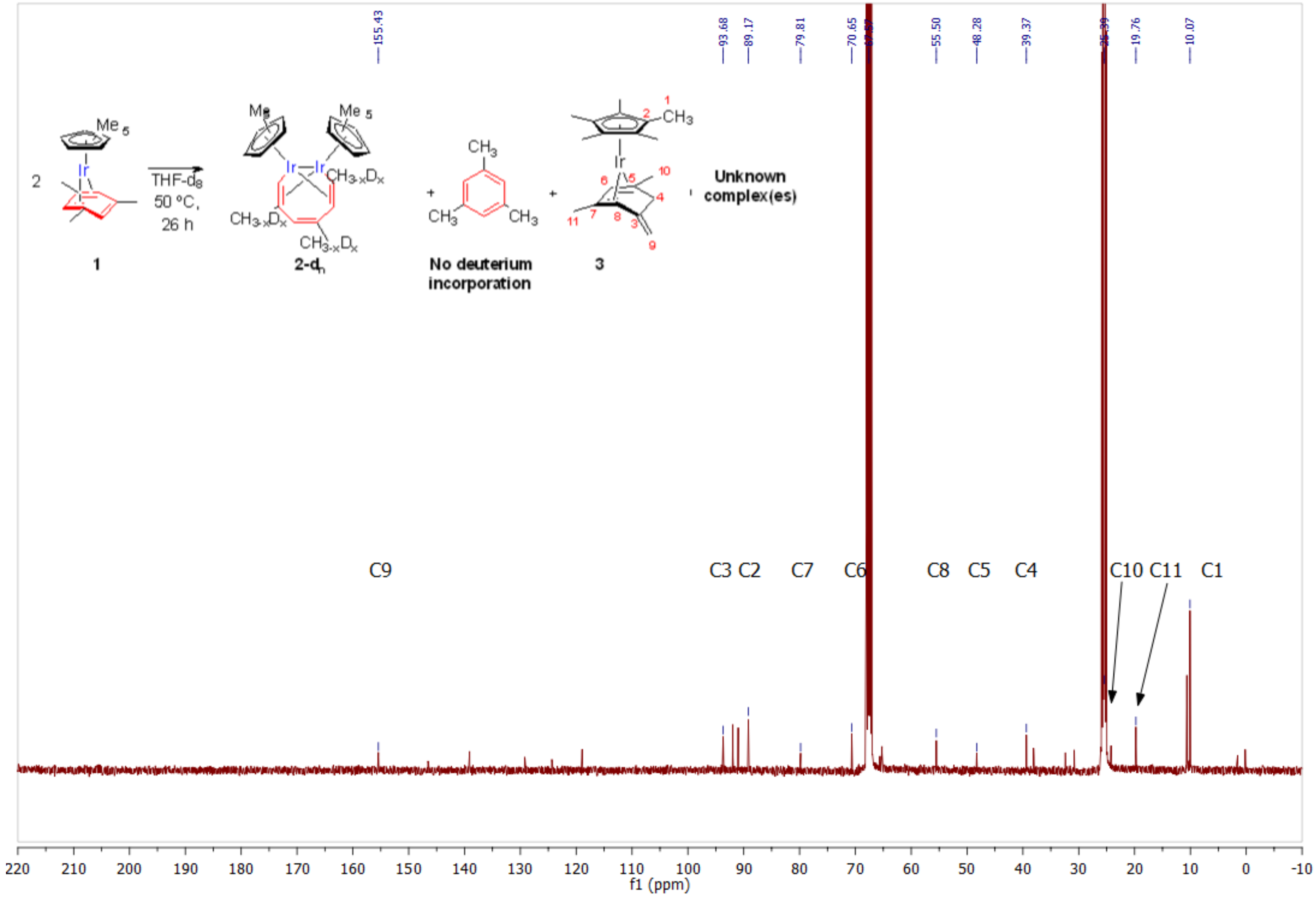

HSQC NMR spectrum of the reaction mixture of thermal cleavage of **1** in THF-d_8 at full conversion of **1**.

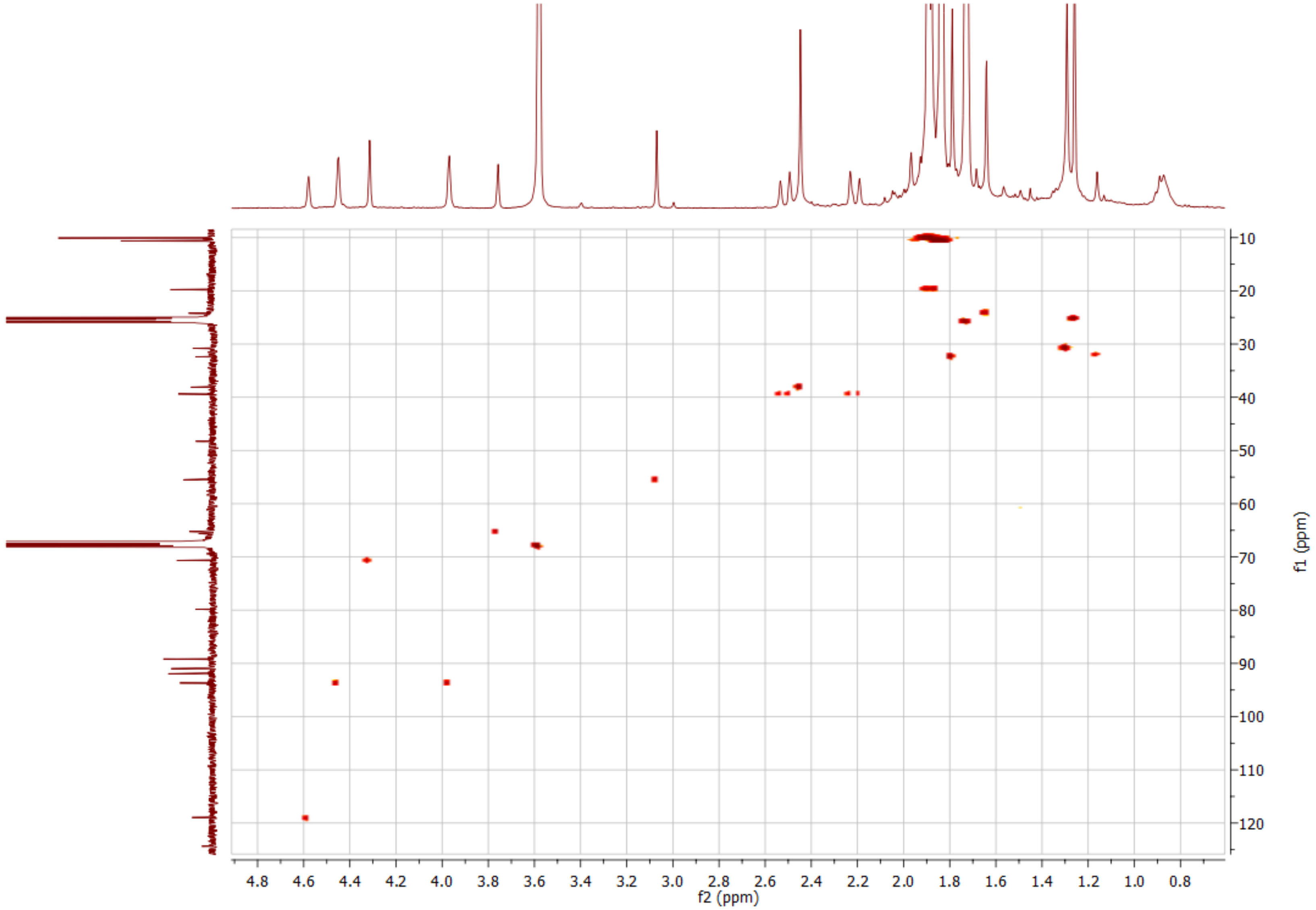

HMBC NMR spectrum of the reaction mixture of thermal cleavage of **1** in THF-d_8 at full conversion of **1**.

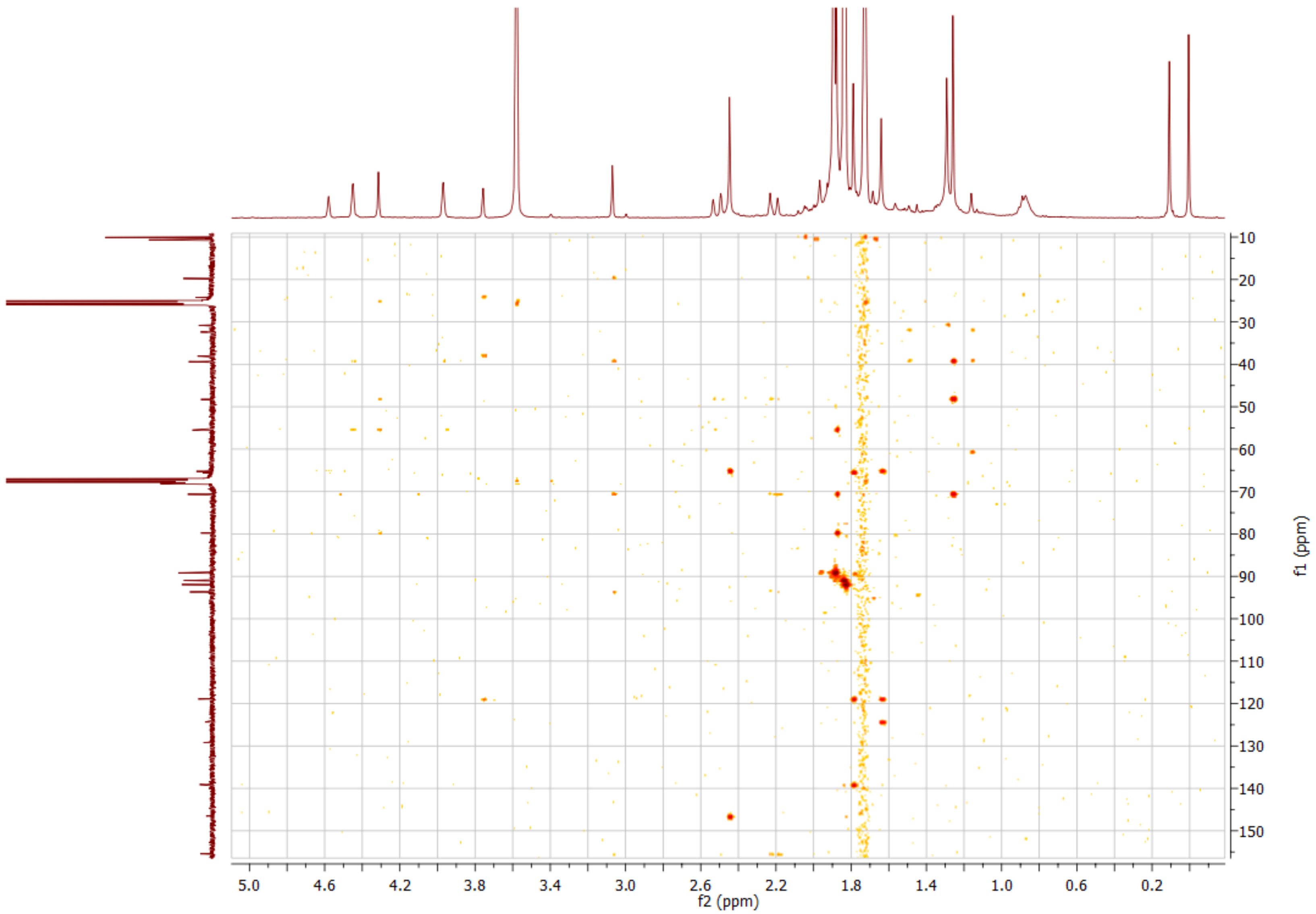

^{1}H NMR spectrum of **4** in C_6D_6.

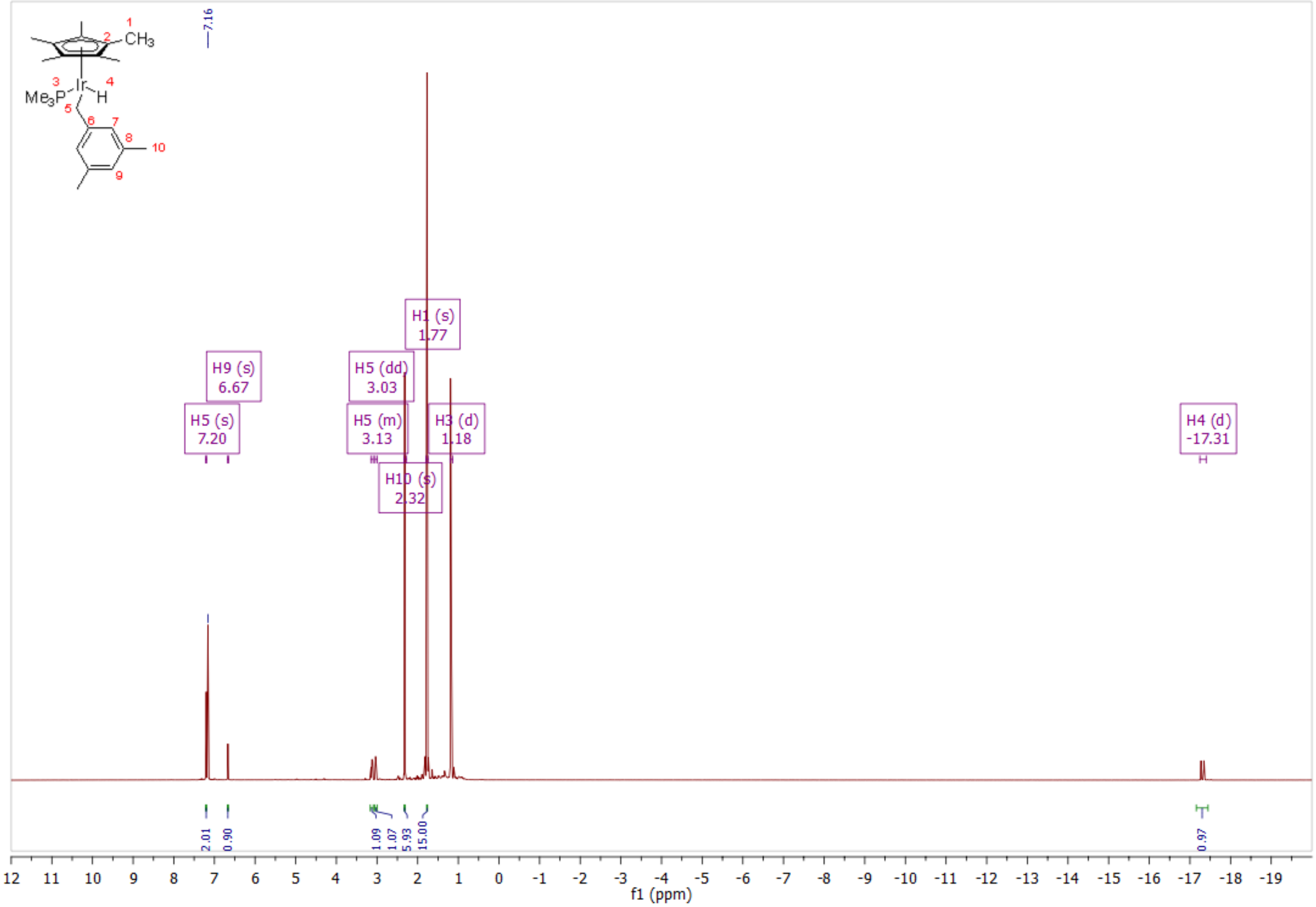

^{1}H NMR spectrum of **4** in C_6D_6.

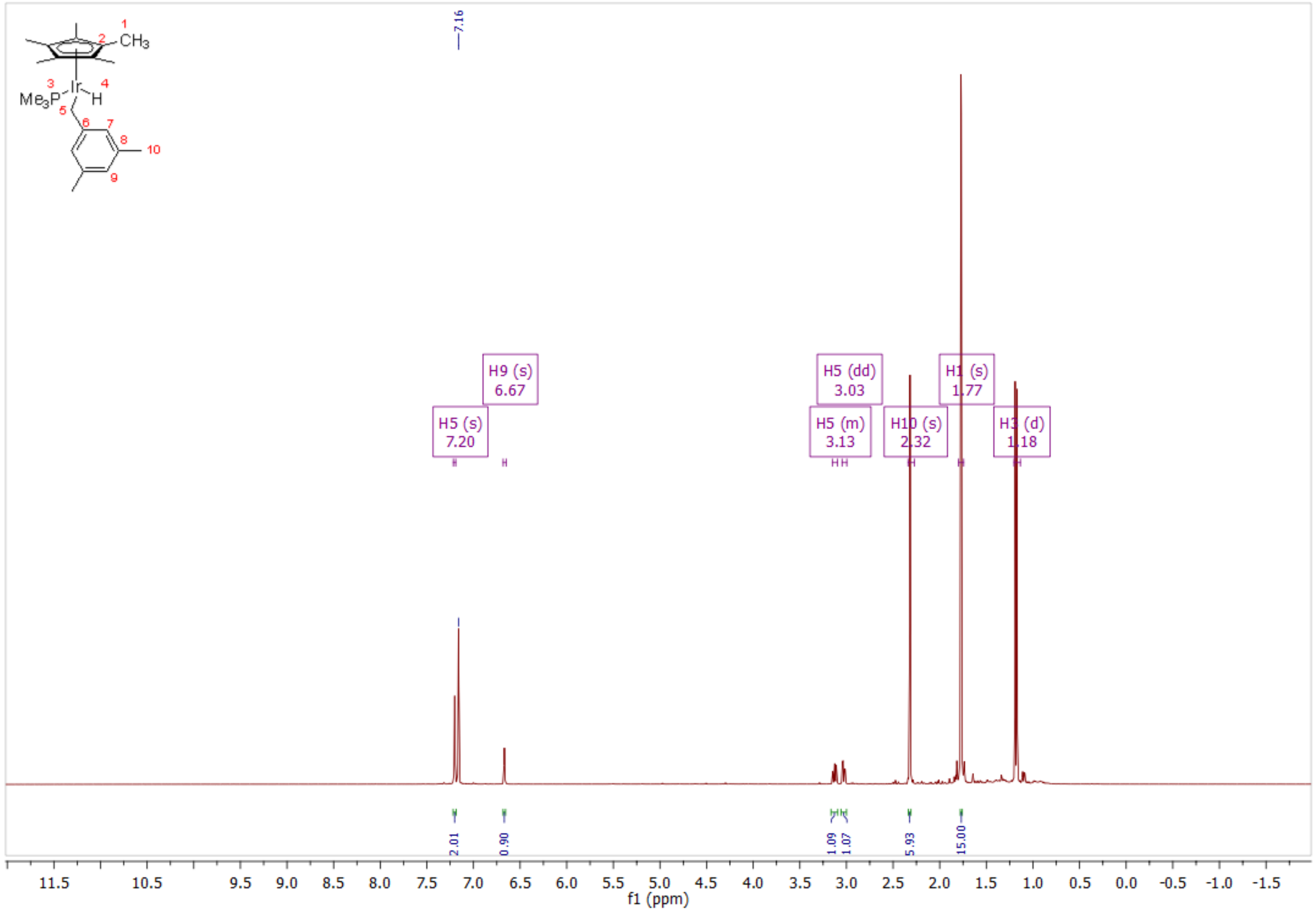

$^{13}C\{^1H\}$ NMR spectrum of **4** in C_6D_6.

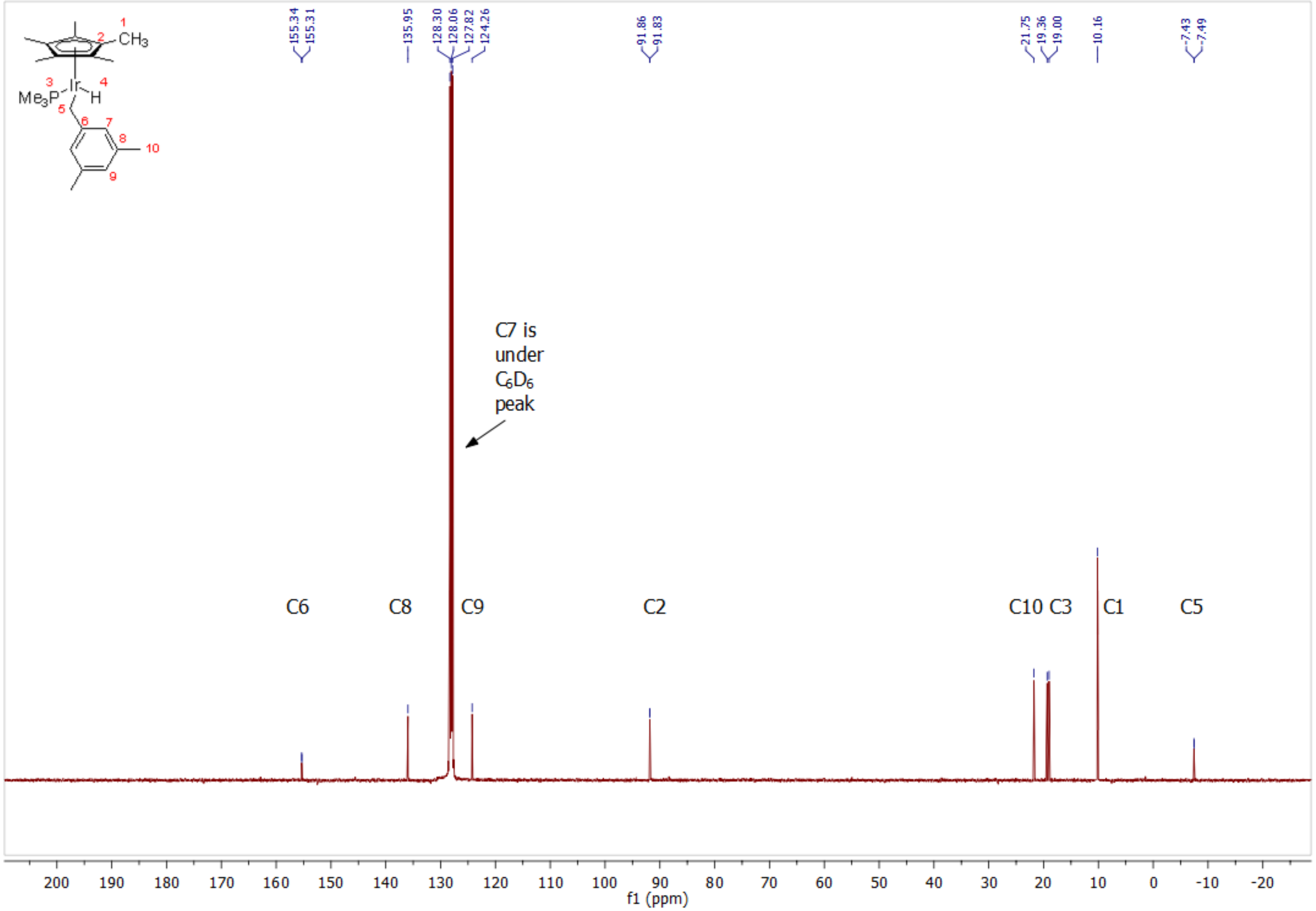

$^{31}P\{^{1}H\}$ NMR spectrum of **4** in C_6D_6

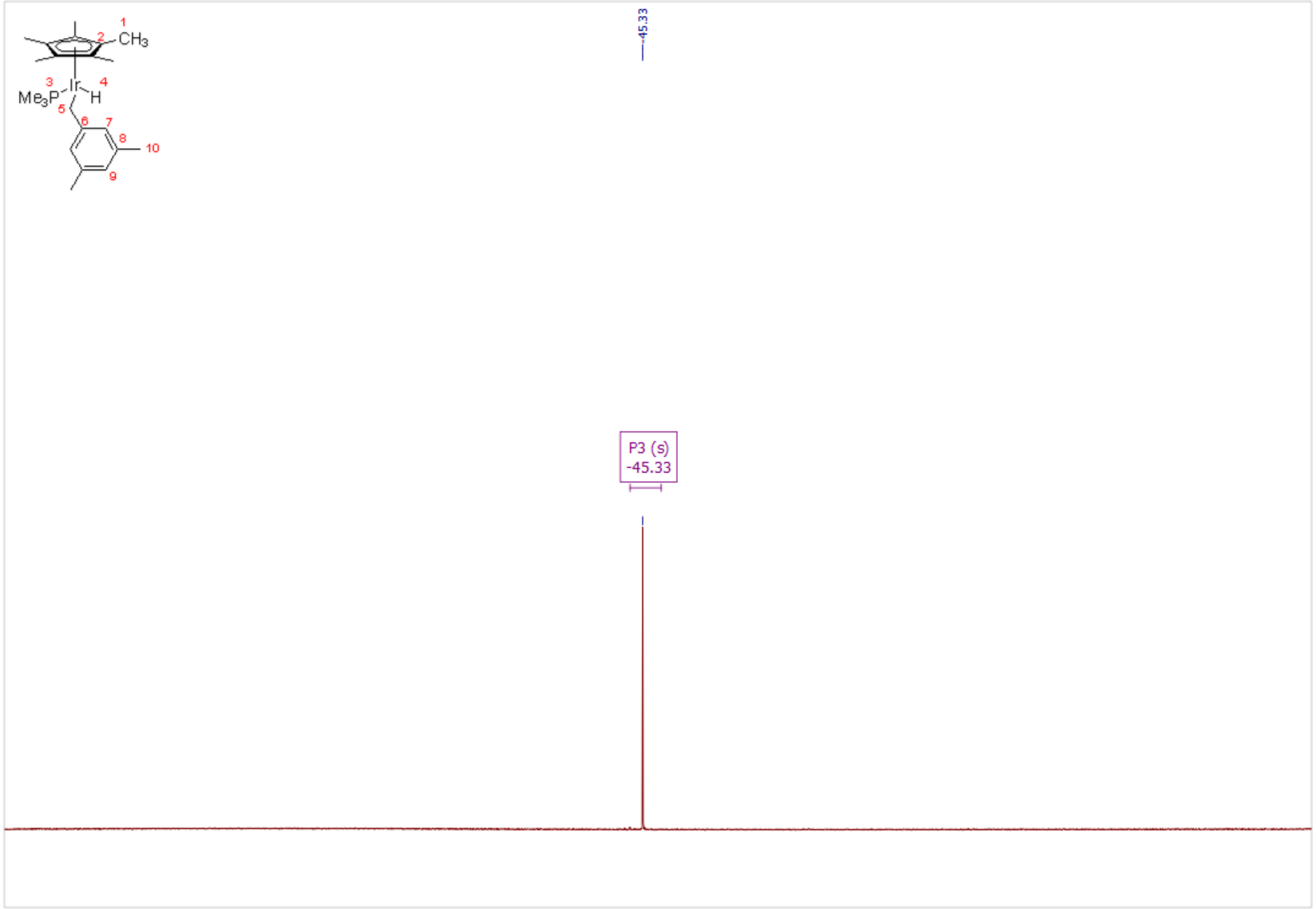

HSQC NMR spectrum of **4** in C_6D_6.

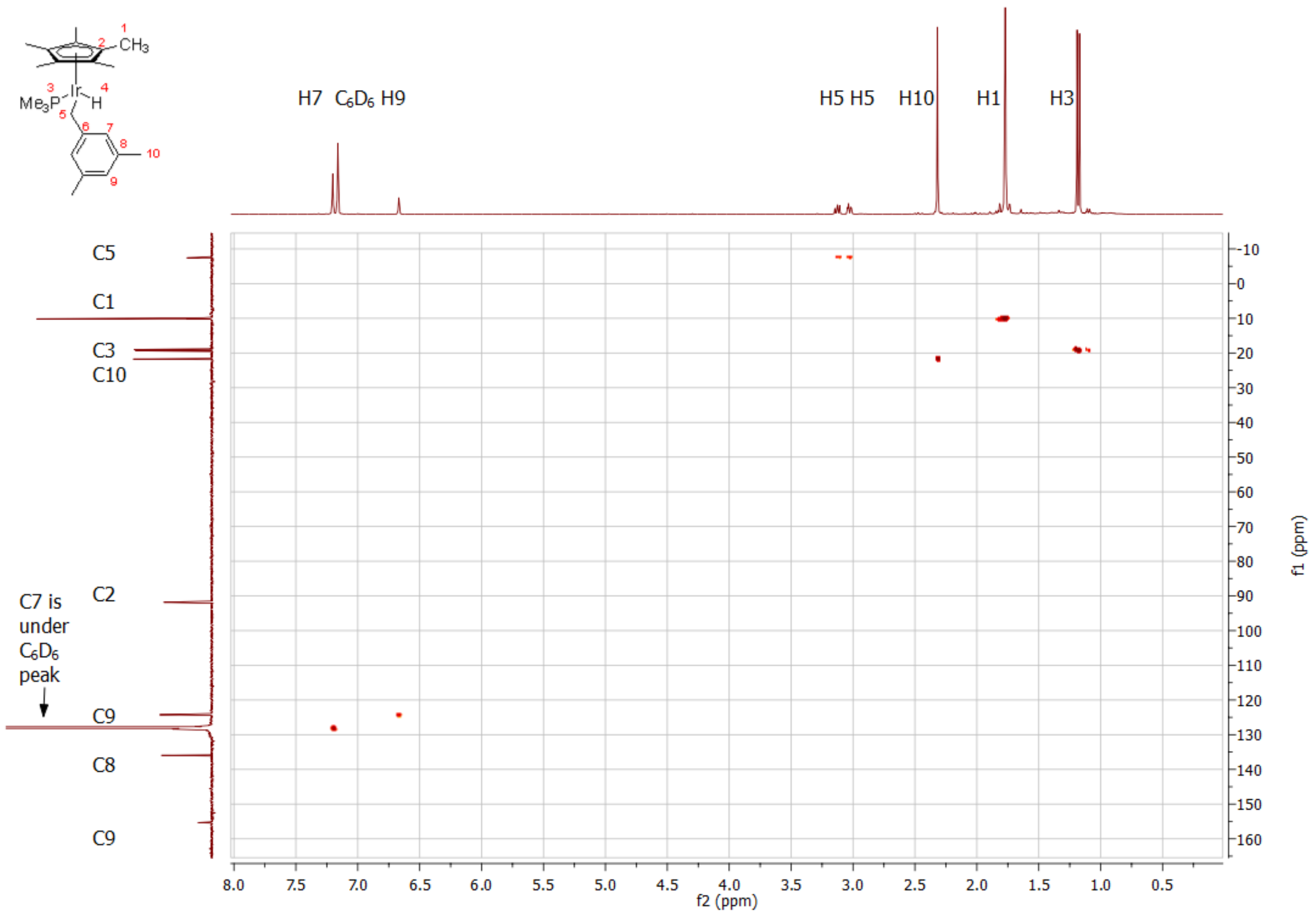

HMBC NMR spectrum of **4** in C_6D_6.

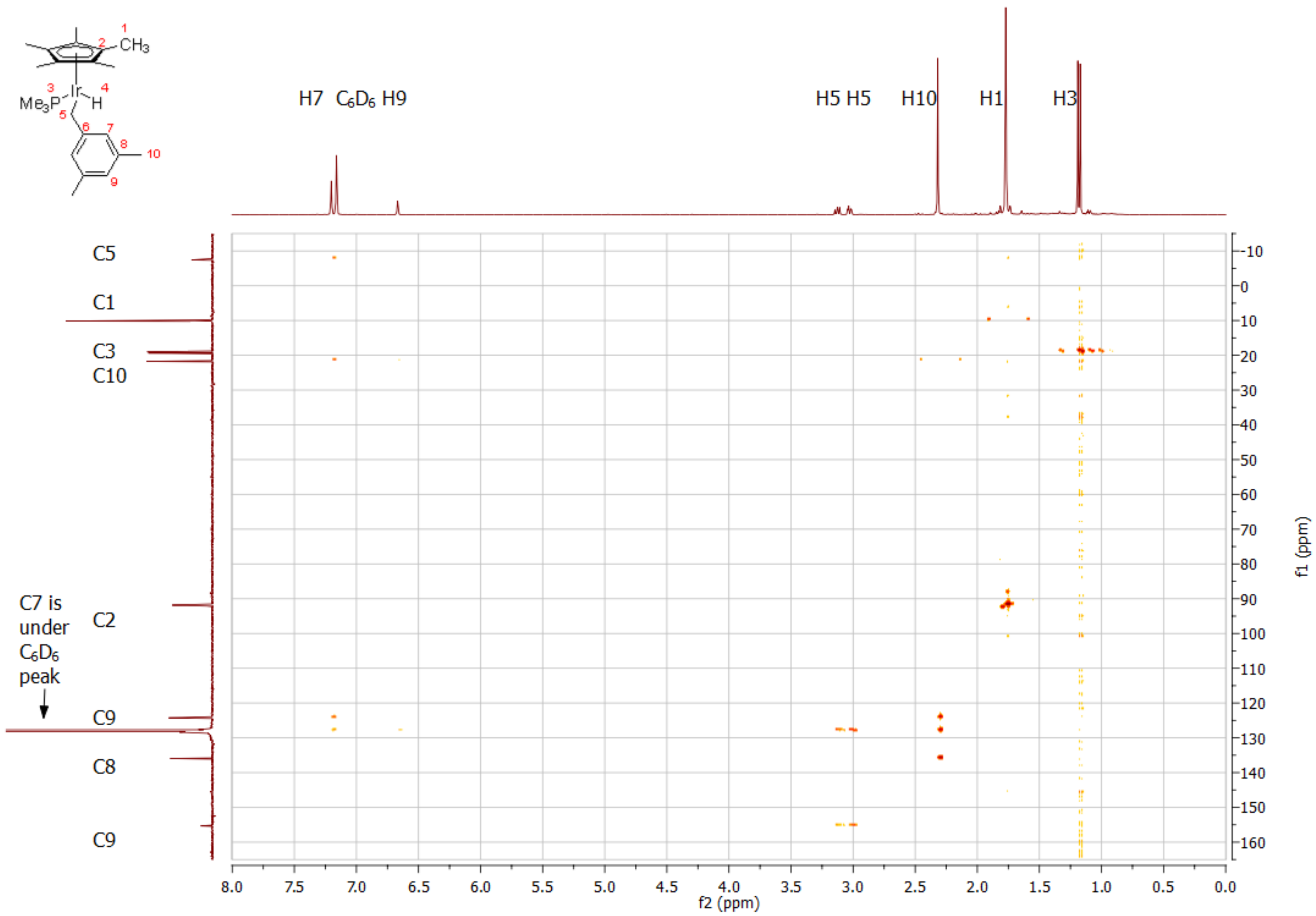

^{1}H NMR spectrum of attempted cleavage of **[Cp*Ir(η^4-C_6Me_6)]** (**39**) at 150 °C.

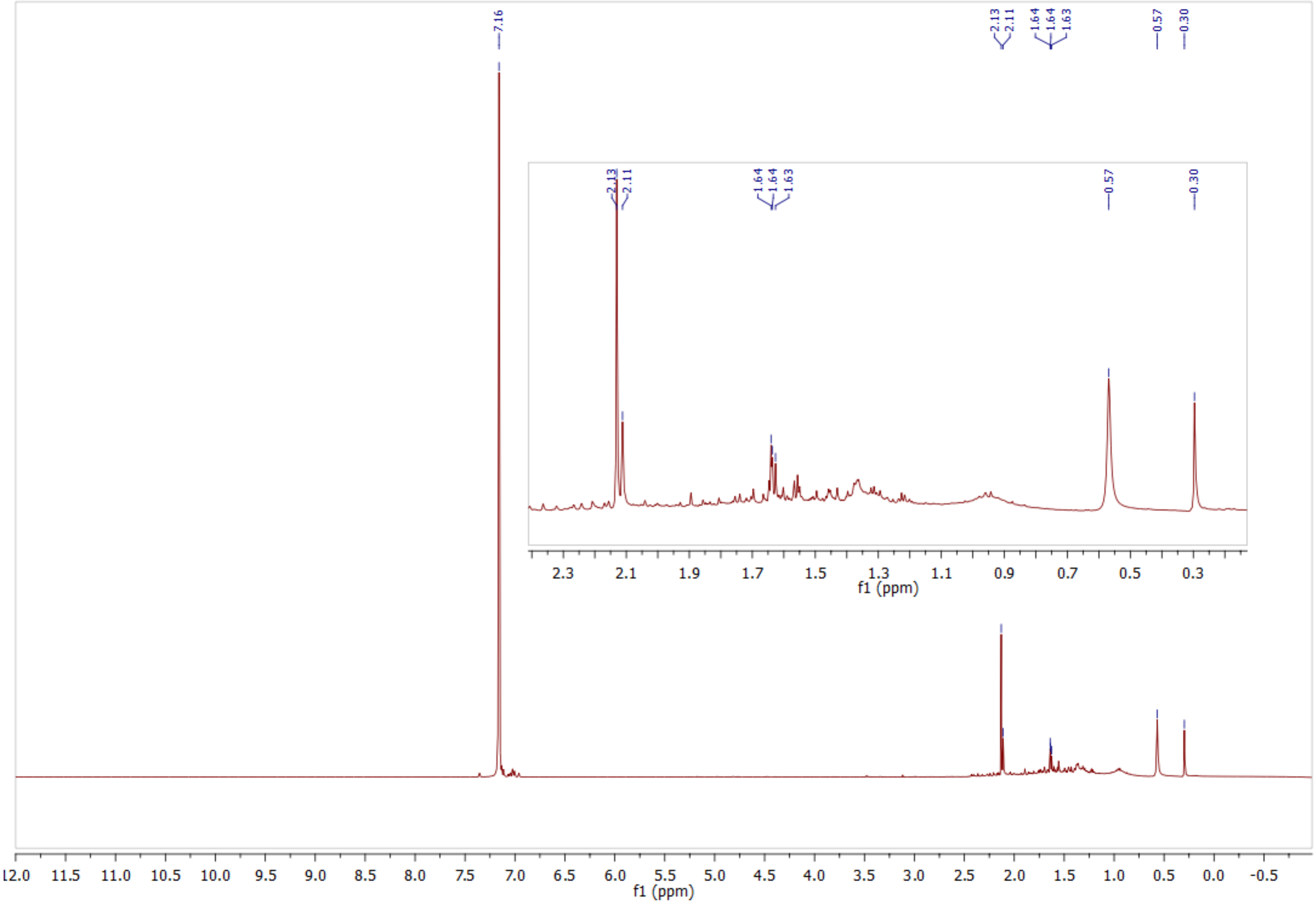

^{1}H NMR spectrum of attempted cleavage of **[Cp*Ir(η^4-naphthalene)] (40)** at 150 °C.

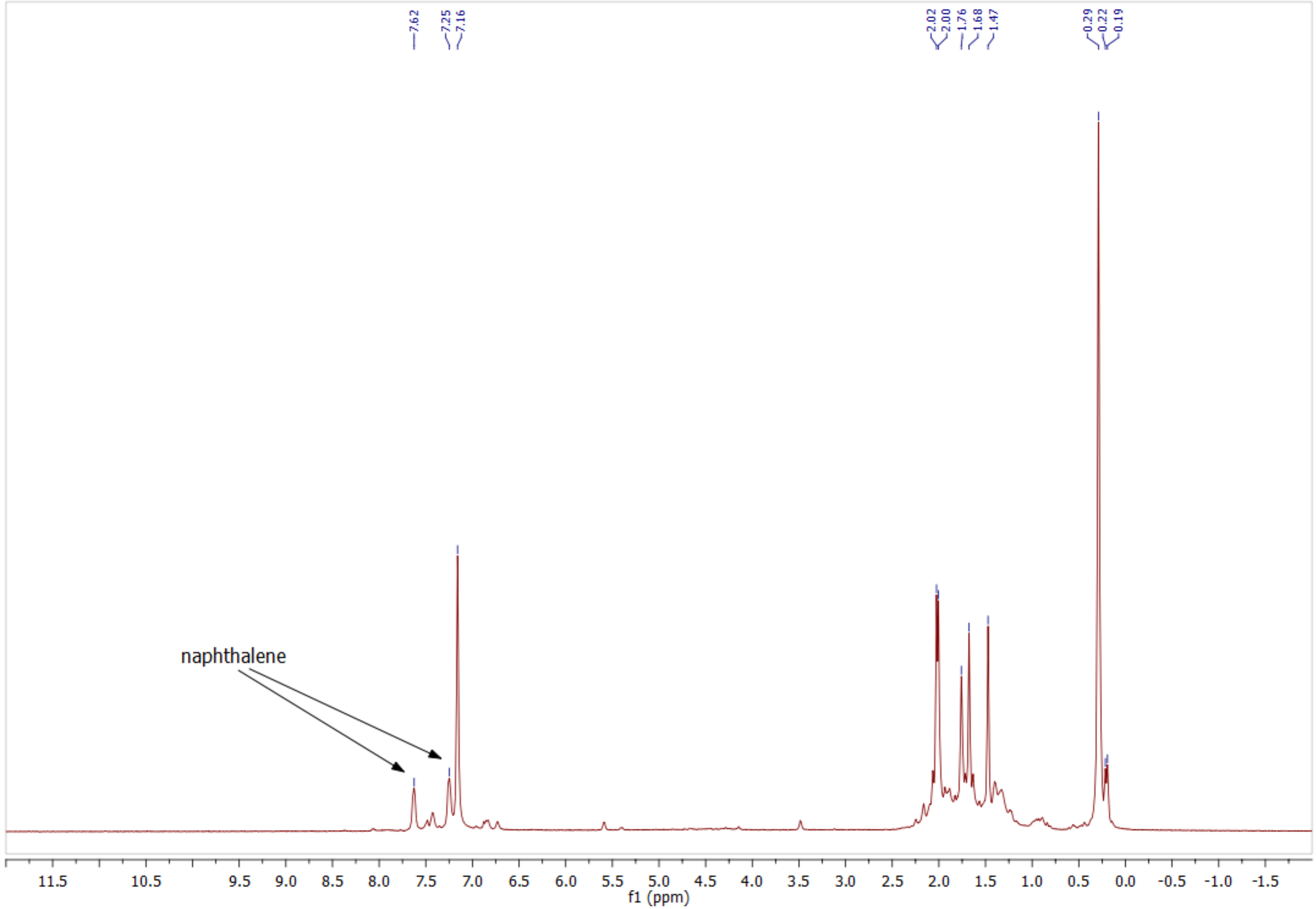

^{1}H NMR spectrum of attempted cleavage of **[Cp*Ir(η⁴-2,6-dimethylnaphthalene)]** (**41**) at 150 °C.

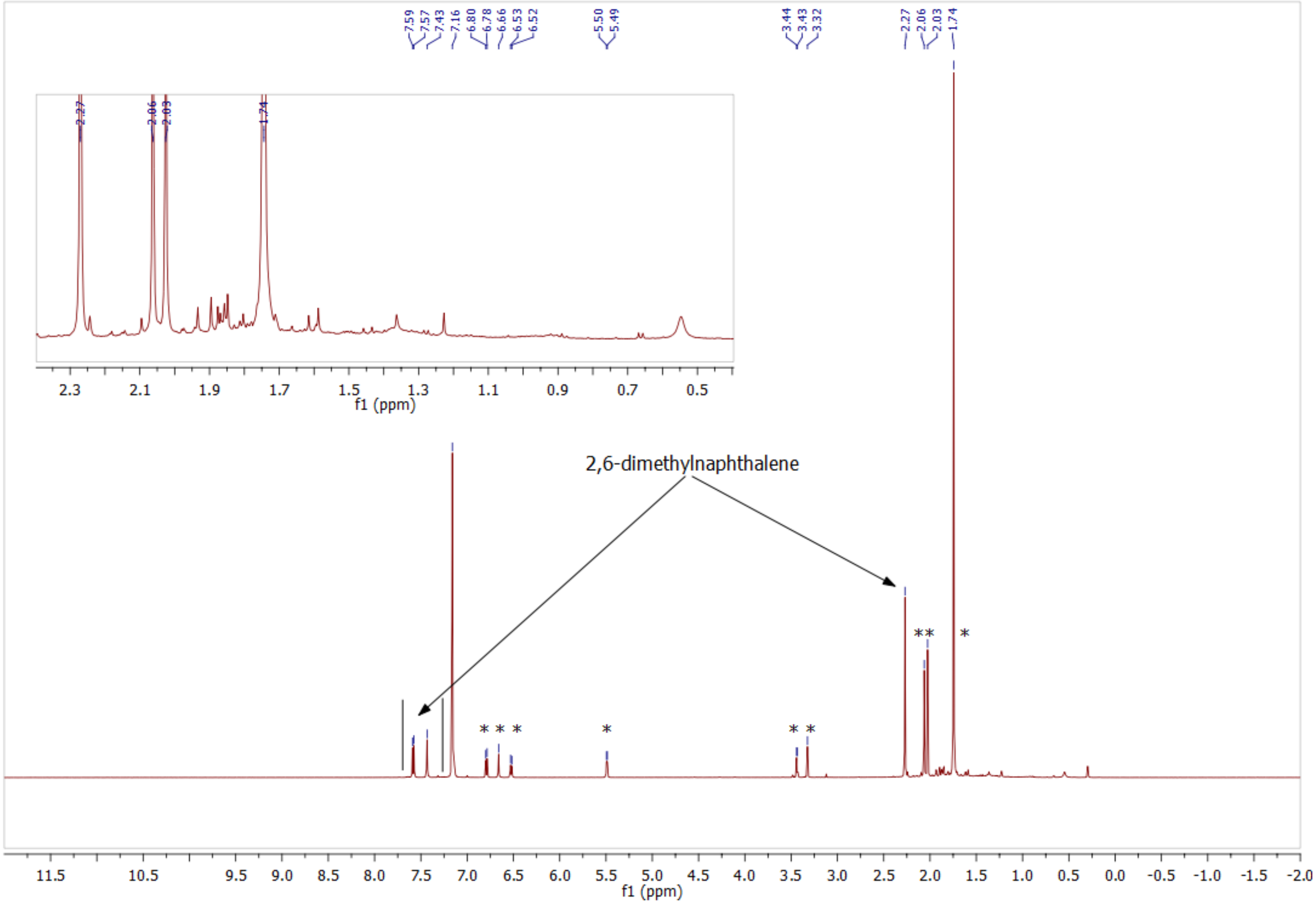

10. X-ray data

Single crystals of [Cp*Ir(η^4-C_6Me_6)] (**39**) and [Cp*Ir(η^4-naphthalene)] (**40**) were selected and mounted on a Mitegen loop using Paratone-N oil on a SuperNova, Dual, Cu at home/near, AtlasS2 diffractometer. The crystal was kept at 100.01(10) K during data collection. Using Olex2[13], the structures were solved with the ShelXT[14] structure solution program using Intrinsic Phasing and refined with the ShelXL[15] refinement package using Least Squares minimisation. The twin law for **39** was determined using CrysAlisPRO[16] and was found to be a 180 degree rotation about 1 0 0 (dir), or alternatively the twin matrix is: (1 0 0, 0 -1 0, -0.19 0 -1) and the refined mass fractions for the two components determined as 0.5285(12) and 0.4715(12).

Table S20. Crystallographic data and structure refinement for η^4-complexes.

Complex	[Cp*Ir(η^4-C_6Me_6)] (**39**)	[Cp*Ir(η^4-naphthalene)] **40**)
CCDC number*	2040076	2040077
Empirical formula	$C_{22}H_{33}Ir$	$C_{40}H_{46}Ir_2$
Formula weight	489.68	911.17
Temperature/K	100.01(10)	100.01(10)
Crystal system	monoclinic	monoclinic
Space group	$P2_1/n$	Cc
a/Å	8.01647(19)	22.2884(4)
b/Å	16.3246(4)	22.2818(4)
c/Å	15.0490(4)	26.1267(5)
α/°	90	90
β/°	92.985(2)	97.5244(17)
γ/°	90	90
Volume/Å^3	1966.73(9)	12863.5(4)
Z	4	16
ρ_{calc}g/cm^3	1.654	1.882
μ/mm^{-1}	13.098	8.295
F(000)	968.0	7040.0
Crystal size/mm^3	0.26 × 0.106 × 0.071	0.08 × 0.06 × 0.04
Radiation	CuKα (λ = 1.54184)	MoKα (λ = 0.71073)
2Θ range for data collection/°	7.996 to 153.248	4.822 to 59.546
Index ranges	$-10 \leq h \leq 10$, $-20 \leq k \leq 20$, $-18 \leq l \leq 18$	$-28 \leq h \leq 30$, $-29 \leq k \leq 28$, $-36 \leq l \leq 33$
Reflections collected	5009	96652
Independent reflections	5009 [R_{merg} = , R_{sigma} = 0.0187]	31301 [R_{int} = 0.0334, R_{sigma} = 0.0416]
Data/restraints/parameters	5009/12/214	31301/388/1553
Goodness-of-fit on F^2	1.084	0.998

Final R indexes [I>=2σ (I)]	R_1 = 0.0342, wR_2 = 0.0893	R_1 = 0.0264, wR_2 = 0.0473
Final R indexes [all data]	R_1 = 0.0379, wR_2 = 0.0911	R_1 = 0.0433, wR_2 = 0.0530
Largest diff. peak/hole / e $Å^{-3}$	1.59/-1.42	2.56/-0.92
Flack parameter		-0.015(5)

*Crystallographic files can be obtained free of charge from: ccdc.cam.ac.uk.

Table 21. Selected C-C bond lengths and angles for [Cp*Ir(η^4- C_6Me_6)] (**39**) and [Cp*Ir(η^4- naphthalene)] (**40**).

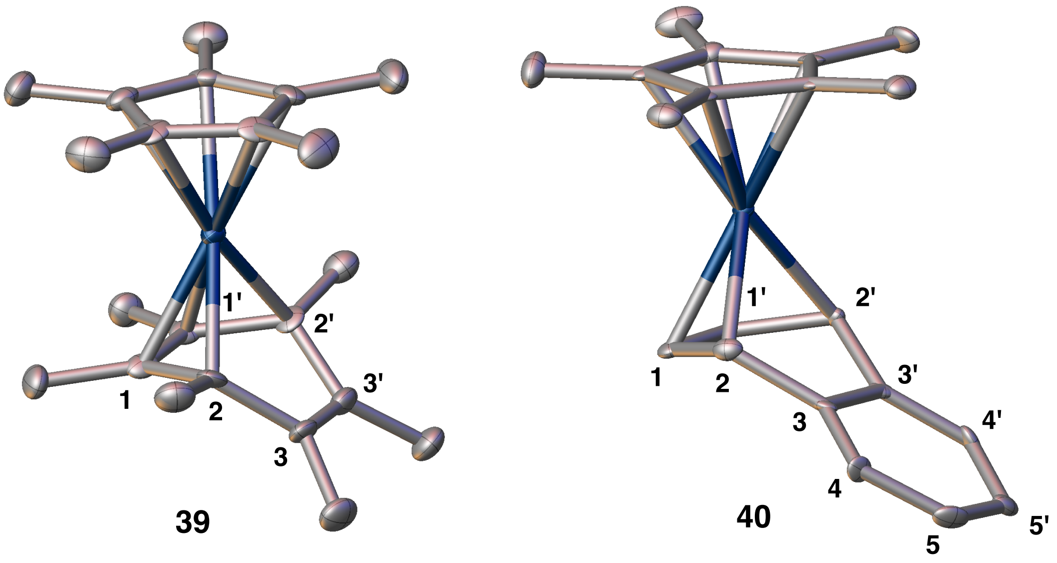

[Cp*Ir(η^4-C_6Me_6)] (**39**)		[Cp*Ir(η^4-naphthalene)] (**40**)	
	Bond lengths, Å		Bond lengths, Å *
C1-C1’	1.435(8)	C1-C1’	1.423(13)
C1-C2	1.487(8)	C1-C2	1.460(12)
C2-C3	1.513(8)	C2-C3	1.487(12)
C3-C3’	1.328(9)	C3-C3’	1.406(12)
C1’-C2’	1.463(8)	C3-C4	1.385(13)
C2’-C3’	1.511(8)	C4-C5	1.391(14)
		C5-C5’	1.381(14)
Bending angle α (torsion angle C3-C2-C1-C1’), °		Bending angle α (torsion angle C3-C2-C1-C1’), °	
45.9		43.7	

*Selected bond lengths are from only one out of eight molecules present in unit cell.

11. References

1. Jakoobi, M.; Halcovitch, N.; Whitehead, G. F. S.; Sergeev, A. G., *Angew. Chem. Int. Ed.* **2017,** *56*, 3266-3269.
2. Rybinskaya, M. I.; Kudinov, A. R.; Kaganovich, V. S., *J. Organomet. Chem.* **1983,** *246*, 279-285.
3. Chen, T. S.; Wolinska ‐ Mocydlarz, J.; Leitch, L. C., *J. Labelled Compd.* **1970,** *6*, 285-288.
4. Maitlis, P. M.; White, C., *J. Chem. Soc. A* **1971**, 3322-3326.
5. White, C.; Thompson, S. J.; Maitlis, P. M., *J. Chem. Soc. Dalton Trans.* **1977**, 1654-1661.
6. Shubin, V. G.; Berezina, R. N.; N., P. V., *J. Organomet. Chem.* **1973,** *54*, 239-246.
7. (a) Becke, A. D., *J. Chem. Phys.* **1993,** *98*, 5648-5652; (b) Lee, C. T.; Yang, W. T.; Parr, R. G., *Phys. Rev. B* **1988,** *37*, 785-789.
8. Zhao, Y.; Truhlar, D. G., *J. Chem. Phys.* **2006,** *125*, 194101.
9. Ribeiro, R. F.; Marenich, A. V.; Cramer, C. J.; Truhlar, D. G., *J. Phys. Chem. B* **2011,** *115*, 14556-14562.
10. (a) Liu, P.; Xu, X. F.; Dong, X. F.; Keitz, B. K.; Herbert, M. B.; Grubbs, R. H.; Houk, K. N., *J. Am. Chem. Soc.* **2012,** *134*, 1464-1467; (b) Lin, M.; Kang, G. Y.; Guo, Y. A.; Yu, Z. X., *J. Am. Chem. Soc.* **2012,** *134*, 398-405; (c) Hong, S. Y.; Park, Y.; Hwang, Y.; Kim, Y. B.; Baik, M. H.; Chang, S., *Science* **2018,** *359*, 1016-1021; (d) Gusev, D. G., *Organometallics* **2013,** *32*, 4239-4243; (e) Hopmann, K. H., *Organometallics* **2016,** *35*, 3795-3807.
11. Zhao, Y.; Truhlar, D. G., *Theor. Chem. Account.* **2008,** *120*, 215-241.
12. Gaussian 09, Revision E.01, Frisch, M. J.; Trucks, G. W.; Schlegel, H. B.; Scuseria, G. E.; Robb, M. A.; Cheeseman, J. R.; Scalmani, G.; Barone, V.; Mennucci, B.; Petersson, G. A.; Nakatsuji, H.; Caricato, M.; Li, X.; Hratchian, H. P.; Izmaylov, A. F.; Bloino, J.; Zheng, G.; Sonnenberg, J. L.; Hada, M.; Ehara, M.; Toyota, K.; Fukuda, R.; Hasegawa, J.; Ishida, M.; Nakajima, T.; Honda, Y.; Kitao, O.; Nakai, H.; Vreven, T.; Montgomery, J. A., Jr.; Peralta, J. E.; Ogliaro, F.; Bearpark, M.; Heyd, J. J.; Brothers, E.; Kudin, K. N.; Staroverov, V. N.; Kobayashi, R.; Normand, J.; Raghavachari, K.; Rendell, A.; Burant, J. C.; Iyengar, S. S.; Tomasi, J.; Cossi, M.; Rega, N.; Millam, J. M.; Klene, M.; Knox, J. E.; Cross, J. B.; Bakken, V.; Adamo, C.; Jaramillo, J.; Gomperts, R.; Stratmann, R. E.; Yazyev, O.; Austin, A. J.; Cammi, R.; Pomelli, C.; Ochterski, J. W.; Martin, R. L.; Morokuma, K.; Zakrzewski, V. G.; Voth, G. A.; Salvador, P.; Dannenberg, J. J.; Dapprich, S.; Daniels, A. D.; Farkas, Ö.; Foresman, J. B.; Ortiz, J. V.; Cioslowski, J.; Fox, D. J. Gaussian, Inc., Wallingford CT, 2015.
13. Dolomanov, O. V.; Bourhis, L. J.; Gildea, R. J.; Howard, J. A. K.; Puschmann, H., *J.Appl. Cryst.* **2009,** *42*, 339-341.
14. Sheldrick, G. M., *Acta Cryst. A* **2015,** *71*, 3-8.
15. Sheldrick, G. M., *Acta Cryst. C* **2015,** *71*, 3-8.
16. CrysAlisPRO, Oxford Diffraction /Agilent Technologies UK Ltd, Yarnton, England.
